# Contribution of Maternal Adherence to the Effect of Multiple Micronutrient Supplementation During Pregnancy: A Systematic Review and Individual Participant Data Meta-analysis

**DOI:** 10.1016/j.advnut.2025.100455

**Published:** 2025-05-30

**Authors:** Emily R Smith, Filomena Gomes, Seth Adu-Afarwuah, Victor M Aguayo, Shams El Arifeen, Zulfiqar A Bhutta, Ellen C Caniglia, Parul Christian, Delanjathan Devakumar, Kathryn G Dewey, Wafaie W Fawzi, Henrik Friis, Exnevia Gomo, Ousmane Guindo, Lotta Hallamaa, Sheila Isanaka, Pernille Kæstel, Carl Lachat, Ken Maleta, Sophie E Moore, Erin M Oakley, David Osrin, Anisur Rahman, Ziaul Rana, Arjumand Rizvi, Dominique Roberfroid, Saijuddin Shaikh, Bakary Sonko, Sajid Bashir Soofi, Inan Subarkah, Rahardjo Sunawang, Dongqing Wang, Keith P West, Lee Shu Fune Wu, Noel Zagre, Megan W Bourassa, Christopher R Sudfeld

**Affiliations:** 1 Department of Global Health, Exercise and Nutrition Sciences, Milken Institute School of Public Health, The George Washington University, Washington, DC, United States; 2 Nutrition Science Program, The New York Academy of Science, New York, NY, United States; 3 NOVA Medical School, Universidade NOVA de Lisboa, Lisboa, Portugal; 4 Healthy Mothers Healthy Babies Program, Micronutrient Forum, Washington, DC, United States; 5 Department of Nutrition and Food Science, University of Ghana, Legon, Accra, Ghana; 6 Child Nutrition and Development, UNICEF, New York, NY, United States; 7 Maternal and Child Health Division, International Centre for Diarrhoeal Diseases Research Bangladesh (ICDDR,B), Dhaka, Bangladesh; 8 Center of Excellence in Women and Child Health, The Aga Khan University, Karachi, Pakistan; 9 Department of Pediatrics & Child Health, The Aga Khan University, Karachi, Pakistan; 10 Institute of Global Health & Development, The Aga Khan University, Karachi, Pakistan; 11 Perelman School of Medicine, University of Pennsylvania, Philadelphia, PA, United States; 12 Center for Human Nutrition, Department of International Health, Johns Hopkins Bloomberg School of Public Health, Baltimore, MD, United States; 13 The JiVitA Project, Johns Hopkins University in Bangladesh, Gaibandha, Bangladesh; 14 Institute for Global Health, University College London, London, United Kingdom; 15 Department of Nutrition and Institute for Global Nutrition, University of California, Davis, Davis, CA, United States; 16 Department of Global Health and Population, Harvard T.H. Chan School of Public Health, Boston, MA, United States; 17 Department of Nutrition Exercise and Sports, University of Copenhagen, Copenhagen, Denmark; 18 College of Health Sciences, University of Zimbabwe, Harare, Zimbabwe; 19 Epicentre, Maradi, Niger; 20 Center for Child, Adolescent and Maternal Health Research, Faculty of Medicine and Health Technology, Tampere University, Tampere, Finland; 21 Department of Nutrition, Harvard T.H. Chan School of Public Health, Boston, MA, United States; 22 Epicentre, Paris, France; 23 Department of Food Technology, Safety and Health, Faculty of Bioscience Engineering, Ghent University, Ghent, Belgium; 24 School of Public Health and Family Medicine, University of Malawi College of Medicine, Blantyre, Malawi; 25 Department of Women & Children’s Health, King’s College London, London, United Kingdom; 26 Medical Research Council Unit the Gambia at the London School of Hygiene and Tropical Medicine, Fajara, The Gambia; 27 Department of Global Health, Milken Institute School of Public Health, The George Washington University, Washington, DC, United States; 28 Department of Medicine, Namur University, Namur, Belgium; 29 Center for Health Research, School of Public Health, University of Indonesia, Depok, Indonesia; 30 Department of Global and Community Health, College of Public Health, George Mason University, Fairfax, VA, United States; 31 Regional Office for West and Central Africa, UNICEF, Dakar, Senegal

**Keywords:** individual participant data meta-analysis, multiple micronutrient supplements, pregnancy, antenatal care

## Abstract

Multiple micronutrient supplements (MMS) in pregnancy reduces risk of infant low birthweight (LBW) and improves other maternal and infant outcomes compared with iron and folic acid (IFA) supplements alone. However, the impact of timing of initiation and adherence on the MMS effectiveness in real-world programs remains unclear. To address this, we conducted a 2-stage individual participant data meta-analysis that included 15 randomized trials (61,204 pregnant women) and assessed whether the relative effect of MMS differed by the following: adherence alone; adherence in combination with gestational age at initiation; and the total number of tablets taken. We also evaluated the observational association of these factors with outcomes among participants who received MMS. Compared with IFA supplements, the relative effect of MMS on the primary outcome of continuous birthweight was greater with higher adherence (*P*-interaction < 0.05). Among women who took ≥90% of supplements, MMS increased birthweight by 56 g (95% CI: 45, 67 g), whereas among women who took <60% of supplements, there was no difference in birthweight between MMS and IFA supplements [mean difference (MD): 9 g; 95% CI: −17, 35 g). Higher adherence was also associated with greater effect of MMS on LBW and birthweight-for-gestational age centile and women who took more supplements experienced a greater relative impact of MMS on birthweight-for-gestational age centile and small-for-gestational age births (SGA) as compared with IFA supplements. Observational analyses among participants who received MMS showed that ≥90% adherence was associated with increased birthweight (MD: 44 g; 95% CI: 31, 56 g) and lower risk of LBW [relative risk (RR): 0.93 g; 95% CI: 0.88, 0.98 g] and small-for-gestational age (RR: 0.95; 95% CI: 0.93, 0.98), whereas <75% adherence was associated with greater risk of stillbirth (RR: 1.43; 95% CI: 1.12, 1.83) and maternal anemia (RR: 1.26; 95% CI: 1.11, 1.43) than 75%–90% adherence. Programs should invest in strategies that promote early initiation and high adherence to MMS.

This trial was registered at PROSPERO as CRD42022319207.

**Statement of Significance**

Multiple micronutrient supplementation in pregnancy reduces risk of low birthweight and improves other maternal and infant outcomes; however, the role of timing of initiation and supplement adherence in the effectiveness of multiple micronutrient supplements (MMS) remains unclear. As the first individual participant data meta-analysis that examine the timing of initiation and adherence of MMS, our study finds that higher MMS adherence and a greater number of tablets taken were generally associated with more positive birth outcomes.

## Introduction

Antenatal multiple micronutrient supplements (MMS) contain several vitamins and minerals in addition to iron and folic acid (IFA) supplements and were designed to reduce the gap between the increased nutritional requirements of pregnant women and the low dietary intake of these nutrients in low- and middle-income countries (LMICs) [1,2]. MMS are recommended by the WHO in the context of rigorous research, including implementation research [3], and in humanitarian contexts [4]. Some countries and programs are now implementing MMS in public health programs. The Improving Maternal Nutrition Acceleration Plan was launched by UNICEF and partners to prevent anemia and malnutrition in pregnant women, with the goal of reaching 16 million women in 16 countries with a package of essential nutrition services that includes MMS by the end of 2025 [5].

Randomized trial evidence has shown that MMS increase infant birth weight and reduce risk of low birthweight (LBW) and other adverse outcomes compared with FA supplementation alone [[6], [7], [8]]. A previous individual participant data (IPD) meta-analysis found that both supplement adherence and the gestational age at supplementation initiation may modify the effect of MMS on several outcomes [6]. For example, MMS started at <20 wk of gestation had a greater beneficial effect on preterm birth compared with initiation at ≥ 20 wk, whereas ≥95% adherence to supplementation was associated with a greater relative effect of MMS on infant mortality. One limitation of these previous analyses is that only very high adherence (≥95%) was evaluated as a modifier, and it is known that adherence is more variable in real-world programs. The previous IPD meta-analysis also did not examine the total number of tablets taken in pregnancy, which is a function of both gestational age at the start of supplementation and adherence.

The overall goal of this IPD meta-analysis was to evaluate the influence of adherence alone and in combination with gestational age at initiation, as well as the total number of tablets taken during pregnancy on the effects of MMS. We addressed the contribution of these factors in 2 different ways: first as potential factors that modify the relative effect of MMS compared with that of IFA and second to evaluate the observational association of these factors with outcomes among MMS users. The first analysis used the randomized design to compare the relative effect of MMS to IFA by adherence, gestational age, and total number of tablets taken. The second observational analysis among MMS users allowed us to determine the absolute contribution of adherence, gestational age at initiation, and the total number of MMS tablets taken on outcomes; this observational analysis is needed because it is not feasible and likely unethical to conduct trials that randomize women to different MMS adherence patterns or timing of initiation to supplementation. We hypothesized that higher adherence, earlier initiation, and the resulting greater number of tablets taken would be associated with greater benefits on birthweight and secondary maternal and infant outcomes.

## Methods

The protocol for this IPD meta-analysis was registered with PROSPERO (CRD42022319207) [9]. We included trials identified in 2 previous meta-analyses with data published up to 28 February, 2018 [6,7] and conducted an updated literature search for trials published until 2 July, 2024, on MEDLINE (Ovid), Embase (Ovid), and Cochrane Central Register of Controlled Trials (CENTRAL), using the same search strategy used in the 2019 Cochrane review [7] (Supplemental Appendix 1). There were no language restrictions. We also searched clinicaltrials.gov and the WHO International Clinical Trials Registry Platform for unpublished, planned, and ongoing MMS trials. The new study records were screened by 2 independent reviewers (FG, ZR), and a third reviewer (MWB) resolved any conflicts. All authors of eligible studies were invited to participate and contribute data. Data use agreements were set up with participating authors. They prepared the data sets according to a harmonized codebook of variables prespecified in the study protocol. Received data sets were secured and checked for data consistency and completeness; data harmonization efforts were coordinated directly with the study teams. Studies for which IPD were not available did not have published aggregate data that could be used for these analyses.

### Eligibility criteria

We included studies that compared the effect of daily MMS with that of iron (with or without folic acid) during pregnancy. Iron (with or without folic acid) was used as the control group because it was considered the standard of care with proven effectiveness, and a placebo group would have been unethical. MMS was defined as supplements that included ≥3 micronutrients (irrespective of dose) in addition to IFA. Trials were included if the comparison group received iron (with or without folic acid), either through the study or as the standard of care. Only randomized trials conducted in LMICs were included, and data from trials or trial arms (e.g., excluding trial arms where other interventions were included), which provided MMS via parenteral nutrition or fortified food products, were excluded.

### Risk of bias assessment

The risk of bias assessment evaluated the bias arising from the randomization process, deviations from intended interventions, missing outcome data, measurement of the outcomes and selection of the reported result, based on 1 of the 3 ratings: low risk of bias, unclear risk of bias, or high bias [10]. Each trial was rated for these criteria, using the RevMan software.

### Analytic objectives

We conducted 2 sets of analyses to better understand whether adherence and timing of supplementation initiation affected the impact of MMS on birthweight and secondary maternal and infant outcomes. First, we investigated whether the relative effect [mean difference (MD) for continuous outcomes or risk ratio (RR) for binomial outcomes] of MMS compared with that of IFA was modified by adherence alone or in combination with the gestational age at initiation of supplementation and the total number of tablets. These analyses made use of the original randomized trial study design. For example, first, participants were divided into adherence subgroups based on their MMS or IFA adherence. Then, outcomes were compared for each subgroup (e.g., women with ≥90% adherence to MMS compared with women who with ≥90% adherence to IFA). The effect sizes between the adherence subgroups were then compared. The analyses were similar for adherence and timing of initiation subgroups and the total number of tablets subgroups.

Second, we conducted an observational analysis among pregnancies randomized to MMS to assess the association of these factors (adherence, adherence and gestational age at initiation, and total number of tablets) as exposures on outcomes. The results of this analysis estimate the effects as if one was to randomize women to different adherence patterns (e.g., a trial randomizing women to MMS with 90% adherence, MMS with 75% adherence, and MMS with 50% adherence). Importantly, in the first analysis, the intervention is MMS (containing IFA) compared with IFA, and therefore, the effect of IFA is equal between the arms; however, when looking among MMS users in the second analysis, women who had higher adherence and took a greater number of MMS tablets consumed a greater amount of micronutrients, including IFA, compared with women with lower MMS adherence and took a lesser number of tablets. Therefore, the contribution of higher adherence and greater IFA is captured in the second analysis.

### Outcomes

The outcomes analyzed in this study were selected a priori by the study team based on biologic plausibility and the availability of data in MMS trials based on previous systematic reviews and an IPD meta-analysis [[6], [7], [8]]. For both study objectives, the primary outcome was continuous birthweight (grams). Birthweight was selected because it was a common outcome reported by nearly all trials, and an analysis of a continuous outcome is expected to have greater statistical power than a binomial outcome (e.g., LBW). The secondary outcomes of interest were LBW (<2500 g among livebirths), birthweight centile (continuous as defined by intergrowth standards among livebirths) [11], preterm birth (<37 wk of gestation among livebirths), small-for-gestational age (SGA: <10th percentile birthweight-for-gestational age by sex as defined by intergrowth standards) [11], large-for-gestational age (LGA; >90th percentile as defined by intergrowth standards) [11], gestation duration (in weeks continuously among livebirths), fetal death (intrauterine death of a fetus at any time during pregnancy), stillbirth (fetal death >28 wk of gestation), neonatal death (death <28 days of age among livebirths), and infant mortality (death <365 d of age among livebirths). We also assessed third-trimester maternal hemoglobin concentration (in grams per deciliter continuous), maternal anemia (hemoglobin <11 g/dL), and IDA (ferritin concentration < 12.0 μg/L and hemoglobin <11 g/Dl). Third-trimester assessment of maternal hemoglobin and iron status was selected because it was the most common timing of postbaseline assessment of these biomarkers across trials.

### Analytic methods for adherence as a modifier of the relative effect of MMS compared with that of IFA

For the first set of analyses that examined the relative effect of MMS compared with that of IFA, there were 3 effect modifiers (subgroups) of interest: *1*) percent adherence categories, *2*) the combination of adherence categories and gestational age at supplementation initiation, and *3*) the total number of daily supplements taken. In order to standardize calculations, we used the day method to define supplement adherence, where adherence was calculated as the percentage of days MMS or IFA tablets were taken from randomization to delivery. For trials that did not have the data available in a way that allowed for the day method calculation, we used the tablet method; we divided the number of MMS or IFA tablets taken by the number of tablets expected to be taken based on 1 tablet per day from randomization to delivery. We categorized the adherence variable (for both MMS and IFA) into 4 categories for the analysis: <60% adherence, 60%–74% adherence, 75%–89% adherence, and ≥90% adherence. These cutoffs were chosen based on the distribution of the trial data. We also constructed a variable combining both adherence categories and gestational age at the start of supplementation. We further stratified these adherence categories by <20 and ≥20 wk of gestational age to create an 8-level variable to examine treatment effects within each level. The gestational age cutoff of 20 wk was selected based on a previous IPD meta-analysis [6] and because few trials had enrolled participants across all trimesters of pregnancy. The total number of tablets taken, which can also be interpreted as the total number of days supplements were taken between randomization and delivery, was categorized into 4 groups: <90, 90–120, 120–180, and ≥180 tablets.

In the first step of the IPD meta-analysis for this objective, each trial data set was analyzed to produce nonparametric MD estimates or relative risk (RR) and 95% Cis for the effect of MMS compared with that of IFA on the outcomes of interest, stratified (separate models) by the 3 subgroups of interest. Cluster randomized trials accounted for clustering using generalized estimating equations and complete case analyses were performed. In the second stage of the IPD meta-analyses, we pooled the study-specific estimates using fixed-effects meta-analyses. We conducted random-effects meta-analyses as sensitivity analyses. For binomial outcomes, we excluded trials that did not contribute ≥1 outcome event to each modifier stratum; a continuity correction was used when there were zero events in 1 study arm but there were 1 or more events in the other study arm within a stratum. For continuous outcomes, if there were no pregnant women within a stratum, the trial was excluded from the subgroup analysis. There were 39 analyses based on 13 primary and secondary outcomes and the 3 modifiers of interest. We did not account for multiple testing due to the exploratory nature of the study. As a sensitivity analysis, we also examined the relative effect of MMS compared with that of IFA on birthweight and maternal hemoglobin separately for the subgroup of trials that used the same dose of iron in MMS and IFA and for the subgroup of trials that used a lower dose of iron in the MMS group than that in IFA.

### Analytic methods for the association of adherence with outcomes among MMS users

We then examined the association of percent adherence categories, the combination of adherence and gestational age at initiation, and the total number of tablets and outcomes among MMS users. The definitions of the 3 exposures of interest were the same as for the effect modifier analyses. The analyses were restricted to pregnant women that were randomized to receive MMS. In the first step of the IPD meta-analysis, each trial data set was analyzed to assess the association of adherence categories (reference group: 75% to <90%), the combination of adherence and gestational age at the start (reference group: 75% to <90% adherence and <20 wk of gestation at initiation), and the total number of daily supplements (reference group: 90 to <120 tablets) with the outcomes of interest. The reference groups for exposure variables were selected as the middle category to allow for better identification of a dose–response pattern and potential nonlinearity. Due to the observational nature of the analysis, the following potential confounders were adjusted for in each analysis: maternal age (<20, 20–24, 25–34, and 35+ y), maternal education (continuous), parity (firstborn, 2–4 born, 5+), baseline maternal BMI (in kg/m^2^; <18.5, 18–24.9, and 25+), maternal height (continuous), baseline maternal anemia (<10, 10–10.9, and ≥11 g/Dl), and study-specific wealth quintile (categorical). We also adjusted for infant sex and twin status for analyses conducted among live births (e.g., birthweight outcome) as these are likely prognostic factors and therefore inclusion increased the precision of the estimates. We did not adjust for sex in analyses of fetal death due to a large degree of missing sex data for fetal deaths. Each trial estimate was adjusted for as many covariates as possible. If data were completely missing for a covariate or was uniform for the study population (e.g., all HIV-negative participants), these covariates were not included in the trial-specific model. In the second step of the IPD meta-analyses, we pooled the study-specific estimates using fixed-effects models. As a sensitivity analysis, we conducted random-effects meta-analyses. We quantified statistical heterogeneity using the *I*^2^ test statistic and considered an *I*^2^ >75% to represent considerable heterogeneity. For binomial and continuous outcomes, we excluded trials that did not have ≥1 event or datapoint in each exposure category of interest. Owing to the multivariable analysis, it was not possible to use continuity corrections. All statistical analyses were performed in R.

## Results

**FIGURE 1:**
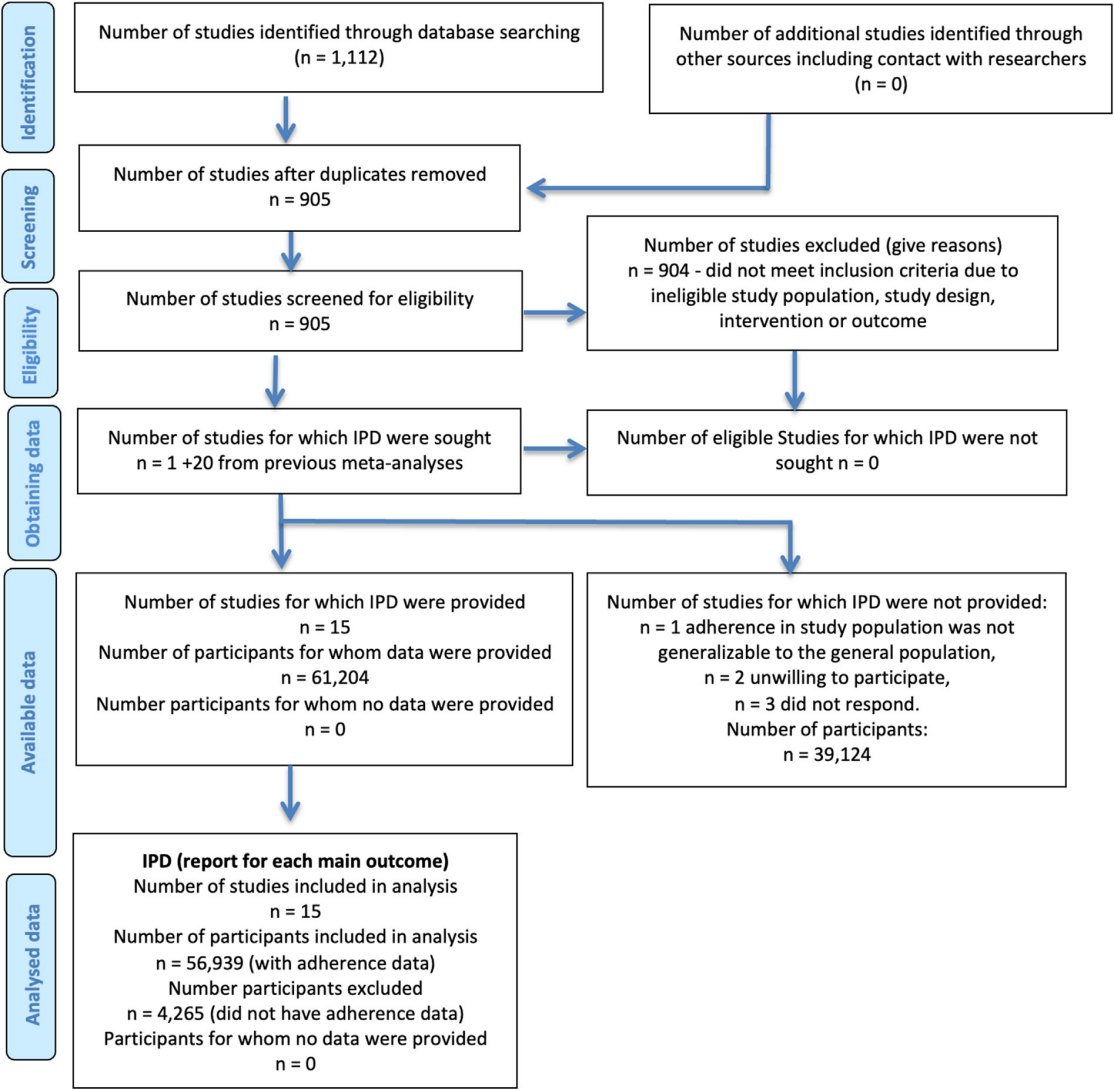
Flow diagram of identified and included studies. IPD, individual participant data.

In the literature search, we identified 1112 citations, of which 905 remained after removing duplicates; only 1 new trial met the inclusion criteria [12]. Therefore, 21 trials met our inclusion criteria [[12], [13], [14], [15], [16], [17], [18], [19], [20], [21], [22], [23], [24], [25], [26], [27], [28], [29], [30], [31], [32]]; 15 trial teams contributed data [[12], [13], [14],^16^,^18^,[20], [21], [22],[24], [25], [26], [27],^29^,^31^,^32^], and 6 trial teams (corresponding to 39,124 participants) were unable or unwilling to participate [15,17,19,23,28,30]. Figure 1 shows a flow diagram of the literature search and eligible studies. Table 1 [[12], [13], [14],^16^,^18^,^21^,[24], [25], [26], [27],^29^,^32^] provides a summary of the included trial populations, adherence assessment methods (pill count, direct observation, electronic counting device, or self-reported), and descriptive characteristics of adherence and timing of supplementation initiation within each trial. Most trials used pill counts to assess adherence, but the frequency of assessments ranged from once a week to a single pill count at delivery. Two trials included direct observation [18,25], whereas 1 trial used electronic monitoring [20]. Overall, the trials included 61,204 pregnancies; adherence data were available for 93% of participants, resulting in an analytic sample of 56,939 pregnancies. Within each trial, the median and IQR for adherence percentage were similar between the MMS and IFA groups (Table 1) [[12], [13], [14],^16^,^18^,^21^,[24], [25], [26], [27],^29^,^32^]. However, adherence differed by trial with the proportion of women with >90% adherence ranging from 15% [14,32] to 83% [22] (Supplemental Table 1). Gestational age at supplementation initiation also varied by trial; the proportion of pregnancies that initiated supplementation <20 wk of gestation ranged from 0% [32] to 100% [22] (Table 1) [[12], [13], [14],^16^,^18^,^21^,[24], [25], [26], [27],^29^,^32^]. Four trials used the same dose of iron in the MMS and IFA arms, 9 trials used a lower dose of iron in the MMS arm, 1 trial used 2 IFA arms (one with the same dose of iron as MMS and other with a higher dose of iron), and 1 trial did not specify the iron dose (Supplemental Table 2). As stated in Methods section, the only issue encountered when checking the IPD was that some trials did not have the data available for the day method calculation of adherence. For these cases, we divided the number of MMS or IFA tablets taken by the number of tablets expected to be taken based on 1 per day from randomization to delivery. Risk of bias was assessed as low for all trials (Supplemental Figure 1); the domain with the highest risk of bias was incomplete outcome data (attrition bias) (Supplemental Figure 2).

**TABLE 1:** Study design characteristics of randomized trials included in the individual participant data meta-analysis.

Study	Country	Study design	No. participants	Median (Q1, Q3) adherence (%)	No. (%) of women started supplementation <20 wk	Study population	Adherence assessment: method and schedule
Adu-Afarwuah et al. [13], 2015	Ghana	RCT	880	MMS: 81 (67, 88); IFA: 82 (69, 89)	MMS: 393 (90); IFA: 376 (85)	Pregnant women <20 wk of gestation (excluding lipid-based nutrient supplement group)	Mainly by journal verified/coupled with count of unused capsules, assessed every 14 d
Ashorn et al. [24], 2015	Malawi	RCT	929	MMS: 89 (75, 98); IFA: 88 (76, 100)	MMS: 448 (96); IFA: 449 (97)	Pregnant women <20 wk of gestation (excluding lipid-based nutrient supplement group)	Tablet count, assessed every 14 d
Bhutta et al. [26], 2009	Pakistan	cRCT	2378	MMS: 88 (74, 94); IFA: 88 (77, 94)	MMS: 1138 (99); IFA: 1215 (99)	Pregnant women <16 wk of gestation	Tablet count, assessed every 14 d
Bliznashka et al. [12], 2022	Niger	cRCT	1992	MMS: 94 (92, 96); IFA: 94 (92, 96)	MMS: 828 (86); IFA: 898 (87)	Pregnant women <30 wk of gestation (excluding lipid-based nutrient supplement group)	Tablet count, assessed every 14 d
Christian et al. [27], 2003	Nepal	cRCT	1710	MMS: 89 (74, 97); IFA: 90 (69, 97)	MMS: 843 (92); IFA: 743 (93)	Pregnant women (only IFA and MMS arms included)	Tablet count, assessed twice each week
Fawzi et al. [29], 2007	Tanzania	RCT	8277	MMS: 96 (83, 100); IFA: 96 (82, 100)	MMS: 1383 (33); IFA: 1383 (33)	Pregnant women of 12–27 wk of gestation	Tablet count, assessed every 28 d
Friis et al. [32], 2004	Zimbabwe	RCT	1669	MMS: 88 (63, 100); IFA: 87 (66, 100)	MMS: 0 (0); IFA: 0 (0)	Pregnant women 22–36 wk of gestation including 725 HIV-infected women	Tablet count, assessed at delivery
Kaestel et al. [14], 2005	Guinea-Bissau	RCT	2098	MMS: 68 (47, 84); IFA: 70 (53, 88)	MMS: 384 (28); IFA: 205 (29)	Pregnant women <37 wk of gestation (both MMS arms included)	Tablet count, assessed every 14 d
Moore [31], 2012	The Gambia	RCT	438	MMS: 95 (91, 98); IFA: 96 (93, 98)	MMS: 209 (95); IFA: 211 (96)	Pregnant women <20 wk of gestation (excluding protein and energy supplements groups)	Tablet count, assessed every 7 d
Osrin et al. [16], 2005	Nepal	RCT	1200	MMS: 98 (92, 100); IFA: 98 (93, 100)	MMS: 580 (97); IFA: 577 (96)	Singleton pregnant women between 12 and 20 wk of gestation	Tablet count, assessed every month
Roberfroid et al. [18], 2008	Burkina Faso	RCT	1371	MMS: 90 (70, 100); IFA: 90 (70, 100)	MMS: 391 (57); IFA: 387 (56)	Pregnant women	Direct observation by research team, assessed daily
Sunawang et al. [25], 2009	Indonesia	cRCT	1694	MMS: 82 (58, 95); IFA: 81 (60, 94)	MMS: 854 (99); IFA: 838 (99)	Pregnant women	Direct observation or tablet count, assessed every day in women who had daily visits and every month in women who had monthly visits
Persson [20], 2012	Bangladesh	RCT	4436	MMS: 70 (44, 91); IFA: 75 (47, 94)	MMS: 1453 (98); IFA: 2916 (99)	Pregnant women between 6 and 8 wk of gestation	Electronic counting devices with microprocessors embedded in the capsules; assessed daily until 30th week of gestation
West et al. [21], 2014	Bangladesh	cRCT	29,600	MMS: 97 (87, 100); IFA: 97 (88, 100)	MMS: 13,797 (93); IFA: 13,583 (92)	Pregnant women (79% <13 wk of gestation)	Tablet count based on number of tablets refilled, assessed every 7 d
Zagre [22], 2007	Niger	cRCT	2902	MMS: 98 (92, 100); IFA: 100 (96, 100)	MMS: 1521 (100); IFA: 1381 (100)	Pregnant women <28 wk of gestation	Tablet count, assessed every month

Abbreviations: cRCT, cluster randomized controlled trial; IFA, iron and folic acid supplements; MMS, prenatal micronutrient supplements.

### Adherence as a modifier of the relative effect of MMS compared with that of IFA

First, we examined the relative effect of MMS compared with that of IFA, stratified by adherence subgroups. The magnitude of the beneficial effect of MMS compared with that of IFA on birthweight (*P*-heterogeneity = 0.006), LBW (*P*-heterogeneity = 0.02), birthweight-for-gestational age centile (*P*-heterogeneity = 0.003), and SGA (*P*-heterogeneity = 0.03) was greater with higher adherence (Table 2; Supplemental Figures 3.1–3.14). With ≥90% adherence, MMS increased birthweight by 56 g (95% CI: 45, 67 g) compared with IFA, increased birthweight by 36 g (95% CI: 17, 56 g) with 75%–90% adherence, and there was little to no effect of MMS compared with that of IFA with <60% adherence (MD: 9 g; 95% CI: −17, 35 g). With ≥90% adherence, MMS decreased LBW compared with IFA (RR: 0.88; 95% CI: 0.84, 0.91); benefits were also observed with adherence of 75%–90% (RR: 0.91; 95% CI: 0.85, 0.97).

**TABLE 2:** Pooled effect size comparing MMS with IFA, stratified by percent adherence groups.

Outcome	No. of participants	No. of studies	MMS vs IFA: <60% adherence	MMS vs IFA: 60%–<75% adherence	MMS vs IFA: 75% to <90% adherence	MMS vs IFA: ≥90% adherence	*P* for between subgroup heterogeneity
			ES (95% CI)	ES (95% CI)	ES (95% CI)	ES (95% CI)	
Birthweight (g) (MD)	45,639	14	8 (−17, 35)	58 (28, 88)	36 (17, 56)	56 (45, 67)	0.006

Low birthweight (RR)	43,969	13	1.02 (0.94, 1.12)	0.92 (0.83, 1.03)	0.91 (0.85, 0.97)	0.88 (0.84, 0.91)	0.02
Birthweight percentile (MD)	42,821	13	−2.31 (−4.05, −0.56)	2.33 (0.4, 4.25)	0.85 (−0.44, 2.13)	0.77 (−0.02, 1.55)	0.003
Preterm birth (RR)	50,548	13	0.92 (0.83, 1.02)	0.98 (0.88, 1.1)	0.95 (0.88, 1.03)	0.89 (0.84, 0.94)	0.29
Gestational age (MD)	50,548	13	0.16 (0.00, 0.33)	0.20 (0.04, 0.35)	0.13 (0.02, 0.25)	0.29 (0.22, 0.37)	0.10
SGA <10th percentile (RR)	42,821	13	1.06 (0.99, 1.14)	1.02 (0.94, 1.11)	0.96 (0.91, 1.01)	0.96 (0.94, 0.99)	0.03
LGA >90th percentile (RR)	41,730	12	0.84 (0.71, 1.01)	1.11 (0.94, 1.31)	1.00 (0.88, 1.14)	1.01 (0.92, 1.10)	0.17

Stillbirth (RR)	48,577	9	0.97 (0.74, 1.27)	0.74 (0.53, 1.05)	0.79 (0.64, 0.98)	0.91 (0.82, 1.02)	0.42
Fetal death (RR)	56,215	12	0.91 (0.81, 1.03)	0.93 (0.77, 1.13)	0.92 (0.8, 1.06)	0.98 (0.92, 1.04)	0.68
Neonatal death (RR)	20,419	9	1.17 (0.76, 1.79)	0.97 (0.61, 1.54)	1.15 (0.79, 1.67)	1.02 (0.82, 1.27)	0.90
Infant death (RR)	9407	6	1.14 (0.68, 1.91)	0.97 (0.59, 1.6)	1.33 (0.85, 2.07)	1.19 (0.91, 1.56)	0.82

Third-trimester hemoglobin (g/dL) (MD)	9418	8	−0.03 (−0.15, 0.09)	−0.1 (−0.2, 0.01)	−0.15 (−0.24, −0.06)	−0.1 (−0.17, −0.03)	0.42
Third-trimester anemia (<11g/dL) (RR)	9418	8	1.07 (0.96, 1.19)	1.14 (1.05, 1.24)	1.09 (0.99, 1.2)	1.06 (0.99, 1.13)	0.53
Third-trimester IDA (RR)	2204	3	1.05 (0.70, 1.59)	1.45 (0.97, 2.17)	0.72 (0.5, 1.04)	1.38 (1.16, 1.63)	0.01

This table presents the results of a fixed-effects meta-analysis for each outcome across 4 subgroups by adherence level. Each estimate (RR or MD) is the pooled effect size comparing MMS with IFA.

Abbreviations: ES, effect size; IDA, iron-deficiency anemia; LGA, large-for-gestational age; MD, mean difference; MMS, prenatal micronutrient supplements; IFA, iron and folic acid supplements; RR, relative risk; SGA, small-for-gestational age.

There was no difference in the magnitude of the effect of MMS relative to IFA on maternal anemia, fetal death, stillbirth, LGA, and neonatal and infant death by adherence groups. There was evidence of effect modification by percent adherence on third-trimester iron-deficiency anemia (IDA), although it did not follow an expected dose–response pattern, with a benefit of MMS only shown in the middle 75%–90% adherence group (*P*-heterogeneity = 0.01). A sensitivity analysis suggested that this effect was driven by the trial by Sunawang et al. [25], which was the only trial of the 3 that assessed IDA that used a lower dose of iron in MMS that that in IFA (Supplemental Table 3; Supplemental Figures 3.15–3.22). Additional sensitivity analyses by iron dose in MMS and IFA were consistent with the primary overall results (Supplemental Table 3). We then analyzed effect modification by the combination of adherence and gestational age at initiation, and there were no subgroup differences for nearly all outcomes except for the birthweight percentile (*P*-heterogeneity = 0.002), where MMS tended to have a greater effect on birthweight when initiated prior to 20 wk and with higher adherence (Supplemental Figures 3.23–3.36; Supplemental Table 4; sensitivity analysis shown in Supplemental Figures 3.37–3.41 and Supplemental Table 5).

We also examined the relative effect of MMS stratified by the total number of tablets taken. There was no difference in the magnitude of the effect of MMS compared with that of IFA stratified by tablet count subgroups for birthweight and most secondary outcomes (Table 3). However, the magnitude of the effect of MMS compared with that of IFA on birthweight-for-gestational age percentile (*P*-heterogeneity = 0.04), SGA (*P*-heterogeneity <0.001), and LGA (*P*-heterogeneity = 0.01) differed between tablet subgroups with a tendency of a larger birth size with a greater number of tablets (Table 3; Supplemental Figures 3.42–3.55). There were no apparent differences between trials using a lower and those using the same dose of iron in MMS (Supplemental Figures 3.56–3.63; Supplemental Table 6).

**TABLE 3:** Pooled effect size comparing MMS with IFA, stratified by tablet count groups.

Outcome	No. of participants	No. of studies	MMS vs IFA: <90 total tablets	MMS vs IFA: 90–120 total tablets	MMS vs IFA: 120–180 total tablets	MMS vs IFA: ≥180 total tablets	*P* for between subgroup heterogeneity
			ES (95% CI)	ES (95% CI)	ES (95% CI)	ES (95% CI)	
Birthweight (g) (MD)	45,175	13	34 (9, 59)	54 (30, 78)	53 (37, 68)	44 (31, 56)	0.55

Low birthweight (RR)	44,092	12	0.93 (0.86, 1.01)	0.90 (0.81, 0.99)	0.92 (0.85, 1.00)	0.88 (0.84, 0.92)	0.58
Birthweight percentile (MD)	42,370	12	−1.31 (−3.00, 0.38)	2.11 (0.32, 3.9)	0.89 (−0.19, 1.97)	1.08 (0.34, 1.81)	0.04
Preterm birth (RR)	39,701	8	0.88 (0.81, 0.95)	0.97 (0.91, 1.05)	0.88 (0.83, 0.93)	0.89 (0.81, 0.97)	0.13
Gestational age (MD)	50,002	12	0.30 (0.13, 0.47)	0.13 (−0.01, 0.28)	0.24 (0.14, 0.33)	0.18 (0.11, 0.25)	0.23
SGA <10th percentile (RR)	39,654	11	1.10 (1.02, 1.18)	0.91 (0.83, 0.99)	0.97 (0.9, 1.04)	0.95 (0.93, 0.98)	0.001
LGA >90th percentile (RR)	39,758	9	0.90 (0.81, 1.00)	0.94 (0.84, 1.04)	1.01 (0.91, 1.12)	1.29 (1.06, 1.57)	0.01

Fetal death (RR)	52,910	9	0.98 (0.93, 1.03)	0.95 (0.78, 1.15)	0.92 (0.79, 1.07)	0.98 (0.84, 1.15)	0.88
Stillbirth (RR)	46,885	7	0.80 (0.66, 0.97)	0.92 (0.73, 1.17)	0.91 (0.78, 1.07)	0.96 (0.82, 1.13)	0.57
Neonatal death (RR)	17,519	7	0.97 (0.69, 1.37)	0.84 (0.57, 1.23)	1.17 (0.88, 1.55)	1.46 (0.92, 2.30)	0.28
Infant death (RR)	9681	6	1.04 (0.71, 1.53)	1.18 (0.75, 1.87)	1.35 (1.00, 1.84)	1.14 (0.78, 1.66)	0.76

Third-trimester hemoglobin (MD)	10,777	9	−0.05 (−0.18, 0.08)	−0.10 (−0.21, 0.02)	−0.13 (−0.24, −0.02)	−0.03 (−0.12, 0.06)	0.49
Third-trimester anemia (<11 g/dL) (RR)	10,777	9	1.05 (0.94, 1.18)	1.08 (0.95, 1.23)	1.07 (0.98, 1.17)	1.03 (0.93, 1.13)	0.91
Third-trimester IDA (RR)	2204	3	1.11 (0.72, 1.69)	1.62 (1.16, 2.27)	1.07 (0.87, 1.32)	1.17 (0.89, 1.54)	0.22

This table presents the results of a fixed-effects meta-analysis for each outcome across 4 subgroups by adherence level. Each estimate (RR or MD) is the pooled effect size comparing MMS with IFA.

Abbreviations: ES, effect size; IDA, iron-deficiency anemia; LGA, large-for-gestational age; MD, mean difference; MMS, prenatal micronutrient supplements; IFA, iron and folic acid supplements; RR, relative risk; SGA, small-for-gestational age.

### Association of adherence with outcomes among MMS users

We then assessed the observational association of adherence with maternal and infant outcomes among participants randomized to MMS (Table 4; Supplemental Figures 4.1–4.14). Infants born to pregnant women who had ≥90% MMS adherence had greater birthweight (MD: 18 g; 95% CI: 3, 33 g), lower risk of LBW (RR: 0.93; 95% CI: 0.88, 0.98), and lower risk of SGA (0.96; 95% CI: 0.92, 1.00) compared with infants born to pregnant women who had ≥75% to <90% adherence to MMS. In addition, <60% adherence and ≥60% to <75% adherence groups had a higher risk of stillbirth and third-trimester anemia compared with the ≥75 to <90% adherence reference group (*P* < 0.05). The association of the combination of adherence and timing of supplementation initiation with outcomes is presented in Supplemental Figures 4.15–4.28 and Supplemental Table 7. Higher adherence groups and MMS initiation before 20 wk of gestation were generally associated with greater increases in birthweight. When compared with the reference group of initiation >20 wk of gestation with 75%−90% adherence, infants with mothers in the lowest adherence group (<60%) and who initiated MMS after 20 wk had 80 g (95% CI: −145, −15 g) lower birthweight, whereas infants whose mothers were in the highest adherence group (>90%) and initiated MMS <20 wk had 23 g (95% CI: 4, 42 g) greater birthweight. Last, as expected, due to the bias related to the duration of gestation, taking >180 MMS tablets in pregnancy was strongly associated with a lower risk of preterm birth, greater birthweight, and lower risk of stillbirth than taking 90–120 MMS tablets in pregnancy (Supplemental Figures 4.29–4.42; Table 5).

**TABLE 4:** Pooled association of adherence with adverse outcomes among MMS users (observational data).

	No. of participants (all subgroups)	No. of studies	MMS: <60% adherence	MMS: 60% to <75% adherence	MMS: 75%–<90% adherence	MMS: ≥90% adherence
			ES (95% CI)	ES (95% CI)	Reference	ES (95% CI)
Birthweight (g) (MD)	22,821	15	−19 (−41, 3.8)	7.5 (−17, 32)	0.00	18 (3, 33)

Low birthweight (RR)	21,602	12	1.00 (0.92, 1.09)	0.99 (0.91, 1.08)	1.00	0.93 (0.88, 0.98)
Birthweight percentile (MD)	21,429	14	−0.78 (−2.29, 0.73)	1.44 (−0.20, 3.08)	0.00	0.51 (−0.50, 1.51)
Preterm birth (RR)	25,231	13	1.07 (0.97, 1.18)	1.08 (0.98, 1.18)	1.00	1.00 (0.94, 1.07)
Gestational age (MD)	25,440	14	−0.11 (−0.24, 0.02)	−0.05 (−0.20, 0.10)	0.00	0.00 (−0.10, 0.10)
SGA <10th percentile (RR)	21,252	13	1.02 (0.96, 1.09)	1.02 (0.95, 1.10)	1.00	0.96 (0.92, 1.00)
LGA >90th percentile (RR)	19,943	10	0.90 (0.80, 1.02)	1.02 (0.90, 1.17)	1.00	0.96 (0.88, 1.06)

Fetal death (RR)	27,894	10	2.71 (2.37, 3.10)	1.69 (1.45, 1.96)	1.00	1.30 (1.17, 1.45)
Stillbirth (RR)	24,267	8	1.43 (1.12, 1.83)	1.34 (1.05, 1.70)	1.00	1.02 (0.86, 1.20)
Neonatal death (RR)	8407	6	1.45 (0.88, 2.40)	2.00 (1.24, 3.23)	1.00	1.22 (0.88, 1.69)
Infant death (RR)	4237	6	1.31 (0.79, 2.18)	1.77 (1.10, 2.86)	1.00	1.13 (0.81, 1.60)

Third-trimester hemoglobin (MD)	5120	10	−0.20 (−0.32, −0.08)	−0.01 (−0.13, 0.11)	0.00	0.02 (−0.06, 0.11)
Third-trimester anemia (<11 g/dL) (RR)	4471	9	1.26 (1.11, 1.43)	1.16 (1.03, 1.31)	1.00	0.95 (0.86, 1.04)
Third-trimester IDA (RR)	1100	3	1.91 (1.30, 2.82)	1.97 (1.47, 2.64)	1.00	1.76 (1.28, 2.42)

Abbreviations: ES, effect size; IDA, iron-deficiency anemia; LGA, large-for-gestational age; MD, mean difference; MMS, prenatal micronutrient supplements; RR, relative risk; SGA, small-for-gestational age.

**TABLE 5:** Pooled association of total tablet count with adverse outcomes among MMS users (observational data).

	No. participants (all subgroups)	No. studies	MMS: <90 total tablets	MMS: 90–120 total tablets	MMS: 120–180 total tablets	MMS: ≥180 total tablets
			ES (95% CI)	Reference	ES (95% CI)	ES (95% CI)
Birthweight (g) (MD)	22,398	13	−50 (−73, −26)	0.00	51 (32, 70)	121 (94, 148)

Low birthweight (RR)	21,683	12	1.04 (0.94, 1.14)	1.00	0.91 (0.84, 0.99)	0.72 (0.66, 0.78)
Birthweight percentile (MD)	21,012	12	2.49 (0.81, 4.17)	0.00	−4.89 (−6.31, −3.47)	−12.24 (−14.26, −10.23)
Preterm birth (RR)	18,994	7	0.86 (0.79, 0.94)	1.00	0.81 (0.76, 0.87)	0.32 (0.29, 0.35)
Gestational age (MD)	24,951	12	−0.92 (−1.06, −0.78)	0.00	1.12 (0.99, 1.25)	2.42 (2.22, 2.62)
SGA <10th percentile (RR)	19,585	11	1.11 (1.01, 1.22)	1.00	1.14 (1.05, 1.23)	1.30 (1.19, 1.43)
LGA >90th percentile (RR)	18,763	8	1.03 (0.93, 1.13)	1.00	0.64 (0.59, 0.70)	0.29 (0.25, 0.33)

Fetal death (RR)	25,644	7	3.92 (3.38, 4.54)	1.00	0.44 (0.36, 0.53)	0.24 (0.20, 0.29)
Stillbirth (RR)	22,108	5	1.13 (0.90, 1.42)	1.00	0.61 (0.49, 0.76)	0.34 (0.27, 0.42)
Neonatal death (RR)	8307	7	1.66 (1.15, 2.42)	1.00	1.04 (0.73, 1.49)	1.18 (0.72, 1.94)
Infant death (RR)	4375	6	1.25 (0.82, 1.90)	1.00	0.77 (0.51, 1.15)	0.78 (0.49, 1.24)

Third-trimester hemoglobin (MD)	4925	9	−0.10 (−0.21, 0.01)	0.00	0.02 (−0.08, 0.12)	−0.01 (−0.12, 0.11)
Third-trimester anemia (<11 g/dL) (RR)	4925	9	1.10 (1.03, 1.17)	1.00	0.94 (0.84, 1.05)	0.99 (0.89, 1.11)
Third-trimester IDA (RR)	1100	3	1.00 (0.73, 1.37)	1.00	0.68 (0.53, 0.87)	0.87 (0.69, 1.10)

Abbreviations: ES, effect size; IDA, iron-deficiency anemia; LGA, large-for-gestational age; MD, mean difference; MMS, prenatal micronutrient supplements; RR, relative risk; SGA, small-for-gestational age.

## Discussion

In this series of IPD meta-analyses, we sought to better understand how supplementation adherence influences the effect of MMS on birthweight and other maternal and infant outcomes. In terms of the relative effect of MMS compared with that of IFA, there was evidence that the magnitude of the beneficial effect on birthweight, LBW, and birthweight-for-gestational age centile was greater with higher adherence. Similarly, we found MMS had a larger effect on birth size, compared with IFA, among participants who took a greater number of MMS tablets. In terms of the association of adherence with outcomes among MMS users, we also found that higher adherence was associated with greater birthweight and a lower risk of LBW and SGA, whereas lower adherence was associated with an increased risk of stillbirth and maternal anemia. Among MMS users, >90% adherence was most strongly associated with birth size. Taken together, these findings suggest that adherence influences the effect of MMS and that higher MMS adherence and a greater number of tablets taken were generally associated with more positive birth outcomes.

A previous IPD meta-analysis, which included a larger number of participants (but included most trials in the current IPD), found that adherence modified the effect of MMS relative to that of IFA on infant mortality, with a greater magnitude of survival benefits for infants born to mothers with >95% adherence [6]. Nevertheless, there were no other infant outcomes modified at this very high adherence threshold. Our new analysis, which considered a wider distribution of adherence categories, found that the effect of MMS relative to IFA on birthweight was modified by supplement adherence, and higher birthweights were found in categories with >60% adherence. However, adherence did not modify the relative effect of MMS compared with that of IFA on other infant outcomes, including preterm birth and stillbirth. The observational analyses among MMS users also showed that higher adherence was associated with greater birthweight. Micronutrients can affect fetal growth and development from conception and throughout pregnancy with varied biological windows of effect [33]. Greater adherence to supplementation would likely contribute to greater stores of minerals and fat-soluble vitamins and increase the availability of water-soluble vitamins during key periods of biological effects for different outcomes. More simply, higher adherence provides a greater probability that micronutrients are present in adequate amounts at the right time. However, it is important to note that although MMS (in a formulation similar to the United Nations International Multiple Micronutrient Antenatal Preparation) was found to improve maternal micronutrient status in a trial in Bangladesh, deficiencies remained prevalent for multiple vitamins and minerals by the third trimester [34].

Our findings have implications for programs that support antenatal care (ANC) and provide MMS during pregnancy. Combining the evidence from this analysis and the previous IPD meta-analysis, starting MMS <20 wk of gestation and having ≥60% adherence may lead to greater benefits on preterm birth and birthweight but achieving >90% adherence likely confers a greater benefit on birth size, including SGA. Therefore, programs and interventions that support early ANC attendance and high MMS adherence will likely have a greater impact. In addition, our findings have implications for the total number of MMS tablets that should be budgeted or allocated per pregnancy in programs, budgets, and cost-effective analyses. The number of MMS tablets required for a pregnant woman starting ANC in the first trimester at 10 wk of gestation, who achieves 90% adherence and has a full-term 40-week pregnancy, would be 189 tablets.

There are several areas for future work in this area. For example, a commonly used indicator of IFA adherence in demographic and health surveys and other population-level surveys is the self-report of taking ≥90 tablets in pregnancy; however, our analysis suggests that using an indicator of >180 tablets (which was achieved within the randomized trials) would likely capture a greater potential for the intervention to provide benefit. Balanced energy and protein dietary supplementation is recommended for pregnant women in undernourished populations with a prevalence of ≥20% underweight women [3], which can be fortified with vitamins and minerals or used in combination with MMS. Future research may further optimize micronutrient dosages in balanced energy and protein and MMS.

There are several limitations to our analysis. First, adherence was measured by tablet count either self-reported or observed by study staff. Tablet counts may be unreliable for various reasons including participant missed visits, failure to return packages or bottles at the visit, sharing of supplements with other household members, and even intentional manipulation of tablet count data by participants [[35], [36], [37]]. A number of studies have shown that tablet counts are not perfectly aligned with other measures of adherence including electronic bottle cap monitoring and blood-based biomarkers; however, in the context of large clinical trials, tablet counts are often most feasible [38]. A second concern is that the analyses are generally underpowered to detect interactions, particularly our analyses of combined adherence and gestational age at initiation categories, even when all trials were pooled [39]. In addition, the analyses conducted among MMS users were observational, and therefore, causal effects cannot be inferred due to a high likelihood of confounding. Our observational analyses related to the total number of tablets taken among MMS users are inherently biased, especially for outcomes like preterm birth, because a shorter gestation duration systematically limits the total number of MMS tablets a pregnant person could take. Thus, the observational analyses of tablet counts should be interpreted cautiously, as they will overestimate the effect of taking a greater number of tablets on any outcome related to gestation duration. Importantly, adherence and total number of tablets taken were postbaseline factors and therefore estimates should be interpreted with caution [40,41]. In addition, the relationships seen in the observational analyses should not be interpreted as causal because they are at risk of residual and unmeasured confounding. Last, we included trials only from LMIC settings, and therefore, our findings may not be directly generalizable to high-income country contexts.

Overall, we found that adherence influences the effect of MMS on birthweight and other maternal and infant outcomes. Although high adherence to supplementation is challenging to achieve, the adherence rates in the randomized trials included in this study demonstrate that it is possible in diverse settings. There are many potential ways to improve MMS adherence including education-based strategies, enhanced individual counseling (from health care workers, pharmacists, or community health workers), better training of health workers, improved packaging, and implementation of SMS reminders [42,43]. Programs should consider integrated strategies to support early ANC attendance and high supplement adherence to increase the effective coverage and impact of MMS.

## Author contributions

The authors’ responsibilities were as follows – ERS, CRS, FG, MWB: designed the study; FG, ZR: conducted the systematic literature searches, abstract screening, and risk of bias assessment of included trials; FG: set up the data use agreements with study authors, secured and checked the incoming data for completeness, and coordinated data harmonization efforts; all authors: had input, reviewed, and approved the final study protocol and statistical analysis plan and conducted or reviewed the statistical analyses for their respective trials; ERS, EMO, CRS: developed statistical program code for trial-specific analyses, as well as pooled the data and did the meta-analysis; ERS, CRS, FG, MWB: drafted the article and have primary responsibility for final content; and all authors: reviewed and approved the final manuscript.

## Funding

The project was supported by JBJ Foundation. The funder was not involved in the development of the protocol, conduct of the study, or interpretation of the findings.

## Conflict of interest

MWB reports financial support was provided by JBJ Foundation. ZAB is an Editorial Board Member for *Advances in Nutrition* and played no role in the Journal’s evaluation of the manuscript. The other authors report no conflicts of interest.
