# Supplementary material for: Contribution of Maternal Adherence to the Effect of Multiple Micronutrient Supplementation During Pregnancy: A Systematic Review and Individual Participant Data Meta-analysis
Source: Adv Nutr. 2025 May 30;16(7):100455. doi: 10.1016/j.advnut.2025.100455 (PMC12268027; doi:10.1016/j.advnut.2025.100455)

Contribution of maternal adherence to the effect of multiple micronutrient supplementation during pregnancy: a systematic review and individual participant data meta-analysis (Smith et al.)

Supplemental files for MMS Adherence IPD

Table of Contents

Supplemental Appendix 1. Medline search strategy 5
Supplemental Table 1. Proportion (percent) of participants in each adherence and tablets category, by study 7
Supplemental Table 2. Iron dose in MMS and IFA supplements provided by each included trial 8
Supplemental Table 3. Pooled effect size comparing MMS vs IFA, stratified by percent adherence groups - sensitivity analysis by MMS iron dose (MMS with lower dose than IFA comparison vs. same dose as IFA comparison) 9
Supplemental Table 4. Pooled effect size comparing MMS vs IFA, stratified by adherence and gestational age groups 10
Supplemental Table 5. Pooled effect size comparing MMS vs IFA, stratified by adherence and gestational age groups - sensitivity analysis by MMS iron dose (MMS with lower dose than IFA comparison vs. same dose as IFA comparison) 11
Supplemental Table 6. Pooled effect size comparing MMS vs IFA, stratified by tablet count groups - sensitivity analysis by MMS iron dose (MMS with lower dose than IFA comparison vs. same dose as IFA comparison) 12
Supplemental Table 7. Pooled association of adherence and gestational age group with adverse outcomes among MMS users (observational data) 13
Supplemental Figure 1. Risk of bias summary for each included study 14
Supplemental Figure 2. Proportion of included studies at low, unclear, or high risk of bias, stratified by category, across all included studies 15

Objective 1 Outcomes stratified by Adherence 16

Supplemental Figure 3.1. The effect of MMS vs. IFA on Anemia, stratified by Adherence 16
Supplemental Figure 3.2. The effect of MMS vs. IFA on Birthweight Percentile, stratified by Adherence 17
Supplemental Figure 3.3. The effect of MMS vs. IFA on Birthweight, stratified by Adherence 18
Supplemental Figure 3.4. The effect of MMS vs. IFA on Continuous Gestation stratified by Adherence 19
Supplemental Figure 3.5. The effect of MMS vs. IFA on Fetal Death, stratified by Adherence 20
Supplemental Figure 3.6. The effect of MMS vs. IFA on Hemoglobin Continuous, stratified by Adherence 21
Supplemental Figure 3.7. The effect of MMS vs. IFA on IDA, stratified by Adherence 22
Supplemental Figure 3.8. The effect of MMS vs. IFA on Infant Mortality, stratified by Adherence 23
Supplemental Figure 3.9. The effect of MMS vs. IFA on LGA90, stratified by Adherence 24
Supplemental Figure 3.10. The effect of MMS vs. IFA on Low Birthweight, stratified by Adherence 25
Supplemental Figure 3.11. The effect of MMS vs. IFA on Neonatal Mortality, stratified by Adherence 26
Supplemental Figure 3.12. The effect of MMS vs. IFA on Preterm, stratified by Adherence 27
Supplemental Figure 3.13. The effect of MMS vs. IFA on SGA10, stratified by Adherence 28
Supplemental Figure 3.14. The effect of MMS vs. IFA on Stillbirth, stratified by Adherence 29

Objective 1 Sensivity analysis by MMS iron dose stratified by Adherence 30

Supplemental Figure 3.15. The effect of MMS vs. IFA on hemoglobin, in trials with lower iron dose in MMS than IFA, stratified by adherence 30
Supplemental Figure 3.16. The effect of MMS vs. IFA on hemoglobin, in trials with same iron dose in MMS than IFA, stratified by adherence 31
Supplemental Figure 3.17. The effect of MMS vs. IFA on anemia, in trials with lower iron dose in MMS than IFA, stratified by adherence 32
Supplemental Figure 3.18. The effect of MMS vs. IFA on anemia, in trials with same iron dose in MMS than IFA, stratified by adherence 33
Supplemental Figure 3.19. The effect of MMS vs. IFA on IDA, in trials with lower iron dose in MMS than IFA, stratified by adherence 34

Supplemental Figure 3.20. The effect of MMS vs. IFA on IDA, in trials with same iron dose in MMS than IFA, stratified by adherence 34
Supplemental Figure 3.21. The effect of MMS vs. IFA on birthweight, in trials with lower iron dose in MMS than IFA, stratified by adherence 35
Supplemental Figure 3.22. The effect of MMS vs. IFA on birthweight, in trials with same iron dose in MMS than IFA, stratified by adherence 36

Objective 1 Outcomes stratified by Adherence and Gestational Age 37
Supplemental Figure 3.23. The effect of MMS vs. IFA on Anemia, stratified by Adherence and Gestational Age 37
Supplemental Figure 3.24. The effect of MMS vs. IFA on Birthweight Percentile, stratified by Adherence and Gestational Age 38
Supplemental Figure 3.25. The effect of MMS vs. IFA on Birthweight, stratified by Adherence and Gestational Age 39
Supplemental Figure 3.26. The effect of MMS vs. IFA on Continuous Gestation, stratified by Adherence and Gestational Age 40
Supplemental Figure 3.27. The effect of MMS vs. IFA on Fetal Death, stratified by Adherence and Gestational Age 41
Supplemental Figure 3.28. The effect of MMS vs. IFA on Hemoglobin Continuous, stratified by Adherence and Gestational Age 42
Supplemental Figure 3.29. The effect of MMS vs. IFA on IDA, stratified by Adherence and Gestational Age 43
Supplemental Figure 3.30. The effect of MMS vs. IFA on Infant Mortality, stratified by Adherence and Gestational Age 43
Supplemental Figure 3.32. The effect of MMS vs. IFA on LGA90, stratified by Adherence and Gestational Age 44
Supplemental Figure 3.32. The effect of MMS vs. IFA on Low Birthweight, stratified by Adherence and Gestational Age 45
Supplemental Figure 3.33. The effect of MMS vs. IFA on Neonatal Mortality, stratified by Adherence and Gestational Age 46
Supplemental Figure 3.34. The effect of MMS vs. IFA on Preterm, stratified by Adherence and Gestational Age 47
Supplemental Figure 3.35. The effect of MMS vs. IFA on SGA10, stratified by Adherence and Gestational Age 48
Supplemental Figure 3.36. The effect of MMS vs. IFA on Stillbirth, stratified by Adherence and Gestational Age 49

Objective 1 Sensivity analysis by MMS iron dose stratified by Adherence and Gestational Age 50
Supplemental Figure 3.37. The effect of MMS vs. IFA on hemoglobin, in trials with lower iron dose in MMS than IFA, stratified by adherence and gestational age ... 50
Supplemental Figure 3.38. The effect of MMS vs. IFA on anemia, in trials with lower iron dose in MMS than IFA, stratified by adherence and gestational age 51
Supplemental Figure 3.39. The effect of MMS vs. IFA on anemia, in trials with same iron dose in MMS than IFA, stratified by adherence and gestational age 52
Supplemental Figure 3.40. The effect of MMS vs. IFA on anemia, in trials with same iron dose in MMS than IFA, stratified by adherence and gestational age 53
Supplemental Figure 3.41. The effect of MMS vs. IFA on birthweight, in trials with same iron dose in MMS than IFA, stratified by adherence and gestational age 54

Objective 1 Outcomes stratified by Total Pill Count 55
Supplemental Figure 3.42. The effect of MMS vs. IFA on Anemia, stratified by Total Pill Count 55
Supplemental Figure 3.43. The effect of MMS vs. IFA on Birthweight Percentile, stratified by Total Pill Count 56
Supplemental Figure 3.44. The effect of MMS vs. IFA on Birthweight, stratified by Total Pill Count 57
Supplemental Figure 3.45. The effect of MMS vs. IFA on Continuous Gestation, stratified by Total Pill Count 58
Supplemental Figure 3.46. The effect of MMS vs. IFA on Fetal Death, stratified by Total Pill Count 59
Supplemental Figure 3.47. The effect of MMS vs. IFA on Hemoglobin Continuous, stratified by Total Pill Count 60
Supplemental Figure 3.48. The effect of MMS vs. IFA on IDA, stratified by Total Pill Count 61
Supplemental Figure 3.49. The effect of MMS vs. IFA on Infant Mortality, stratified by Total Pill Count 62
Supplemental Figure 3.50. The effect of MMS vs. IFA on LGA90, stratified by Total Pill Count 63
Supplemental Figure 3.51. The effect of MMS vs. IFA on Low Birthweight, stratified by Total Pill Count 64
Supplemental Figure 3.52. The effect of MMS vs. IFA on Neonatal Mortality, stratified by Total Pill Count 65
Supplemental Figure 3.53. The effect of MMS vs. IFA on Preterm, stratified by Total Pill Count 66
Supplemental Figure 3.54. The effect of MMS vs. IFA on SGA10, stratified by Total Pill Count 67
Supplemental Figure 3.55. The effect of MMS vs. IFA on Stillbirth, stratified by Total Pill Count 68

Objective 1 Sensivity analysis by MMS iron dose stratified by Total Pill Count 69
Supplemental Figure 3.56. The effect of MMS vs. IFA on hemoglobin, in trials with lower iron dose in MMS than IFA, stratified by total pill count 69

Supplemental Figure 3.57. The effect of MMS vs. IFA on hemoglobin, in trials with same iron dose in MMS than IFA, stratified by total pill count 70
Supplemental Figure 3.58. The effect of MMS vs. IFA on anemia, in trials with lower iron dose in MMS than IFA, stratified by total pill count 71
Supplemental Figure 3.59. The effect of MMS vs. IFA on anemia, in trials with same iron dose in MMS than IFA, stratified by total pill count 72
Supplemental Figure 3.60. The effect of MMS vs. IFA on IDA, in trials with lower iron dose in MMS than IFA, stratified by total pill count 72
Supplemental Figure 3.61. The effect of MMS vs. IFA on IDA, in trials with same iron dose in MMS than IFA, stratified by total pill count 73
Supplemental Figure 3.62. The effect of MMS vs. IFA on birthweight, in trials with lower iron dose in MMS than IFA, stratified by total pill count 74
Supplemental Figure 3.63. The effect of MMS vs. IFA on birthweight, in trials with same iron dose in MMS than IFA, stratified by total pill count 75

Objective 2 Outcomes stratified by Adherence 76

Supplemental Figure 4.1. The effect of MMS on Anemia, stratified by Adherence 76
Supplemental Figure 4.2. The effect of MMS on Birthweight Percentile, stratified by Adherence 77
Supplemental Figure 4.3. The effect of MMS on Birthweight, stratified by Adherence 78
Supplemental Figure 4.4. The effect of MMS on Continuous Gestation, stratified by Adherence 79
Supplemental Figure 4.5. The effect of MMS on Fetal Death, stratified by Adherence 80
Supplemental Figure 4.6. The effect of MMS on Hemoglobin Continuous, stratified by Adherence 81
Supplemental Figure 4.7. The effect of MMS on IDA, stratified by Adherence 82
Supplemental Figure 4.8. The effect of MMS on Infant Mortality, stratified by Adherence 82
Supplemental Figure 4.9. The effect of MMS on LGA90, stratified by Adherence 83
Supplemental Figure 4.10. The effect of MMS on Low Birthweight, stratified by Adherence 84
Supplemental Figure 4.11. The effect of MMS on Neonatal Mortality, stratified by Adherence 85
Supplemental Figure 4.12. The effect of MMS on Preterm, stratified by Adherence 86
Supplemental Figure 4.13. The effect of MMS on SGA10, stratified by Adherence 87
Supplemental Figure 4.14. The effect of MMS on Stillbirth, stratified by Adherence 88

Objective 2 Outcomes stratified by Adherence and Gestational Age 89

Supplemental Figure 4.15. The effect of MMS on Anemia, stratified by Adherence and Gestational Age 89
Supplemental Figure 4.16. The effect of MMS on Birthweight Percentile, stratified by Adherence and Gestational Age 90
Supplemental Figure 4.17. The effect of MMS on Birthweight, stratified by Adherence and Gestational Age 91
Supplemental Figure 4.18. The effect of MMS on Continuous Gestation, stratified by Adherence and Gestational Age 92
Supplemental Figure 4.19. The effect of MMS on Fetal Death, stratified by Adherence and Gestational Age 93
Supplemental Figure 4.20. The effect of MMS on Hemoglobin Continuous, stratified by Adherence and Gestational Age 94
Supplemental Figure 4.21. The effect of MMS on IDA stratified by Adherence and Gestational Age 95
Supplemental Figure 4.22. The effect of MMS on Infant Mortality, stratified by Adherence and Gestational Age 95
Supplemental Figure 4.23. The effect of MMS on LGA90, stratified by Adherence and Gestational Age 96
Supplemental Figure 4.24. The effect of MMS on Low Birthweight, stratified by Adherence and Gestational Age 97
Supplemental Figure 4.25. The effect of MMS on Neonatal Mortality, stratified by Adherence and Gestational Age 98
Supplemental Figure 4.26. The effect of MMS on Preterm, stratified by Adherence and Gestational Age 99
Supplemental Figure 4.27. The effect of MMS on SGA10, stratified by Adherence and Gestational Age 100
Supplemental Figure 4.28. The effect of MMS on Stillbirth, stratified by Adherence and Gestational Age 101

Objective 2 Outcomes stratified by Total Pill Count 102

Supplemental Figure 4.29. The effect of MMS on Anemia, stratified by Total Pill Count 102
Supplemental Figure 4.30. The effect of MMS on Birthweight Percentile, stratified by Total Pill Count 103
Supplemental Figure 4.31. The effect of MMS on Birthweight, stratified by Total Pill Count 104
Supplemental Figure 4.32. The effect of MMS on Continuous Gestation, stratified by Total Pill Count 105

Supplemental Figure 4.33. The effect of MMS on Fetal Death, stratified by Total Pill Count 106
Supplemental Figure 4.34. The effect of MMS on Hemoglobin Continuous, stratified by Total Pill Count 107
Supplemental Figure 4.35. The effect of MMS on IDA, stratified by Total Pill Count 108
Supplemental Figure 4.36. The effect of MMS on Infant Mortality, stratified by Total Pill Count 108
Supplemental Figure 4.37. The effect of MMS on LGA90, stratified by Total Pill Count 109
Supplemental Figure 4.38. The effect of MMS on Low Birthweight, stratified by Total Pill Count 110
Supplemental Figure 4.39. The effect of MMS on Neonatal Mortality, stratified by Total Pill Count 111
Supplemental Figure 4.40. The effect of MMS on Preterm, stratified by Total Pill Count 112
Supplemental Figure 4.41. The effect of MMS on SGA10, stratified by Total Pill Count 113
Supplemental Figure 4.42. The effect of MMS on Stillbirth, stratified by Total Pill Count 114

Supplemental Appendix 1. Medline search strategy

Medline (Ovid MEDLINE(R) ALL 1946 to February 01, 2022)

1. randomized controlled trial.mp. [mp=title, abstract, original title, name of substance word, subject heading word, floating sub-heading word, keyword heading word, organism supplementary concept word, protocol supplementary concept word, rare disease supplementary concept word, unique identifier, synonyms]
2. controlled clinical trial.mp. [mp=title, abstract, original title, name of substance word, subject heading word, floating sub-heading word, keyword heading word, organism supplementary concept word, protocol supplementary concept word, rare disease supplementary concept word, unique identifier, synonyms]
3. ?randomi*ed.mp. [mp=title, abstract, original title, name of substance word, subject heading word, floating sub-heading word, keyword heading word, organism supplementary concept word, protocol supplementary concept word, rare disease supplementary concept word, unique identifier, synonyms]
4. 1 or 2 or 3
5. exp PREGNANCY/
6. (pregnan* or maternal).mp. [mp=title, abstract, original title, name of substance word, subject heading word, floating sub-heading word, keyword heading word, organism supplementary concept word, protocol supplementary concept word, rare disease supplementary concept word, unique identifier, synonyms]
7. antenatal.mp. [mp=title, abstract, original title, name of substance word, subject heading word, floating sub-heading word, keyword heading word, organism supplementary concept word, protocol supplementary concept word, rare disease supplementary concept word, unique identifier, synonyms]
8. prenatal.mp. [mp=title, abstract, original title, name of substance word, subject heading word, floating sub-heading word, keyword heading word, organism supplementary concept word, protocol supplementary concept word, rare disease supplementary concept word, unique identifier, synonyms]
9. 5 or 6 or 7 or 8
10. ((micronutrient* or multivitamin*) adj3 supplement*).mp. [mp=title, abstract, original title, name of substance word, subject heading word, floating sub-heading word, keyword heading word, organism supplementary concept word, protocol supplementary concept word, rare disease supplementary concept word, unique identifier, synonyms]
11. (nutrition* adj3 supplement*).mp. [mp=title, abstract, original title, name of substance word, subject heading word, floating sub-heading word, keyword heading word, organism supplementary concept word, protocol supplementary concept word, rare disease supplementary concept word, unique identifier, synonyms]
12. (nutrient* adj3 supplement*).mp. [mp=title, abstract, original title, name of substance word, subject heading word, floating sub-heading word, keyword heading word, organism supplementary concept word, protocol supplementary concept word, rare disease supplementary concept word, unique identifier, synonyms]
13. (multiple* adj3 micronutrient*).mp. [mp=title, abstract, original title, name of substance word, subject heading word, floating sub-heading word,

keyword heading word, organism supplementary concept word, protocol supplementary concept word, rare disease supplementary concept word, unique identifier, synonyms]

14. (multi* adj3 supplement*).mp. [mp=title, abstract, original title, name of substance word, subject heading word, floating sub-heading word, keyword heading word, organism supplementary concept word, protocol supplementary concept word, rare disease supplementary concept word, unique identifier, synonyms]
15. 10 or 11 or 12 or 13 or 14
16. 4 and 9 and 15
17. exp animals/ not humans.sh.
18. 16 not 17
19. limit 18 to yr="2018 - 2022"

Supplemental Table 1. Proportion (percent) of participants in each adherence and tablets category, by study

	Adherence Categories					Tablet Count Categories				
	<60%	60-74%	75-89%	≥90%	Missing	<90 tablets	90-120 tablets	120-180 tablets	≥180 tablets	Missing
Adu-Afuwah, 2015	16	17	44	19	4	16	25	51	4	4
Ashorn , 2015	9	13	29	42	7	11	20	57	5	7
Bhutta, 2009	7	9	23	28	33	5	8	38	16	33
Bliznashka, 2022	1	1	15	82	1	12	15	45	27	1
Christian, 2003	19	9	23	49	0	17	10	33	41	0
Fawzi, 2007	7	9	18	63	4	24	30	40	2	4
Friis, 2004	8	6	8	20	58	38	4	0	0	58
Kaestel, 2005	30	18	16	15	21	48	17	13	1	21
Moore, 2009	0	3	16	80	1	1	2	53	41	4
Osrin, 2005	1	2	13	76	8	1	6	71	14	8
Roberfroid, 2008	9	19	17	49	5	29	18	29	19	5
Sunawang, 20009	25	15	23	33	4	23	19	45	9	4
Persson, 2012	24	10	13	19	35	15	7	20	21	36
West, 2014	7	5	17	71	0	8	5	27	59	0
Zagre, 2007	1	2	13	83	1	2	5	44	47	1

Supplemental Table 2. Iron dose in MMS and IFA supplements provided by each included trial

Study	Iron dose in MMS	Iron dose in IFA
Adu-Afarwuah, 2015	20 mg	60 mg
Ashorn, 2015	20mg	60mg
Bhutta, 2009	30mg	60mg
Bliznashka, 2022	30 mg	60 mg
Christian, 2003	60 mg	60 mg
Fawzi, 2007	60 mg	60 mg
Friis, 2004	not specified	not specified
Kaestel, 2005	30 mg (1 RDA)<br>30 mg (2 RDA)	60 mg
Moore, 2009	60 mg	60 mg
Osrin, 2005	30 mg	60 mg
Roberfroid, 2008	30 mg	60 mg
Sunawang, 2009	30 mg	60 mg
Persson, 2012	30mg	30 mg<br>60mg
West, 2014	27 mg	27 mg
Zagre, 2007	30 mg	60mg

MMS = prenatal micronutrient supplements, IFA = iron and folic acid supplements

Supplemental Table 3. Pooled effect size comparing MMS vs IFA, stratified by percent adherence groups - sensitivity analysis by MMS iron dose (MMS with lower dose than IFA comparison vs. same dose as IFA comparison)

Outcome	N participants	N Studies	MMS vs IFA <60% adherence *ES (95% CI)*	MMS vs IFA 60 - <75% adherence *ES (95% CI)*	MMS vs IFA 75 - <90% adherence *ES (95% CI)*	MMS vs IFA ≥90% adherence *ES (95% CI)*	P value for between sub-group heterogeneity
Lower iron dose in MMS than IFA							
3rd Trimester Hemoglobin (MD)	5,462	5	-0.06 (-0.21, 0.09)	-0.11 (-0.23, 0.01)	-0.18 (-0.29, -0.07)	-0.13 (-0.23, -0.04)	0.63
3rd Trimester Anemia (<11g/dL) (RR)	5,462	5	1.10 (0.97, 1.25)	1.15 (1.05, 1.26)	1.07 (0.95, 1.21)	1.06 (0.97, 1.15)	0.59
3rd Trimester IDA (RR)	433	1	1.08 (0.68, 1.71)	1.43 (0.91, 2.25)	0.57 (0.35, 0.93)	1.41 (1.17, 1.70)	0.007
Birthweight (MD)	12,890	9	7 (-33, 46)	56 (7, 106)	25 (-12, 62)	42 (14, 71)	0.37
Same iron dose in MMS as IFA							
3rd Trimester Hemoglobin (MD)	3,983	4	-0.06 (-0.23, 0.12)	-0.03 (-0.32, 0.26)	-0.19 (-0.36, -0.02)	-0.08 (-0.19, 0.03)	0.67
3rd Trimester Anemia (<11g/dL) (RR)	3,983	4	1.09 (0.90, 1.32)	1.09 (0.84, 1.42)	1.15 (0.97, 1.35)	1.05 (0.93, 1.18)	0.85
3rd Trimester IDA (RR)	1,771	2	0.96 (0.38, 2.39)	1.53 (0.63, 3.75)	0.97 (0.55, 1.70)	1.26 (0.87, 1.82)	0.78
Birthweight (g) (MD)	32,776	6	9 (-26, 43)	59 (21, 98)	39 (16, 62)	58 (46, 70)	0.04

CI = confidence interval, ES = effect size, IDA = iron deficiency anemia, MD = mean difference, MMS = prenatal micronutrient supplements, IFA = iron and folic acid supplements, RR = relative risk

Supplemental Table 4. Pooled effect size comparing MMS vs IFA, stratified by adherence and gestational age groups

Outcome	N participants	N Studies	<60% adherence, <20 weeks ES (95% CI)	<60% adherence, >20 weeks ES (95% CI)	60-74% adherence, <20 weeks ES (95% CI)	60-74% adherence, >20 weeks ES (95% CI)	75-90% adherence, <20 weeks ES (95% CI)	75-90% adherence, >20 weeks ES (95% CI)	≥90% adherence, <20 weeks ES (95% CI)	≥90% adherence, >20 weeks ES (95% CI)	P value for heterogeneity
Birthweight (g) (MD)	32725	6	19 (-25, 62)	19 (-50, 87)	64 (19, 109)	88 (20, 155)	45 (20, 71)	45 (-8, 99)	54 (41, 67)	77 (48, 107)	0.35
Low Birthweight (RR)	32725	6	0.97 (0.86, 1.1)	0.79 (0.54, 1.15)	0.89 (0.79, 1.02)	0.73 (0.49, 1.08)	0.88 (0.82, 0.95)	0.95 (0.74, 1.24)	0.88 (0.85, 0.92)	0.85 (0.75, 0.97)	0.77
Birthweight Percentile (MD)	32728	6	-4.03 (-6.82, -1.24)	1.44 (-3.18, 6.07)	2.24 (-0.64, 5.12)	4.52 (0.04, 8.99)	0.56 (-1.13, 2.24)	2.7 (-0.63, 6.04)	0.1 (-0.87, 1.06)	2.85 (0.99, 4.7)	**0.002**
Preterm Birth (RR)	38618	5	0.87 (0.76, 1)	1.12 (0.87, 1.46)	0.95 (0.81, 1.12)	1 (0.72, 1.38)	0.89 (0.8, 1)	0.95 (0.72, 1.25)	0.86 (0.81, 0.92)	0.94 (0.81, 1.09)	0.52
Gestational Age (MD)	40259	7	0.2 (-0.04, 0.44)	-0.04 (-0.48, 0.4)	0.18 (-0.07, 0.44)	0.06 (-0.3, 0.42)	0.18 (0.04, 0.33)	0.15 (-0.13, 0.44)	0.33 (0.23, 0.42)	0.26 (0.09, 0.43)	0.42
SGA <10th percentile (RR)	31970	6	1.12 (1.01, 1.23)	0.98 (0.77, 1.26)	1 (0.9, 1.11)	0.91 (0.73, 1.14)	0.95 (0.9, 1.01)	0.94 (0.8, 1.11)	0.97 (0.94, 1)	0.93 (0.85, 1.01)	0.17
LGA >90th percentile (RR)	29607	4	0.7 (0.49, 1)	0.99 (0.68, 1.44)	1.22 (0.86, 1.74)	0.98 (0.65, 1.49)	1.02 (0.81, 1.29)	1.2 (0.86, 1.66)	0.95 (0.84, 1.08)	1.06 (0.87, 1.29)	0.40
Fetal Death (RR)	42581	3	0.93 (0.82, 1.06)	0.82 (0.49, 1.37)	1.01 (0.82, 1.25)	0.5 (0.24, 1.04)	0.93 (0.79, 1.09)	0.82 (0.48, 1.39)	0.98 (0.92, 1.05)	0.82 (0.63, 1.05)	0.50
Stillbirth (RR)	39209	3	0.95 (0.67, 1.34)	0.82 (0.48, 1.39)	1.06 (0.68, 1.68)	0.5 (0.24, 1.04)	0.77 (0.6, 0.98)	0.84 (0.49, 1.45)	0.93 (0.81, 1.05)	0.79 (0.61, 1.02)	0.58
Neonatal Death (RR)	9308	2	1.04 (0.24, 4.58)	1.2 (0.46, 3.1)	0.98 (0.3, 3.21)	0.95 (0.37, 2.47)	0.81 (0.28, 2.38)	0.84 (0.39, 1.81)	0.64 (0.38, 1.07)	1.09 (0.73, 1.62)	0.88
Infant Death (RR)	2900	2	1.66 (0.71, 3.85)	0.9 (0.17, 4.76)	1.32 (0.53, 3.33)	0.69 (0.15, 3.15)	0.99 (0.44, 2.24)	1.72 (0.54, 5.46)	1.33 (0.84, 2.09)	2.63 (0.95, 7.27)	0.83
3rd Trimester Hemoglobin (MD)	1455	2	-0.19 (-0.74, 0.36)	0.83 (-1.47, 3.12)	0.09 (-0.3, 0.47)	0.58 (-0.32, 1.48)	-0.5 (-0.73, -0.27)	0.03 (-0.5, 0.56)	-0.19 (-0.45, 0.08)	-0.21 (-0.68, 0.26)	0.08
3rd Trimester Anemia (< 11g/dL) (RR)	2290	2	1.05 (0.6, 1.85)	1.01 (0.33, 3.07)	1.04 (0.66, 1.64)	1.02 (0.38, 2.78)	1.21 (0.94, 1.57)	1.32 (0.62, 2.82)	1.05 (0.91, 1.22)	0.89 (0.51, 1.55)	0.98
3rd Trimester IDA (RR)*	--	--	--	--	--	--	--	--	--	--	--

** This table presents the results of a fixed-effects meta-analysis for each outcome across four subgroups by adherence level. Each estimate (relative risk or mean difference) is the pooled effect size comparing MMS vs IFA*

CI = confidence interval, ES = effect size, IDA = iron deficiency anemia, LGA = large for gestational age, MD = mean difference, MMS = prenatal micronutrient supplements, IFA = iron and folic acid supplements, RR = relative risk, SGA = small for gestational age

Supplemental Table 5. Pooled effect size comparing MMS vs IFA, stratified by adherence and gestational age groups - sensitivity analysis by MMS iron dose (MMS with lower dose than IFA comparison vs. same dose as IFA comparison)

Outcome	N participants	N Studies	<60% adherence, <20 weeks *ES (95% CI)*	<60% adherence, >20 weeks *ES (95% CI)*	60-74% adherence, <20 weeks *ES (95% CI)*	60-74% adherence, >20 weeks *ES (95% CI)*	75-90% adherence, <20 weeks *ES (95% CI)*	75-90% adherence, >20 weeks *ES (95% CI)*	≥90% adherence, <20 weeks *ES (95% CI)*	≥90% adherence, >20 weeks *ES (95% CI)*	P value for between sub-group heterogeneity
Lower iron dose in MMS than IFA											
3rd Trimester Hemoglobin (MD)	1455	2	-0.19 (-0.74, 0.36)	0.83 (-1.47, 3.12)	0.09 (-0.30, 0.47)	0.58 (-0.32, 1.48)	-0.50 (-0.73, -0.27)	0.03 (-0.5, 0.56)	-0.19 (-0.45, 0.08)	-0.21 (-0.68, 0.26)	0.08
3rd Trimester Anemia (<11g/dL) (RR)	709	1	2.21 (0.80, 6.08)	0.50 (0.08, 3.20)	1.20 (0.54, 2.66)	1.04 (0.29, 3.72)	2.08 (1.27, 3.40)	0.80 (0.23, 2.80)	1.46 (0.75, 2.83)	0.36 (0.05, 2.63)	0.41
3rd Trimester IDA (RR)	--	--	--	--	--	--	--	--	--	--	--
Birthweight (g) (MD)	2788	3	84 (-41, 209)	30 (-94, 154)	93 (-7, 193)	111 (-13, 235)	53 (-21, 126)	43 (-70, 157)	-4 (-76, 68)	52 (-41, 144)	0.75
Same iron dose in MMS as IFA	--	--	--	--	--	--	--	--	--	--	--
3rd Trimester Hemoglobin (MD)	1581	1	0.75 (0.38, 1.48)	1.50 (0.38, 6.00)	0.98 (0.56, 1.69)	1.00 (0.20, 4.95)	0.99 (0.73, 1.34)	1.77 (0.69, 4.55)	1.03 (0.89, 1.21)	0.96 (0.53, 1.72)	0.93
3rd Trimester Anemia (<11g/dL) (RR)	--	--	--	--	--	--	--	--	--	--	--
3rd Trimester IDA (RR)	29937	3	10(-36, 56)	14 (-69, 96)	57 (7, 107)	78 (-2, 159)	44 (17, 72)	46 (-14, 106)	56 (42, 69)	80 (450, 111)	0.31

CI = confidence interval, ES = effect size, IDA = iron deficiency anemia, MD = mean difference, MMS = prenatal micronutrient supplements, IFA = iron and folic acid supplements, RR = relative risk

Supplemental Table 6. Pooled effect size comparing MMS vs IFA, stratified by tablet count groups - sensitivity analysis by MMS iron dose (MMS with lower dose than IFA comparison vs. same dose as IFA comparison)

Outcome	N participants	N studies	MMS vs IFA <90 total tablets<br>*ES (95% CI)*	MMS vs IFA 90-120 total tablets<br>*ES (95% CI)*	MMS vs IFA 120-180 total tablets<br>*ES (95% CI)*	MMS vs IFA ≥180 total tablets<br>*ES (95% CI)*	P value for between sub-group heterogeneity
Lower iron dose in MMS than IFA							
3rd Trimester Hemoglobin (MD)	6,821	6	-0.01 (-0.17, 0.15)	-0.11 (-0.24, 0.02)	-0.14 (-0.22, -0.05)	0.00 (-0.13, 0.13)	0.24
3rd Trimester Anemia (<11g/dL) (RR)	6,821	6	1.07 (0.94, 1.23)	1.07 (0.92, 1.24)	1.10 (1.01, 1.19)	0.98 (0.86, 1.12)	0.58
3rd Trimester IDA (RR)	433	1	1.11 (0.69, 1.78)	1.66 (1.16, 2.37)	1.08 (0.86, 1.36)	1.04 (0.71, 1.53)	0.21
Birthweight (g) (MD)	12,890	9	26 (-15, 66)	27 (-13, 67)	52 (24, 80)	22 (-10, 53)	0.49
Same iron dose in MMS as IFA	3,983	4	-0.10 (-0.34, 0.13)	-0.19 (-0.45, 0.08)	-0.07 (-0.20, 0.06)	-0.05 (-0.17, 0.07)	0.83
3rd Trimester Hemoglobin (MD)	3,983	4	1.07 (0.87, 1.31)	1.18 (0.92, 1.50)	1.02 (0.91, 1.15)	1.07 (0.93, 1.22)	0.76
3rd Trimester Anemia (<11g/dL) (RR)	1,771	2	1.09 (0.40, 2.98)	1.41 (0.57, 3.52)	1.03 (0.62, 1.72)	1.33 (0.89, 1.98)	0.87
3rd Trimester IDA (RR)	32,312	5	37 (4, 69)	70 (39, 100)	53 (35, 71)	47 (33, 61)	0.48

CI = confidence interval, ES = effect size, IDA = iron deficiency anemia, MD = mean difference, MMS = prenatal micronutrient supplements, IFA = iron and folic acid supplements, RR = relative risk

Supplemental Table 7. Pooled association of adherence and gestational age group with adverse outcomes among MMS users (observational data)

Outcome	N participants (all subgroups)	N Studies	<60% adherence, <20 weeks ES (95% CI)	<60% adherence, >20 weeks ES (95% CI)	60-74% adherence, <20 weeks ES (95% CI)	60-74% adherence, >20 weeks ES (95% CI)	75-90% adherence, <20 weeks Reference	75-90% adherence, >20 weeks ES (95% CI)	≥90% adherence, <20 weeks ES (95% CI)	≥90% adherence, >20 weeks ES (95% CI)
Birthweight (g) (MD)	17036	7	-17 (-48, 14)	-80 (-146, -14.50)	-12 (-43, 19)	24 (-38, 86)	*0.00*	-18 (-70, 33)	23 (4, 42)	-6 (-42, 30)
Low Birthweight (RR)	16276	5	0.97 (0.87, 1.08)	1.14 (0.94, 1.37)	0.97 (0.88, 1.08)	1.12 (0.91, 1.37)	*1.00*	0.96 (0.83, 1.11)	0.92 (0.87, 0.98)	1.01 (0.90, 1.14)
Birthweight Percentile (MD)	16635	7	-1.23 (-3.25, 0.79)	-6.91 (-10.89, -2.93)	1.02 (-1.19, 3.24)	-7.28 (-10.96, -3.59)	*0.00*	-6.2 (-9.4, -2.99)	0.15 (-1.10, 1.4)	-4.38 (-6.68, -2.08)
Preterm Birth (RR)	18847	4	1.14 (0.99, 1.31)	0.96 (0.73, 1.26)	1.15 (0.99, 1.34)	0.64 (0.47, 0.87)	*1.00*	0.55 (0.42, 0.73)	0.98 (0.89, 1.07)	0.69 (0.57, 0.83)
Gestational Age (MD)	20525	7	-0.22 (-0.40, -0.04)	0.36 (0.00, 0.72)	-0.20 (-0.40, 0.00)	0.96 (0.61, 1.31)	*0.00*	0.69 (0.4, 0.99)	0.02 (-0.10, 0.14)	0.75 (0.52, 0.97)
SGA <10th percentile (RR)	15875	5	1.03 (0.96, 1.11)	1.16 (0.94, 1.44)	1.00 (0.92, 1.09)	1.45 (1.25, 1.69)	*1.00*	1.22 (1.05, 1.42)	0.96 (0.92, 1)	1.09 (0.99, 1.19)
LGA >90th percentile (RR)	4863	3	0.92 (0.58, 1.45)	0.68 (0.47, 1.00)	1.15 (0.76, 1.74)	0.55 (0.37, 0.81)	*1.00*	0.59 (0.42, 0.83)	0.85 (0.62, 1.17)	0.50 (0.37, 0.69)
Fetal Death (RR)	21640	3	3.05 (2.64, 3.52)	1.44 (0.91, 2.27)	1.72 (1.44, 2.05)	0.62 (0.30, 1.28)	*1.00*	0.88 (0.56, 1.39)	1.37 (1.22, 1.54)	0.99 (0.77, 1.26)
Stillbirth (RR)	19930	3	1.29 (0.94, 1.77)	2.70 (1.64, 4.43)	1.08 (0.76, 1.53)	1.05 (0.50, 2.21)	*1.00*	1.84 (1.13, 2.99)	1.01 (0.84, 1.23)	2.01 (1.49, 2.73)
Neonatal Death (RR)	4922	2	1.71 (0.38, 7.62)	1.19 (0.37, 3.85)	1.51 (0.41, 5.59)	1.25 (0.41, 3.82)	*1.00*	1.13 (0.43, 3.01)	1.44 (0.59, 3.53)	1.45 (0.62, 3.4)
Infant Death (RR)	1494	2	1.81 (0.90, 3.64)	1.38 (0.31, 6.13)	1.41 (0.61, 3.26)	2.09 (0.52, 8.39)	*1.00*	2.61 (1.02, 6.67)	1.44 (0.78, 2.65)	2.06 (0.92, 4.62)
3rd Trimester Hemoglobin (MD)	1502	3	0.04 (-0.20, 0.29)	0.91 (0.22, 1.59)	0.28 (0.01, 0.55)	-1.04 (-1.23, -0.84)	*0.00*	0.16 (-0.18, 0.49)	0.07 (-0.09, 0.24)	0.16 (-0.15, 0.48)
3rd Trimester Anemia (<11g/dL) (RR)*	--	--	--	--	--	--	--	--	--	--
3rd Trimester IDA (RR)*	--	--	--	--	--	--	--	--	--	--

*Outcomes marked with an asterisk (*) had insufficient data for analysis.

CI = confidence interval, ES = effect size, IDA = iron deficiency anemia, LGA = large for gestational age, MD = mean difference, MMS = prenatal micronutrient supplements, IFA = iron and folic acid supplements, RR = relative risk, SGA = small for gestational age

Supplemental Figure 1. Risk of bias summary for each included study

	Random sequence generation (selection bias)	Allocation concealment (selection bias)	Blinding of participants and personnel (performance bias)	Blinding of outcome assessment (detection bias)	Incomplete outcome data (attrition bias)	Selective reporting (reporting bias)	Other bias
Ashorn 2015	+	+	+	+	+	+	+
Bhutta 2009	+	+	+	+	+	+	+
Bliznashka, 2021	+	+	+	+	+	+	+
Christian 2003	+	+	+	+	+	+	+
Dewey, 2009	+	+	+	+	+	+	+
Fawzi 2007	+	+	+	+	+	+	+
Friis 2004	+	+	+	+	?	+	+
Kaestel 2005	+	?	+	+	−	+	+
Moore 2009	+	+	+	+	+	+	+
Osrin 2005	+	+	+	+	+	+	+
Persson 2012	+	?	+	+	−	+	+
Roberfroid 2008	+	+	+	+	+	+	+
Sunawang 2009	+	?	?	?	+	+	+
West 2014	+	+	+	+	+	+	+
Zagre 2007	+	?	?	+	?	+	+

Supplemental Figure 2. Proportion of included studies at low, unclear, or high risk of bias, stratified by category, across all included studies

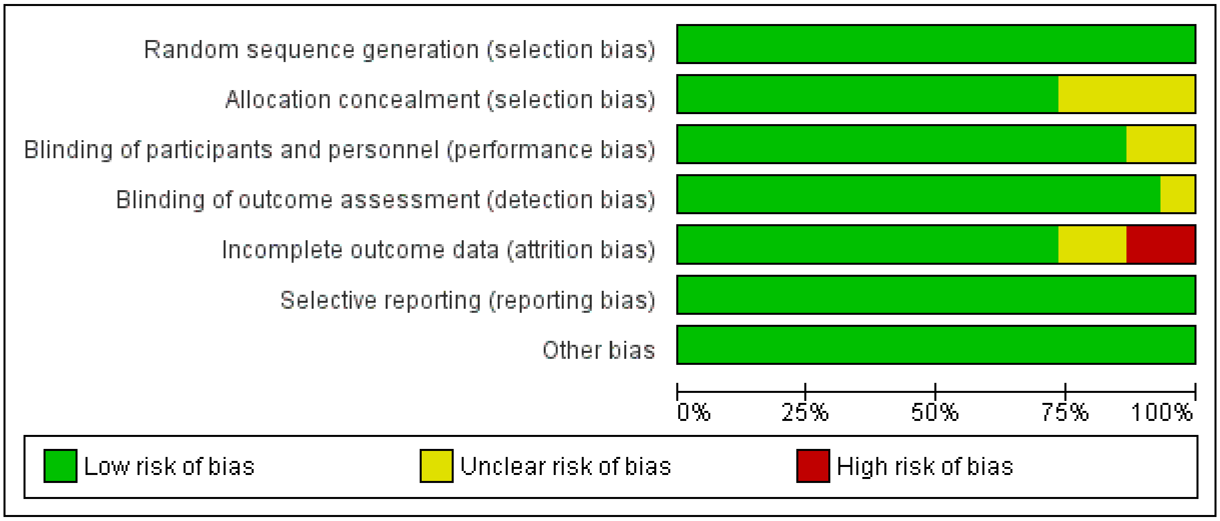

Objective 1 Outcomes stratified by Adherence

Supplemental Figure 3.1. The effect of MMS vs. IFA on Anemia, stratified by Adherence

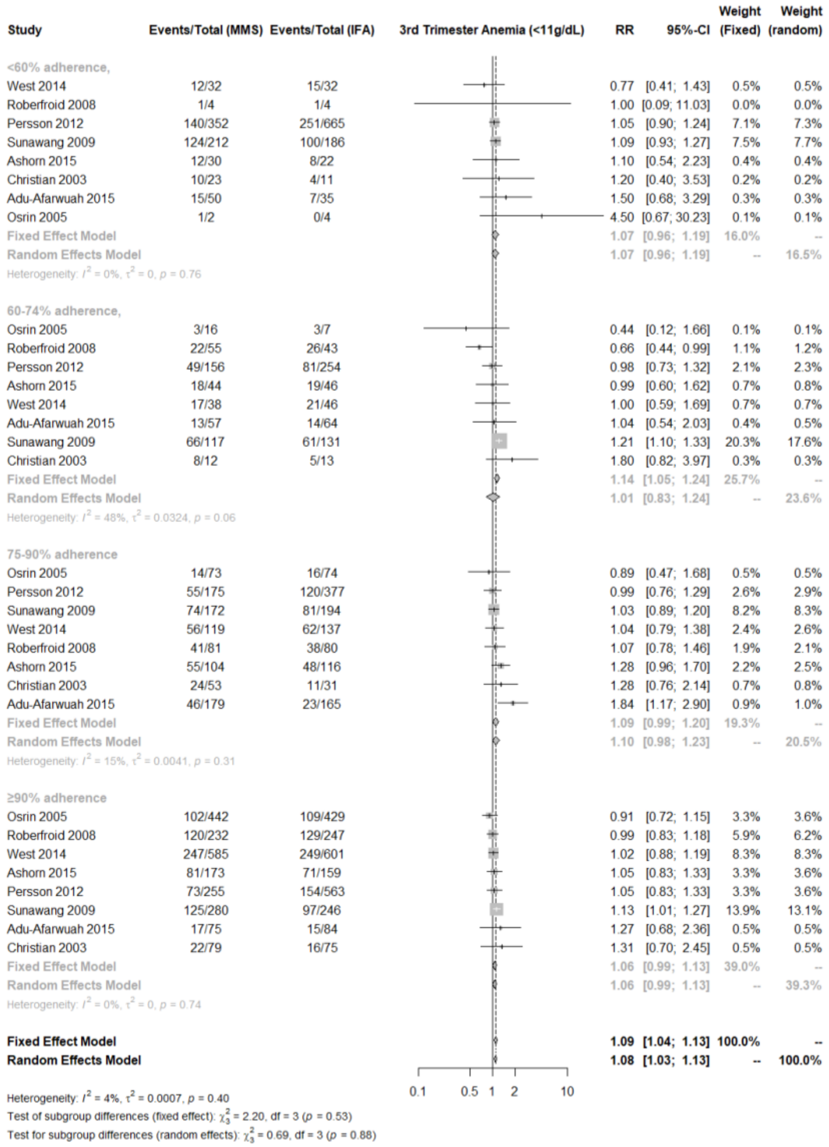

Supplemental Figure 3.2. The effect of MMS vs. IFA on Birthweight Percentile, stratified by Adherence

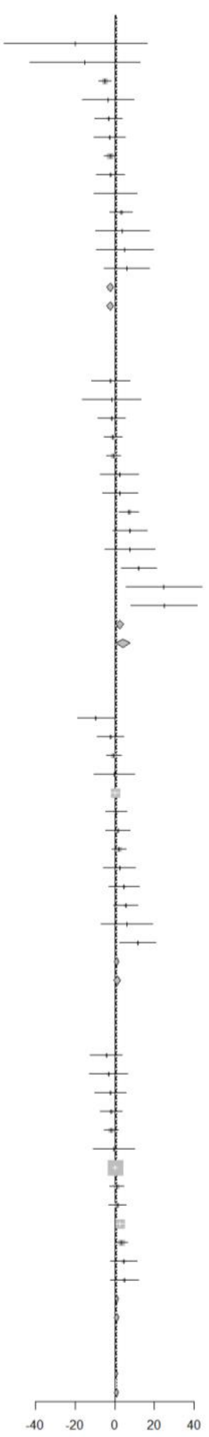

Study	Total MMS	MMS Mean (SD)	Total IFA	IFA Mean (SD)	MD	95%-CI	Weight (Fixed)	Weight (random)
**<60% adherence,**								
Zagre 2007	12	52 (39.8)	8	71.9 (43.9)	-19.87	[-55.89; 16.15]	0.0%	0.1%
Osrin 2005	5	17.3 (13.5)	5	32.4 (23.4)	-15.10	[-42.90; 12.80]	0.0%	0.1%
West 2014	551	17.85 (25.52)	463	22.87 (29.45)	-5.02	[-8.28; -1.75]	3.3%	4.0%
Ashorn 2015	33	27.3 (19.9)	26	30.6 (29.9)	-3.30	[-16.30; 9.70]	0.2%	0.6%
Christian 2003	150	20.9 (28.7)	150	24.1 (32.4)	-3.20	[-10.22; 3.81]	0.7%	1.6%
Kaestel 2005	216	43.3 (33.5)	99	46 (33)	-2.70	[-10.70; 5.30]	0.6%	1.3%
Persson 2012	355	16.5 (20.6)	675	19 (23.7)	-2.40	[-5.40; 0.50]	4.1%	4.4%
Sunawang 2009	206	49.4 (34.4)	190	51.5 (33.5)	-2.11	[-9.23; 5.00]	0.7%	1.6%
Adu-Afarwuah 2015	63	31.7 (28.7)	48	31.6 (28.5)	0.20	[-10.70; 11.00]	0.3%	0.8%
Fawzi 2007	274	50.6 (33.9)	269	47.4 (34.5)	3.20	[-2.60; 8.90]	1.1%	2.1%
Friis 2004	52	39 (31)	31	35.3 (27.9)	3.80	[-9.70; 17.30]	0.2%	0.5%
Roberfroid 2008	38	34.8 (31.7)	38	29.7 (30.5)	5.00	[-9.20; 19.30]	0.2%	0.5%
Bhutta 2009	79	56.6 (39.1)	45	50.5 (38.2)	6.06	[-5.32; 17.44]	0.3%	0.7%
Fixed Effect Model					-2.31	[-4.05; -0.56]	11.6%	--
Random Effects Model					-2.29	[-4.05; -0.53]	--	18.3%
Heterogeneity: $I^2 = 1\%$, $\tau^2 = 0.0783$, $p = 0.44$								
**60-74% adherence,**								
Christian 2003	70	23.8 (31.7)	66	25.9 (30.4)	-2.16	[-11.88; 7.57]	0.4%	0.9%
Friis 2004	33	41.1 (30.1)	40	42.8 (32)	-1.60	[-16.30; 13.00]	0.2%	0.4%
Sunawang 2009	117	51.4 (34.6)	132	52.9 (32.4)	-1.54	[-8.43; 5.35]	0.7%	1.6%
Persson 2012	156	18.1 (22.5)	261	19.1 (22.3)	-0.90	[-5.40; 3.50]	1.8%	3.0%
West 2014	461	17.36 (26.09)	403	18.15 (25.79)	-0.79	[-4.30; 2.72]	2.9%	3.8%
Ashorn 2015	50	32.6 (25.2)	55	30.3 (23.8)	2.30	[-7.20; 11.80]	0.4%	1.0%
Kaestel 2005	175	42.9 (33.2)	79	40.3 (33.8)	2.60	[-6.30; 11.60]	0.4%	1.1%
Fawzi 2007	345	51.9 (33)	326	44.7 (32.9)	7.10	[2.10; 12.10]	1.4%	2.6%
Roberfroid 2008	99	41.7 (31.8)	96	34.1 (28.7)	7.60	[-1.00; 16.10]	0.5%	1.2%
Bhutta 2009	85	67.1 (35.7)	105	59.5 (38.3)	7.69	[-4.84; 20.22]	0.2%	0.6%
Adu-Afarwuah 2015	63	40.9 (26)	69	28.8 (26.7)	12.10	[3.10; 21.20]	0.4%	1.1%
Osrin 2005	16	36.4 (29.6)	11	11.6 (8.5)	24.70	[5.80; 43.70]	0.1%	0.3%
Zagre 2007	36	70.5 (33)	22	45.7 (37.5)	24.77	[8.19; 41.35]	0.1%	0.4%
Fixed Effect Model					2.33	[0.40; 4.25]	9.5%	--
Random Effects Model					4.08	[0.58; 7.57]	--	17.9%
Heterogeneity: $I^2 = 60\%$, $\tau^2 = 20.6268$, $p < 0.01$								
**75-90% adherence**								
Zagre 2007	240	60.6 (36.7)	114	70.2 (34.5)	-9.60	[-18.95; -0.25]	0.4%	1.0%
Christian 2003	227	25.1 (31.2)	150	27.3 (30.9)	-2.15	[-8.79; 4.50]	0.8%	1.7%
Persson 2012	175	16 (20.6)	386	16.6 (20.7)	-0.60	[-4.30; 3.10]	2.6%	3.6%
Bhutta 2009	229	63.8 (37.2)	283	64 (36.7)	-0.20	[-10.49; 10.09]	0.3%	0.9%
West 2014	1600	18.11 (25.89)	1637	17.87 (26.37)	0.22	[-1.70; 2.14]	9.6%	5.5%
Adu-Afarwuah 2015	185	34.7 (24.7)	180	34.2 (27.2)	0.50	[-4.80; 5.90]	1.2%	2.4%
Osrin 2005	75	19.2 (18.3)	80	17.7 (20.6)	1.50	[-4.70; 7.70]	0.9%	1.9%
Fawzi 2007	661	47.2 (32.7)	684	45.1 (33.2)	2.10	[-1.40; 5.60]	2.9%	3.8%
Sunawang 2009	172	52.5 (34.5)	191	50.1 (32.6)	2.40	[-5.67; 10.48]	0.5%	1.3%
Roberfroid 2008	96	32.3 (26.6)	93	27.6 (27.6)	4.60	[-3.10; 12.40]	0.6%	1.4%
Ashorn 2015	114	35.8 (26.3)	131	30.3 (23.3)	5.50	[-0.80; 11.70]	0.9%	1.9%
Friis 2004	44	46.1 (31.6)	43	40 (29.6)	6.20	[-6.90; 19.20]	0.2%	0.6%
Kaestel 2005	153	48.3 (34.9)	85	36.8 (32.8)	11.50	[2.40; 20.60]	0.4%	1.1%
Fixed Effect Model					0.85	[-0.44; 2.13]	21.4%	--
Random Effects Model					1.15	[-0.63; 2.92]	--	27.0%
Heterogeneity: $I^2 = 26\%$, $\tau^2 = 2.4157$, $p = 0.18$								
**≥90% adherence**								
Adu-Afarwuah 2015	72	29.9 (24.5)	83	34.2 (26.2)	-4.30	[-12.40; 3.80]	0.5%	1.3%
Bhutta 2009	290	62.9 (36.8)	348	66.1 (36.8)	-3.21	[-12.93; 6.52]	0.4%	0.9%
Friis 2004	111	42.3 (28.5)	97	44.5 (29.4)	-2.20	[-10.10; 5.80]	0.6%	1.3%
Ashorn 2015	180	30.6 (26.7)	169	32.4 (27.2)	-1.80	[-7.50; 3.80]	1.1%	2.2%
Persson 2012	254	19 (22.6)	574	20.8 (24.2)	-1.80	[-5.30; 1.80]	2.8%	3.7%
Kaestel 2005	127	42.7 (34.8)	70	43.1 (35.7)	-0.50	[-10.80; 9.90]	0.3%	0.8%
West 2014	7547	19.44 (26.45)	7592	19.44 (27.05)	0.16	[-0.91; 1.22]	31.1%	6.4%
Christian 2003	406	24.3 (29.8)	381	23.3 (27.7)	0.99	[-2.47; 4.44]	3.0%	3.8%
Roberfroid 2008	272	29.4 (27.1)	277	28.3 (25.2)	1.20	[-3.20; 5.50]	1.9%	3.0%
Fawzi 2007	2407	45.9 (32.2)	2374	43.4 (32.7)	2.50	[0.60; 4.30]	10.3%	5.6%
Osrin 2005	453	22.7 (23)	446	19.4 (21.8)	3.30	[0.40; 6.30]	4.1%	4.4%
Zagre 2007	1139	64.9 (35.6)	1145	60.4 (36.3)	4.44	[-2.23; 11.11]	0.8%	1.7%
Sunawang 2009	283	54.9 (34.2)	244	50.1 (34.1)	4.79	[-2.44; 12.02]	0.7%	1.5%
Fixed Effect Model					0.77	[-0.02; 1.55]	57.5%	--
Random Effects Model					0.91	[-0.23; 2.06]	--	36.9%
Heterogeneity: $I^2 = 22\%$, $\tau^2 = 0.8237$, $p = 0.22$								
**Fixed Effect Model**					**0.57**	**[-0.02; 1.17]**	**100.0%**	**--**
**Random Effects Model**					**0.84**	**[-0.17; 1.86]**	**--**	**100.0%**

Heterogeneity: $I^2 = 42\%$, $\tau^2 = 3.8637$, $p < 0.01$
Test of subgroup differences (fixed effect): $\chi^2_3 = 14.09$, df = 3 ($p < 0.01$)
Test for subgroup differences (random effects): $\chi^2_3 = 14.64$, df = 3 ($p < 0.01$)

Supplemental Figure 3.3. The effect of MMS vs. IFA on Birthweight, stratified by Adherence

Study	Total MMS	MMS Mean (SD)	Total IFA	IFA Mean (SD)	MD	95%-CI	Weight (Fixed)	Weight (random)
<60% adherence,								
Osrin 2005	5	2704 (218)	5	2830 (645)	-126.00	[-828.00; 576.00]	0.0%	0.0%
Ashorn 2015	33	2859 (482)	27	2964 (478)	-106.00	[-355.00; 144.00]	0.1%	0.2%
Bliznashka 2022	2	2750 (1061)	4	2838 (390)	-87.50	[-439.03; 264.03]	0.1%	0.1%
Sunawang 2009	206	3054 (463)	190	3100 (419)	-46.14	[-107.61; 15.34]	2.0%	2.7%
Zagre 2007	12	3008 (363)	8	3025 (587)	-16.67	[-509.27; 475.94]	0.0%	0.0%
West 2014	574	2568 (395)	486	2565 (424)	3.13	[-46.65; 52.91]	3.0%	4.0%
Persson 2012	360	2658 (405)	688	2651 (438)	7.00	[-48.00; 61.00]	2.5%	3.4%
Friis 2004	53	3104 (477)	32	3089 (385)	15.00	[-183.00; 213.00]	0.2%	0.3%
Christian 2003	150	2564 (493)	151	2544 (458)	20.54	[-101.11; 142.20]	0.5%	0.7%
Adu-Afarwuah 2015	63	2897 (552)	48	2864 (497)	33.00	[-168.00; 234.00]	0.2%	0.3%
Fawzi 2007	274	3104 (478)	269	3068 (487)	36.00	[-45.00; 118.00]	1.1%	1.6%
Kaestel 2005	231	3074 (470)	104	3018 (468)	57.00	[-52.00; 166.00]	0.6%	0.9%
Roberfroid 2008	40	3004 (415)	42	2900 (410)	104.00	[-78.00; 285.00]	0.2%	0.3%
Bhutta 2009	81	3046 (574)	47	2899 (530)	146.74	[13.91; 279.57]	0.4%	0.6%
Fixed Effect Model					8.78	[-17.26; 34.82]	11.0%	--
Random Effects Model					8.78	[-17.26; 34.82]	--	15.1%
Heterogeneity: $I^2 = 0\%$, $\tau^2 = 0$, $p = 0.62$								
60-74% adherence,								
Zagre 2007	38	2942 (440)	23	3074 (568)	-131.81	[-413.48; 149.86]	0.1%	0.1%
Christian 2003	70	2521 (507)	66	2577 (510)	-56.40	[-227.70; 114.90]	0.3%	0.4%
Sunawang 2009	117	3108 (462)	132	3121 (451)	-12.71	[-114.79; 89.36]	0.7%	1.0%
Persson 2012	161	2690 (434)	266	2689 (409)	0.00	[-82.00; 82.00]	1.1%	1.6%
Kaestel 2005	185	3089 (486)	86	3026 (407)	63.00	[-56.00; 182.00]	0.5%	0.8%
West 2014	482	2559 (383)	421	2491 (425)	65.38	[11.16; 119.60]	2.5%	3.4%
Ashorn 2015	50	3021 (450)	55	2943 (422)	78.00	[-91.00; 246.00]	0.3%	0.4%
Fawzi 2007	345	3218 (507)	326	3137 (483)	81.00	[6.00; 156.00]	1.3%	1.9%
Roberfroid 2008	104	2919 (477)	102	2808 (457)	112.00	[-17.00; 240.00]	0.5%	0.7%
Bhutta 2009	85	3099 (746)	108	2954 (530)	144.75	[-32.52; 322.01]	0.2%	0.4%
Adu-Afarwuah 2015	63	3084 (430)	69	2938 (471)	145.00	[-10.00; 301.00]	0.3%	0.5%
Friis 2004	34	3207 (443)	40	3058 (417)	149.00	[-51.00; 349.00]	0.2%	0.3%
Bliznashka 2022	5	3140 (586)	3	2967 (289)	173.33	[-384.96; 731.63]	0.0%	0.0%
Osrin 2005	16	3021 (513)	11	2749 (302)	272.00	[-84.00; 627.00]	0.1%	0.1%
Fixed Effect Model					57.93	[27.57; 88.29]	8.1%	--
Random Effects Model					57.93	[27.57; 88.29]	--	11.4%
Heterogeneity: $I^2 = 0\%$, $\tau^2 = 0$, $p = 0.46$								
75-90% adherence								
Zagre 2007	246	3078 (641)	119	3125 (568)	-47.57	[-174.77; 79.63]	0.5%	0.7%
Persson 2012	180	2661 (399)	389	2685 (400)	-24.00	[-95.00; 47.00]	1.5%	2.1%
Sunawang 2009	173	3075 (445)	191	3073 (428)	1.53	[-82.20; 85.26]	1.1%	1.5%
Roberfroid 2008	97	2904 (490)	93	2890 (510)	13.00	[-130.00; 156.00]	0.4%	0.5%
Friis 2004	44	3136 (435)	44	3123 (398)	13.00	[-164.00; 190.00]	0.2%	0.4%
Ashorn 2015	114	2991 (493)	131	2971 (362)	20.00	[-88.00; 128.00]	0.6%	0.9%
Bliznashka 2022	111	3020 (449)	120	2997 (409)	23.25	[-93.86; 140.36]	0.5%	0.8%
West 2014	1685	2557 (425)	1701	2521 (405)	35.91	[7.57; 64.24]	9.3%	9.7%
Adu-Afarwuah 2015	185	3015 (359)	180	2961 (422)	54.00	[-27.00; 134.00]	1.2%	1.6%
Osrin 2005	75	2785 (447)	81	2720 (416)	66.00	[-71.00; 202.00]	0.4%	0.6%
Christian 2003	227	2744 (455)	150	2676 (442)	67.78	[-30.28; 165.85]	0.8%	1.1%
Fawzi 2007	661	3174 (524)	684	3102 (522)	72.00	[16.00; 128.00]	2.4%	3.2%
Kaestel 2005	162	3086 (520)	88	3007 (473)	79.00	[-52.00; 211.00]	0.4%	0.6%
Bhutta 2009	232	3016 (620)	287	2873 (570)	142.80	[3.70; 281.89]	0.4%	0.6%
Fixed Effect Model					36.35	[16.84; 55.86]	19.6%	--
Random Effects Model					36.35	[16.84; 55.86]	--	24.3%
Heterogeneity: $I^2 = 0\%$, $\tau^2 = 0$, $p = 0.66$								
≥90% adherence								
Friis 2004	115	3046 (439)	102	3052 (407)	-6.00	[-120.00; 108.00]	0.6%	0.8%
Roberfroid 2008	273	2902 (439)	279	2900 (385)	2.00	[-67.00; 71.00]	1.6%	2.2%
Christian 2003	407	2681 (438)	384	2673 (428)	8.34	[-47.27; 63.96]	2.4%	3.2%
Persson 2012	258	2718 (440)	579	2706 (424)	11.00	[-52.00; 74.00]	1.9%	2.6%
Adu-Afarwuah 2015	72	2968 (382)	83	2946 (457)	22.00	[-113.00; 156.00]	0.4%	0.6%
Ashorn 2015	180	2962 (447)	169	2939 (481)	24.00	[-74.00; 121.00]	0.8%	1.1%
Bhutta 2009	291	2916 (555)	349	2885 (550)	30.84	[-57.05; 118.73]	1.0%	1.4%
Sunawang 2009	283	3100 (419)	244	3066 (408)	33.96	[-41.21; 109.13]	1.3%	1.9%
West 2014	7898	2594 (404)	7918	2534 (416)	60.10	[46.13; 74.06]	38.3%	20.4%
Kaestel 2005	137	3079 (509)	76	3015 (504)	64.00	[-79.00; 207.00]	0.4%	0.5%
Zagre 2007	1161	3082 (537)	1177	3011 (507)	70.53	[-26.20; 167.25]	0.8%	1.2%
Fawzi 2007	2407	3170 (493)	2374	3095 (510)	75.00	[47.00; 104.00]	9.2%	9.6%
Osrin 2005	453	2813 (452)	447	2735 (424)	78.00	[21.00; 135.00]	2.3%	3.1%
Bliznashka 2022	678	3129 (485)	747	3043 (438)	85.81	[-66.25; 237.87]	0.3%	0.5%
Fixed Effect Model					55.89	[44.85; 66.94]	61.2%	--
Random Effects Model					55.89	[44.85; 66.94]	--	49.1%
Heterogeneity: $I^2 = 0\%$, $\tau^2 = 0$, $p = 0.49$								
**Fixed Effect Model**					**47.03**	**[38.39; 55.67]**	**100.0%**	**--**
**Random Effects Model**					**42.34**	**[31.75; 52.92]**	**--**	**100.0%**

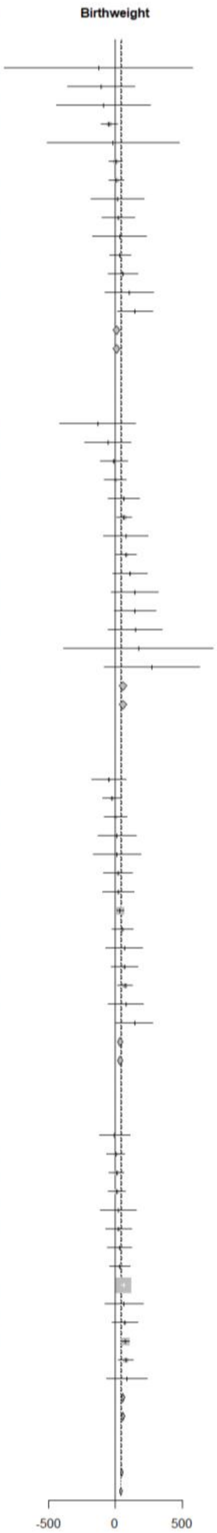

Heterogeneity: $I^2 = 7\%$, $\tau^2 = 92.5086$, $p = 0.33$

Test of subgroup differences (fixed effect): $\chi^2_3 = 12.41$, df = 3 ($p < 0.01$)

Test for subgroup differences (random effects): $\chi^2_3 = 12.41$, df = 3 ($p < 0.01$)

Supplemental Figure 3.4. The effect of MMS vs. IFA on Continuous Gestation stratified by Adherence

Study	Total MMS	MMS Mean (SD)	Total IFA	IFA Mean (SD)	MD	95%-CI	Weight (Fixed)	Weight (random)
<60% adherence,								
Ashorn 2015	45	39 (1.8)	36	39.2 (1.8)	-0.20	[-1.00; 0.60]	0.5%	0.6%
Fawzi 2007	281	38.8 (3.2)	282	38.9 (3.2)	-0.10	[-0.60; 0.40]	1.2%	1.4%
Adu-Afarwuah 2015	69	39 (2.4)	55	39 (2.3)	0.00	[-0.80; 0.80]	0.5%	0.6%
Friis 2004	62	40.2 (4.1)	42	40.2 (1.8)	0.00	[-1.40; 1.30]	0.2%	0.2%
Bhutta 2009	80	37.5 (4.1)	47	37.5 (3.6)	0.04	[-1.18; 1.25]	0.2%	0.2%
Sunawang 2009	206	38.3 (3)	191	38.3 (3.1)	0.06	[-0.73; 0.85]	0.5%	0.6%
Roberfroid 2008	47	39.3 (3.4)	50	39.2 (3.9)	0.10	[-1.40; 1.60]	0.1%	0.2%
West 2014	952	38.73 (3.05)	806	38.56 (3.23)	0.18	[-0.11; 0.47]	3.5%	4.0%
Persson 2012	355	39.5 (2.2)	675	39.2 (2.4)	0.20	[-0.10; 0.50]	3.2%	3.7%
Osrin 2005	5	38.7 (2.3)	5	38.5 (1.7)	0.20	[-2.80; 3.10]	0.0%	0.0%
Kaestel 2005	362	39.1 (4.2)	152	38.6 (3.9)	0.50	[-0.20; 1.30]	0.5%	0.6%
Christian 2003	150	39.1 (3.1)	154	38.6 (3.6)	0.53	[-0.19; 1.25]	0.6%	0.7%
Zagre 2007	12	37.7 (2.9)	8	34.7 (3.5)	2.95	[0.29; 5.62]	0.0%	0.1%
Fixed Effect Model					0.16	[0.00; 0.33]	11.0%	--
Random Effects Model					0.16	[0.00; 0.33]	--	12.7%
Heterogeneity: $I^2 = 0\%$, $\tau^2 = 0$, $p = 0.77$								
60-74% adherence,								
Zagre 2007	38	35.8 (3.3)	23	38.3 (5)	-2.49	[-4.50; -0.47]	0.1%	0.1%
Osrin 2005	16	39.5 (1.7)	11	40 (1.3)	-0.50	[-1.80; 0.70]	0.2%	0.2%
Bhutta 2009	86	36.4 (4.3)	108	36.6 (4.3)	-0.17	[-1.97; 1.63]	0.1%	0.1%
Christian 2003	71	38.4 (4)	66	38.4 (3.4)	-0.02	[-1.28; 1.23]	0.2%	0.2%
Fawzi 2007	351	39.4 (3)	337	39.5 (3)	0.00	[-0.50; 0.40]	1.4%	1.7%
Adu-Afarwuah 2015	68	39.5 (1.5)	73	39.4 (1.9)	0.10	[-0.50; 0.60]	1.0%	1.2%
West 2014	757	38.78 (3.20)	644	38.60 (3.24)	0.19	[-0.16; 0.53]	2.4%	2.8%
Persson 2012	156	39.3 (1.8)	261	39.1 (2)	0.20	[-0.20; 0.60]	1.8%	2.1%
Kaestel 2005	239	39.6 (3.5)	105	39.3 (3.7)	0.30	[-0.50; 1.20]	0.4%	0.5%
Roberfroid 2008	119	38.6 (3.3)	119	38.3 (3.2)	0.30	[-0.50; 1.10]	0.5%	0.6%
Sunawang 2009	117	38.6 (3.1)	132	38.2 (3.2)	0.33	[0.02; 0.64]	3.0%	3.4%
Ashorn 2015	56	39.7 (1.8)	64	39.1 (2.5)	0.60	[-0.20; 1.40]	0.5%	0.6%
Friis 2004	36	40.2 (1.9)	45	39.6 (2.5)	0.60	[-0.40; 1.60]	0.3%	0.4%
Fixed Effect Model					0.20	[0.04; 0.35]	11.9%	--
Random Effects Model					0.20	[0.04; 0.35]	--	13.8%
Heterogeneity: $I^2 = 0\%$, $\tau^2 = 0$, $p = 0.48$								
75-90% adherence								
Roberfroid 2008	118	39.1 (3.5)	111	39.7 (3.2)	-0.60	[-1.50; 0.20]	0.4%	0.5%
Friis 2004	52	39 (2.8)	55	39.5 (2.6)	-0.50	[-1.60; 0.50]	0.3%	0.3%
Sunawang 2009	172	38.2 (3.1)	191	38.4 (3)	-0.26	[-0.77; 0.25]	1.1%	1.3%
Kaestel 2005	192	39.4 (3.8)	111	39.7 (3.3)	-0.20	[-1.00; 0.60]	0.5%	0.6%
Ashorn 2015	130	39.3 (2.4)	136	39.5 (1.6)	-0.20	[-0.70; 0.30]	1.2%	1.4%
Persson 2012	175	39.4 (1.8)	386	39.6 (1.9)	-0.10	[-0.50; 0.20]	2.4%	2.7%
Adu-Afarwuah 2015	189	39.4 (1.6)	181	39.2 (1.8)	0.20	[-0.10; 0.60]	2.4%	2.7%
Osrin 2005	75	39.7 (1.8)	81	39.5 (2.1)	0.20	[-0.40; 0.80]	0.8%	1.0%
West 2014	2290	38.90 (2.96)	2283	38.67 (2.99)	0.23	[0.05; 0.42]	8.7%	8.7%
Fawzi 2007	688	39.6 (3)	700	39.3 (3.1)	0.30	[-0.10; 0.60]	2.4%	2.7%
Zagre 2007	246	36.9 (4.5)	119	36.4 (5.5)	0.50	[-0.88; 1.88]	0.2%	0.2%
Christian 2003	227	39.4 (3)	151	38.8 (3.5)	0.59	[-0.06; 1.24]	0.7%	0.8%
Bhutta 2009	238	36.5 (3.9)	285	35.6 (4.2)	0.91	[-0.25; 2.08]	0.2%	0.3%
Fixed Effect Model					0.13	[0.02; 0.25]	21.1%	--
Random Effects Model					0.10	[-0.07; 0.26]	--	23.3%
Heterogeneity: $I^2 = 28\%$, $\tau^2 = 0.0216$, $p = 0.16$								
≥90% adherence								
Sunawang 2009	283	38 (3.4)	244	38.3 (3.1)	-0.37	[-1.30; 0.56]	0.3%	0.4%
Friis 2004	130	39 (3)	124	39.1 (2.5)	-0.10	[-0.80; 0.60]	0.6%	0.7%
Zagre 2007	1161	36.7 (5.1)	1177	36.7 (5.7)	-0.08	[-1.08; 0.92]	0.3%	0.4%
Christian 2003	406	39 (3.2)	383	39 (3.1)	0.01	[-0.39; 0.41]	1.8%	2.1%
Roberfroid 2008	319	39.5 (2.7)	330	39.4 (2.5)	0.10	[-0.30; 0.50]	1.8%	2.1%
Osrin 2005	453	39.4 (1.9)	447	39.3 (1.8)	0.20	[0.00; 0.40]	7.3%	7.5%
Fawzi 2007	2486	39.7 (3)	2442	39.4 (3)	0.30	[0.10; 0.40]	13.0%	12.0%
West 2014	9468	38.91 (2.91)	9593	38.55 (3.07)	0.34	[0.23; 0.45]	25.9%	19.2%
Ashorn 2015	194	39.4 (2.2)	186	39 (2.7)	0.40	[-0.10; 0.90]	1.2%	1.4%
Persson 2012	254	39.6 (2.7)	574	39.2 (2)	0.40	[0.10; 0.80]	2.4%	2.7%
Bhutta 2009	298	35.9 (4)	352	35.4 (4)	0.46	[-0.73; 1.64]	0.2%	0.3%
Adu-Afarwuah 2015	73	39.6 (1.5)	86	39.1 (2)	0.60	[0.00; 1.10]	1.0%	1.2%
Kaestel 2005	178	39.4 (3.9)	98	38.8 (5.2)	0.70	[-0.40; 1.80]	0.2%	0.3%
Fixed Effect Model					0.29	[0.22; 0.37]	56.1%	--
Random Effects Model					0.29	[0.22; 0.37]	--	50.2%
Heterogeneity: $I^2 = 0\%$, $\tau^2 = 0$, $p = 0.58$								
**Fixed Effect Model**					**0.23**	**[0.18; 0.29]**	**100.0%**	**--**
**Random Effects Model**					**0.22**	**[0.16; 0.28]**	**--**	**100.0%**

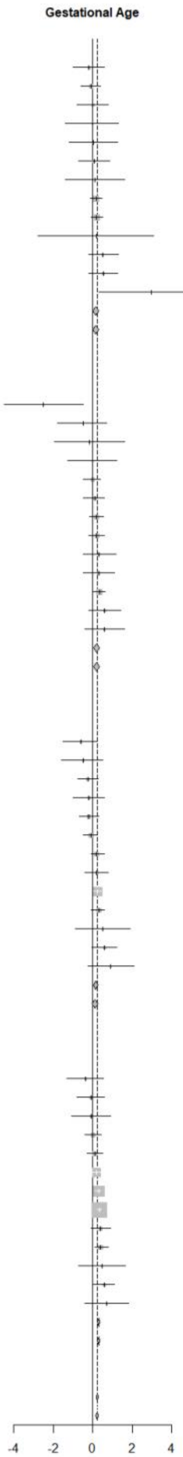

Heterogeneity: $I^2 = 4\%$, $\tau^2 = 0.0019$, $p = 0.39$

Test of subgroup differences (fixed effect): $\chi^2_3 = 6.28$, df = 3 ($p = 0.10$)

Test for subgroup differences (random effects): $\chi^2_3 = 6.23$, df = 3 ($p = 0.10$)

Supplemental Figure 3.5. The effect of MMS vs. IFA on Fetal Death, stratified by Adherence

Study	Events/Total (MMS)	Events/Total (IFA)	RR	95%-CI	Weight (Fixed)	Weight (random)
<60% adherence,						
Bhutta 2009	1/98	2/61	0.31	[0.03; 3.28]	0.0%	0.0%
Kaestel 2005	20/419	16/177	0.53	[0.28; 0.99]	0.6%	0.6%
Zagre 2007	1/13	1/9	0.69	[0.04; 11.19]	0.0%	0.0%
Bliznashka 2022	4/9	11/18	0.73	[0.27; 1.94]	0.3%	0.3%
Sunawang 2009	7/219	8/201	0.80	[0.56; 1.15]	1.9%	1.9%
West 2014						
Moore 2012	0/1	1/1	1.00	[0.20; 4.95]	0.1%	0.1%
Adu-Afarwuah 2015	3/77	2/63	1.23	[0.21; 7.12]	0.1%	0.1%
Fawzi 2007	17/308	11/305	1.53	[0.73; 3.21]	0.4%	0.4%
Ashorn 2015	1/46	0/36	1.59	[0.13; 19.19]	0.0%	0.0%
Christian 2003	8/162	3/164	2.70	[0.74; 9.89]	0.1%	0.1%
Roberfroid 2008	4/69	1/63	3.65	[0.42; 31.81]	0.1%	0.1%
Fixed Effect Model			0.91	[0.81; 1.03]	18.1%	--
Random Effects Model			0.91	[0.81; 1.03]	--	18.1%
Heterogeneity: $I^2 = 0\%$, $\tau^2 = 0$, $p = 0.45$						
60-74% adherence,						
Moore 2012	0/7	2/4	0.13	[0.02; 0.96]	0.1%	0.1%
Ashorn 2015	0/56	3/67	0.20	[0.02; 1.77]	0.1%	0.1%
Christian 2003	3/78	7/74	0.41	[0.11; 1.47]	0.1%	0.1%
Adu-Afarwuah 2015	1/72	2/76	0.53	[0.05; 5.70]	0.0%	0.0%
Fawzi 2007	9/364	16/366	0.57	[0.25; 1.26]	0.4%	0.4%
Bhutta 2009	1/95	2/120	0.63	[0.04; 10.57]	0.0%	0.0%
Zagre 2007	2/40	2/25	0.63	[0.11; 3.47]	0.1%	0.1%
West 2014						
Kaestel 2005	5/258	2/114	1.10	[0.22; 5.61]	0.1%	0.1%
Roberfroid 2008	7/138	4/140	1.78	[0.53; 5.93]	0.2%	0.2%
Bliznashka 2022	5/13	2/10	1.92	[0.48; 7.76]	0.1%	0.1%
Sunawang 2009	5/125	1/137	5.48	[0.61; 48.96]	0.1%	0.1%
Fixed Effect Model			0.93	[0.77; 1.13]	6.8%	--
Random Effects Model			0.84	[0.57; 1.23]	--	6.8%
Heterogeneity: $I^2 = 21\%$, $\tau^2 = 0.0859$, $p = 0.23$						
75-90% adherence						
Moore 2012	0/37	1/28	0.37	[0.03; 4.47]	0.0%	0.0%
Bhutta 2009	1/248	3/303	0.41	[0.04; 3.80]	0.0%	0.0%
Adu-Afarwuah 2015	3/194	5/190	0.59	[0.14; 2.42]	0.1%	0.1%
Fawzi 2007	18/744	30/759	0.61	[0.34; 1.09]	0.7%	0.7%
Roberfroid 2008	6/129	8/122	0.71	[0.25; 1.99]	0.2%	0.2%
Christian 2003	8/238	7/162	0.78	[0.29; 2.10]	0.2%	0.2%
West 2014						
Ashorn 2015	1/133	1/139	1.05	[0.07; 16.54]	0.0%	0.0%
Bliznashka 2022	16/146	16/153	1.05	[0.59; 1.85]	0.7%	0.7%
Sunawang 2009	3/181	3/202	1.12	[0.19; 6.65]	0.1%	0.1%
Zagre 2007	12/258	4/123	1.43	[0.51; 4.01]	0.2%	0.2%
Kaestel 2005	6/206	1/115	3.35	[0.41; 27.48]	0.1%	0.1%
Fixed Effect Model			0.92	[0.80; 1.06]	11.8%	--
Random Effects Model			0.92	[0.80; 1.06]	--	11.8%
Heterogeneity: $I^2 = 0\%$, $\tau^2 = 0$, $p = 0.87$						
≥90% adherence						
Kaestel 2005	6/194	7/114	0.50	[0.17; 1.46]	0.2%	0.2%
Ashorn 2015	3/197	5/193	0.59	[0.14; 2.43]	0.1%	0.1%
Sunawang 2009	8/294	9/263	0.80	[0.33; 1.91]	0.3%	0.3%
Fawzi 2007	78/2638	94/2633	0.83	[0.62; 1.11]	2.9%	2.9%
West 2014						
Roberfroid 2008	15/347	16/365	0.99	[0.50; 1.96]	0.5%	0.5%
Moore 2012	5/169	5/175	1.04	[0.31; 3.51]	0.2%	0.2%
Bliznashka 2022	13/787	13/841	1.07	[0.33; 3.45]	0.2%	0.2%
Zagre 2007	40/1201	36/1213	1.12	[0.68; 1.86]	1.0%	1.0%
Adu-Afarwuah 2015	3/78	3/91	1.17	[0.24; 5.62]	0.1%	0.1%
Bhutta 2009	6/311	5/363	1.40	[0.36; 5.48]	0.1%	0.1%
Christian 2003	28/441	12/402	2.13	[0.98; 4.62]	0.4%	0.4%
Fixed Effect Model			0.98	[0.92; 1.04]	63.3%	--
Random Effects Model			0.98	[0.92; 1.04]	--	63.3%
Heterogeneity: $I^2 = 0\%$, $\tau^2 = 0$, $p = 0.72$						
**Fixed Effect Model**			**0.96**	**[0.91; 1.00]**	**100.0%**	**--**
**Random Effects Model**			**0.96**	**[0.91; 1.00]**	**--**	**100.0%**

Heterogeneity: $I^2 = 0\%$, $\tau^2 = 0$, $p = 0.74$

Test of subgroup differences (fixed effect): $\chi^2_3 = 1.51$, df = 3 ($p = 0.68$)

Test for subgroup differences (random effects): $\chi^2_3 = 1.89$, df = 3 ($p = 0.60$)

Supplemental Figure 3.6. The effect of MMS vs. IFA on Hemoglobin Continuous, stratified by Adherence

Study	Total MMS	MMS Mean (SD)	Total IFA	IFA Mean (SD)	MD	95%-CI	Weight (Fixed)	Weight (random)
<60% adherence,								
Ashorn 2015	30	11 (1.5)	22	11.3 (1.6)	-0.40	[-1.20; 0.50]	0.3%	0.5%
Sunawang 2009	212	10.7 (1.2)	186	10.9 (1.3)	-0.14	[-0.38; 0.10]	3.5%	4.4%
Adu-Afarwuah 2015	50	11.4 (1.2)	35	11.5 (1.2)	-0.10	[-0.60; 0.50]	0.7%	1.2%
Persson 2012	352	11.4 (1.2)	665	11.3 (1.2)	0.00	[-0.10; 0.20]	9.1%	7.3%
Osrin 2005	2	12 (1.6)	4	11.9 (0.8)	0.00	[-2.60; 2.60]	0.0%	0.1%
Christian 2003	23	11.1 (1.7)	11	11 (1.3)	0.07	[-1.08; 1.23]	0.2%	0.3%
West 2014	32	11.32 (0.94)	32	11.07 (0.85)	0.28	[-0.20; 0.76]	0.9%	1.5%
Roberfroid 2008	4	13.8 (3.3)	4	12 (1.5)	1.80	[-2.60; 6.10]	0.0%	0.0%
Fixed Effect Model					-0.03	[-0.15; 0.09]	14.6%	--
Random Effects Model					-0.03	[-0.15; 0.09]	--	15.3%
Heterogeneity: $I^2 = 0\%$, $\tau^2 = 0$, $p = 0.77$								
60-74% adherence,								
Christian 2003	12	10.2 (1.8)	13	11.4 (1.7)	-1.20	[-2.59; 0.19]	0.1%	0.2%
Sunawang 2009	117	10.8 (1.3)	131	11.1 (1.2)	-0.23	[-0.36; -0.09]	11.2%	7.9%
Ashorn 2015	44	11.3 (1.3)	46	11.5 (1.6)	-0.20	[-0.80; 0.40]	0.6%	1.0%
Adu-Afarwuah 2015	57	11.8 (1.2)	64	11.8 (1.3)	0.00	[-0.50; 0.40]	1.0%	1.7%
West 2014	38	11.09 (1.46)	46	10.94 (1.29)	0.08	[-0.57; 0.72]	0.5%	0.9%
Persson 2012	156	11.7 (1.3)	254	11.5 (1.2)	0.20	[-0.10; 0.40]	3.3%	4.2%
Osrin 2005	16	12.1 (1)	7	11.3 (1.3)	0.80	[-0.20; 1.90]	0.2%	0.4%
Roberfroid 2008	55	11.5 (1.8)	43	10.7 (1.4)	0.80	[0.10; 1.50]	0.4%	0.8%
Fixed Effect Model					-0.10	[-0.20; 0.01]	17.2%	--
Random Effects Model					0.05	[-0.23; 0.33]	--	17.0%
Heterogeneity: $I^2 = 67\%$, $\tau^2 = 0.0820$, $p < 0.01$								
75-90% adherence								
Adu-Afarwuah 2015	179	11.6 (1.2)	165	12 (1)	-0.40	[-0.60; -0.20]	5.1%	5.5%
Ashorn 2015	104	10.9 (1.5)	116	11.2 (1.4)	-0.30	[-0.70; 0.10]	1.3%	2.1%
Roberfroid 2008	81	10.7 (1.4)	80	11 (1.7)	-0.30	[-0.80; 0.10]	1.0%	1.7%
Christian 2003	53	11.4 (1.7)	31	11.6 (1.5)	-0.19	[-0.85; 0.48]	0.5%	0.8%
West 2014	119	11.02 (0.95)	137	11.13 (0.98)	-0.13	[-0.38; 0.13]	3.0%	4.0%
Sunawang 2009	172	11.1 (1.2)	194	11.2 (1.2)	-0.08	[-0.24; 0.09]	7.5%	6.7%
Persson 2012	175	11.5 (1.2)	377	11.5 (1.2)	0.00	[-0.20; 0.20]	5.1%	5.5%
Osrin 2005	73	11.9 (1.2)	74	11.8 (1.3)	0.00	[-0.40; 0.40]	1.3%	2.1%
Fixed Effect Model					-0.15	[-0.24; -0.06]	24.7%	--
Random Effects Model					-0.16	[-0.28; -0.04]	--	28.5%
Heterogeneity: $I^2 = 33\%$, $\tau^2 = 0.0091$, $p = 0.17$								
≥90% adherence								
Adu-Afarwuah 2015	75	11.7 (1.1)	84	12 (1.2)	-0.30	[-0.60; 0.10]	1.7%	2.6%
Sunawang 2009	280	11 (1.2)	246	11.2 (1.2)	-0.22	[-0.36; -0.08]	10.4%	7.7%
Ashorn 2015	173	11 (1.4)	159	11.2 (1.5)	-0.20	[-0.50; 0.10]	2.3%	3.3%
Christian 2003	79	11.7 (1.5)	75	11.8 (1.4)	-0.10	[-0.59; 0.39]	0.8%	1.5%
Roberfroid 2008	232	10.8 (1.8)	247	10.9 (1.5)	-0.10	[-0.40; 0.20]	2.3%	3.3%
West 2014	585	11.06 (1.04)	601	11.12 (1.03)	-0.07	[-0.20; 0.06]	11.9%	8.0%
Persson 2012	255	11.6 (1.1)	563	11.6 (1.2)	0.00	[-0.20; 0.10]	9.1%	7.3%
Osrin 2005	442	11.8 (1.3)	429	11.8 (1.4)	0.00	[-0.20; 0.20]	5.1%	5.5%
Fixed Effect Model					-0.10	[-0.17; -0.03]	43.5%	--
Random Effects Model					-0.10	[-0.17; -0.03]	--	39.1%
Heterogeneity: $I^2 = 6\%$, $\tau^2 = 0.0007$, $p = 0.39$								
**Fixed Effect Model**					**-0.10**	**[-0.15; -0.06]**	**100.0%**	**--**
**Random Effects Model**					**-0.10**	**[-0.16; -0.03]**	**--**	**100.0%**

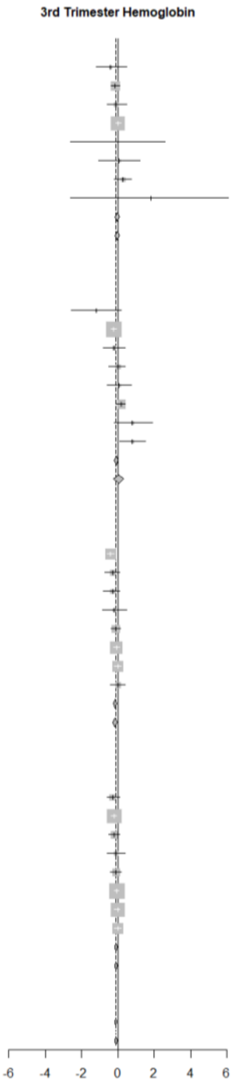

Heterogeneity: $I^2 = 33\%$, $\tau^2 = 0.0086$, $p = 0.04$
Test of subgroup differences (fixed effect): $\chi^2_3 = 2.80$, df = 3 ($p = 0.42$)
Test for subgroup differences (random effects): $\chi^2_3 = 3.46$, df = 3 ($p = 0.33$)

Supplemental Figure 3.7. The effect of MMS vs. IFA on IDA, stratified by Adherence

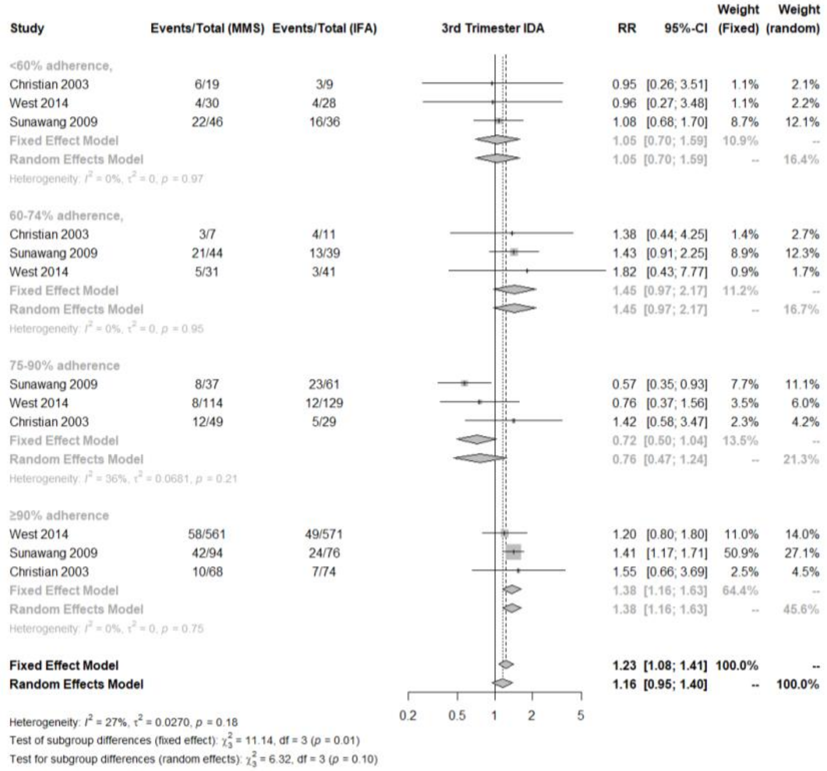

Supplemental Figure 3.8. The effect of MMS vs. IFA on Infant Mortality, stratified by Adherence

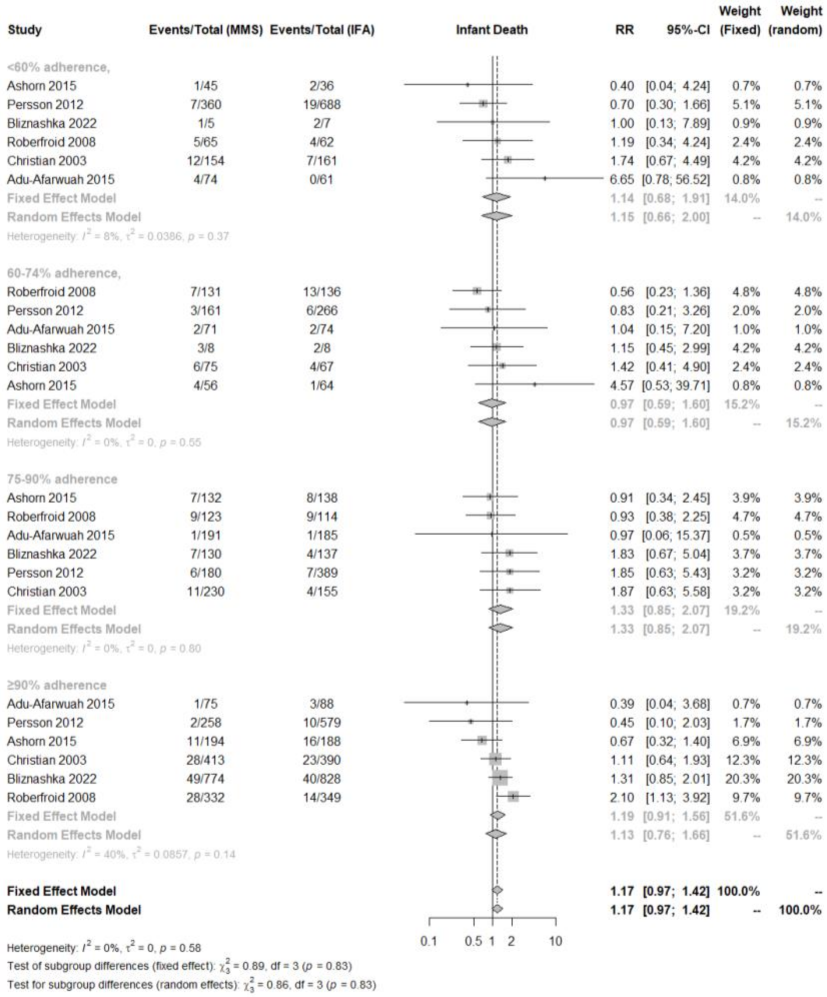

Supplemental Figure 3.9. The effect of MMS vs. IFA on LGA90, stratified by Adherence

Study	Events/Total (MMS)	Events/Total (IFA)	RR	95%-CI	Weight (Fixed)	Weight (random)
**<60% adherence,**						
Persson 2012	1/355	12/675	0.16	[0.02; 1.21]	0.1%	0.1%
Ashorn 2015	0/33	2/26	0.19	[0.02; 1.86]	0.1%	0.1%
Christian 2003	7/150	14/150	0.51	[0.20; 1.27]	0.5%	0.5%
West 2014	21/551	33/463	0.53	[0.32; 0.88]	1.5%	1.5%
Zagre 2007	4/12	5/8	0.60	[0.24; 1.49]	0.5%	0.5%
Roberfroid 2008	3/38	4/38	0.75	[0.18; 3.13]	0.2%	0.2%
Adu-Afarwuah 2015	3/63	3/48	0.76	[0.16; 3.61]	0.2%	0.2%
Sunawang 2009	41/206	43/190	0.88	[0.61; 1.27]	2.9%	2.9%
Kaestel 2005	30/216	15/99	0.92	[0.52; 1.62]	1.2%	1.2%
Fawzi 2007	49/274	48/269	1.00	[0.70; 1.44]	3.0%	3.0%
Bhutta 2009	25/79	14/45	1.07	[0.72; 1.61]	2.4%	2.4%
Friis 2004	2/52	0/31	2.42	[0.25; 23.61]	0.1%	0.1%
Fixed Effect Model			0.84	[0.71; 1.01]	12.5%	--
Random Effects Model			0.83	[0.68; 1.01]	--	12.5%
Heterogeneity: $I^2 = 11\%$, $\tau^2 = 0.0127$, $p = 0.34$						
**60-74% adherence,**						
Ashorn 2015	0/50	1/55	0.54	[0.04; 6.64]	0.1%	0.1%
Kaestel 2005	18/175	13/79	0.63	[0.32; 1.21]	0.9%	0.9%
Adu-Afarwuah 2015	2/63	3/69	0.73	[0.13; 4.23]	0.1%	0.1%
Friis 2004	3/33	5/40	0.73	[0.19; 2.82]	0.2%	0.2%
Persson 2012	2/156	4/261	0.84	[0.16; 4.51]	0.1%	0.1%
Sunawang 2009	25/117	27/132	1.03	[0.73; 1.46]	3.2%	3.2%
West 2014	24/461	20/403	1.06	[0.58; 1.91]	1.1%	1.1%
Bhutta 2009	37/85	40/105	1.07	[0.75; 1.51]	3.2%	3.2%
Christian 2003	7/70	5/66	1.27	[0.44; 3.65]	0.3%	0.3%
Fawzi 2007	60/345	44/326	1.29	[0.90; 1.84]	3.1%	3.1%
Zagre 2007	16/36	6/22	1.63	[0.87; 3.07]	1.0%	1.0%
Roberfroid 2008	11/99	5/96	2.13	[0.77; 5.91]	0.4%	0.4%
Fixed Effect Model			1.11	[0.94; 1.31]	13.7%	--
Random Effects Model			1.11	[0.94; 1.31]	--	13.7%
Heterogeneity: $I^2 = 0\%$, $\tau^2 = 0$, $p = 0.73$						
**75-90% adherence**						
Adu-Afarwuah 2015	4/185	8/180	0.49	[0.15; 1.59]	0.3%	0.3%
Persson 2012	1/175	3/386	0.74	[0.08; 7.02]	0.1%	0.1%
Zagre 2007	93/240	54/114	0.82	[0.61; 1.09]	4.6%	4.6%
Bhutta 2009	88/229	115/283	0.95	[0.70; 1.28]	4.3%	4.3%
Fawzi 2007	99/661	100/684	1.02	[0.79; 1.32]	5.9%	5.9%
Sunawang 2009	37/172	39/191	1.05	[0.65; 1.69]	1.7%	1.7%
West 2014	78/1600	73/1637	1.09	[0.79; 1.50]	3.8%	3.8%
Roberfroid 2008	5/96	4/93	1.21	[0.34; 4.37]	0.2%	0.2%
Christian 2003	20/227	11/150	1.22	[0.59; 2.49]	0.8%	0.8%
Kaestel 2005	28/153	10/85	1.56	[0.79; 3.04]	0.9%	0.9%
Friis 2004	6/44	3/43	1.95	[0.52; 7.32]	0.2%	0.2%
Ashorn 2015	5/114	1/131	5.75	[0.68; 48.46]	0.1%	0.1%
Fixed Effect Model			1.00	[0.88; 1.14]	22.9%	--
Random Effects Model			1.00	[0.88; 1.14]	--	22.9%
Heterogeneity: $I^2 = 0\%$, $\tau^2 = 0$, $p = 0.59$						
**≥90% adherence**						
Persson 2012	2/254	9/574	0.50	[0.11; 2.31]	0.2%	0.2%
Ashorn 2015	4/180	7/169	0.54	[0.16; 1.80]	0.3%	0.3%
Adu-Afarwuah 2015	0/72	1/83	0.57	[0.05; 7.05]	0.1%	0.1%
Friis 2004	7/111	7/97	0.87	[0.32; 2.40]	0.4%	0.4%
Bhutta 2009	114/290	150/348	0.92	[0.71; 1.18]	6.0%	6.0%
West 2014	356/7547	386/7592	0.94	[0.80; 1.10]	15.4%	15.4%
Kaestel 2005	18/127	10/70	0.99	[0.48; 2.03]	0.8%	0.8%
Fawzi 2007	297/2407	288/2374	1.02	[0.87; 1.18]	16.8%	16.8%
Christian 2003	27/406	22/381	1.15	[0.69; 1.91]	1.5%	1.5%
Zagre 2007	456/1139	395/1145	1.16	[0.92; 1.45]	7.5%	7.5%
Roberfroid 2008	11/272	9/277	1.24	[0.52; 2.96]	0.5%	0.5%
Sunawang 2009	69/283	44/244	1.35	[0.81; 2.27]	1.5%	1.5%
Fixed Effect Model			1.01	[0.92; 1.10]	50.9%	--
Random Effects Model			1.01	[0.92; 1.10]	--	50.9%
Heterogeneity: $I^2 = 0\%$, $\tau^2 = 0$, $p = 0.83$						
**Fixed Effect Model**			**1.00**	**[0.94; 1.06]**	**100.0%**	**--**
**Random Effects Model**			**1.00**	**[0.94; 1.06]**	**--**	**100.0%**

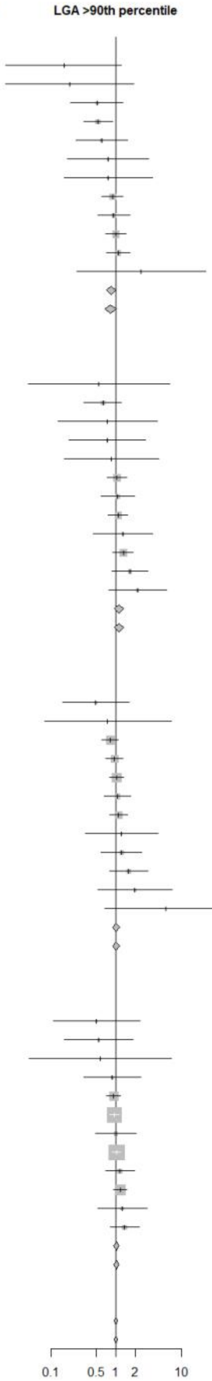

Heterogeneity: $I^2 = 0\%$, $\tau^2 = 0$, $p = 0.72$

Test of subgroup differences (fixed effect): $\chi^2_3 = 5.06$, df = 3 ($p = 0.17$)

Test for subgroup differences (random effects): $\chi^2_3 = 4.98$, df = 3 ($p = 0.17$)

Supplemental Figure 3.10. The effect of MMS vs. IFA on Low Birthweight, stratified by Adherence

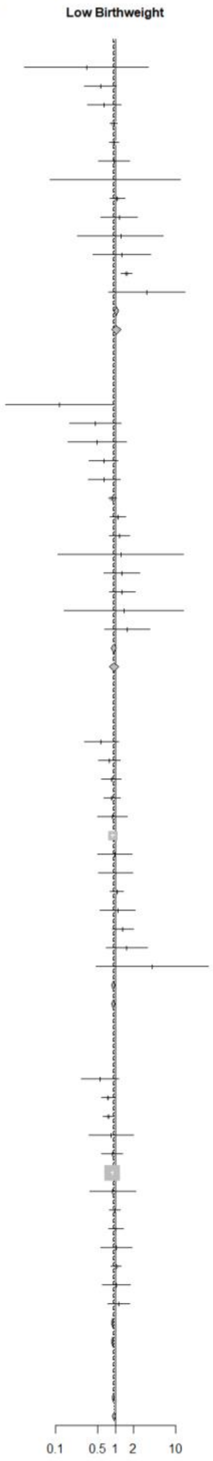

Study	Events/Total (MMS)	Events/Total (IFA)	RR	95%-CI	Weight (Fixed)	Weight (random)
<60% adherence,						
Zagre 2007	1/12	2/8	0.33	[0.03; 3.43]	0.0%	0.1%
Bhutta 2009	11/81	11/47	0.56	[0.30; 1.02]	0.2%	0.8%
Kaestel 2005	20/231	14/104	0.64	[0.34; 1.22]	0.2%	0.7%
West 2014	234/574	214/486	0.93	[0.81; 1.06]	4.3%	6.6%
Persson 2012	117/360	238/688	0.94	[0.78; 1.13]	2.4%	5.1%
Fawzi 2007	20/274	21/269	0.94	[0.52; 1.68]	0.2%	0.9%
Osrin 2005	1/5	1/5	1.00	[0.08; 11.93]	0.0%	0.1%
Christian 2003	66/150	64/151	1.06	[0.80; 1.40]	1.1%	3.0%
Adu-Afarwuah 2015	15/63	10/48	1.14	[0.56; 2.32]	0.2%	0.6%
Friis 2004	4/53	2/32	1.21	[0.23; 6.22]	0.0%	0.1%
Roberfroid 2008	6/40	5/42	1.26	[0.42; 3.80]	0.1%	0.3%
Sunawang 2009	18/206	11/190	1.51	[1.23; 1.85]	2.0%	4.5%
Ashorn 2015	8/33	2/27	3.27	[0.76; 14.14]	0.0%	0.1%
Fixed Effect Model			1.02	[0.94; 1.12]	10.8%	--
Random Effects Model			1.01	[0.84; 1.21]	--	22.8%
Heterogeneity: $I^2 = 54\%$, $\tau^2 = 0.0385$, $p < 0.01$						
60-74% adherence,						
Friis 2004	0/34	5/40	0.12	[0.01; 0.93]	0.0%	0.1%
Adu-Afarwuah 2015	5/63	12/69	0.46	[0.17; 1.22]	0.1%	0.3%
Ashorn 2015	4/50	9/55	0.49	[0.16; 1.49]	0.1%	0.3%
Roberfroid 2008	16/104	25/102	0.63	[0.36; 1.10]	0.3%	0.9%
Fawzi 2007	17/345	25/326	0.64	[0.35; 1.17]	0.2%	0.8%
West 2014	199/482	196/421	0.88	[0.77; 1.01]	4.3%	6.6%
Persson 2012	52/161	79/266	1.09	[0.81; 1.45]	1.0%	2.8%
Christian 2003	33/70	28/66	1.16	[0.78; 1.72]	0.5%	1.7%
Zagre 2007	2/38	1/23	1.21	[0.11; 13.43]	0.0%	0.1%
Sunawang 2009	7/117	7/132	1.26	[0.64; 2.49]	0.2%	0.6%
Bhutta 2009	14/85	16/108	1.26	[0.77; 2.07]	0.3%	1.2%
Osrin 2005	2/16	1/11	1.38	[0.14; 13.37]	0.0%	0.1%
Kaestel 2005	20/185	6/86	1.55	[0.65; 3.72]	0.1%	0.4%
Fixed Effect Model			0.92	[0.83; 1.03]	7.1%	--
Random Effects Model			0.93	[0.78; 1.12]	--	15.9%
Heterogeneity: $I^2 = 29\%$, $\tau^2 = 0.0248$, $p = 0.15$						
75-90% adherence						
Adu-Afarwuah 2015	13/185	22/180	0.57	[0.30; 1.11]	0.2%	0.7%
Bhutta 2009	40/232	64/287	0.78	[0.51; 1.19]	0.5%	1.5%
Fawzi 2007	45/661	55/684	0.85	[0.58; 1.24]	0.6%	1.8%
Christian 2003	66/227	52/150	0.86	[0.63; 1.17]	0.9%	2.6%
Osrin 2005	17/75	21/81	0.87	[0.50; 1.53]	0.3%	0.9%
West 2014	723/1685	818/1701	0.89	[0.83; 0.96]	15.1%	9.3%
Roberfroid 2008	15/97	15/93	0.96	[0.50; 1.85]	0.2%	0.7%
Kaestel 2005	22/162	12/88	1.00	[0.52; 1.91]	0.2%	0.7%
Persson 2012	57/180	119/389	1.04	[0.80; 1.34]	1.2%	3.3%
Ashorn 2015	14/114	15/131	1.07	[0.54; 2.12]	0.2%	0.6%
Sunawang 2009	13/173	11/191	1.30	[0.87; 1.95]	0.5%	1.7%
Zagre 2007	22/246	7/119	1.52	[0.69; 3.33]	0.1%	0.5%
Friis 2004	4/44	1/44	4.00	[0.47; 34.38]	0.0%	0.1%
Fixed Effect Model			0.91	[0.85; 0.97]	20.0%	--
Random Effects Model			0.91	[0.85; 0.97]	--	24.5%
Heterogeneity: $I^2 = 0\%$, $\tau^2 = 0$, $p = 0.54$						
≥90% adherence						
Kaestel 2005	13/137	13/76	0.55	[0.27; 1.13]	0.2%	0.6%
Osrin 2005	86/453	114/447	0.74	[0.58; 0.95]	1.4%	3.6%
Fawzi 2007	135/2407	177/2374	0.75	[0.61; 0.93]	1.9%	4.4%
Adu-Afarwuah 2015	8/72	11/83	0.84	[0.36; 1.97]	0.1%	0.4%
Zagre 2007	90/1161	105/1177	0.87	[0.58; 1.30]	0.5%	1.7%
West 2014	3117/7898	3579/7918	0.87	[0.84; 0.91]	52.0%	10.5%
Friis 2004	9/115	9/102	0.89	[0.37; 2.15]	0.1%	0.4%
Persson 2012	79/258	186/579	0.95	[0.77; 1.19]	1.8%	4.2%
Bhutta 2009	55/291	66/349	0.99	[0.75; 1.32]	1.0%	2.9%
Sunawang 2009	19/283	16/244	1.01	[0.56; 1.85]	0.2%	0.8%
Christian 2003	129/407	122/384	1.01	[0.83; 1.22]	2.2%	4.8%
Ashorn 2015	25/180	23/169	1.02	[0.60; 1.73]	0.3%	1.0%
Roberfroid 2008	39/273	36/279	1.11	[0.73; 1.69]	0.5%	1.6%
Fixed Effect Model			0.88	[0.84; 0.91]	62.1%	--
Random Effects Model			0.88	[0.84; 0.91]	--	36.9%
Heterogeneity: $I^2 = 0\%$, $\tau^2 = 0$, $p = 0.56$						
**Fixed Effect Model**			**0.90**	**[0.87; 0.93]**	**100.0%**	**--**
**Random Effects Model**			**0.94**	**[0.88; 0.99]**	**--**	**100.0%**

Heterogeneity: $I^2 = 32\%$, $\tau^2 = 0.0075$, $p = 0.02$
Test of subgroup differences (fixed effect): $\chi^2_3 = 10.47$, df = 3 ($p = 0.01$)
Test for subgroup differences (random effects): $\chi^2_3 = 3.22$, df = 3 ($p = 0.36$)

Supplemental Figure 3.11. The effect of MMS vs. IFA on Neonatal Mortality, stratified by Adherence

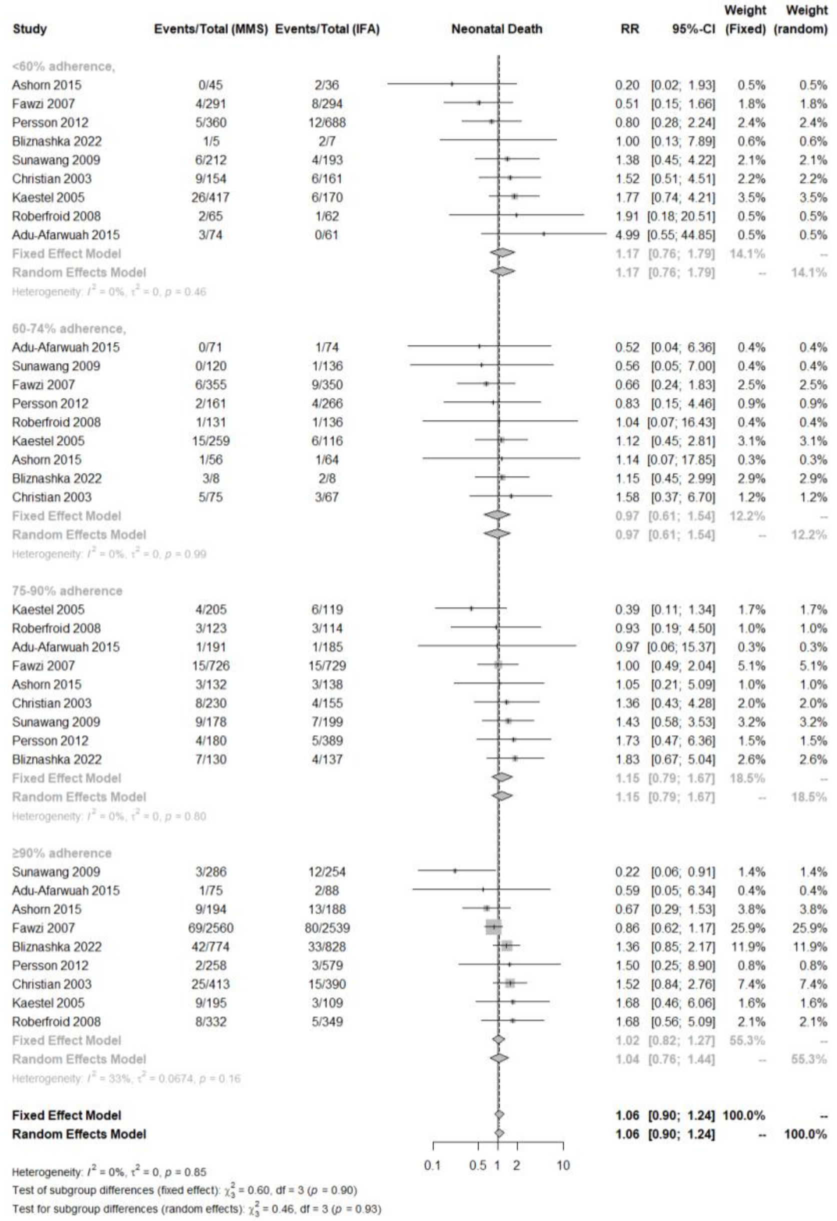

Supplemental Figure 3.12. The effect of MMS vs. IFA on Preterm, stratified by Adherence

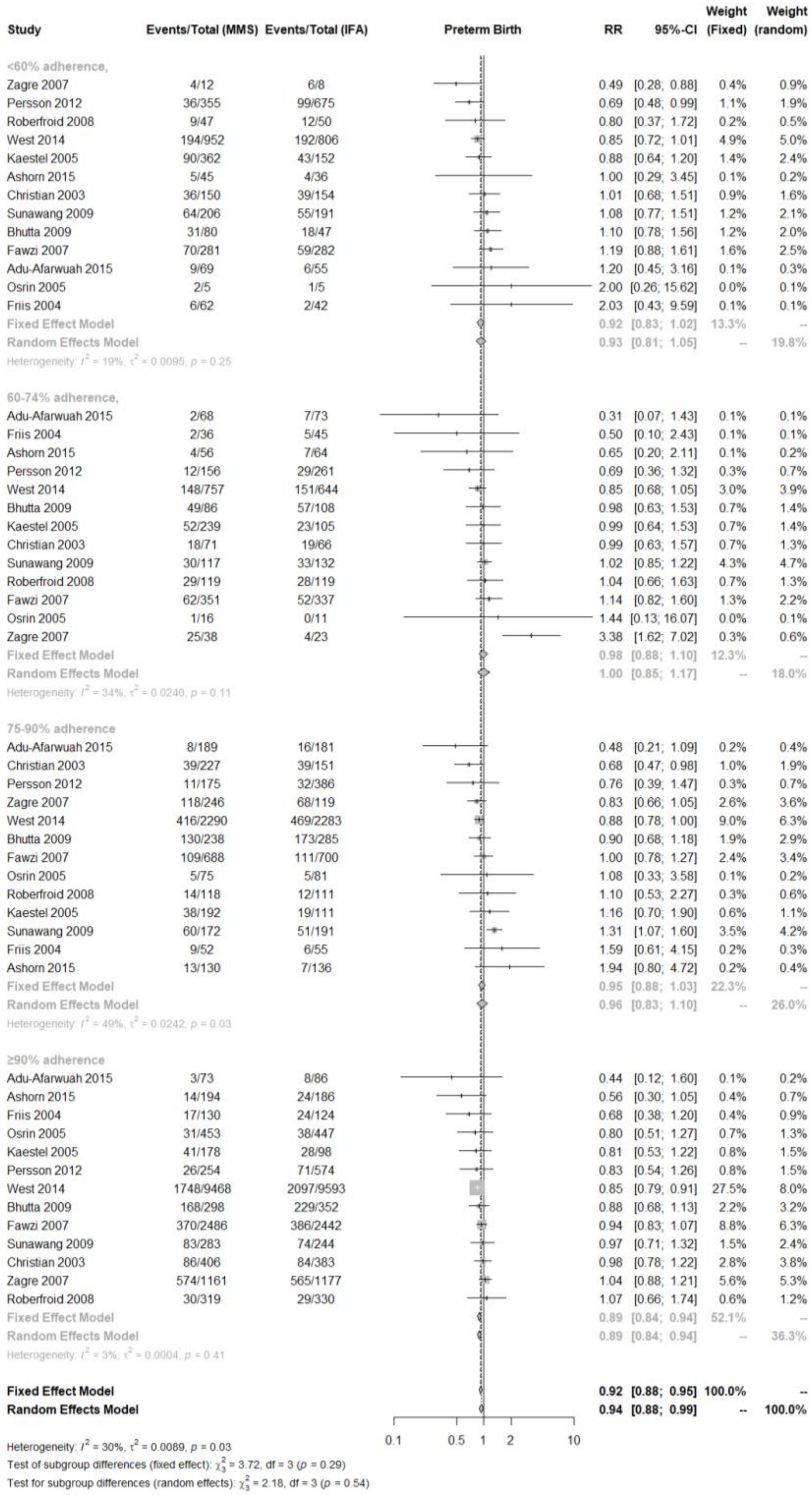

Supplemental Figure 3.13. The effect of MMS vs. IFA on SGA10, stratified by Adherence

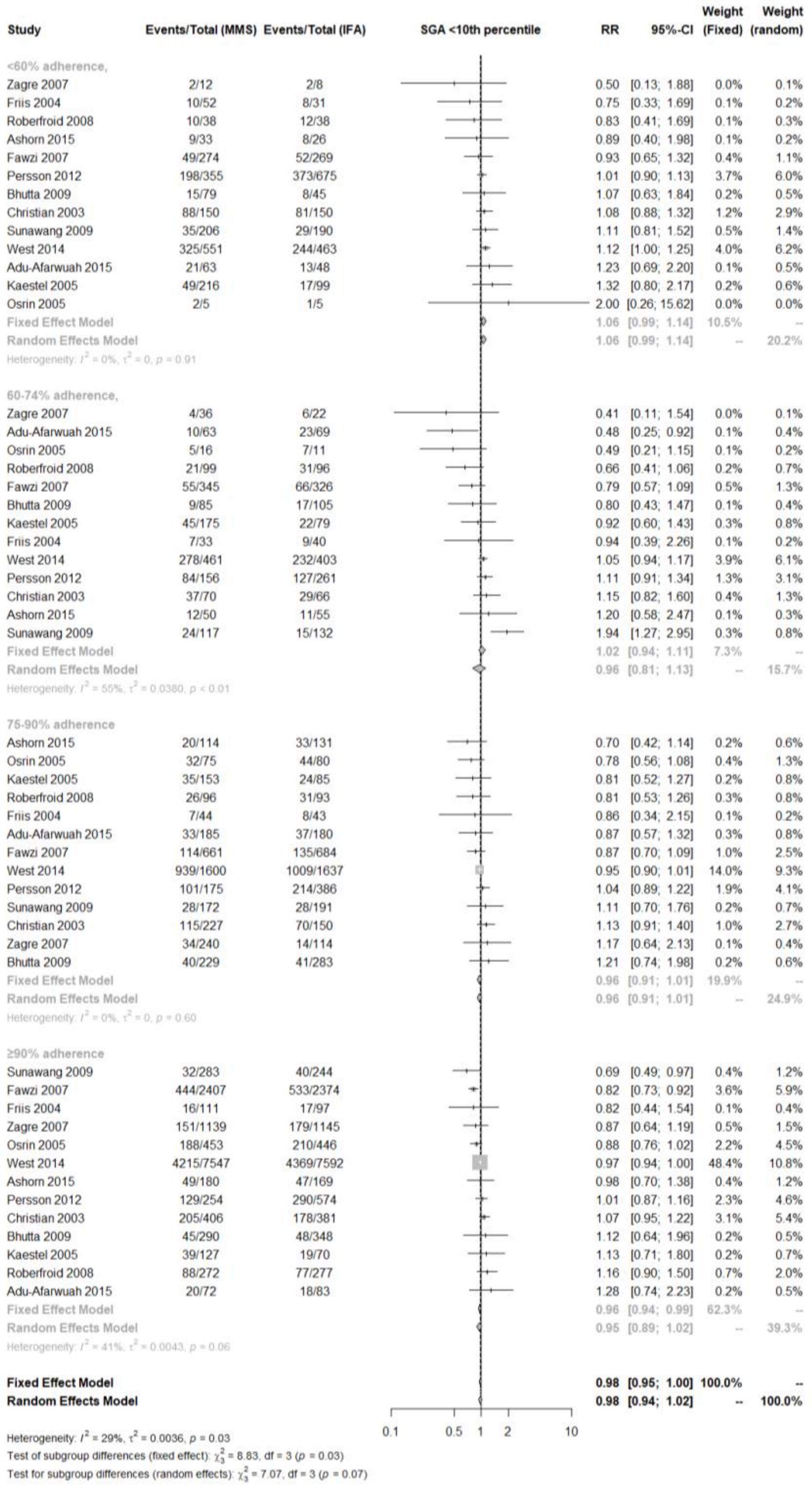

Supplemental Figure 3.14. The effect of MMS vs. IFA on Stillbirth, stratified by Adherence

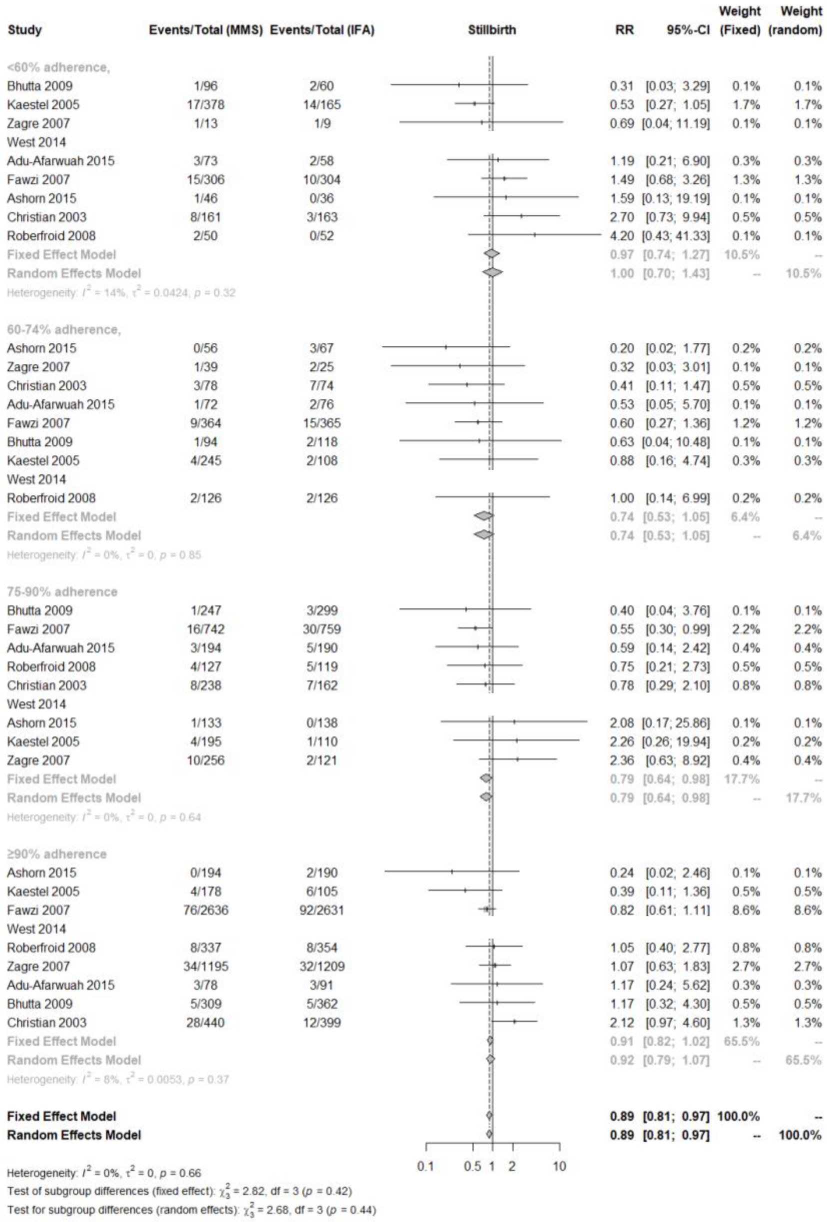

Objective 1 Sensivity analysis by MMS iron dose stratified by Adherence

Supplemental Figure 3.15. The effect of MMS vs. IFA on hemoglobin, in trials with lower iron dose in MMS than IFA, stratified by adherence

Study	Total MMS	MMS Mean (SD)	Total IFA	IFA Mean (SD)	MD	95%-CI	Weight (Fixed)	Weight (random)
**Lower Dose Iron: <60% adherence,**								
Sunawang 2009	212	10.7 (1.2)	186	10.9 (1.3)	-0.14	[-0.38; 0.10]	5.6%	6.7%
Adu-Afarwuah 2015	50	11.4 (1.2)	35	11.5 (1.2)	-0.10	[-0.60; 0.50]	1.1%	2.1%
Osrin 2005	2	12 (1.6)	4	11.9 (0.8)	0.00	[-2.60; 2.60]	0.0%	0.1%
Persson 2012	166	11.3 (1.2)	332	11.3 (1.2)	0.00	[-0.20; 0.20]	8.0%	8.1%
Roberfroid 2008	4	13.8 (3.3)	4	12 (1.5)	1.80	[-2.60; 6.10]	0.0%	0.0%
Fixed Effect Model					-0.06	[-0.21; 0.09]	14.7%	--
Random Effects Model					-0.06	[-0.21; 0.09]	--	17.0%
Heterogeneity: $I^2 = 0\%$, $\tau^2 = 0$, $p = 0.83$								
**Lower Dose Iron: 60-74% adherence,**								
Sunawang 2009	117	10.8 (1.3)	131	11.1 (1.2)	-0.23	[-0.36; -0.09]	17.6%	10.6%
Adu-Afarwuah 2015	57	11.8 (1.2)	64	11.8 (1.3)	0.00	[-0.50; 0.40]	1.6%	2.9%
Persson 2012	82	11.7 (1.3)	143	11.5 (1.2)	0.20	[-0.10; 0.50]	3.6%	5.2%
Osrin 2005	16	12.1 (1)	7	11.3 (1.3)	0.80	[-0.20; 1.90]	0.3%	0.6%
Roberfroid 2008	55	11.5 (1.8)	43	10.7 (1.4)	0.80	[0.10; 1.50]	0.7%	1.4%
Fixed Effect Model					-0.11	[-0.23; 0.01]	23.7%	--
Random Effects Model					0.17	[-0.19; 0.53]	--	20.7%
Heterogeneity: $I^2 = 76\%$, $\tau^2 = 0.1069$, $p < 0.01$								
**Lower Dose Iron: 75-90% adherence**								
Adu-Afarwuah 2015	179	11.6 (1.2)	165	12 (1)	-0.40	[-0.60; -0.20]	8.0%	8.1%
Roberfroid 2008	81	10.7 (1.4)	80	11 (1.7)	-0.30	[-0.80; 0.10]	1.6%	2.9%
Sunawang 2009	172	11.1 (1.2)	194	11.2 (1.2)	-0.08	[-0.24; 0.09]	11.8%	9.4%
Osrin 2005	73	11.9 (1.2)	74	11.8 (1.3)	0.00	[-0.40; 0.40]	2.0%	3.5%
Persson 2012	84	11.5 (1.3)	193	11.5 (1.2)	0.00	[-0.40; 0.30]	2.6%	4.2%
Fixed Effect Model					-0.18	[-0.29; -0.07]	26.0%	--
Random Effects Model					-0.17	[-0.35; 0.01]	--	28.0%
Heterogeneity: $I^2 = 51\%$, $\tau^2 = 0.0195$, $p = 0.09$								
**Lower Dose Iron: ≥90% adherence**								
Adu-Afarwuah 2015	75	11.7 (1.1)	84	12 (1.2)	-0.30	[-0.60; 0.10]	2.6%	4.2%
Sunawang 2009	280	11 (1.2)	246	11.2 (1.2)	-0.22	[-0.36; -0.08]	16.3%	10.4%
Roberfroid 2008	232	10.8 (1.8)	247	10.9 (1.5)	-0.10	[-0.40; 0.20]	3.6%	5.2%
Osrin 2005	442	11.8 (1.3)	429	11.8 (1.4)	0.00	[-0.20; 0.20]	8.0%	8.1%
Persson 2012	143	11.7 (1.2)	279	11.6 (1.2)	0.00	[-0.20; 0.30]	5.1%	6.5%
Fixed Effect Model					-0.13	[-0.23; -0.04]	35.7%	--
Random Effects Model					-0.12	[-0.24; -0.01]	--	34.3%
Heterogeneity: $I^2 = 23\%$, $\tau^2 = 0.0040$, $p = 0.27$								
**Fixed Effect Model**					**-0.13**	**[-0.18; -0.07]**	**100.0%**	**--**
**Random Effects Model**					**-0.10**	**[-0.18; -0.01]**	**--**	**100.0%**

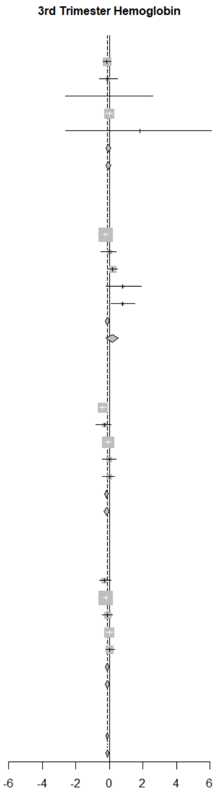

Heterogeneity: $I^2 = 43\%$, $\tau^2 = 0.0133$, $p = 0.02$
Test of subgroup differences (fixed effect): $\chi^2_3 = 1.75$, df = 3 ($p = 0.63$)
Test for subgroup differences (random effects): $\chi^2_3 = 3.16$, df = 3 ($p = 0.37$)

Supplemental Figure 3.16. The effect of MMS vs. IFA on hemoglobin, in trials with same iron dose in MMS than IFA, stratified by adherence

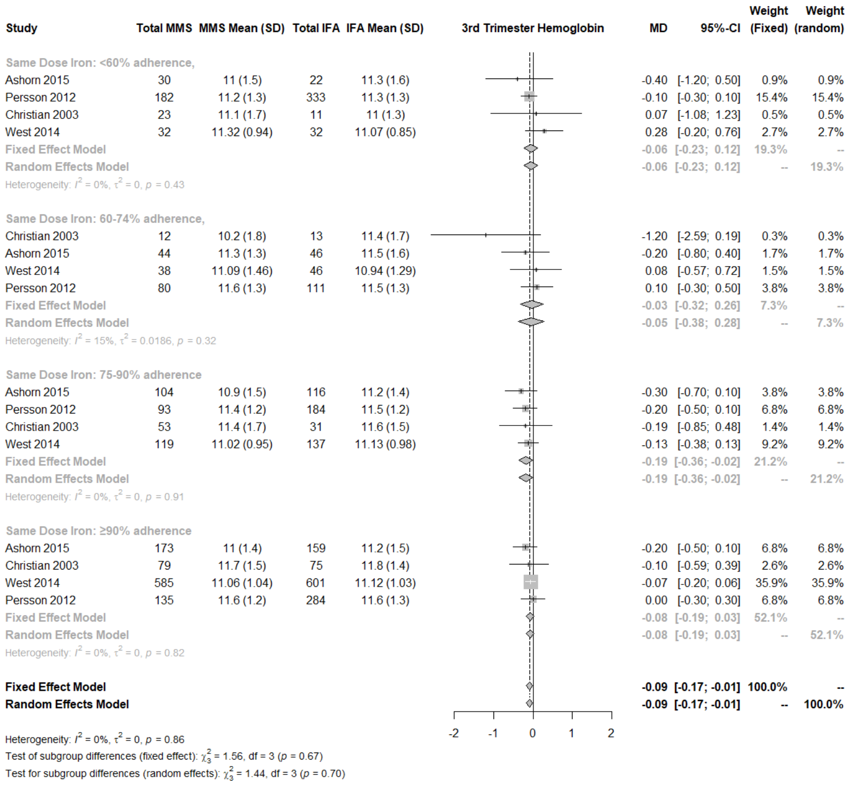

Supplemental Figure 3.17. The effect of MMS vs. IFA on anemia, in trials with lower iron dose in MMS than IFA, stratified by adherence

Study	Events/Total (MMS)	Events/Total (IFA)	RR	95%-CI	Weight (Fixed)	Weight (random)
**Lower Dose Iron: <60% adherence,**						
Roberfroid 2008	1/4	1/4	1.00	[0.09; 11.03]	0.0%	0.1%
Persson 2012	66/166	122/332	1.08	[0.86; 1.37]	4.7%	6.6%
Sunawang 2009	124/212	100/186	1.09	[0.93; 1.27]	10.4%	11.0%
Adu-Afarwuah 2015	15/50	7/35	1.50	[0.68; 3.29]	0.4%	0.8%
Osrin 2005	1/2	0/4	4.50	[0.67; 30.23]	0.1%	0.1%
Fixed Effect Model			1.10	[0.97; 1.25]	15.6%	--
Random Effects Model			1.10	[0.97; 1.25]	--	18.6%
Heterogeneity: $I^2 = 0\%$, $\tau^2 = 0$, $p = 0.60$						
**Lower Dose Iron: 60-74% adherence,**						
Osrin 2005	3/16	3/7	0.44	[0.12; 1.66]	0.1%	0.3%
Roberfroid 2008	22/55	26/43	0.66	[0.44; 0.99]	1.5%	2.7%
Persson 2012	26/82	49/143	0.93	[0.63; 1.37]	1.7%	2.9%
Adu-Afarwuah 2015	13/57	14/64	1.04	[0.54; 2.03]	0.6%	1.1%
Sunawang 2009	66/117	61/131	1.21	[1.10; 1.33]	28.0%	16.5%
Fixed Effect Model			1.15	[1.05; 1.26]	31.9%	--
Random Effects Model			0.92	[0.68; 1.26]	--	23.5%
Heterogeneity: $I^2 = 66\%$, $\tau^2 = 0.0693$, $p = 0.02$						
**Lower Dose Iron: 75-90% adherence**						
Osrin 2005	14/73	16/74	0.89	[0.47; 1.68]	0.6%	1.2%
Persson 2012	27/84	61/193	1.02	[0.70; 1.48]	1.8%	3.1%
Sunawang 2009	74/172	81/194	1.03	[0.89; 1.20]	11.3%	11.5%
Roberfroid 2008	41/81	38/80	1.07	[0.78; 1.46]	2.6%	4.2%
Adu-Afarwuah 2015	46/179	23/165	1.84	[1.17; 2.90]	1.2%	2.2%
Fixed Effect Model			1.07	[0.95; 1.21]	17.5%	--
Random Effects Model			1.10	[0.92; 1.32]	--	22.1%
Heterogeneity: $I^2 = 35\%$, $\tau^2 = 0.0145$, $p = 0.19$						
**Lower Dose Iron: ≥90% adherence**						
Osrin 2005	102/442	109/429	0.91	[0.72; 1.15]	4.6%	6.6%
Roberfroid 2008	120/232	129/247	0.99	[0.83; 1.18]	8.2%	9.6%
Persson 2012	40/143	77/279	1.01	[0.73; 1.40]	2.4%	3.9%
Sunawang 2009	125/280	97/246	1.13	[1.01; 1.27]	19.2%	14.5%
Adu-Afarwuah 2015	17/75	15/84	1.27	[0.68; 2.36]	0.7%	1.2%
Fixed Effect Model			1.06	[0.97; 1.15]	35.0%	--
Random Effects Model			1.06	[0.97; 1.15]	--	35.8%
Heterogeneity: $I^2 = 0\%$, $\tau^2 = 0$, $p = 0.43$						
**Fixed Effect Model**			**1.10**	**[1.04; 1.15]**	**100.0%**	**--**
**Random Effects Model**			**1.07**	**[1.00; 1.15]**	**--**	**100.0%**

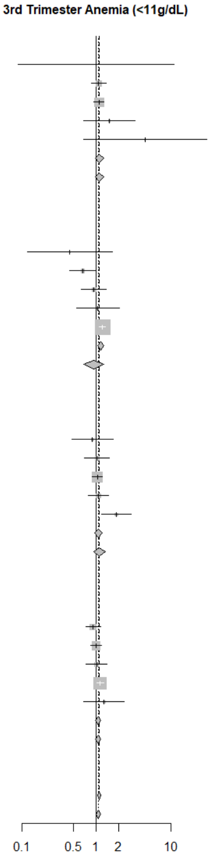

Heterogeneity: $I^2 = 27\%$, $\tau^2 = 0.0056$, $p = 0.13$
Test of subgroup differences (fixed effect): $\chi^2_3 = 1.91$, df = 3 ($p = 0.59$)
Test for subgroup differences (random effects): $\chi^2_3 = 1.22$, df = 3 ($p = 0.75$)

Supplemental Figure 3.18. The effect of MMS vs. IFA on anemia, in trials with same iron dose in MMS than IFA, stratified by adherence

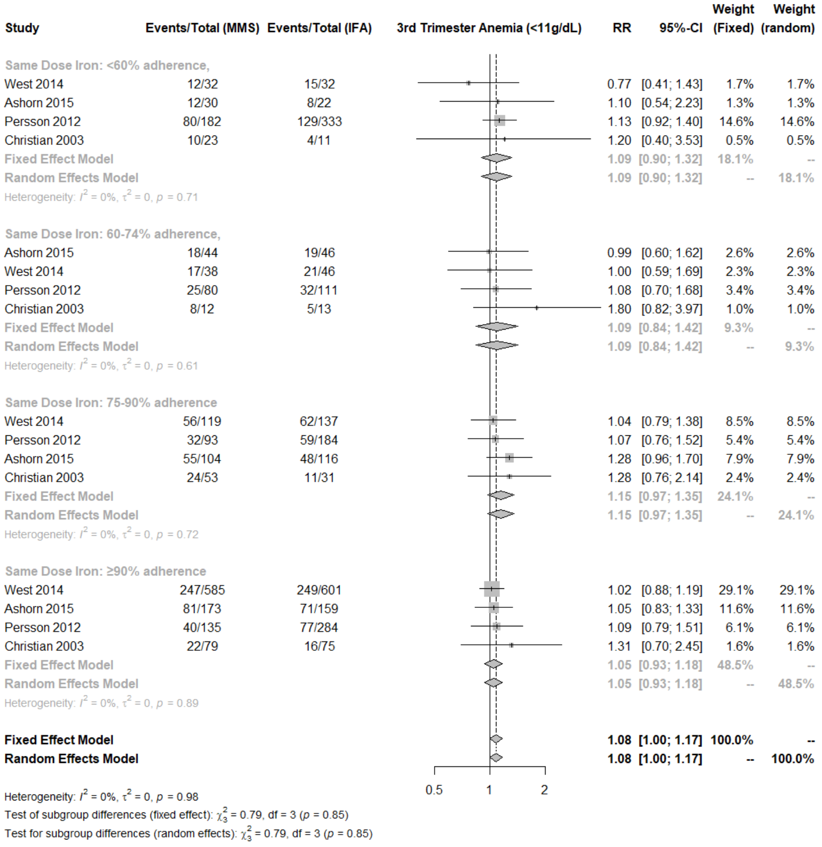

Supplemental Figure 3.19. The effect of MMS vs. IFA on IDA, in trials with lower iron dose in MMS than IFA, stratified by adherence

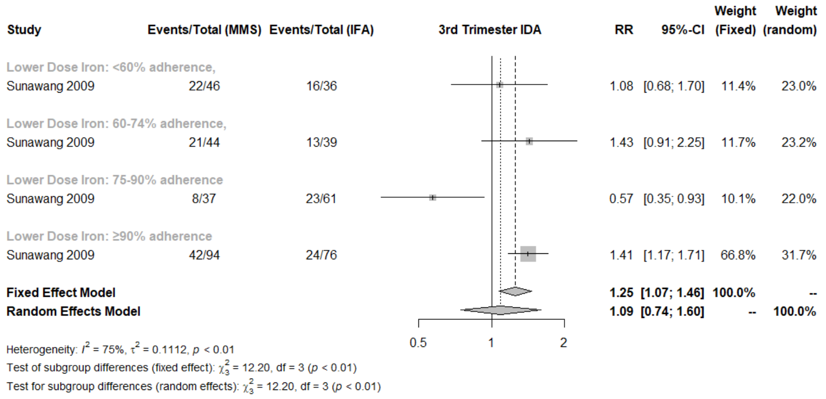

Supplemental Figure 3.20. The effect of MMS vs. IFA on IDA, in trials with same iron dose in MMS than IFA, stratified by adherence

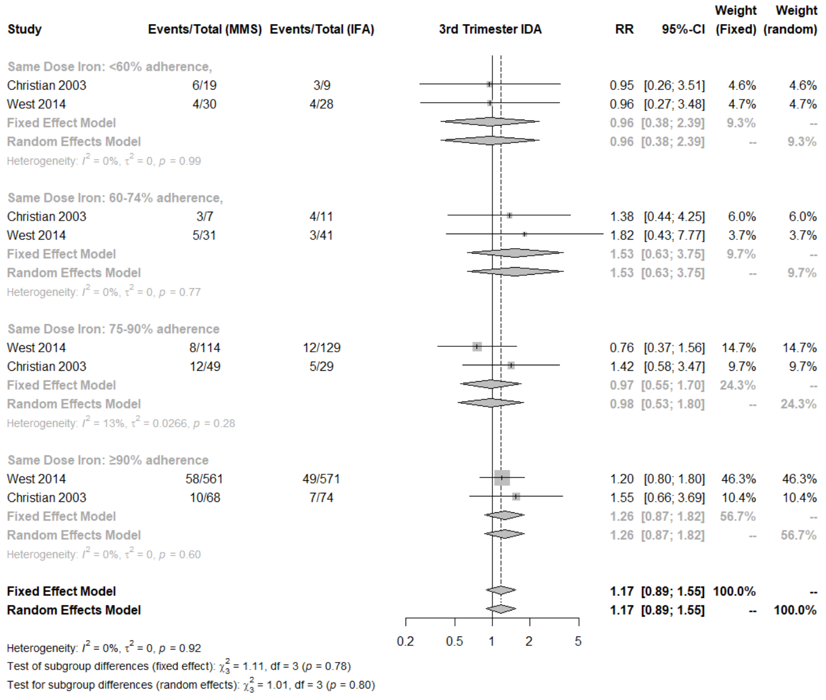

Supplemental Figure 3.21. The effect of MMS vs. IFA on birthweight, in trials with lower iron dose in MMS than IFA, stratified by adherence

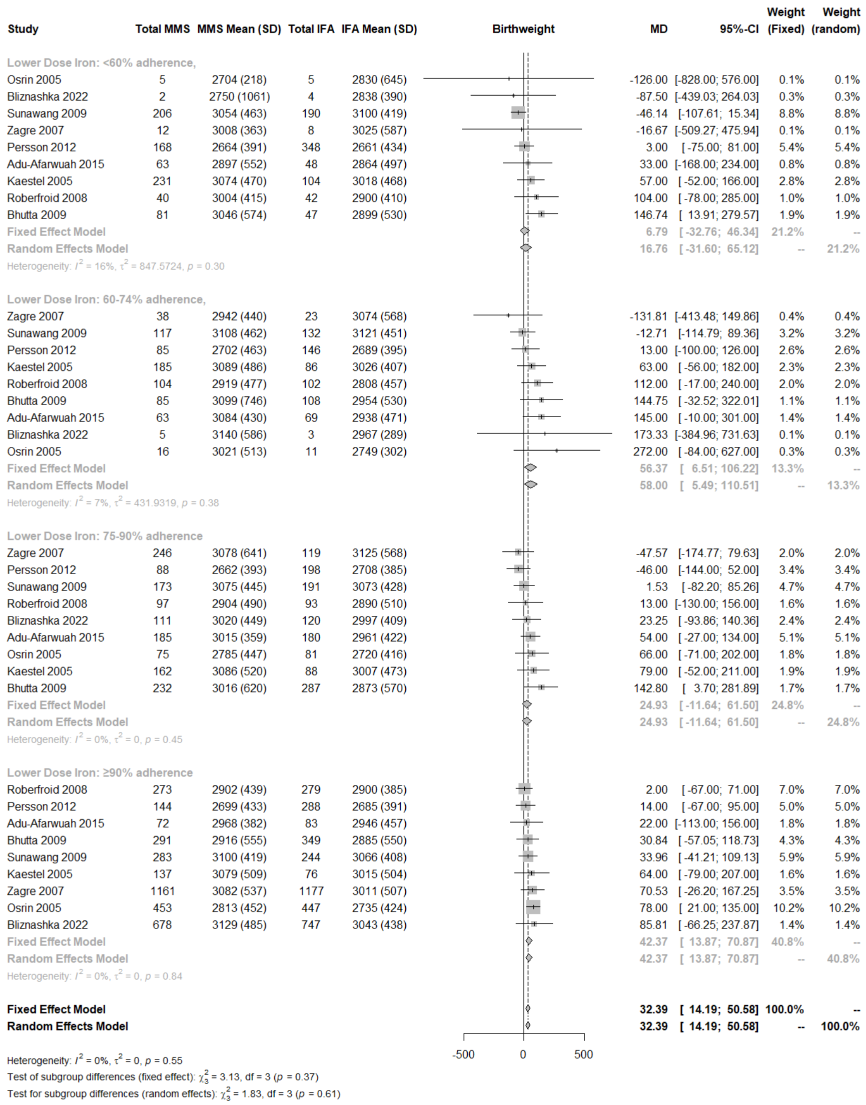

Supplemental Figure 3.22. The effect of MMS vs. IFA on birthweight, in trials with same iron dose in MMS than IFA, stratified by adherence

Study	Total MMS	MMS Mean (SD)	Total IFA	IFA Mean (SD)	MD	95%-CI	Weight (Fixed)	Weight (random)
**Same Dose Iron: <60% adherence,**								
Ashorn 2015	33	2859 (482)	27	2964 (478)	-106.00	[-355.00; 144.00]	0.2%	0.3%
Persson 2012	186	2646 (388)	340	2642 (441)	3.00	[-72.00; 79.00]	1.7%	2.8%
West 2014	574	2568 (395)	486	2565 (424)	3.13	[-46.65; 52.91]	3.9%	6.0%
Friis 2004	53	3104 (477)	32	3089 (385)	15.00	[-183.00; 213.00]	0.2%	0.4%
Christian 2003	150	2564 (493)	151	2544 (458)	20.54	[-101.11; 142.20]	0.7%	1.1%
Fawzi 2007	274	3104 (478)	269	3068 (487)	36.00	[-45.00; 118.00]	1.4%	2.5%
**Fixed Effect Model**					8.68	[-25.85; 43.21]	8.1%	--
**Random Effects Model**					8.68	[-25.85; 43.21]	--	13.2%
Heterogeneity: $I^2 = 0\%$, $\tau^2 = 0$, $p = 0.93$								
**Same Dose Iron: 60-74% adherence,**								
Christian 2003	70	2521 (507)	66	2577 (510)	-56.40	[-227.70; 114.90]	0.3%	0.6%
Persson 2012	83	2676 (449)	120	2689 (426)	-13.00	[-136.00; 109.00]	0.6%	1.1%
West 2014	482	2559 (383)	421	2491 (425)	65.38	[11.16; 119.60]	3.3%	5.2%
Ashorn 2015	50	3021 (450)	55	2943 (422)	78.00	[-91.00; 246.00]	0.3%	0.6%
Fawzi 2007	345	3218 (507)	326	3137 (483)	81.00	[6.00; 156.00]	1.7%	2.9%
Friis 2004	34	3207 (443)	40	3058 (417)	149.00	[-51.00; 349.00]	0.2%	0.4%
**Fixed Effect Model**					59.40	[21.02; 97.78]	6.5%	--
**Random Effects Model**					59.40	[21.02; 97.78]	--	10.8%
Heterogeneity: $I^2 = 0\%$, $\tau^2 = 0$, $p = 0.51$								
**Same Dose Iron: 75-90% adherence**								
Persson 2012	95	2633 (353)	191	2661 (415)	-27.00	[-125.00; 70.00]	1.0%	1.8%
Friis 2004	44	3136 (435)	44	3123 (398)	13.00	[-164.00; 190.00]	0.3%	0.5%
Ashorn 2015	114	2991 (493)	131	2971 (362)	20.00	[-88.00; 128.00]	0.8%	1.4%
West 2014	1685	2557 (425)	1701	2521 (405)	35.91	[7.57; 64.24]	12.0%	14.1%
Christian 2003	227	2744 (455)	150	2676 (442)	67.78	[-30.28; 165.85]	1.0%	1.7%
Fawzi 2007	661	3174 (524)	684	3102 (522)	72.00	[16.00; 128.00]	3.1%	4.9%
**Fixed Effect Model**					39.14	[16.14; 62.13]	18.2%	--
**Random Effects Model**					39.14	[16.14; 62.13]	--	24.5%
Heterogeneity: $I^2 = 0\%$, $\tau^2 = 0$, $p = 0.60$								
**Same Dose Iron: ≥90% adherence**								
Persson 2012	137	2709 (475)	291	2728 (454)	-19.00	[-113.00; 75.00]	1.1%	1.9%
Friis 2004	115	3046 (439)	102	3052 (407)	-6.00	[-120.00; 108.00]	0.7%	1.3%
Christian 2003	407	2681 (438)	384	2673 (428)	8.34	[-47.27; 63.96]	3.1%	4.9%
Ashorn 2015	180	2962 (447)	169	2939 (481)	24.00	[-74.00; 121.00]	1.0%	1.8%
West 2014	7898	2594 (404)	7918	2534 (416)	60.10	[46.13; 74.06]	49.4%	27.7%
Fawzi 2007	2407	3170 (493)	2374	3095 (510)	75.00	[47.00; 104.00]	11.9%	14.0%
**Fixed Effect Model**					57.77	[45.80; 69.74]	67.2%	--
**Random Effects Model**					47.70	[23.12; 72.28]	--	51.5%
Heterogeneity: $I^2 = 43\%$, $\tau^2 = 330.1515$, $p = 0.12$								
**Fixed Effect Model**					**50.52**	**[40.71; 60.33]**	**100.0%**	**--**
**Random Effects Model**					**44.68**	**[31.46; 57.90]**	**--**	**100.0%**

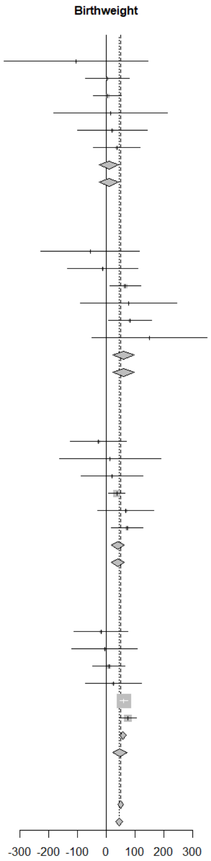

Heterogeneity: $I^2 = 12\%$, $\tau^2 = 113.6372$, $p = 0.29$

Test of subgroup differences (fixed effect): $\chi^2_3 = 8.20$, df = 3 ($p = 0.04$)

Test for subgroup differences (random effects): $\chi^2_3 = 4.52$, df = 3 ($p = 0.21$)

Objective 1 Outcomes stratified by Adherence and Gestational Age

Supplemental Figure 3.23. The effect of MMS vs. IFA on Anemia, stratified by Adherence and Gestational Age

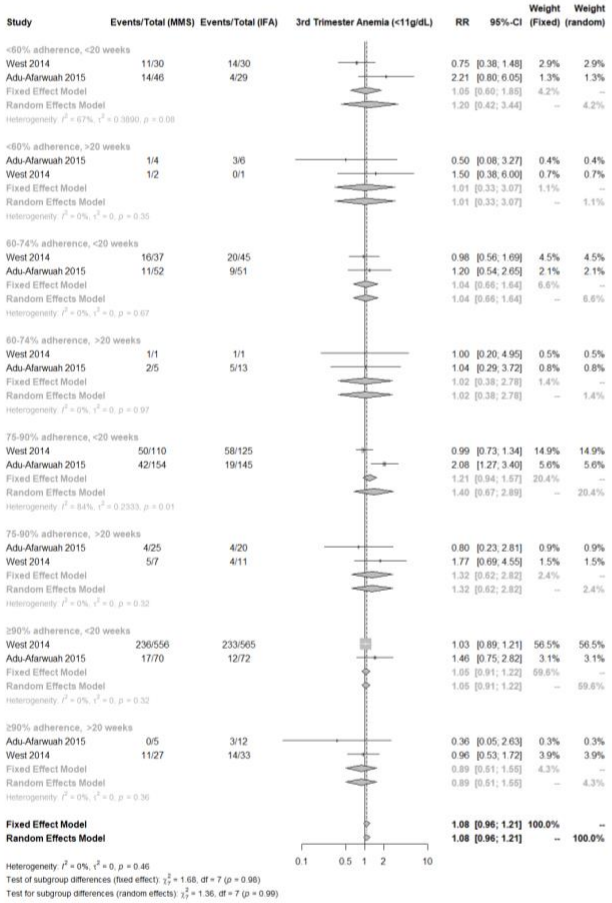

Supplemental Figure 3.24. The effect of MMS vs. IFA on Birthweight Percentile, stratified by Adherence and Gestational Age

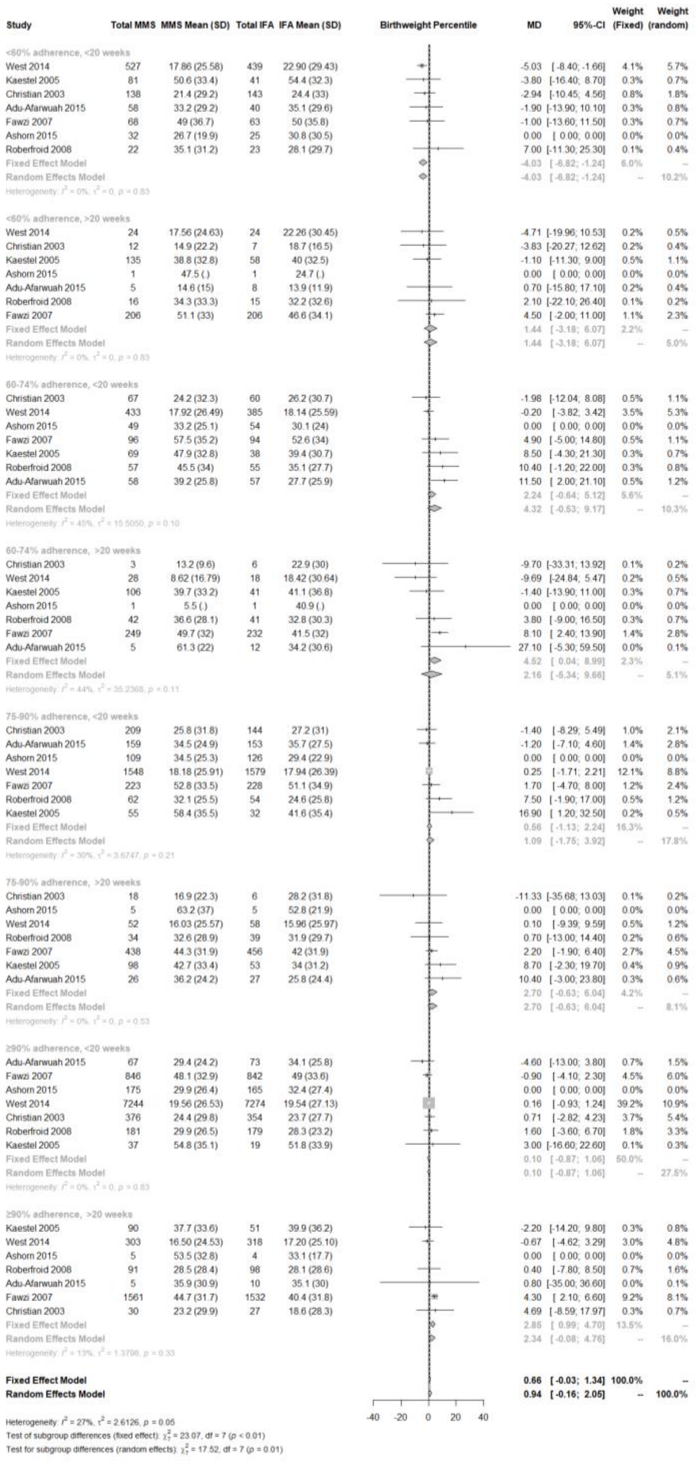

Supplemental Figure 3.25. The effect of MMS vs. IFA on Birthweight, stratified by Adherence and Gestational Age

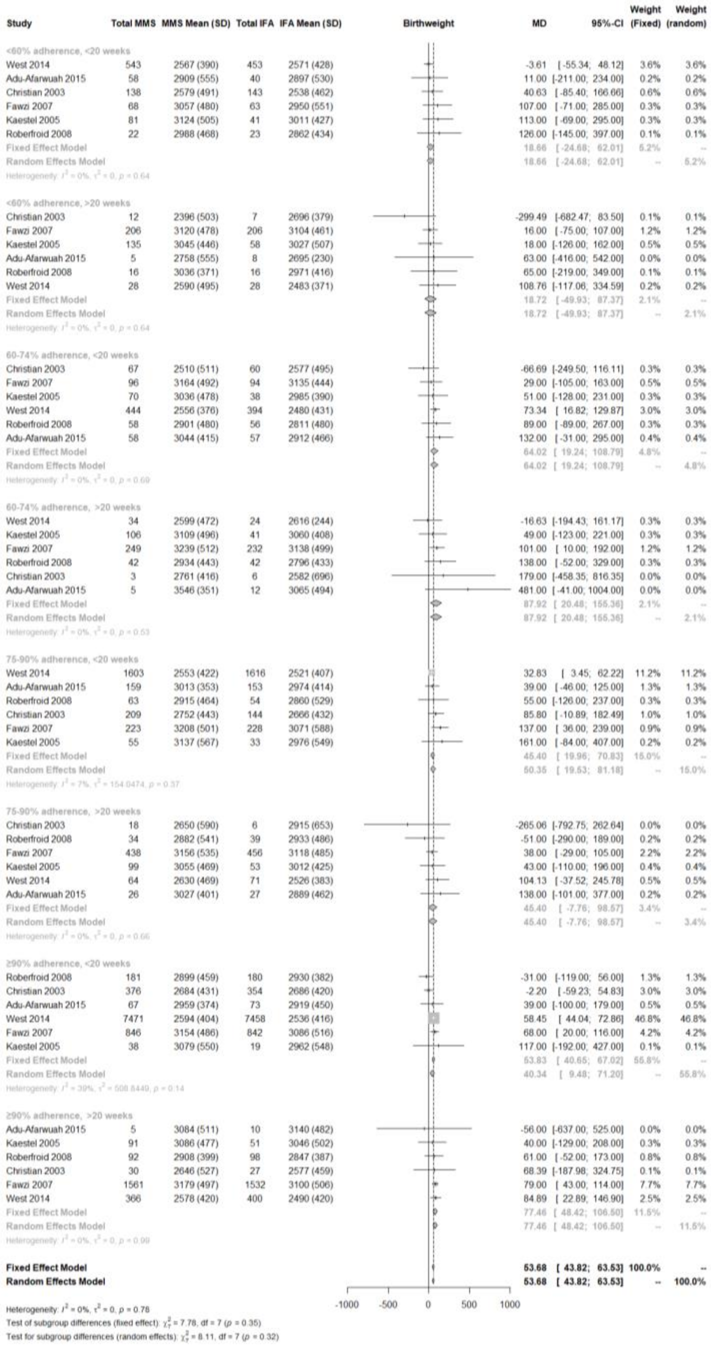

Supplemental Figure 3.26. The effect of MMS vs. IFA on Continuous Gestation, stratified by Adherence and Gestational Age

Study	Total MMS	MMS Mean (SD)	Total IFA	IFA Mean (SD)	Gestational Age	MD	95%-CI	Weight (Fixed)	Weight (random)
<60% adherence, <20 weeks									
Ashorn 2015	43	39.1 (1.8)	34	39.3 (1.6)		-0.20	[-1.00; 0.50]	0.7%	0.7%
Adu-Afarwuah 2015	63	38.9 (2.4)	47	38.9 (2.3)		0.00	[-0.90; 0.90]	0.5%	0.5%
West 2014	887	38.67 (3.05)	756	38.51 (3.23)		0.16	[-0.14; 0.46]	4.3%	4.3%
Fawzi 2007	71	38.6 (3.4)	69	38.3 (3.3)		0.30	[-0.80; 1.50]	0.3%	0.3%
Christian 2003	138	39.2 (3.1)	147	38.6 (3.6)		0.60	[-0.14; 1.33]	0.7%	0.7%
Kaestel 2005	129	37.9 (4.8)	64	37 (3.8)		0.90	[-0.40; 2.30]	0.2%	0.2%
Roberfroid 2008	26	39.4 (2.3)	29	38.5 (3.8)		0.90	[-0.90; 2.60]	0.1%	0.1%
Fixed Effect Model						0.20	[-0.04; 0.44]	6.9%	--
Random Effects Model						0.20	[-0.04; 0.44]	--	6.9%
Heterogeneity: $I^2 = 0\%$, $\tau^2 = 0$, $p = 0.65$									
<60% adherence, >20 weeks									
Roberfroid 2008	21	39.2 (4.5)	21	40.2 (3.9)		-1.00	[-3.60; 1.70]	0.1%	0.1%
Christian 2003	12	38.5 (2.7)	7	38.9 (3.2)		-0.41	[-3.02; 2.21]	0.1%	0.1%
Fawzi 2007	210	38.9 (3.1)	213	39.1 (3.2)		-0.20	[-0.80; 0.40]	1.1%	1.1%
Kaestel 2005	233	39.8 (3.7)	88	39.7 (3.6)		0.00	[-0.90; 1.00]	0.4%	0.4%
West 2014	65	39.60 (2.91)	50	39.21 (3.21)		0.40	[-0.70; 1.51]	0.3%	0.3%
Ashorn 2015	2	37.6 (1.6)	2	36.8 (3.9)		0.80	[-12.20; 13.70]	0.0%	0.0%
Adu-Afarwuah 2015	6	40.5 (1.9)	8	39.6 (1.8)		1.00	[-1.10; 3.10]	0.1%	0.1%
Fixed Effect Model						-0.04	[-0.48; 0.40]	2.0%	--
Random Effects Model						-0.04	[-0.48; 0.40]	--	2.0%
Heterogeneity: $I^2 = 0\%$, $\tau^2 = 0$, $p = 0.88$									
60-74% adherence, <20 weeks									
Kaestel 2005	94	38.7 (3.9)	50	38.8 (3.1)		-0.10	[-1.40; 1.20]	0.2%	0.2%
Roberfroid 2008	63	38 (3)	64	38 (3.3)		0.00	[-1.10; 1.10]	0.3%	0.3%
Christian 2003	68	38.4 (4)	60	38.4 (3.5)		0.01	[-1.33; 1.35]	0.2%	0.2%
Adu-Afarwuah 2015	63	39.4 (1.5)	60	39.4 (2)		0.10	[-0.50; 0.70]	1.1%	1.1%
Fawzi 2007	98	38.5 (3.6)	97	38.4 (3.8)		0.10	[-0.90; 1.10]	0.4%	0.4%
West 2014	700	38.69 (3.23)	604	38.49 (3.21)		0.20	[-0.16; 0.55]	3.0%	3.0%
Ashorn 2015	54	39.7 (1.8)	63	39.1 (2.5)		0.60	[-0.20; 1.40]	0.6%	0.6%
Fixed Effect Model						0.18	[-0.07; 0.44]	5.9%	--
Random Effects Model						0.18	[-0.07; 0.44]	--	5.9%
Heterogeneity: $I^2 = 0\%$, $\tau^2 = 0$, $p = 0.96$									
60-74% adherence, >20 weeks									
West 2014	57	39.96 (2.67)	40	40.19 (3.23)		-0.27	[-1.50; 0.97]	0.3%	0.3%
Fawzi 2007	253	39.8 (2.7)	240	39.9 (2.5)		-0.10	[-0.60; 0.30]	1.9%	1.9%
Christian 2003	3	39.1 (1.5)	6	39.1 (2.1)		0.07	[-2.03; 2.17]	0.1%	0.1%
Ashorn 2015	2	39.1 (2.9)	1	38.9 (.)		0.20	[-45.40; 45.80]	0.0%	0.0%
Kaestel 2005	145	40.3 (3.1)	55	39.8 (4.1)		0.50	[-0.60; 1.50]	0.4%	0.4%
Adu-Afarwuah 2015	5	40.4 (0.6)	13	39.8 (1.5)		0.60	[-0.90; 2.10]	0.2%	0.2%
Roberfroid 2008	56	39.3 (3.6)	55	38.6 (3)		0.60	[-0.60; 1.90]	0.2%	0.2%
Fixed Effect Model						0.06	[-0.30; 0.42]	3.0%	--
Random Effects Model						0.06	[-0.30; 0.42]	--	3.0%
Heterogeneity: $I^2 = 0\%$, $\tau^2 = 0$, $p = 0.85$									
75-90% adherence, <20 weeks									
Roberfroid 2008	72	38.8 (3.4)	60	39.7 (2.1)		-1.00	[-1.90; 0.00]	0.4%	0.4%
Kaestel 2005	66	38.3 (3.6)	46	39 (3.2)		-0.70	[-2.10; 0.60]	0.2%	0.2%
Ashorn 2015	123	39.4 (2.4)	130	39.6 (1.6)		-0.20	[-0.70; 0.30]	1.6%	1.6%
Adu-Afarwuah 2015	163	39.4 (1.6)	154	39.2 (1.8)		0.20	[-0.10; 0.60]	3.2%	3.2%
West 2014	2202	38.87 (2.97)	2186	38.66 (2.99)		0.21	[0.02; 0.40]	11.0%	11.0%
Christian 2003	209	39.4 (3)	145	38.7 (3.5)		0.64	[-0.04; 1.32]	0.8%	0.8%
Fawzi 2007	233	39.2 (3.3)	236	38.5 (3.5)		0.70	[0.10; 1.30]	1.1%	1.1%
Fixed Effect Model						0.18	[0.04; 0.33]	18.3%	--
Random Effects Model						0.13	[-0.17; 0.43]	--	18.3%
Heterogeneity: $I^2 = 59\%$, $\tau^2 = 0.0795$, $p = 0.02$									
75-90% adherence, >20 weeks									
Christian 2003	18	39.7 (2.8)	6	40.6 (2.4)		-0.91	[-3.12; 1.31]	0.1%	0.1%
Ashorn 2015	7	38.4 (2.9)	6	38.7 (2.2)		-0.30	[-3.40; 2.80]	0.0%	0.0%
Roberfroid 2008	46	39.6 (3.7)	51	39.7 (4.2)		-0.10	[-1.70; 1.40]	0.2%	0.2%
Kaestel 2005	126	40 (3.7)	65	40.1 (3.3)		0.00	[-1.10; 1.00]	0.4%	0.4%
Adu-Afarwuah 2015	26	39.3 (2)	27	39.2 (1.9)		0.10	[-1.00; 1.20]	0.3%	0.3%
Fawzi 2007	455	39.8 (2.8)	464	39.8 (2.7)		0.10	[-0.30; 0.40]	3.2%	3.2%
West 2014	88	39.69 (2.65)	97	38.91 (2.99)		0.78	[-0.02; 1.57]	0.6%	0.6%
Fixed Effect Model						0.15	[-0.13; 0.44]	4.8%	--
Random Effects Model						0.15	[-0.13; 0.44]	--	4.8%
Heterogeneity: $I^2 = 0\%$, $\tau^2 = 0$, $p = 0.73$									
≥90% adherence, <20 weeks									
Roberfroid 2008	201	39.3 (2.2)	202	39.5 (2.2)		-0.20	[-0.60; 0.20]	2.4%	2.4%
Christian 2003	376	39 (3.1)	356	39 (3)		0.02	[-0.39; 0.43]	2.3%	2.3%
West 2014	9071	38.88 (2.91)	9149	38.53 (3.07)		0.34	[0.23; 0.45]	34.0%	34.0%
Ashorn 2015	189	39.5 (2.1)	182	38.9 (2.7)		0.50	[0.00; 1.00]	1.6%	1.6%
Fawzi 2007	869	39.2 (2.9)	870	38.7 (3.1)		0.50	[0.20; 0.80]	4.3%	4.3%
Adu-Afarwuah 2015	68	39.6 (1.5)	74	39 (2)		0.70	[0.10; 1.30]	1.1%	1.1%
Kaestel 2005	55	37.5 (4.1)	25	35.9 (7.1)		1.60	[-0.90; 4.10]	0.1%	0.1%
Fixed Effect Model						0.33	[0.23; 0.42]	45.8%	--
Random Effects Model						0.30	[0.09; 0.52]	--	45.8%
Heterogeneity: $I^2 = 54\%$, $\tau^2 = 0.0364$, $p = 0.04$									
≥90% adherence, >20 weeks									
Ashorn 2015	5	36.5 (5.2)	4	39.6 (2)		-3.10	[-9.60; 3.50]	0.0%	0.0%
Christian 2003	30	39.6 (3.9)	27	39.8 (3.4)		-0.18	[-2.07; 1.72]	0.1%	0.1%
Adu-Afarwuah 2015	5	40 (1.1)	12	39.7 (1.4)		0.20	[-1.30; 1.80]	0.2%	0.2%
Fawzi 2007	1617	40 (2.9)	1572	39.9 (2.8)		0.20	[0.00; 0.40]	9.8%	9.8%
West 2014	397	39.52 (2.82)	444	39.09 (3.07)		0.42	[0.01; 0.83]	2.3%	2.3%
Roberfroid 2008	118	39.8 (3.4)	128	39.3 (3)		0.50	[-0.30; 1.30]	0.6%	0.6%
Kaestel 2005	123	40.3 (3.5)	73	39.7 (3.9)		0.60	[-0.50; 1.60]	0.4%	0.4%
Fixed Effect Model						0.26	[0.09; 0.43]	13.3%	--
Random Effects Model						0.26	[0.09; 0.43]	--	13.3%
Heterogeneity: $I^2 = 0\%$, $\tau^2 = 0$, $p = 0.82$									
**Fixed Effect Model**						**0.25**	**[0.19; 0.31]**	**100.0%**	**--**
**Random Effects Model**						**0.25**	**[0.19; 0.31]**	**--**	**100.0%**

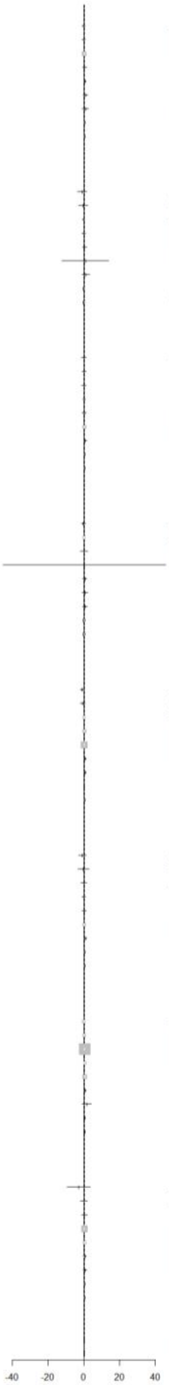

Heterogeneity: $I^2 = 0\%$, $\tau^2 = 0$, $p = 0.59$
Test of subgroup differences (fixed effect): $\chi^2_7 = 7.08$, df = 7 ($p = 0.42$)
Test for subgroup differences (random effects): $\chi^2_7 = 3.42$, df = 7 ($p = 0.84$)

Supplemental Figure 3.27. The effect of MMS vs. IFA on Fetal Death, stratified by Adherence and Gestational Age

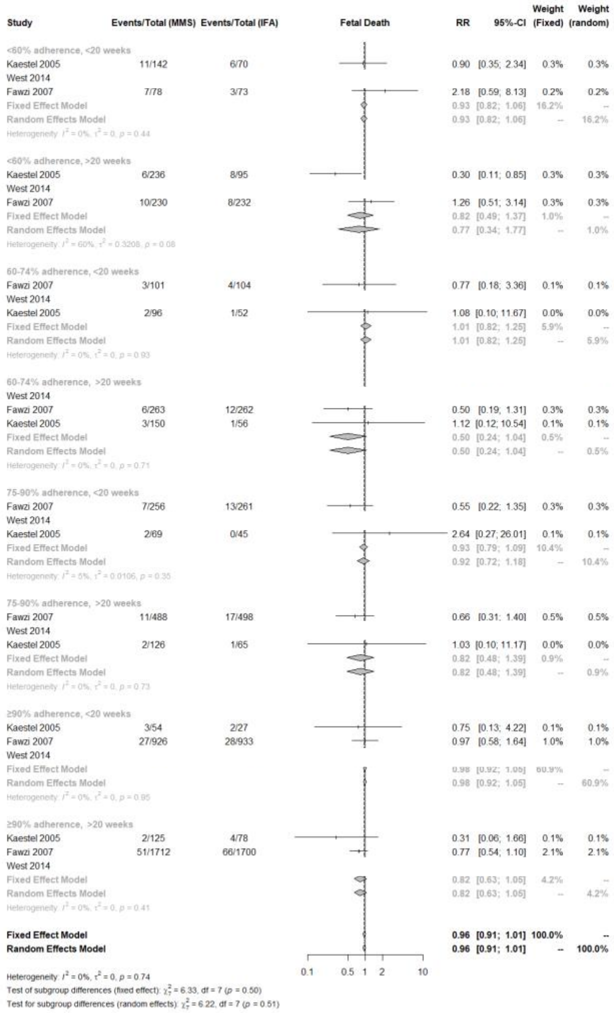

Supplemental Figure 3.28. The effect of MMS vs. IFA on Hemoglobin Continuous, stratified by Adherence and Gestational Age

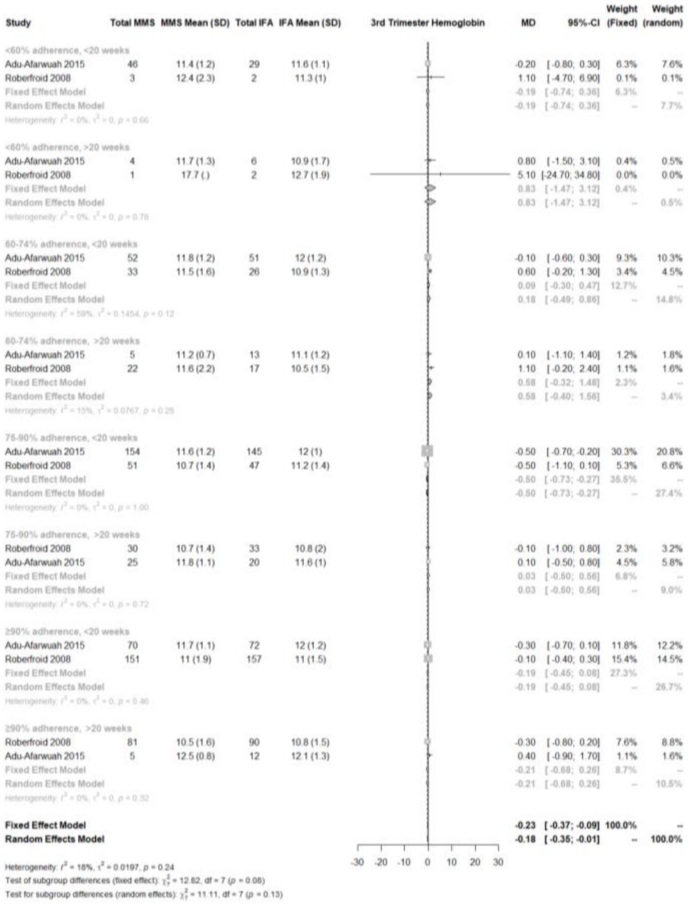

Supplemental Figure 3.29. The effect of MMS vs. IFA on IDA, stratified by Adherence and Gestational Age

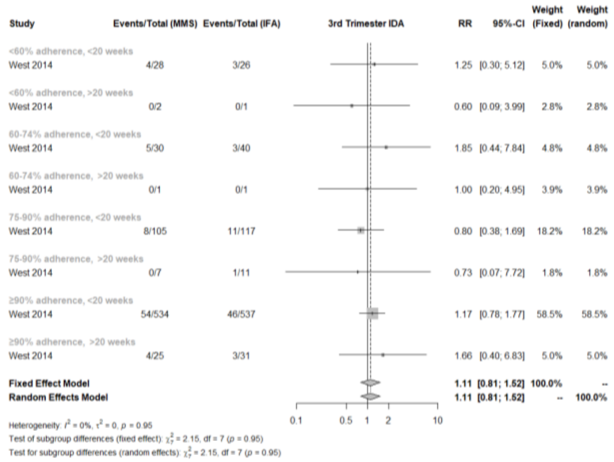

Supplemental Figure 3.30. The effect of MMS vs. IFA on Infant Mortality, stratified by Adherence and Gestational Age

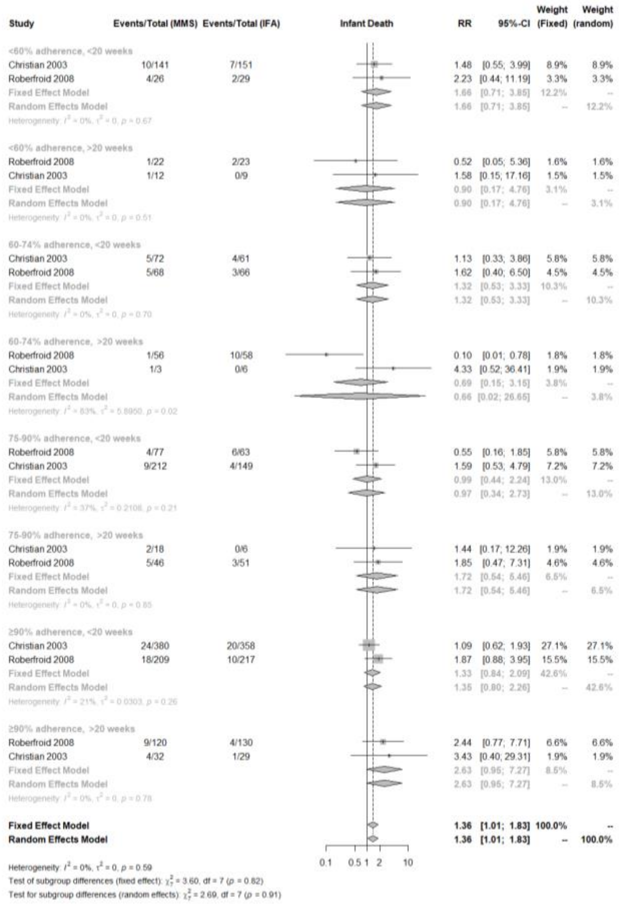

Supplemental Figure 3.32. The effect of MMS vs. IFA on LGA90, stratified by Adherence and Gestational Age

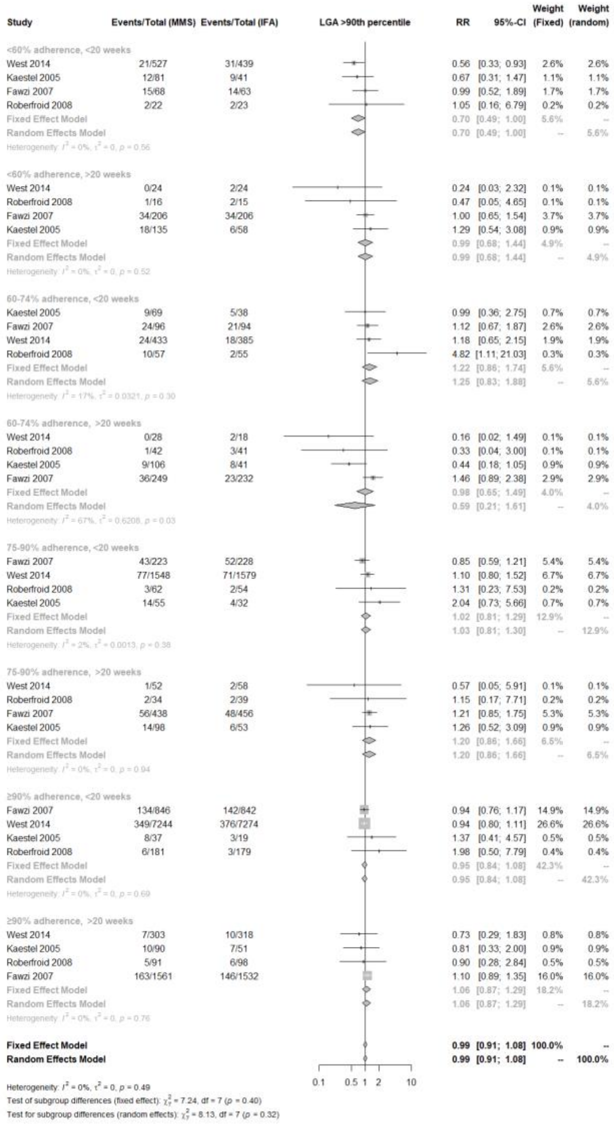

Supplemental Figure 3.32. The effect of MMS vs. IFA on Low Birthweight, stratified by Adherence and Gestational Age

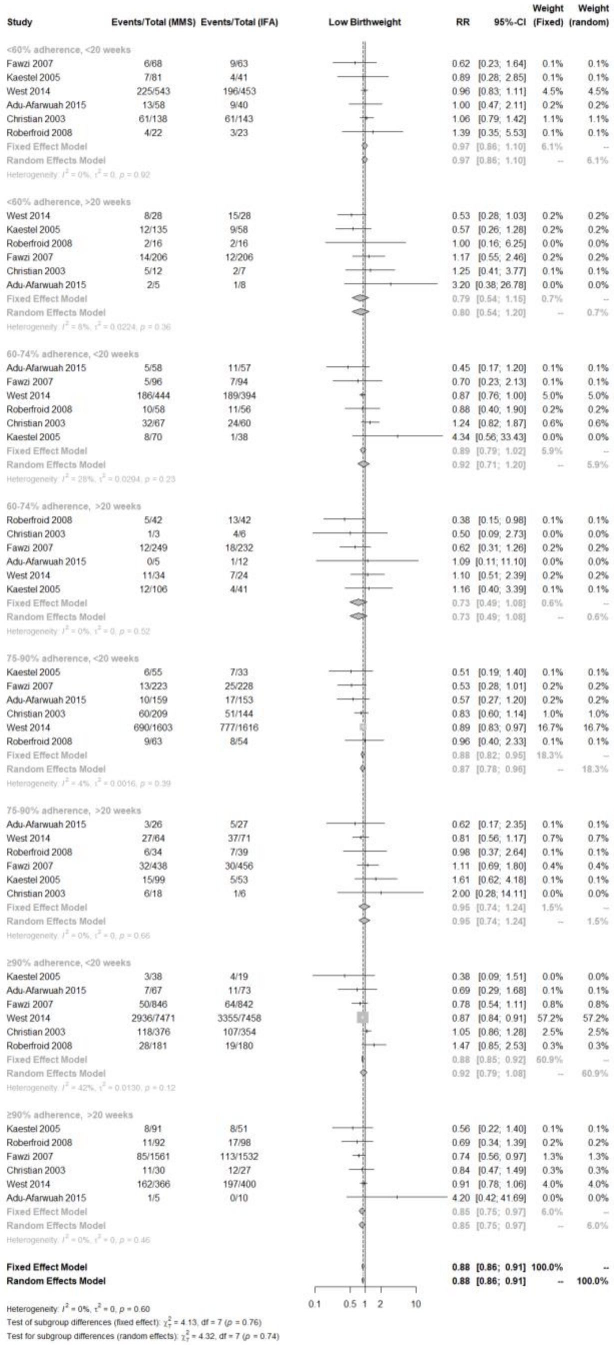

Supplemental Figure 3.33. The effect of MMS vs. IFA on Neonatal Mortality, stratified by Adherence and Gestational Age

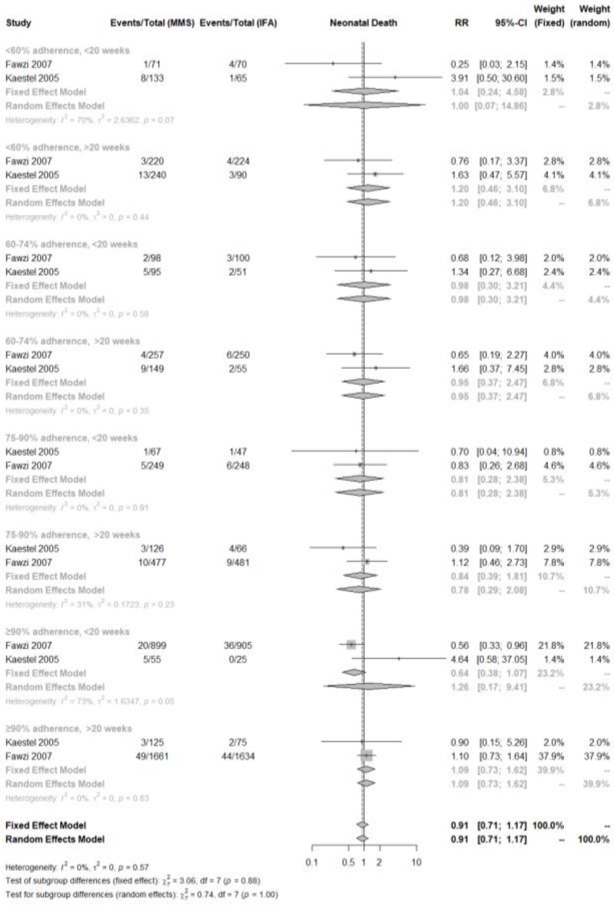

Supplemental Figure 3.34. The effect of MMS vs. IFA on Preterm, stratified by Adherence and Gestational Age

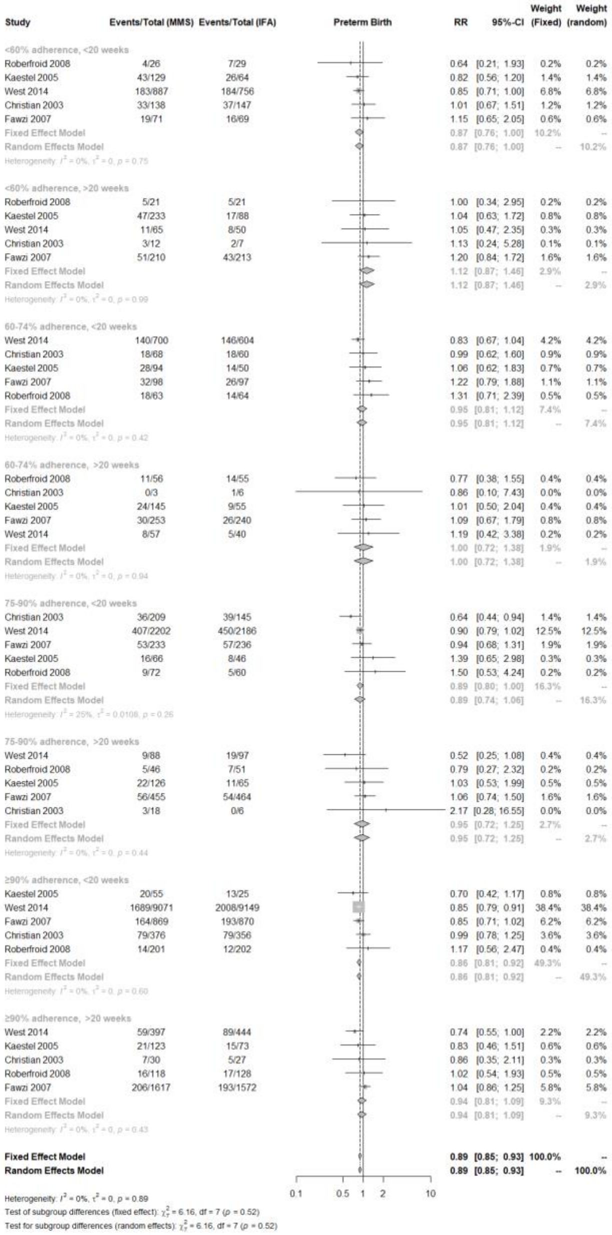

Supplemental Figure 3.35. The effect of MMS vs. IFA on SGA10, stratified by Adherence and Gestational Age

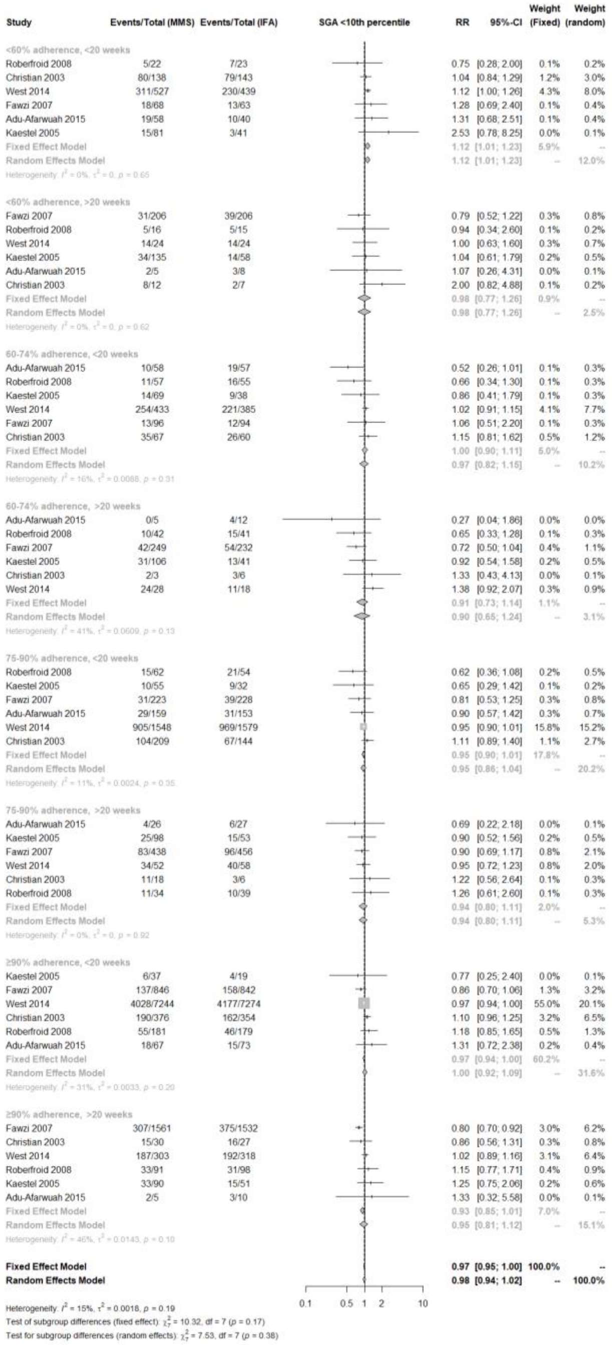

Supplemental Figure 3.36. The effect of MMS vs. IFA on Stillbirth, stratified by Adherence and Gestational Age

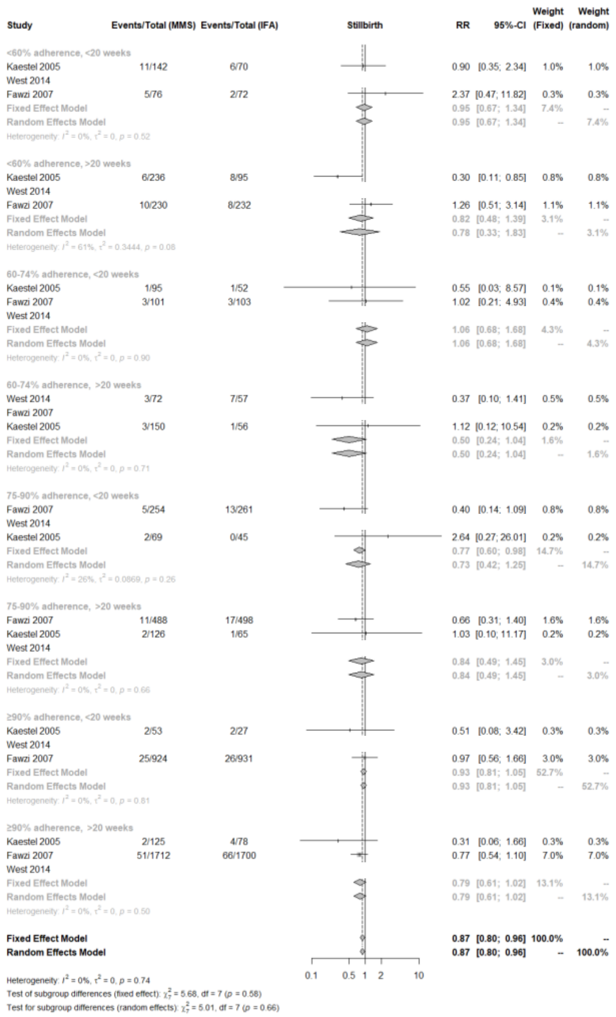

Objective 1 Sensivity analysis by MMS iron dose stratified by Adherence and Gestational Age

Supplemental Figure 3.37. The effect of MMS vs. IFA on hemoglobin, in trials with lower iron dose in MMS than IFA, stratified by adherence and gestational age

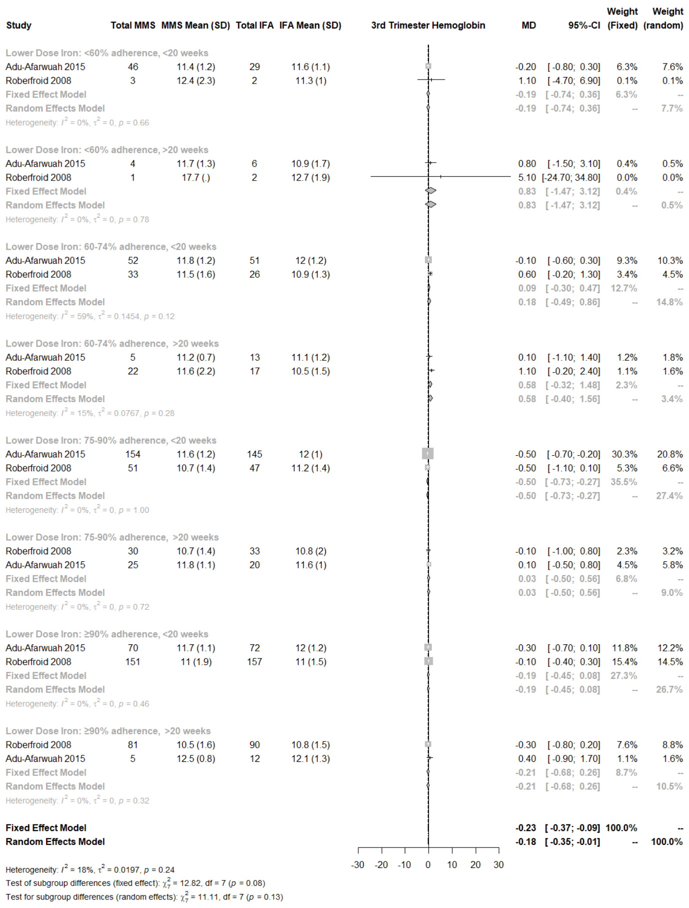

Supplemental Figure 3.38. The effect of MMS vs. IFA on anemia, in trials with lower iron dose in MMS than IFA, stratified by adherence and gestational age

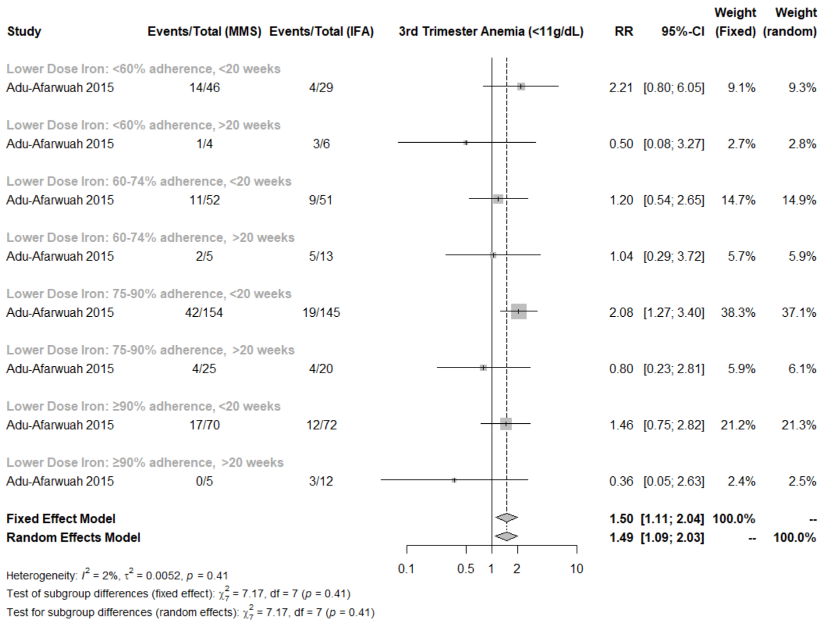

Supplemental Figure 3.39. The effect of MMS vs. IFA on anemia, in trials with same iron dose in MMS than IFA, stratified by adherence and gestational age

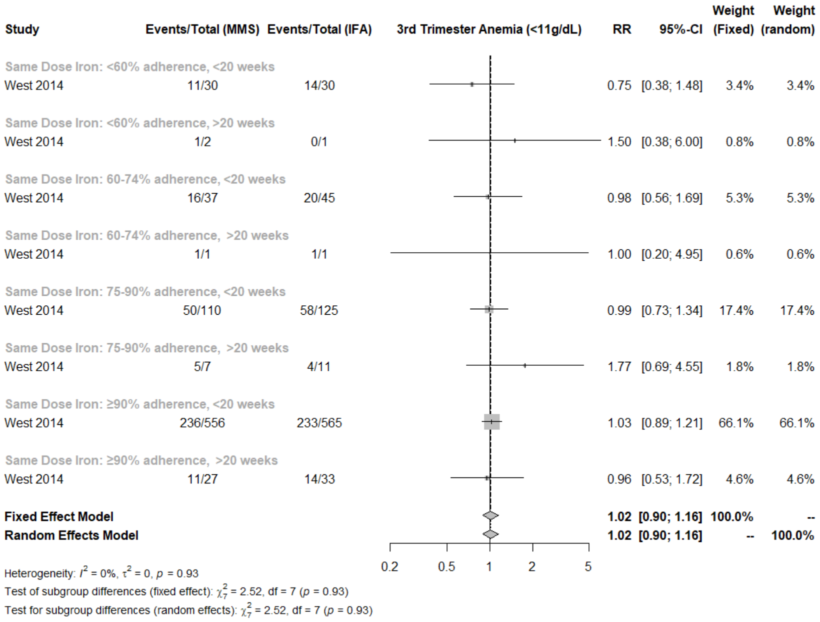

Supplemental Figure 3.40. The effect of MMS vs. IFA on anemia, in trials with same iron dose in MMS than IFA, stratified by adherence and gestational age

Study	Total MMS	MMS Mean (SD)	Total IFA	IFA Mean (SD)	MD	95%-CI	Weight (Fixed)	Weight (random)
**Lower Dose Iron: <60% adherence, <20 weeks**								
Adu-Afarwuah 2015	58	2909 (555)	40	2897 (530)	11.00	[-211.00; 234.00]	2.3%	2.3%
Kaestel 2005	81	3124 (505)	41	3011 (427)	113.00	[-69.00; 295.00]	3.5%	3.5%
Roberfroid 2008	22	2988 (468)	23	2862 (434)	126.00	[-145.00; 397.00]	1.6%	1.6%
Fixed Effect Model					83.58	[-41.42; 208.57]	7.4%	--
Random Effects Model					83.58	[-41.42; 208.57]	--	7.4%
Heterogeneity: $I^2 = 0\%$, $\tau^2 = 0$, $p = 0.74$								
**Lower Dose Iron: <60% adherence, >20 weeks**								
Kaestel 2005	135	3045 (446)	58	3027 (507)	18.00	[-126.00; 162.00]	5.6%	5.6%
Adu-Afarwuah 2015	5	2758 (555)	8	2695 (230)	63.00	[-416.00; 542.00]	0.5%	0.5%
Roberfroid 2008	16	3036 (371)	16	2971 (416)	65.00	[-219.00; 349.00]	1.4%	1.4%
Fixed Effect Model					29.99	[-94.07; 154.04]	7.5%	--
Random Effects Model					29.99	[-94.07; 154.04]	--	7.5%
Heterogeneity: $I^2 = 0\%$, $\tau^2 = 0$, $p = 0.95$								
**Lower Dose Iron: 60-74% adherence, <20 weeks**								
Kaestel 2005	70	3036 (478)	38	2985 (390)	51.00	[-128.00; 231.00]	3.6%	3.6%
Roberfroid 2008	58	2901 (480)	56	2811 (480)	89.00	[-89.00; 267.00]	3.6%	3.6%
Adu-Afarwuah 2015	58	3044 (415)	57	2912 (466)	132.00	[-31.00; 295.00]	4.3%	4.3%
Fixed Effect Model					93.38	[-6.50; 193.26]	11.6%	--
Random Effects Model					93.38	[-6.50; 193.26]	--	11.6%
Heterogeneity: $I^2 = 0\%$, $\tau^2 = 0$, $p = 0.81$								
**Lower Dose Iron: 60-74% adherence, >20 weeks**								
Kaestel 2005	106	3109 (496)	41	3060 (408)	49.00	[-123.00; 221.00]	3.9%	3.9%
Roberfroid 2008	42	2934 (443)	42	2796 (433)	138.00	[-52.00; 329.00]	3.2%	3.2%
Adu-Afarwuah 2015	5	3546 (351)	12	3065 (494)	481.00	[-41.00; 1004.00]	0.4%	0.4%
Fixed Effect Model					111.05	[-12.96; 235.07]	7.5%	--
Random Effects Model					119.79	[-27.35; 266.93]	--	7.5%
Heterogeneity: $I^2 = 20\%$, $\tau^2 = 3679.0697$, $p = 0.29$								
**Lower Dose Iron: 75-90% adherence, <20 weeks**								
Adu-Afarwuah 2015	159	3013 (353)	153	2974 (414)	39.00	[-46.00; 125.00]	15.8%	15.8%
Roberfroid 2008	63	2915 (464)	54	2860 (529)	55.00	[-126.00; 237.00]	3.5%	3.5%
Kaestel 2005	55	3137 (567)	33	2976 (549)	161.00	[-84.00; 407.00]	1.9%	1.9%
Fixed Effect Model					52.66	[-21.11; 126.43]	21.2%	--
Random Effects Model					52.66	[-21.11; 126.43]	--	21.2%
Heterogeneity: $I^2 = 0\%$, $\tau^2 = 0$, $p = 0.65$								
**Lower Dose Iron: 75-90% adherence, >20 weeks**								
Roberfroid 2008	34	2882 (541)	39	2933 (486)	-51.00	[-290.00; 189.00]	2.0%	2.0%
Kaestel 2005	99	3055 (469)	53	3012 (425)	43.00	[-110.00; 196.00]	4.9%	4.9%
Adu-Afarwuah 2015	26	3027 (401)	27	2889 (462)	138.00	[-101.00; 377.00]	2.0%	2.0%
Fixed Effect Model					43.31	[-70.16; 156.79]	9.0%	--
Random Effects Model					43.31	[-70.16; 156.79]	--	9.0%
Heterogeneity: $I^2 = 0\%$, $\tau^2 = 0$, $p = 0.55$								
**Lower Dose Iron: ≥90% adherence, <20 weeks**								
Roberfroid 2008	181	2899 (459)	180	2930 (382)	-31.00	[-119.00; 56.00]	15.1%	15.1%
Adu-Afarwuah 2015	67	2959 (374)	73	2919 (450)	39.00	[-100.00; 179.00]	5.9%	5.9%
Kaestel 2005	38	3079 (550)	19	2962 (548)	117.00	[-192.00; 427.00]	1.2%	1.2%
Fixed Effect Model					-4.28	[-76.37; 67.81]	22.2%	--
Random Effects Model					-4.28	[-76.37; 67.81]	--	22.2%
Heterogeneity: $I^2 = 0\%$, $\tau^2 = 0$, $p = 0.52$								
**Lower Dose Iron: ≥90% adherence, >20 weeks**								
Adu-Afarwuah 2015	5	3084 (511)	10	3140 (482)	-56.00	[-637.00; 525.00]	0.3%	0.3%
Kaestel 2005	91	3086 (477)	51	3046 (502)	40.00	[-129.00; 208.00]	4.1%	4.1%
Roberfroid 2008	92	2908 (399)	98	2847 (387)	61.00	[-52.00; 173.00]	9.1%	9.1%
Fixed Effect Model					51.73	[-40.64; 144.10]	13.5%	--
Random Effects Model					51.73	[-40.64; 144.10]	--	13.5%
Heterogeneity: $I^2 = 0\%$, $\tau^2 = 0$, $p = 0.92$								
**Fixed Effect Model**					**48.72**	**[14.73; 82.72]**	**100.0%**	**--**
**Random Effects Model**					**48.72**	**[14.73; 82.72]**	**--**	**100.0%**

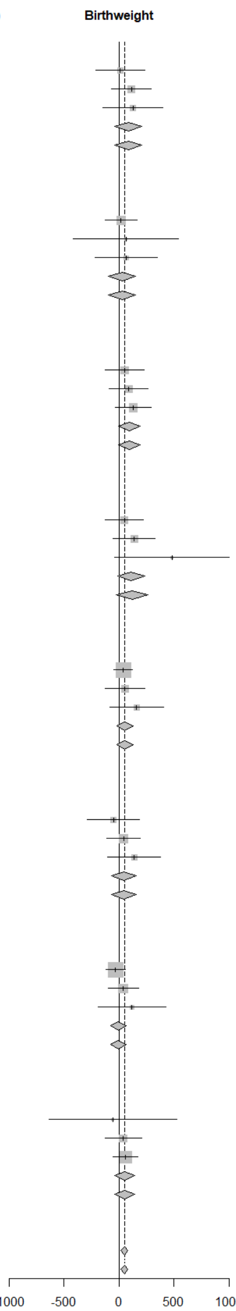

Heterogeneity: $I^2 = 0\%$, $\tau^2 = 0$, $p = 0.98$
Test of subgroup differences (fixed effect): $\chi^2_7 = 4.23$, df = 7 ($p = 0.75$)
Test for subgroup differences (random effects): $\chi^2_7 = 4.15$, df = 7 ($p = 0.76$)

Supplemental Figure 3.41. The effect of MMS vs. IFA on birthweight, in trials with same iron dose in MMS than IFA, stratified by adherence and gestational age

Study	Total MMS	MMS Mean (SD)	Total IFA	IFA Mean (SD)	MD	95%-CI	Weight (Fixed)	Weight (random)
**Same Dose Iron: <60% adherence, <20 weeks**								
West 2014	543	2567 (390)	453	2571 (428)	-3.61	[-55.34; 48.12]	4.0%	6.6%
Christian 2003	138	2579 (491)	143	2538 (462)	40.63	[-85.40; 166.66]	0.7%	1.3%
Fawzi 2007	68	3057 (480)	63	2950 (551)	107.00	[-71.00; 285.00]	0.3%	0.7%
Fixed Effect Model					9.79	[-36.42; 56.01]	5.0%	--
Random Effects Model					9.79	[-36.42; 56.01]	--	8.6%
Heterogeneity: I^2 = 0%, τ^2 = 0, p = 0.44								
**Same Dose Iron: <60% adherence, >20 weeks**								
Christian 2003	12	2396 (503)	7	2696 (379)	-299.49	[-682.47; 83.50]	0.1%	0.2%
Fawzi 2007	206	3120 (478)	206	3104 (461)	16.00	[-75.00; 107.00]	1.3%	2.5%
West 2014	28	2590 (495)	28	2483 (371)	108.76	[-117.06; 334.59]	0.2%	0.4%
Fixed Effect Model					13.74	[-68.68; 96.17]	1.6%	--
Random Effects Model					2.36	[-144.73; 149.45]	--	3.1%
Heterogeneity: I^2 = 39%, τ^2 = 7214.1803, p = 0.20								
**Same Dose Iron: 60-74% adherence, <20 weeks**								
Christian 2003	67	2510 (511)	60	2577 (495)	-66.69	[-249.50; 116.11]	0.3%	0.7%
Fawzi 2007	96	3164 (492)	94	3135 (444)	29.00	[-105.00; 163.00]	0.6%	1.2%
West 2014	444	2556 (376)	394	2480 (431)	73.34	[16.82; 129.87]	3.3%	5.7%
Fixed Effect Model					56.63	[6.54; 106.72]	4.2%	--
Random Effects Model					51.24	[-8.29; 110.77]	--	7.5%
Heterogeneity: I^2 = 11%, τ^2 = 450.9123, p = 0.33								
**Same Dose Iron: 60-74% adherence, >20 weeks**								
West 2014	34	2599 (472)	24	2616 (244)	-16.63	[-194.43; 161.17]	0.3%	0.7%
Fawzi 2007	249	3239 (512)	232	3138 (499)	101.00	[10.00; 192.00]	1.3%	2.5%
Christian 2003	3	2761 (416)	6	2582 (696)	179.00	[-458.35; 816.35]	0.0%	0.1%
Fixed Effect Model					78.21	[-2.15; 158.57]	1.6%	--
Random Effects Model					78.21	[-2.15; 158.57]	--	3.2%
Heterogeneity: I^2 = 0%, τ^2 = 0, p = 0.49								
**Same Dose Iron: 75-90% adherence, <20 weeks**								
West 2014	1603	2553 (422)	1616	2521 (407)	32.83	[3.45; 62.22]	12.3%	14.1%
Christian 2003	209	2752 (443)	144	2666 (432)	85.80	[-10.89; 182.49]	1.1%	2.2%
Fawzi 2007	223	3208 (501)	228	3071 (588)	137.00	[36.00; 239.00]	1.0%	2.0%
Fixed Effect Model					44.42	[17.32; 71.51]	14.4%	--
Random Effects Model					70.31	[6.89; 133.73]	--	18.4%
Heterogeneity: I^2 = 56%, τ^2 = 1794.4939, p = 0.11								
**Same Dose Iron: 75-90% adherence, >20 weeks**								
Christian 2003	18	2650 (590)	6	2915 (653)	-265.06	[-792.75; 262.64]	0.0%	0.1%
Fawzi 2007	438	3156 (535)	456	3118 (485)	38.00	[-29.00; 105.00]	2.4%	4.3%
West 2014	64	2630 (469)	71	2526 (383)	104.13	[-37.52; 245.78]	0.5%	1.1%
Fixed Effect Model					45.99	[-14.18; 106.16]	2.9%	--
Random Effects Model					46.36	[-16.12; 108.84]	--	5.4%
Heterogeneity: I^2 = 2%, τ^2 = 108.8063, p = 0.36								
**Same Dose Iron: ≥90% adherence, <20 weeks**								
Christian 2003	376	2684 (431)	354	2686 (420)	-2.20	[-59.23; 54.83]	3.3%	5.6%
West 2014	7471	2594 (404)	7458	2536 (416)	58.45	[44.04; 72.86]	51.1%	24.3%
Fawzi 2007	846	3154 (486)	842	3086 (516)	68.00	[20.00; 116.00]	4.6%	7.4%
Fixed Effect Model					55.84	[42.43; 69.25]	58.9%	--
Random Effects Model					48.22	[15.82; 80.62]	--	37.3%
Heterogeneity: I^2 = 54%, τ^2 = 459.1189, p = 0.11								
**Same Dose Iron: ≥90% adherence, >20 weeks**								
Christian 2003	30	2646 (527)	27	2577 (459)	68.39	[-187.98; 324.75]	0.2%	0.3%
Fawzi 2007	1561	3179 (497)	1532	3100 (506)	79.00	[43.00; 114.00]	8.4%	11.3%
West 2014	366	2578 (420)	400	2490 (420)	84.89	[22.89; 146.90]	2.8%	4.9%
Fixed Effect Model					80.28	[49.69; 110.87]	11.3%	--
Random Effects Model					80.28	[49.69; 110.87]	--	16.5%
Heterogeneity: I^2 = 0%, τ^2 = 0, p = 0.98								
**Fixed Effect Model**					**54.13**	**[43.84; 64.43]**	**100.0%**	**--**
**Random Effects Model**					**52.25**	**[37.34; 67.15]**	**--**	**100.0%**

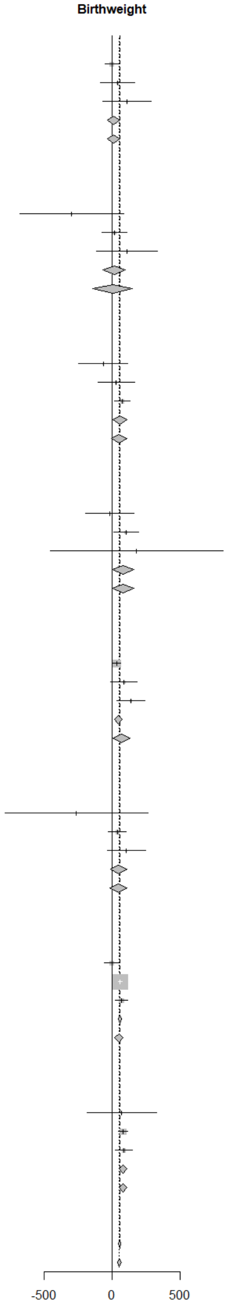

Heterogeneity: I^2 = 17%, τ^2 = 183.9758, p = 0.23
Test of subgroup differences (fixed effect): χ^2_7 = 8.25, df = 7 (p = 0.31)
Test for subgroup differences (random effects): χ^2_7 = 7.59, df = 7 (p = 0.37)

Objective 1 Outcomes stratified by Total Pill Count

Supplemental Figure 3.42. The effect of MMS vs. IFA on Anemia, stratified by Total Pill Count

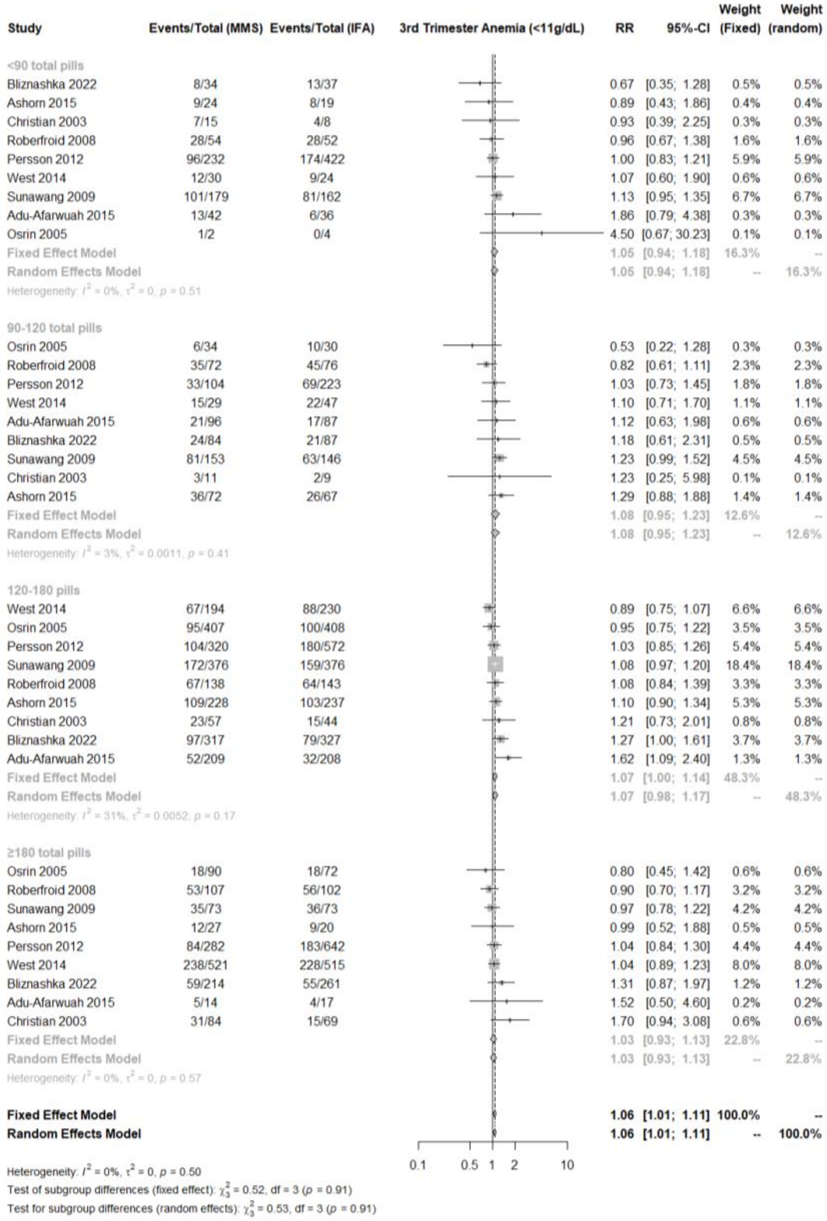

Supplemental Figure 3.43. The effect of MMS vs. IFA on Birthweight Percentile, stratified by Total Pill Count

Study	Total MMS	MMS Mean (SD)	Total IFA	IFA Mean (SD)	MD	95%-CI	Weight (Fixed)	Weight (random)
**<90 total pills**								
Zagre 2007	27	90.9 (23.6)	26	98.9 (5.7)	-8.00	[-18.54; 2.54]	0.3%	0.6%
West 2014	583	21.91 (30.20)	555	27.72 (32.71)	-5.81	[-9.55; -2.08]	2.1%	3.5%
Christian 2003	143	24.2 (31.6)	121	28.4 (35.5)	-4.28	[-12.30; 3.73]	0.4%	1.0%
Sunawang 2009	179	56.2 (35.3)	169	60 (34.5)	-3.81	[-10.39; 2.77]	0.7%	1.5%
Persson 2012	235	16.6 (21.1)	428	19.4 (24.4)	-2.80	[-6.50; 0.90]	2.1%	3.5%
Adu-Afarwuah 2015	59	31 (29.5)	48	31.5 (29.9)	-0.50	[-11.90; 11.00]	0.2%	0.5%
Kaestel 2005	382	47 (34.4)	175	46.2 (34.9)	0.80	[-5.40; 7.00]	0.7%	1.6%
Fawzi 2007	893	56.2 (33.3)	897	53.6 (34.4)	2.70	[-0.50; 5.80]	2.9%	4.3%
Ashorn 2015	34	37 (24.7)	32	34 (30.1)	3.10	[-10.40; 16.60]	0.2%	0.4%
Roberfroid 2008	110	44.3 (34)	108	39.8 (33.2)	4.50	[-4.50; 13.50]	0.4%	0.8%
Bhutta 2009	64	61.9 (38.5)	35	56.8 (39.2)	5.10	[-9.52; 19.73]	0.1%	0.3%
Osrin 2005	6	38.4 (31.2)	5	32.4 (23.4)	6.00	[-32.40; 44.30]	0.0%	0.0%
Fixed Effect Model					-1.31	[-3.00; 0.38]	10.0%	--
Random Effects Model					-1.39	[-3.95; 1.18]	--	18.2%
Heterogeneity: $I^2 = 40\%$, $\tau^2 = 6.7441$, $p = 0.07$								
**90-120 total pills**								
Bhutta 2009	78	63 (38.7)	79	68.6 (35.6)	-5.60	[-20.66; 9.45]	0.1%	0.3%
Osrin 2005	35	23.5 (26.2)	31	27.6 (24.7)	-4.10	[-16.70; 8.40]	0.2%	0.4%
Zagre 2007	80	90.4 (24.4)	66	93.8 (22.6)	-3.48	[-12.19; 5.23]	0.4%	0.9%
Persson 2012	100	17.2 (20.7)	226	20.6 (24)	-3.40	[-8.90; 2.00]	1.0%	2.0%
West 2014	372	31.56 (36.64)	387	34.37 (39.16)	-2.98	[-8.50; 2.55]	0.9%	1.9%
Christian 2003	78	31.7 (36.7)	77	32.7 (38.1)	-0.98	[-12.59; 10.62]	0.2%	0.5%
Ashorn 2015	77	36.2 (26.4)	80	34.2 (27.8)	2.00	[-6.60; 10.50]	0.4%	0.9%
Sunawang 2009	157	62 (35.3)	149	58.5 (34.3)	3.50	[-6.81; 13.81]	0.3%	0.6%
Kaestel 2005	167	39.5 (33.7)	90	35.4 (31)	4.10	[-4.40; 12.50]	0.4%	0.9%
Roberfroid 2008	102	33 (29.8)	105	28.7 (26.6)	4.30	[-3.50; 12.00]	0.5%	1.1%
Fawzi 2007	1106	47.9 (32)	1166	43.1 (32.3)	4.80	[2.20; 7.50]	4.1%	5.2%
Adu-Afarwuah 2015	101	38.6 (25.8)	107	33 (28.3)	5.60	[-1.90; 13.00]	0.5%	1.2%
Fixed Effect Model					2.11	[0.32; 3.90]	8.9%	--
Random Effects Model					1.12	[-1.46; 3.69]	--	16.1%
Heterogeneity: $I^2 = 33\%$, $\tau^2 = 5.9366$, $p = 0.13$								
**120-180 pills**								
Zagre 2007	676	77 (30.1)	540	78.9 (28)	-1.90	[-6.83; 3.03]	1.2%	2.3%
West 2014	2581	25.90 (32.66)	2692	27.26 (33.47)	-1.31	[-3.38; 0.76]	6.7%	6.5%
Bhutta 2009	364	66.1 (36.8)	475	66.7 (36.5)	-0.60	[-12.72; 11.52]	0.2%	0.5%
Adu-Afarwuah 2015	209	33.9 (24)	207	34.1 (25.9)	-0.20	[-5.00; 4.60]	1.2%	2.4%
Ashorn 2015	240	31.1 (25.8)	252	31 (24.1)	0.10	[-4.40; 4.50]	1.4%	2.7%
Persson 2012	323	18.2 (21.6)	586	17.9 (21.7)	0.30	[-2.70; 3.20]	3.3%	4.7%
Fawzi 2007	1611	41.7 (31.3)	1517	40 (31.5)	1.70	[-0.50; 3.90]	5.9%	6.2%
Christian 2003	267	29.4 (33.5)	250	27.2 (29.5)	2.16	[-3.25; 7.56]	1.0%	2.0%
Roberfroid 2008	166	26.5 (23.7)	166	24.1 (23.1)	2.40	[-2.60; 7.50]	1.1%	2.3%
Sunawang 2009	371	49.9 (33.3)	367	47.4 (31.3)	2.49	[-0.89; 5.87]	2.5%	4.0%
Kaestel 2005	109	41.3 (31.8)	63	38 (33.6)	3.30	[-6.80; 13.50]	0.3%	0.7%
Osrin 2005	414	22.7 (22.9)	435	18.9 (21.9)	3.80	[0.80; 6.80]	3.2%	4.6%
Fixed Effect Model					0.85	[-0.16; 1.86]	28.0%	--
Random Effects Model					0.89	[-0.19; 1.97]	--	38.9%
Heterogeneity: $I^2 = 7\%$, $\tau^2 = 0.2696$, $p = 0.37$								
**≥180 total pills**								
Persson 2012	282	17.1 (22)	656	19.2 (23.2)	-2.20	[-5.30; 1.00]	2.9%	4.3%
Christian 2003	365	18 (23.6)	299	18.5 (22.8)	-0.44	[-3.68; 2.80]	2.7%	4.2%
West 2014	6623	15.42 (21.20)	6461	14.34 (20.49)	1.13	[0.32; 1.94]	43.4%	9.6%
Bhutta 2009	177	57 (36)	192	54.9 (37.8)	2.11	[-7.77; 11.99]	0.3%	0.7%
Sunawang 2009	71	34 (26.7)	72	31.7 (26.8)	2.37	[-3.24; 7.98]	0.9%	1.9%
Adu-Afarwuah 2015	14	24.2 (30)	18	21.8 (22.9)	2.40	[-16.70; 21.50]	0.1%	0.2%
Roberfroid 2008	127	30.8 (25.7)	125	27.9 (22.7)	2.90	[-3.20; 8.90]	0.8%	1.7%
Kaestel 2005	13	48.1 (36.3)	5	43.6 (25.7)	4.50	[-33.40; 42.40]	0.0%	0.1%
Zagre 2007	644	46.4 (34.4)	657	41.7 (33.2)	4.66	[-2.79; 12.10]	0.5%	1.2%
Osrin 2005	94	20.7 (19.4)	71	15.5 (15.8)	5.20	[-0.40; 10.70]	0.9%	1.9%
Ashorn 2015	26	23.9 (25.3)	17	16.7 (22.6)	7.20	[-8.10; 22.50]	0.1%	0.3%
Fawzi 2007	77	38.6 (31.6)	73	30.7 (30.7)	7.90	[-2.20; 17.90]	0.3%	0.7%
Fixed Effect Model					1.08	[0.34; 1.81]	53.0%	--
Random Effects Model					1.07	[0.30; 1.85]	--	26.8%
Heterogeneity: $I^2 = 0\%$, $\tau^2 = 0.0240$, $p = 0.44$								
**Fixed Effect Model**					**0.87**	**[0.33; 1.40]**	**100.0%**	**--**
**Random Effects Model**					**0.71**	**[-0.15; 1.56]**	**--**	**100.0%**

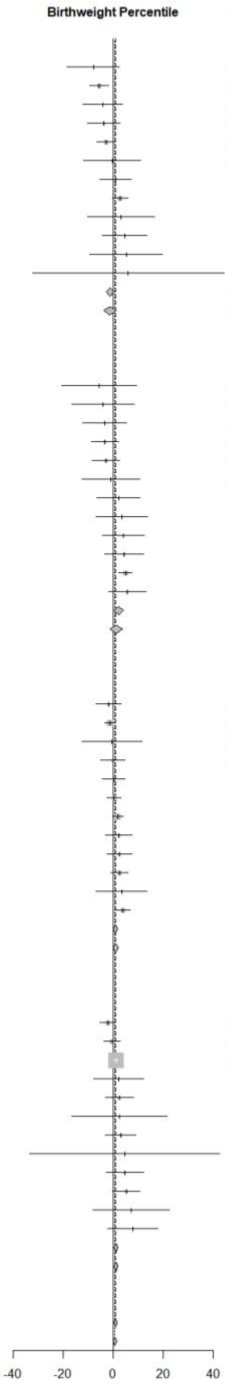

Heterogeneity: $I^2 = 29\%$, $\tau^2 = 1.8051$, $p = 0.03$
Test of subgroup differences (fixed effect): $\chi^2_3 = 8.53$, df = 3 ($p = 0.04$)
Test for subgroup differences (random effects): $\chi^2_3 = 3.27$, df = 3 ($p = 0.35$)

Supplemental Figure 3.44. The effect of MMS vs. IFA on Birthweight, stratified by Total Pill Count

Study	Total MMS	MMS Mean (SD)	Total IFA	IFA Mean (SD)	MD	95%-CI	Weight (Fixed)	Weight (random)
<90 total pills								
Osrin 2005	6	2430 (816)	5	2830 (645)	-400.00	[-1420.00; 620.00]	0.0%	0.0%
Ashorn 2015	34	2713 (624)	33	2772 (484)	-59.00	[-333.00; 214.00]	0.1%	0.1%
Zagre 2007	29	3141 (759)	28	3164 (487)	-22.91	[-398.14; 352.33]	0.1%	0.1%
Sunawang 2009	179	3037 (477)	169	3054 (427)	-16.97	[-85.99; 52.05]	1.5%	1.5%
Christian 2003	143	2537 (544)	125	2535 (480)	1.81	[-136.53; 140.15]	0.4%	0.4%
Persson 2012	237	2638 (422)	436	2631 (444)	8.00	[-62.00; 77.00]	1.5%	1.5%
Kaestel 2005	403	3043 (493)	184	3015 (468)	28.00	[-57.00; 113.00]	1.0%	1.0%
West 2014	631	2549 (430)	599	2508 (432)	40.95	[-8.45; 90.34]	3.0%	3.0%
Adu-Afarwuah 2015	59	2805 (557)	48	2761 (511)	44.00	[-163.00; 250.00]	0.2%	0.2%
Fawzi 2007	893	3036 (549)	897	2977 (586)	59.00	[6.00; 111.00]	2.6%	2.6%
Roberfroid 2008	125	2854 (512)	126	2787 (489)	67.00	[-58.00; 191.00]	0.5%	0.5%
Bliznashka 2022	77	2999 (525)	71	2894 (551)	104.21	[-139.66; 348.07]	0.1%	0.1%
Bhutta 2009	66	3083 (661)	37	2919 (605)	163.99	[4.45; 323.53]	0.3%	0.3%
Fixed Effect Model					33.94	[8.51; 59.38]	11.2%	--
Random Effects Model					33.94	[8.51; 59.38]	--	11.2%
Heterogeneity: $I^2 = 0\%$, $\tau^2 = 0$, $p = 0.77$								
90-120 total pills								
Osrin 2005	35	2499 (538)	32	2546 (567)	-48.00	[-317.00; 222.00]	0.1%	0.1%
Persson 2012	106	2656 (380)	230	2674 (441)	-18.00	[-115.00; 80.00]	0.8%	0.8%
Sunawang 2009	157	3061 (486)	149	3074 (484)	-13.35	[-72.23; 45.53]	2.1%	2.1%
Ashorn 2015	77	2872 (483)	80	2866 (518)	7.00	[-151.00; 165.00]	0.3%	0.3%
Roberfroid 2008	101	2910 (436)	104	2874 (433)	37.00	[-83.00; 156.00]	0.5%	0.5%
Zagre 2007	81	2953 (685)	69	2901 (751)	51.64	[-263.71; 366.99]	0.1%	0.1%
Bliznashka 2022	134	3077 (453)	117	3020 (444)	57.64	[-100.50; 215.79]	0.3%	0.3%
West 2014	412	2500 (455)	423	2435 (472)	65.85	[4.95; 126.74]	2.0%	2.0%
Fawzi 2007	1106	3167 (471)	1166	3089 (483)	78.00	[39.00; 117.00]	4.8%	4.8%
Christian 2003	79	2605 (453)	77	2522 (461)	82.27	[-70.44; 234.98]	0.3%	0.3%
Kaestel 2005	178	3093 (506)	95	2971 (441)	122.00	[1.00; 243.00]	0.5%	0.5%
Bhutta 2009	80	3030 (631)	81	2907 (598)	123.46	[-72.30; 319.21]	0.2%	0.2%
Adu-Afarwuah 2015	101	3009 (390)	107	2883 (477)	126.00	[6.00; 246.00]	0.5%	0.5%
Fixed Effect Model					54.17	[29.93; 78.41]	12.3%	--
Random Effects Model					52.73	[25.91; 79.54]	--	12.3%
Heterogeneity: $I^2 = 7\%$, $\tau^2 = 175.7076$, $p = 0.38$								
120-180 pills								
Persson 2012	330	2647 (420)	595	2646 (416)	2.00	[-55.00; 58.00]	2.3%	2.3%
Sunawang 2009	372	3102 (416)	367	3094 (408)	8.01	[-62.16; 78.18]	1.5%	1.5%
Ashorn 2015	240	3033 (416)	252	3004 (385)	29.00	[-42.00; 100.00]	1.4%	1.4%
Adu-Afarwuah 2015	209	3053 (366)	207	3013 (408)	39.00	[-35.00; 114.00]	1.3%	1.3%
Christian 2003	267	2662 (428)	250	2616 (460)	46.06	[-38.42; 130.54]	1.0%	1.0%
West 2014	2666	2486 (427)	2784	2433 (437)	52.74	[28.67; 76.81]	12.5%	12.5%
Bliznashka 2022	380	3111 (481)	424	3056 (390)	55.09	[-88.63; 198.82]	0.4%	0.4%
Zagre 2007	686	3056 (554)	564	3000 (565)	55.89	[-50.90; 162.69]	0.6%	0.6%
Roberfroid 2008	163	2927 (429)	167	2871 (411)	56.00	[-35.00; 147.00]	0.9%	0.9%
Fawzi 2007	1611	3239 (474)	1517	3172 (465)	67.00	[34.00; 100.00]	6.7%	6.7%
Osrin 2005	414	2818 (440)	436	2728 (412)	89.00	[32.00; 147.00]	2.2%	2.2%
Bhutta 2009	366	2958 (636)	480	2851 (530)	107.02	[-9.17; 223.22]	0.5%	0.5%
Kaestel 2005	120	3172 (457)	70	3055 (472)	118.00	[-19.00; 255.00]	0.4%	0.4%
Fixed Effect Model					52.57	[37.43; 67.70]	31.6%	--
Random Effects Model					52.57	[37.43; 67.70]	--	31.6%
Heterogeneity: $I^2 = 0\%$, $\tau^2 = 0$, $p = 0.69$								
≥180 total pills								
Kaestel 2005	14	3277 (511)	5	3394 (328)	-117.00	[-638.00; 405.00]	0.0%	0.0%
Roberfroid 2008	125	2959 (441)	119	2995 (344)	-37.00	[-137.00; 63.00]	0.7%	0.7%
Adu-Afarwuah 2015	14	2913 (362)	18	2941 (361)	-28.00	[-291.00; 235.00]	0.1%	0.1%
Christian 2003	365	2728 (446)	299	2732 (399)	-3.61	[-66.28; 59.07]	1.8%	1.8%
Sunawang 2009	71	3155 (381)	72	3146 (351)	9.10	[-39.79; 57.99]	3.0%	3.0%
Bhutta 2009	177	2993 (510)	193	2978 (574)	14.76	[-99.32; 128.83]	0.6%	0.6%
Persson 2012	286	2761 (419)	661	2745 (399)	15.00	[-41.00; 72.00]	2.3%	2.3%
West 2014	6930	2631 (384)	6720	2580 (391)	50.95	[36.42; 65.49]	34.3%	34.3%
Ashorn 2015	26	3012 (463)	17	2951 (461)	61.00	[-230.00; 352.00]	0.1%	0.1%
Zagre 2007	661	3111 (519)	666	3049 (433)	62.55	[-30.89; 155.98]	0.8%	0.8%
Osrin 2005	94	2938 (381)	71	2842 (362)	96.00	[-19.00; 212.00]	0.5%	0.5%
Bliznashka 2022	205	3184 (483)	262	3049 (454)	134.67	[-50.58; 319.93]	0.2%	0.2%
Fawzi 2007	77	3337 (493)	73	3190 (452)	147.00	[-6.00; 300.00]	0.3%	0.3%
Fixed Effect Model					43.74	[31.03; 56.45]	44.8%	--
Random Effects Model					37.29	[18.57; 56.01]	--	44.8%
Heterogeneity: $I^2 = 8\%$, $\tau^2 = 113.7279$, $p = 0.36$								
**Fixed Effect Model**					**46.72**	**[38.21; 55.23]**	**100.0%**	**--**
**Random Effects Model**					**46.72**	**[38.21; 55.23]**	**--**	**100.0%**

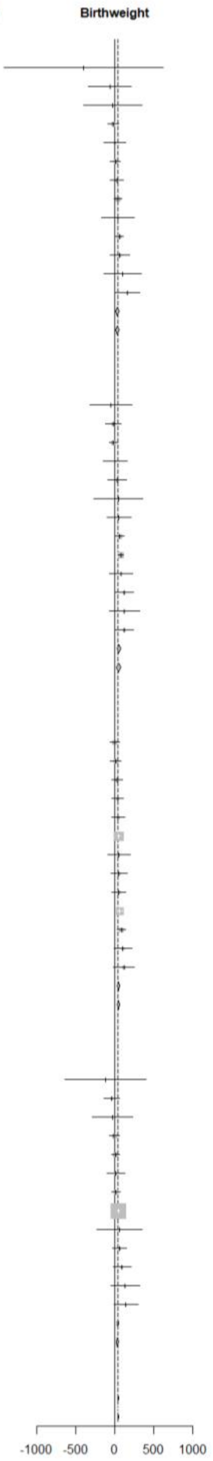

Heterogeneity: $I^2 = 0\%$, $\tau^2 = 0$, $p = 0.69$
Test of subgroup differences (fixed effect): $\chi^2_3 = 2.12$, df = 3 ($p = 0.55$)
Test for subgroup differences (random effects): $\chi^2_3 = 2.65$, df = 3 ($p = 0.45$)

Supplemental Figure 3.45. The effect of MMS vs. IFA on Continuous Gestation, stratified by Total Pill Count

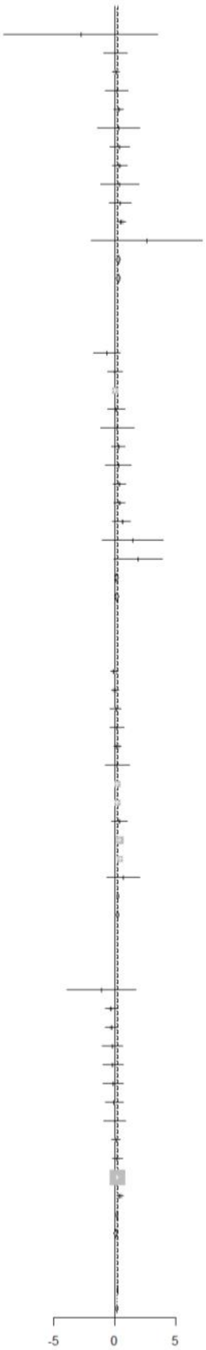

Study	Total MMS	MMS Mean (SD)	Total IFA	IFA Mean (SD)	MD	95%-CI	Weight (Fixed)	Weight (random)
**<90 total pills**								
Osrin 2005	6	35.7 (6)	5	38.5 (1.7)	-2.80	[-9.10; 3.50]	0.0%	0.0%
Roberfroid 2008	149	37.9 (4.2)	150	37.9 (4.2)	0.00	[-0.90; 1.00]	0.2%	0.4%
Fawzi 2007	916	37.6 (3.3)	930	37.5 (3.5)	0.10	[-0.20; 0.40]	2.4%	3.5%
Adu-Afarwuah 2015	65	38.6 (2.7)	57	38.5 (2.6)	0.20	[-0.80; 1.10]	0.2%	0.4%
Persson 2012	235	39.3 (2.4)	428	39 (2.3)	0.30	[-0.10; 0.70]	1.3%	2.1%
Bhutta 2009	67	36.3 (4.8)	37	36 (4.3)	0.32	[-1.40; 2.03]	0.1%	0.1%
Sunawang 2009	179	37.3 (3.6)	170	36.9 (3.6)	0.38	[-0.43; 1.18]	0.3%	0.6%
Kaestel 2005	590	38.8 (4.2)	266	38.4 (4.2)	0.40	[-0.20; 1.00]	0.6%	1.0%
Ashorn 2015	48	37.3 (3.5)	45	36.9 (4.2)	0.40	[-1.20; 2.00]	0.1%	0.1%
Christian 2003	143	38.6 (3.6)	124	38.2 (3.9)	0.43	[-0.49; 1.34]	0.3%	0.4%
West 2014	981	38.19 (3.74)	910	37.66 (3.80)	0.53	[0.18; 0.87]	1.8%	2.7%
Zagre 2007	29	26 (6.7)	28	23.4 (5.3)	2.62	[-1.91; 7.15]	0.0%	0.0%
Fixed Effect Model					0.30	[0.13; 0.47]	7.4%	--
Random Effects Model					0.30	[0.13; 0.47]	--	11.4%
Heterogeneity: $I^2 = 0\%$, $\tau^2 = 0$, $p = 0.88$								
**90-120 total pills**								
Sunawang 2009	157	36.8 (3.5)	149	37.5 (3.5)	-0.64	[-1.75; 0.46]	0.2%	0.3%
Ashorn 2015	93	38.5 (2.1)	92	38.5 (2.3)	0.00	[-0.60; 0.60]	0.6%	1.0%
Fawzi 2007	1145	39.3 (2.6)	1205	39.3 (2.5)	0.00	[-0.20; 0.20]	5.4%	6.5%
Roberfroid 2008	122	39.3 (3.2)	127	39.1 (2.3)	0.10	[-0.60; 0.80]	0.4%	0.7%
Osrin 2005	35	37.4 (2.9)	32	37.2 (2.8)	0.20	[-1.20; 1.60]	0.1%	0.2%
Persson 2012	100	39.4 (2.2)	226	39.1 (2.5)	0.30	[-0.30; 0.80]	0.7%	1.2%
Christian 2003	78	37.6 (3.8)	78	37.3 (3.3)	0.30	[-0.76; 1.36]	0.2%	0.3%
West 2014	599	36.87 (4.42)	632	36.51 (4.78)	0.37	[-0.16; 0.90]	0.8%	1.3%
Adu-Afarwuah 2015	105	39.1 (1.3)	109	38.7 (2.2)	0.40	[-0.10; 0.80]	1.1%	1.7%
Kaestel 2005	215	40.2 (3.3)	109	39.6 (3.3)	0.60	[-0.20; 1.30]	0.4%	0.6%
Bhutta 2009	84	36.3 (4.6)	82	34.8 (5.4)	1.46	[-1.01; 3.94]	0.0%	0.1%
Zagre 2007	81	30.1 (4.2)	69	28.2 (4.4)	1.89	[-0.10; 3.89]	0.1%	0.1%
Fixed Effect Model					0.13	[-0.01; 0.28]	9.9%	--
Random Effects Model					0.17	[-0.01; 0.34]	--	13.9%
Heterogeneity: $I^2 = 8\%$, $\tau^2 = 0.0078$, $p = 0.37$								
**120-180 pills**								
Sunawang 2009	371	38.7 (2.5)	367	38.8 (2.4)	-0.06	[-0.36; 0.25]	2.3%	3.4%
Persson 2012	323	39.1 (2.1)	586	39.1 (2.1)	0.00	[-0.30; 0.30]	2.4%	3.5%
Roberfroid 2008	194	39.9 (2.3)	195	39.9 (2.3)	0.10	[-0.40; 0.50]	1.1%	1.7%
Christian 2003	268	38.5 (3.4)	253	38.3 (3.4)	0.17	[-0.41; 0.75]	0.6%	1.1%
Adu-Afarwuah 2015	215	39.7 (1.5)	211	39.5 (1.5)	0.20	[-0.10; 0.50]	2.4%	3.5%
Kaestel 2005	149	40.1 (3.3)	83	39.9 (4)	0.20	[-0.80; 1.20]	0.2%	0.4%
Ashorn 2015	257	39.8 (1.4)	265	39.7 (1.4)	0.20	[0.00; 0.40]	5.4%	6.5%
Osrin 2005	414	39.5 (1.5)	436	39.3 (1.7)	0.20	[0.00; 0.40]	5.4%	6.5%
Zagre 2007	686	35 (3.3)	564	34.7 (3.2)	0.36	[-0.29; 1.01]	0.5%	0.9%
Fawzi 2007	1666	40.8 (2.4)	1547	40.4 (2.4)	0.40	[0.20; 0.50]	9.6%	9.3%
West 2014	3628	37.80 (3.41)	3708	37.40 (3.62)	0.41	[0.22; 0.60]	5.9%	6.8%
Bhutta 2009	372	36 (4)	479	35.3 (4)	0.68	[-0.67; 2.04]	0.1%	0.2%
Fixed Effect Model					0.26	[0.18; 0.33]	35.8%	--
Random Effects Model					0.24	[0.14; 0.33]	--	43.5%
Heterogeneity: $I^2 = 25\%$, $\tau^2 = 0.0067$, $p = 0.20$								
**≥180 total pills**								
Kaestel 2005	17	41.4 (3.5)	8	42.5 (2.3)	-1.10	[-3.90; 1.70]	0.0%	0.0%
Sunawang 2009	71	40.7 (1.8)	72	41.1 (1.9)	-0.34	[-0.77; 0.10]	1.1%	1.8%
Roberfroid 2008	138	39.6 (1.7)	138	39.9 (1.9)	-0.30	[-0.80; 0.10]	1.1%	1.7%
Zagre 2007	661	39.7 (4)	666	39.9 (4.8)	-0.21	[-1.04; 0.62]	0.3%	0.5%
Fawzi 2007	79	42.3 (2.7)	79	42.5 (2.7)	-0.20	[-1.00; 0.70]	0.3%	0.5%
Bhutta 2009	179	37.2 (3.3)	194	37.4 (3.2)	-0.15	[-0.96; 0.66]	0.3%	0.6%
Ashorn 2015	27	41.6 (1.3)	20	41.6 (1.1)	-0.10	[-0.80; 0.70]	0.4%	0.6%
Adu-Afarwuah 2015	14	40.2 (1.2)	18	40.2 (1.3)	0.00	[-0.90; 0.90]	0.3%	0.5%
Christian 2003	365	40.1 (2.3)	299	40 (2.5)	0.09	[-0.29; 0.46]	1.5%	2.4%
Osrin 2005	94	40.6 (1.3)	71	40.4 (1.2)	0.20	[-0.20; 0.60]	1.3%	2.1%
West 2014	8259	39.59 (2.16)	8076	39.38 (2.12)	0.20	[0.13; 0.28]	36.8%	15.7%
Persson 2012	282	40.1 (2.2)	656	39.7 (1.8)	0.40	[0.20; 0.70]	3.4%	4.7%
Fixed Effect Model					0.18	[0.11; 0.25]	46.9%	--
Random Effects Model					0.08	[-0.08; 0.23]	--	31.1%
Heterogeneity: $I^2 = 36\%$, $\tau^2 = 0.0196$, $p = 0.10$								
**Fixed Effect Model**					**0.21**	**[0.16; 0.26]**	**100.0%**	**--**
**Random Effects Model**					**0.20**	**[0.14; 0.26]**	**--**	**100.0%**

Heterogeneity: $I^2 = 13\%$, $\tau^2 = 0.0047$, $p = 0.22$
Test of subgroup differences (fixed effect): $\chi^2_3 = 4.33$, df = 3 ($p = 0.23$)
Test for subgroup differences (random effects): $\chi^2_3 = 4.31$, df = 3 ($p = 0.23$)

Supplemental Figure 3.46. The effect of MMS vs. IFA on Fetal Death, stratified by Total Pill Count

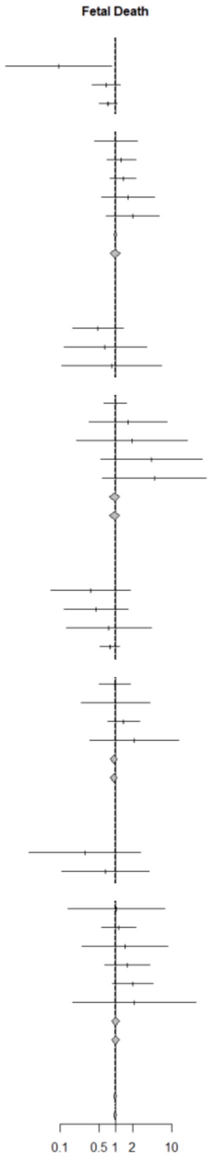

Study	Events/Total (MMS)	Events/Total (IFA)	RR	95%-CI	Weight (Fixed)	Weight (random)
**<90 total pills**						
Bhutta 2009	0/82	3/47	0.09	[0.01; 0.85]	0.0%	0.0%
Bliznashka 2022	23/116	35/119	0.67	[0.38; 1.21]	0.5%	0.5%
Fawzi 2007	43/1001	62/1048	0.73	[0.50; 1.06]	1.3%	1.3%
West 2014						
Moore 2012	2/2	2/2	1.00	[0.42; 2.40]	0.2%	0.2%
Roberfroid 2008	22/206	18/211	1.25	[0.69; 2.26]	0.5%	0.5%
Sunawang 2009	16/204	11/191	1.36	[0.80; 2.30]	0.6%	0.6%
Christian 2003	9/155	5/141	1.64	[0.55; 4.87]	0.1%	0.1%
Zagre 2007	7/36	3/31	2.01	[0.68; 5.95]	0.2%	0.2%
Fixed Effect Model			0.98	[0.93; 1.03]	80.8%	--
Random Effects Model			0.98	[0.78; 1.21]	--	80.8%
Heterogeneity: $I^2 = 39\%$, $\tau^2 = 0.0341$, $p = 0.11$						
**90-120 total pills**						
Sunawang 2009	2/164	4/158	0.48	[0.17; 1.38]	0.2%	0.2%
Christian 2003	2/84	3/81	0.64	[0.12; 3.54]	0.1%	0.1%
Moore 2012	1/6	0/2	0.83	[0.11; 6.51]	0.0%	0.0%
West 2014						
Fawzi 2007	34/1223	37/1298	0.98	[0.62; 1.54]	0.9%	0.9%
Zagre 2007	6/87	3/72	1.66	[0.33; 8.20]	0.1%	0.1%
Bhutta 2009	2/93	1/91	1.96	[0.20; 19.04]	0.0%	0.0%
Bliznashka 2022	5/157	1/137	4.36	[0.54; 35.15]	0.0%	0.0%
Roberfroid 2008	5/131	1/129	4.92	[0.58; 41.57]	0.0%	0.0%
Fixed Effect Model			0.95	[0.78; 1.15]	4.6%	--
Random Effects Model			0.95	[0.78; 1.15]	--	4.6%
Heterogeneity: $I^2 = 0\%$, $\tau^2 = 0$, $p = 0.52$						
**120-180 pills**						
Moore 2012	2/122	5/109	0.36	[0.07; 1.80]	0.1%	0.1%
Roberfroid 2008	3/205	7/210	0.44	[0.12; 1.67]	0.1%	0.1%
Sunawang 2009	3/378	4/380	0.75	[0.13; 4.30]	0.1%	0.1%
Fawzi 2007	42/1746	50/1634	0.79	[0.52; 1.18]	1.1%	1.1%
West 2014						
Zagre 2007	22/708	19/583	0.95	[0.50; 1.83]	0.4%	0.4%
Bhutta 2009	6/391	8/510	0.98	[0.24; 4.04]	0.1%	0.1%
Christian 2003	18/294	12/269	1.37	[0.71; 2.64]	0.4%	0.4%
Bliznashka 2022	2/439	1/465	2.12	[0.34; 13.20]	0.1%	0.1%
Fixed Effect Model			0.92	[0.79; 1.07]	7.4%	--
Random Effects Model			0.92	[0.79; 1.07]	--	7.4%
Heterogeneity: $I^2 = 0\%$, $\tau^2 = 0$, $p = 0.72$						
**≥180 total pills**						
Moore 2012	0/84	2/95	0.28	[0.03; 2.80]	0.0%	0.0%
Roberfroid 2008	2/141	3/140	0.66	[0.11; 3.90]	0.1%	0.1%
West 2014						
Sunawang 2009	2/73	2/74	1.01	[0.14; 7.53]	0.0%	0.0%
Zagre 2007	20/681	18/684	1.12	[0.55; 2.25]	0.4%	0.4%
Fawzi 2007	3/84	2/83	1.48	[0.25; 8.64]	0.1%	0.1%
Christian 2003	18/386	9/311	1.61	[0.64; 4.07]	0.2%	0.2%
Bliznashka 2022	8/243	5/301	1.98	[0.86; 4.58]	0.3%	0.3%
Bhutta 2009	1/186	0/199	2.15	[0.17; 26.73]	0.0%	0.0%
Fixed Effect Model			0.98	[0.84; 1.15]	7.2%	--
Random Effects Model			0.98	[0.84; 1.15]	--	7.2%
Heterogeneity: $I^2 = 0\%$, $\tau^2 = 0$, $p = 0.63$						
**Fixed Effect Model**			**0.97**	**[0.93; 1.01]**	**100.0%**	**--**
**Random Effects Model**			**0.97**	**[0.93; 1.01]**	**--**	**100.0%**

Heterogeneity: $I^2 = 0\%$, $\tau^2 = 0$, $p = 0.60$
Test of subgroup differences (fixed effect): $\chi^2_3 = 0.69$, df = 3 ($p = 0.87$)
Test for subgroup differences (random effects): $\chi^2_3 = 0.41$, df = 3 ($p = 0.94$)

Supplemental Figure 3.47. The effect of MMS vs. IFA on Hemoglobin Continuous, stratified by Total Pill Count

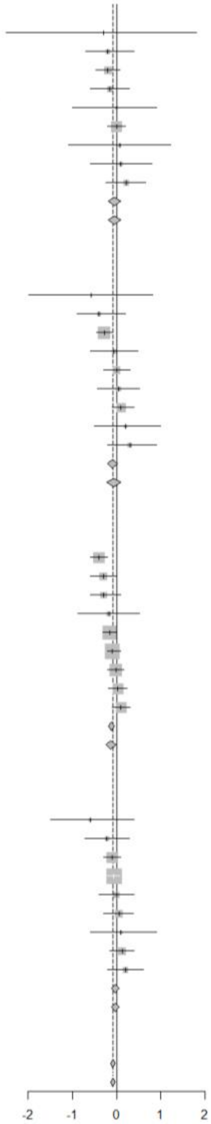

Study	Total MMS	MMS Mean (SD)	Total IFA	IFA Mean (SD)	3rd Trimester Hemoglobin	MD	95%-CI	Weight (Fixed)	Weight (random)
<90 total pills									
Osrin 2005	2	11.6 (1.1)	4	11.9 (0.8)		-0.30	[-2.50; 1.80]	0.0%	0.1%
Adu-Afarwuah 2015	42	11.5 (1.1)	36	11.7 (1.4)		-0.20	[-0.70; 0.40]	0.7%	0.9%
Sunawang 2009	179	10.8 (1.3)	162	11 (1.3)		-0.20	[-0.48; 0.08]	2.8%	3.2%
West 2014	30	11.29 (0.92)	24	11.44 (0.96)		-0.15	[-0.59; 0.29]	1.1%	1.4%
Ashorn 2015	24	11.2 (1.6)	19	11.2 (1.5)		0.00	[-1.00; 0.90]	0.2%	0.3%
Persson 2012	232	11.3 (1.2)	422	11.3 (1.3)		0.00	[-0.20; 0.20]	5.5%	5.5%
Christian 2003	15	10.8 (1.5)	8	10.7 (1.4)		0.07	[-1.08; 1.23]	0.2%	0.2%
Roberfroid 2008	54	11 (2)	52	11 (1.5)		0.10	[-0.60; 0.80]	0.5%	0.6%
Bliznashka 2022	34	11.2 (1.1)	37	11 (1.1)		0.21	[-0.24; 0.65]	1.1%	1.4%
Fixed Effect Model						-0.05	[-0.18; 0.08]	12.2%	--
Random Effects Model						-0.05	[-0.18; 0.08]	--	13.6%
Heterogeneity: $I^2 = 0\%$, $\tau^2 = 0$, $p = 0.90$									
90-120 total pills									
Christian 2003	11	11.4 (1.8)	9	12 (1.7)		-0.58	[-1.98; 0.82]	0.1%	0.1%
Ashorn 2015	72	11 (1.5)	67	11.4 (1.6)		-0.40	[-0.90; 0.20]	0.7%	0.9%
Sunawang 2009	153	10.9 (1.3)	146	11.2 (1.1)		-0.28	[-0.46; -0.10]	6.8%	6.4%
Bliznashka 2022	84	11.2 (1.6)	87	11.2 (1.5)		-0.06	[-0.59; 0.48]	0.8%	1.0%
Adu-Afarwuah 2015	96	11.7 (1.1)	87	11.7 (1)		0.00	[-0.30; 0.30]	2.5%	2.8%
West 2014	29	11.06 (1.31)	47	11.03 (1.05)		0.04	[-0.44; 0.52]	1.0%	1.2%
Persson 2012	104	11.6 (1.3)	223	11.4 (1.1)		0.10	[-0.10; 0.40]	3.5%	3.9%
Osrin 2005	34	12 (1.4)	30	11.8 (1.6)		0.20	[-0.50; 1.00]	0.4%	0.5%
Roberfroid 2008	72	11 (1.7)	76	10.7 (1.7)		0.30	[-0.20; 0.90]	0.7%	0.9%
Fixed Effect Model						-0.10	[-0.21; 0.02]	16.6%	--
Random Effects Model						-0.06	[-0.22; 0.10]	--	17.8%
Heterogeneity: $I^2 = 29\%$, $\tau^2 = 0.0154$, $p = 0.18$									
120-180 pills									
Adu-Afarwuah 2015	209	11.7 (1.2)	208	12 (1.1)		-0.40	[-0.60; -0.20]	5.5%	5.5%
Ashorn 2015	228	10.9 (1.4)	237	11.2 (1.5)		-0.30	[-0.60; 0.00]	2.5%	2.8%
Roberfroid 2008	138	10.9 (1.7)	143	11.1 (1.5)		-0.30	[-0.60; 0.10]	1.8%	2.1%
Christian 2003	57	11.4 (1.9)	44	11.6 (1.5)		-0.18	[-0.88; 0.51]	0.5%	0.6%
Sunawang 2009	376	11 (1.2)	376	11.2 (1.2)		-0.16	[-0.32; 0.00]	8.6%	7.6%
Osrin 2005	407	11.8 (1.2)	408	11.8 (1.4)		-0.10	[-0.20; 0.10]	9.8%	8.2%
West 2014	194	11.20 (1.00)	230	11.22 (1.10)		-0.02	[-0.20; 0.17]	6.5%	6.2%
Bliznashka 2022	317	11.4 (1.2)	327	11.3 (1.2)		0.03	[-0.19; 0.24]	4.8%	4.9%
Persson 2012	320	11.6 (1.3)	572	11.5 (1.2)		0.10	[-0.10; 0.30]	5.5%	5.5%
Fixed Effect Model						-0.12	[-0.19; -0.05]	45.6%	--
Random Effects Model						-0.13	[-0.24; -0.02]	--	43.5%
Heterogeneity: $I^2 = 55\%$, $\tau^2 = 0.0148$, $p = 0.02$									
≥180 total pills									
Adu-Afarwuah 2015	14	11.5 (1.4)	17	12.1 (1.2)		-0.60	[-1.50; 0.40]	0.2%	0.3%
Christian 2003	84	11.6 (1.5)	69	11.8 (1.4)		-0.22	[-0.72; 0.28]	0.9%	1.1%
Persson 2012	282	11.5 (1.1)	642	11.6 (1.2)		-0.10	[-0.30; 0.10]	5.5%	5.5%
West 2014	521	11.00 (1.05)	515	11.06 (0.99)		-0.06	[-0.20; 0.08]	11.1%	8.9%
Roberfroid 2008	107	10.9 (1.7)	102	10.9 (1.5)		0.00	[-0.40; 0.40]	1.4%	1.7%
Sunawang 2009	73	10.9 (1.1)	73	10.9 (0.9)		0.05	[-0.29; 0.38]	2.0%	2.3%
Ashorn 2015	27	11.3 (1.3)	20	11.2 (1.2)		0.10	[-0.60; 0.90]	0.4%	0.5%
Bliznashka 2022	214	11.6 (1.4)	261	11.5 (1.1)		0.12	[-0.16; 0.40]	2.8%	3.2%
Osrin 2005	90	11.8 (1.3)	72	11.6 (1.3)		0.20	[-0.20; 0.60]	1.4%	1.7%
Fixed Effect Model						-0.03	[-0.12; 0.06]	25.7%	--
Random Effects Model						-0.03	[-0.12; 0.06]	--	25.2%
Heterogeneity: $I^2 = 0\%$, $\tau^2 = 0$, $p = 0.72$									
**Fixed Effect Model**						**-0.08**	**[-0.13; -0.04]**	**100.0%**	**--**
**Random Effects Model**						**-0.08**	**[-0.13; -0.03]**	**--**	**100.0%**

Heterogeneity: $I^2 = 13\%$, $\tau^2 = 0.0033$, $p = 0.24$
Test of subgroup differences (fixed effect): $\chi^2_3 = 2.42$, df = 3 ($p = 0.49$)
Test for subgroup differences (random effects): $\chi^2_3 = 1.75$, df = 3 ($p = 0.63$)

Supplemental Figure 3.48. The effect of MMS vs. IFA on IDA, stratified by Total Pill Count

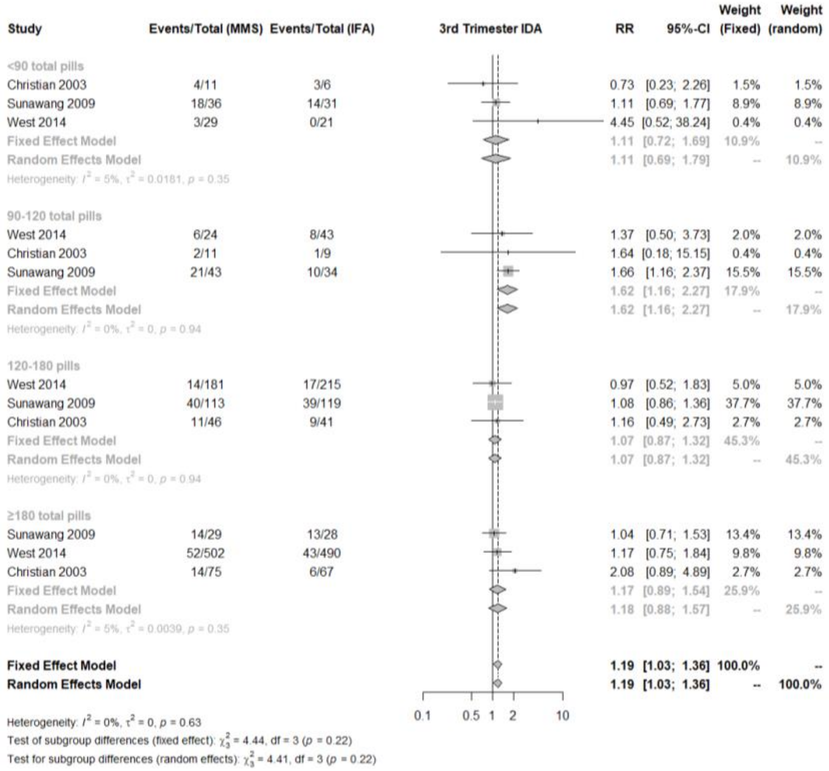

Supplemental Figure 3.49. The effect of MMS vs. IFA on Infant Mortality, stratified by Total Pill Count

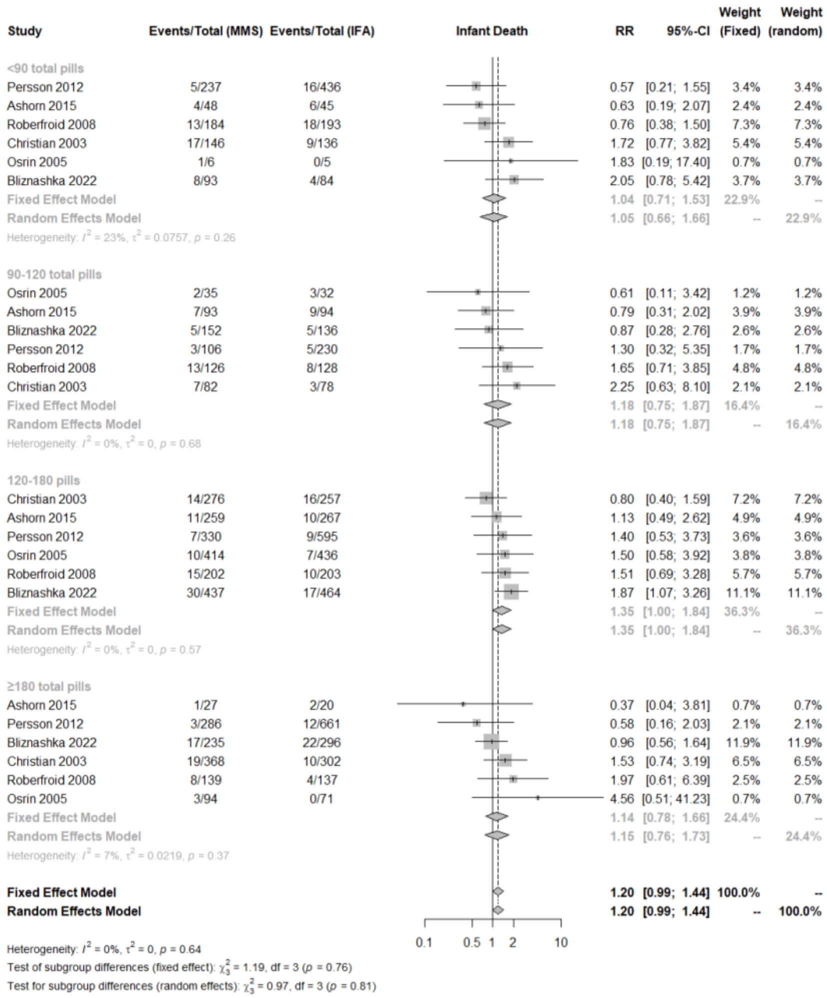

Supplemental Figure 3.50. The effect of MMS vs. IFA on LGA90, stratified by Total Pill Count

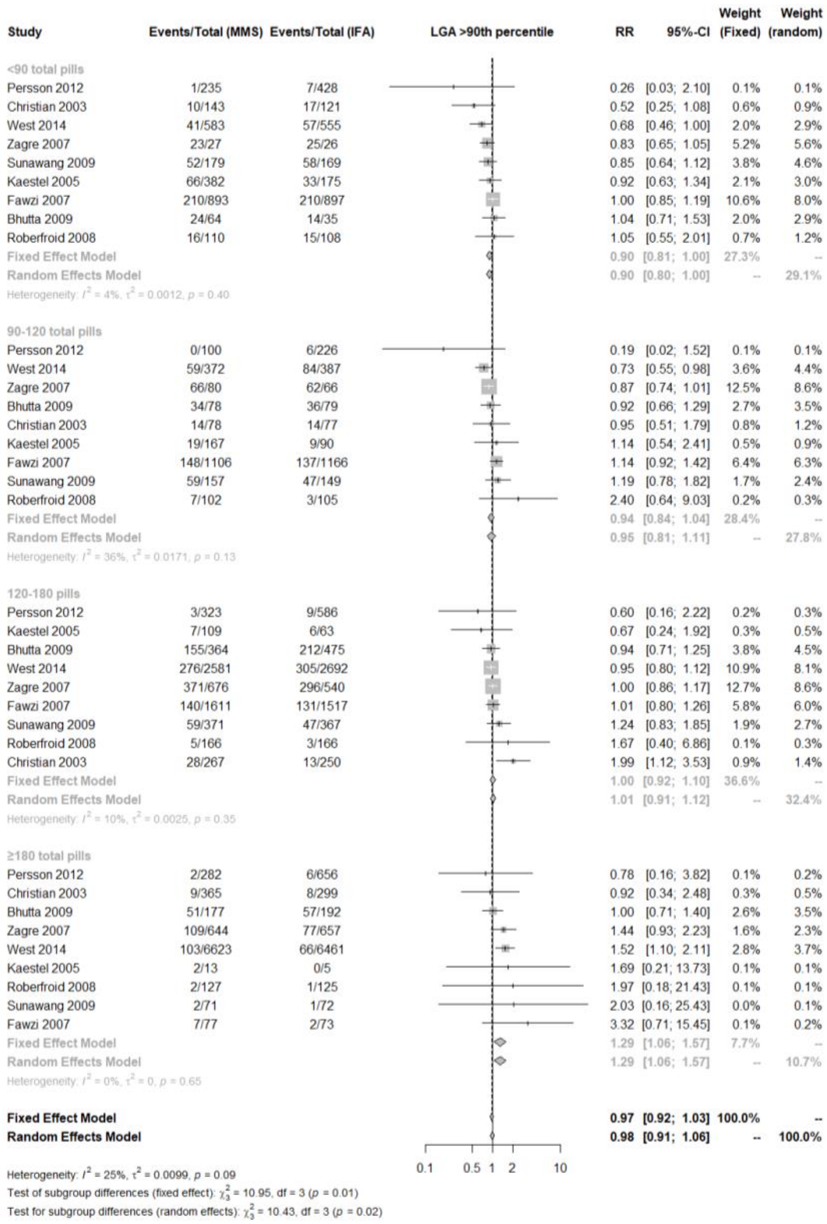

Supplemental Figure 3.51. The effect of MMS vs. IFA on Low Birthweight, stratified by Total Pill Count

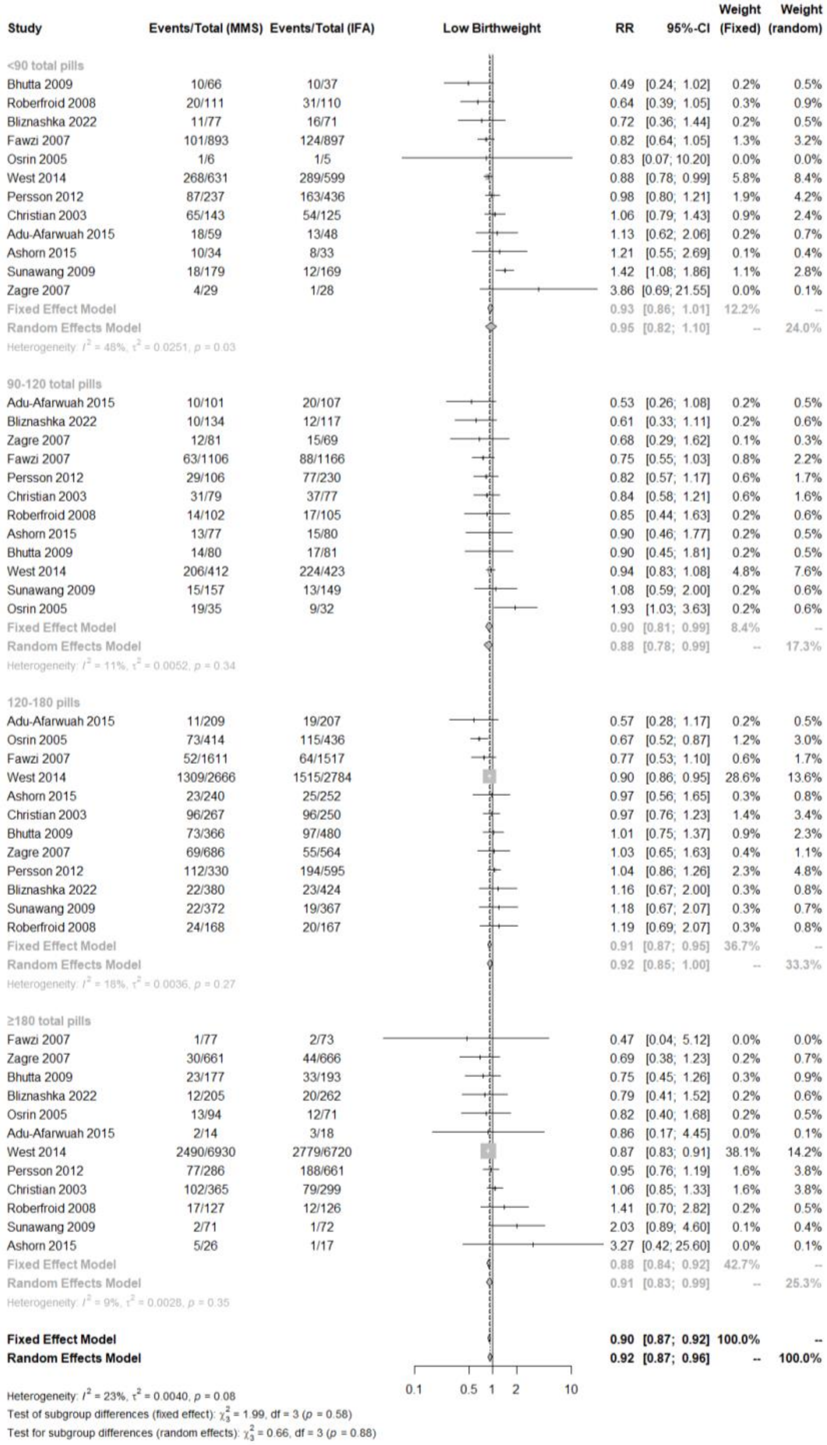

Supplemental Figure 3.52. The effect of MMS vs. IFA on Neonatal Mortality, stratified by Total Pill Count

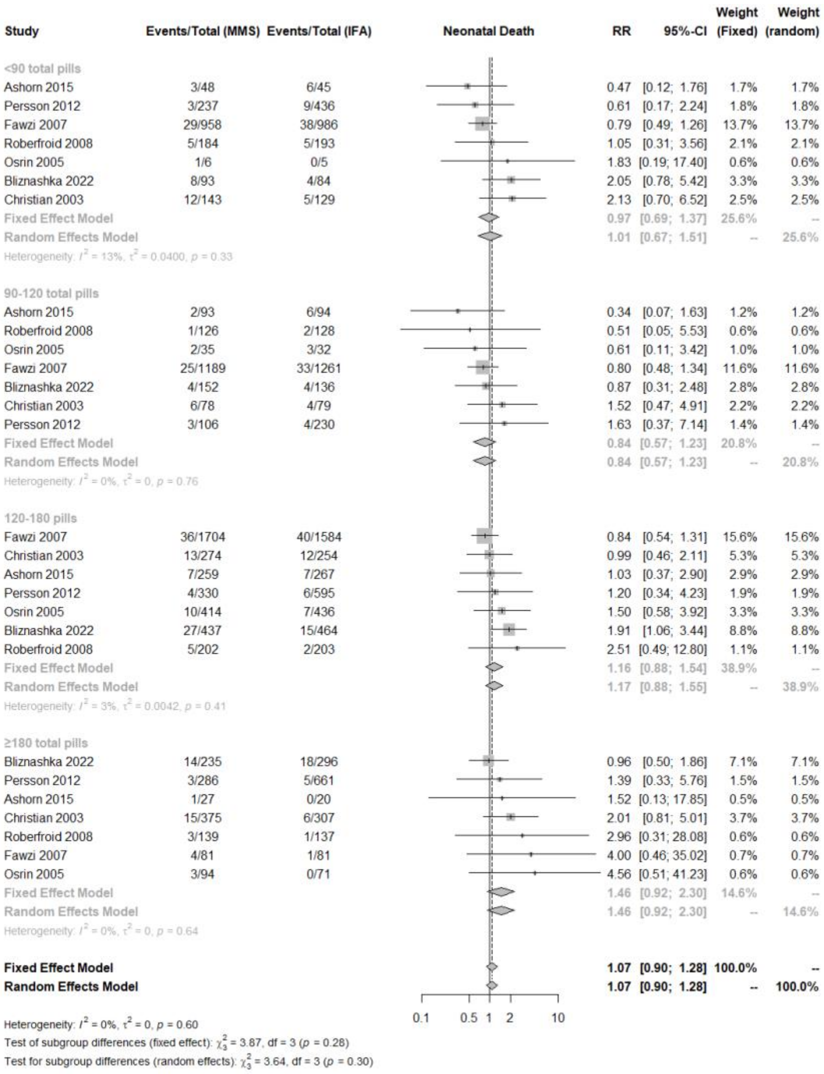

Supplemental Figure 3.53. The effect of MMS vs. IFA on Preterm, stratified by Total Pill Count

Study	Events/Total (MMS)	Events/Total (IFA)	RR	95%-CI	Weight (Fixed)	Weight (random)
<90 total pills						
Persson 2012	30/235	72/428	0.76	[0.51; 1.13]	0.8%	0.8%
West 2014	256/981	304/910	0.78	[0.68; 0.90]	6.5%	6.5%
Zagre 2007	25/29	28/28	0.89	[0.77; 1.03]	6.0%	6.0%
Kaestel 2005	168/590	80/266	0.95	[0.76; 1.18]	2.6%	2.6%
Sunawang 2009	75/179	75/170	0.95	[0.73; 1.24]	1.8%	1.8%
Roberfroid 2008	47/149	47/150	1.01	[0.72; 1.41]	1.1%	1.1%
Christian 2003	41/143	37/124	1.02	[0.69; 1.51]	0.8%	0.8%
Bhutta 2009	34/67	19/37	1.04	[0.69; 1.57]	0.7%	0.7%
Fixed Effect Model			0.88	[0.81; 0.95]	20.3%	--
Random Effects Model			0.88	[0.81; 0.95]	--	20.3%
Heterogeneity: $I^2 = 0\%$, $\tau^2 = 0$, $p = 0.53$						
90-120 total pills						
Persson 2012	9/100	33/226	0.62	[0.31; 1.24]	0.3%	0.3%
Bhutta 2009	44/84	48/82	0.84	[0.59; 1.20]	1.0%	1.0%
Kaestel 2005	32/215	19/109	0.85	[0.51; 1.43]	0.5%	0.5%
West 2014	230/599	268/632	0.90	[0.79; 1.04]	6.3%	6.3%
Zagre 2007	75/81	64/69	1.00	[0.91; 1.10]	13.8%	13.8%
Christian 2003	31/78	31/78	1.04	[0.73; 1.47]	1.0%	1.0%
Roberfroid 2008	22/122	20/127	1.15	[0.66; 1.99]	0.4%	0.4%
Sunawang 2009	76/157	57/149	1.24	[0.89; 1.74]	1.1%	1.1%
Fixed Effect Model			0.97	[0.91; 1.05]	24.4%	--
Random Effects Model			0.97	[0.91; 1.05]	--	24.4%
Heterogeneity: $I^2 = 0\%$, $\tau^2 = 0$, $p = 0.49$						
120-180 pills						
Roberfroid 2008	6/194	10/195	0.60	[0.22; 1.63]	0.1%	0.1%
Kaestel 2005	20/149	14/83	0.80	[0.42; 1.49]	0.3%	0.3%
Persson 2012	37/323	83/586	0.81	[0.56; 1.16]	0.9%	0.9%
West 2014	1180/3628	1408/3708	0.86	[0.79; 0.92]	22.8%	22.8%
Zagre 2007	473/686	429/564	0.90	[0.81; 1.01]	10.2%	10.2%
Christian 2003	75/268	81/253	0.91	[0.71; 1.18]	1.9%	1.9%
Bhutta 2009	221/372	312/479	0.92	[0.69; 1.23]	1.5%	1.5%
Sunawang 2009	86/371	79/367	1.09	[0.85; 1.38]	2.1%	2.1%
Fixed Effect Model			0.88	[0.83; 0.93]	39.9%	--
Random Effects Model			0.88	[0.83; 0.93]	--	39.9%
Heterogeneity: $I^2 = 0\%$, $\tau^2 = 0$, $p = 0.69$						
≥180 total pills						
Sunawang 2009	0/71	2/72	0.25	[0.03; 2.50]	0.0%	0.0%
Persson 2012	9/282	43/656	0.49	[0.24; 0.99]	0.2%	0.2%
Christian 2003	32/365	32/299	0.81	[0.52; 1.24]	0.7%	0.7%
Bhutta 2009	79/179	98/194	0.87	[0.67; 1.12]	1.9%	1.9%
West 2014	840/8259	929/8076	0.88	[0.80; 0.98]	11.8%	11.8%
Kaestel 2005	1/17	0/8	1.00	[0.09; 10.94]	0.0%	0.0%
Zagre 2007	148/661	122/666	1.24	[0.83; 1.84]	0.8%	0.8%
Roberfroid 2008	7/138	4/138	1.75	[0.52; 5.84]	0.1%	0.1%
Fixed Effect Model			0.89	[0.81; 0.97]	15.5%	--
Random Effects Model			0.89	[0.78; 1.02]	--	15.5%
Heterogeneity: $I^2 = 12\%$, $\tau^2 = 0.0051$, $p = 0.33$						
**Fixed Effect Model**			**0.90**	**[0.87; 0.93]**	**100.0%**	**--**
**Random Effects Model**			**0.90**	**[0.87; 0.93]**	**--**	**100.0%**

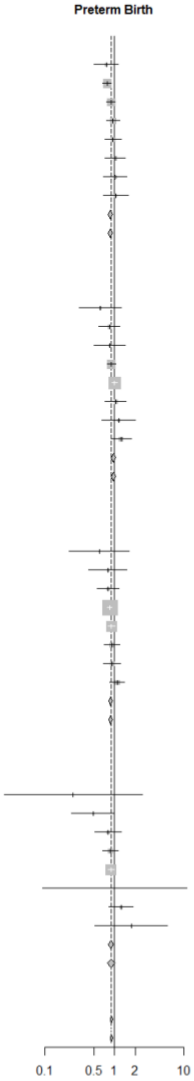

Heterogeneity: $I^2 = 0\%$, $\tau^2 = 0$, $p = 0.47$
Test of subgroup differences (fixed effect): $\chi^2_3 = 5.69$, df = 3 ($p = 0.13$)
Test for subgroup differences (random effects): $\chi^2_3 = 5.60$, df = 3 ($p = 0.13$)

Supplemental Figure 3.54. The effect of MMS vs. IFA on SGA10, stratified by Total Pill Count

Study	Events/Total (MMS)	Events/Total (IFA)	RR	95%-CI	Weight (Fixed)	Weight (random)
**<90 total pills**						
Ashorn 2015	6/34	7/32	0.81	[0.30; 2.14]	0.0%	0.2%
Fawzi 2007	116/893	129/897	0.90	[0.72; 1.14]	0.9%	2.6%
Roberfroid 2008	25/110	27/108	0.91	[0.57; 1.46]	0.2%	0.8%
Persson 2012	132/235	237/428	1.01	[0.88; 1.17]	2.2%	4.9%
Bhutta 2009	10/64	5/35	1.04	[0.46; 2.38]	0.1%	0.3%
Christian 2003	82/143	63/121	1.08	[0.87; 1.34]	1.0%	2.9%
Kaestel 2005	88/382	37/175	1.09	[0.78; 1.53]	0.4%	1.4%
West 2014	334/583	262/555	1.21	[1.08; 1.35]	3.7%	6.1%
Adu-Afarwuah 2015	21/59	14/48	1.22	[0.70; 2.13]	0.1%	0.6%
Sunawang 2009	26/179	18/169	1.36	[0.89; 2.09]	0.2%	0.9%
Osrin 2005	2/6	1/5	1.67	[0.21; 13.43]	0.0%	0.0%
Fixed Effect Model			1.10	[1.02; 1.18]	8.9%	--
Random Effects Model			1.10	[1.02; 1.18]	--	20.7%
Heterogeneity: $I^2 = 0\%$, $\tau^2 = 0$, $p = 0.49$						
**90-120 total pills**						
Adu-Afarwuah 2015	16/101	29/107	0.58	[0.34; 1.01]	0.2%	0.6%
Fawzi 2007	174/1106	247/1166	0.74	[0.62; 0.89]	1.4%	3.7%
Sunawang 2009	17/157	20/149	0.85	[0.50; 1.47]	0.2%	0.6%
Ashorn 2015	16/77	18/80	0.92	[0.51; 1.68]	0.1%	0.5%
Roberfroid 2008	31/102	34/105	0.94	[0.63; 1.40]	0.3%	1.1%
West 2014	163/372	178/387	0.96	[0.81; 1.13]	1.7%	4.1%
Christian 2003	36/78	35/77	0.98	[0.70; 1.37]	0.4%	1.4%
Persson 2012	51/100	113/226	1.02	[0.81; 1.29]	0.8%	2.6%
Kaestel 2005	48/167	24/90	1.08	[0.71; 1.64]	0.3%	1.0%
Osrin 2005	15/35	9/31	1.48	[0.76; 2.89]	0.1%	0.4%
Bhutta 2009	13/78	9/79	1.59	[0.70; 3.65]	0.1%	0.3%
Fixed Effect Model			0.91	[0.83; 0.99]	5.4%	--
Random Effects Model			0.92	[0.82; 1.04]	--	16.3%
Heterogeneity: $I^2 = 27\%$, $\tau^2 = 0.0098$, $p = 0.19$						
**120-180 pills**						
Kaestel 2005	28/109	21/63	0.77	[0.48; 1.24]	0.2%	0.8%
Osrin 2005	168/414	220/435	0.80	[0.69; 0.93]	2.0%	4.6%
Fawzi 2007	352/1611	380/1517	0.87	[0.77; 0.99]	2.9%	5.5%
Ashorn 2015	57/240	65/252	0.92	[0.68; 1.25]	0.5%	1.7%
Roberfroid 2008	54/166	57/166	0.95	[0.70; 1.28]	0.5%	1.7%
Adu-Afarwuah 2015	39/209	40/207	0.97	[0.65; 1.44]	0.3%	1.1%
West 2014	1313/2581	1330/2692	1.03	[0.97; 1.09]	12.2%	8.5%
Persson 2012	173/323	303/586	1.04	[0.91; 1.18]	2.7%	5.3%
Christian 2003	119/267	105/250	1.08	[0.91; 1.29]	1.5%	3.9%
Sunawang 2009	61/371	55/367	1.11	[0.86; 1.44]	0.7%	2.2%
Bhutta 2009	56/364	63/475	1.19	[0.63; 2.23]	0.1%	0.5%
Fixed Effect Model			0.99	[0.94; 1.03]	23.6%	--
Random Effects Model			0.97	[0.90; 1.04]	--	35.7%
Heterogeneity: $I^2 = 42\%$, $\tau^2 = 0.0053$, $p = 0.07$						
**≥180 total pills**						
Fawzi 2007	20/77	30/73	0.63	[0.40; 1.01]	0.2%	0.8%
Sunawang 2009	15/71	19/72	0.80	[0.61; 1.06]	0.6%	2.0%
Ashorn 2015	11/26	9/17	0.80	[0.42; 1.51]	0.1%	0.4%
Bhutta 2009	30/177	37/192	0.87	[0.52; 1.47]	0.2%	0.7%
West 2014	3947/6623	4084/6461	0.94	[0.92; 0.97]	54.6%	9.7%
Osrin 2005	42/94	32/71	0.99	[0.70; 1.39]	0.4%	1.4%
Persson 2012	156/282	351/656	1.03	[0.91; 1.17]	2.9%	5.5%
Roberfroid 2008	35/127	33/125	1.04	[0.70; 1.57]	0.3%	1.0%
Christian 2003	208/365	155/299	1.10	[0.97; 1.26]	2.7%	5.3%
Adu-Afarwuah 2015	8/14	8/18	1.29	[0.65; 2.56]	0.1%	0.4%
Kaestel 2005	4/13	0/5	3.38	[0.49; 23.18]	0.0%	0.1%
Fixed Effect Model			0.95	[0.93; 0.98]	62.0%	--
Random Effects Model			0.97	[0.90; 1.05]	--	27.3%
Heterogeneity: $I^2 = 30\%$, $\tau^2 = 0.0037$, $p = 0.16$						
**Fixed Effect Model**			**0.97**	**[0.95; 0.99]**	**100.0%**	**--**
**Random Effects Model**			**0.98**	**[0.94; 1.03]**	**--**	**100.0%**

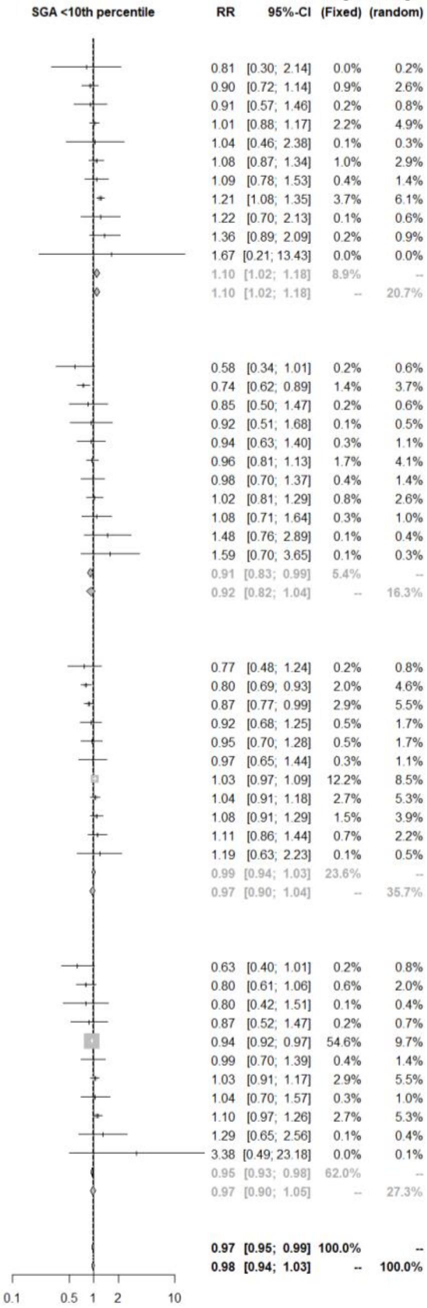

Heterogeneity: $I^2 = 39\%$, $\tau^2 = 0.0049$, $p < 0.01$
Test of subgroup differences (fixed effect): $\chi^2_3 = 16.17$, df = 3 ($p < 0.01$)
Test for subgroup differences (random effects): $\chi^2_3 = 9.58$, df = 3 ($p = 0.02$)

Supplemental Figure 3.55. The effect of MMS vs. IFA on Stillbirth, stratified by Total Pill Count

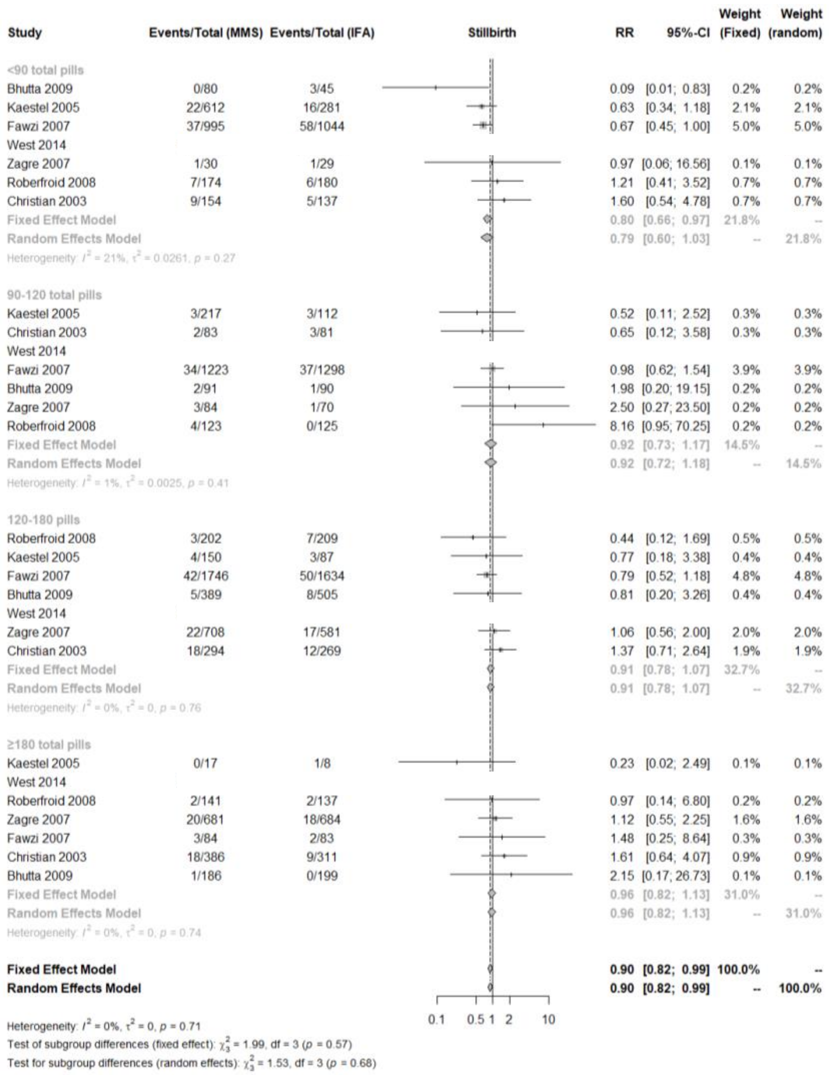

Objective 1 Sensivity analysis by MMS iron dose stratified by Total Pill Count

Supplemental Figure 3.56. The effect of MMS vs. IFA on hemoglobin, in trials with lower iron dose in MMS than IFA, stratified by total pill count

Study	Total MMS	MMS Mean (SD)	Total IFA	IFA Mean (SD)	MD	95%-CI	Weight (Fixed)	Weight (random)
**Lower Dose Iron: <90 total pills**								
Osrin 2005	2	11.6 (1.1)	4	11.9 (0.8)	-0.30	[-2.50; 1.80]	0.1%	0.1%
Adu-Afarwuah 2015	42	11.5 (1.1)	36	11.7 (1.4)	-0.20	[-0.70; 0.40]	1.1%	1.7%
Sunawang 2009	179	10.8 (1.3)	162	11 (1.3)	-0.20	[-0.48; 0.08]	4.1%	4.9%
Roberfroid 2008	54	11 (2)	52	11 (1.5)	0.10	[-0.60; 0.80]	0.7%	1.1%
Persson 2012	115	11.4 (1.2)	203	11.3 (1.3)	0.10	[-0.20; 0.30]	5.1%	5.7%
Bliznashka 2022	34	11.2 (1.1)	37	11 (1.1)	0.21	[-0.24; 0.65]	1.6%	2.4%
Fixed Effect Model					-0.01	[-0.17; 0.15]	12.6%	--
Random Effects Model					-0.01	[-0.17; 0.15]	--	15.8%
Heterogeneity: $I^2 = 0\%$, $\tau^2 = 0$, $p = 0.54$								
**Lower Dose Iron: 90-120 total pills**								
Sunawang 2009	153	10.9 (1.3)	146	11.2 (1.1)	-0.28	[-0.46; -0.10]	9.9%	8.0%
Bliznashka 2022	84	11.2 (1.6)	87	11.2 (1.5)	-0.06	[-0.59; 0.48]	1.1%	1.8%
Adu-Afarwuah 2015	96	11.7 (1.1)	87	11.7 (1)	0.00	[-0.30; 0.30]	3.6%	4.4%
Persson 2012	44	11.5 (1.1)	116	11.3 (1.1)	0.10	[-0.20; 0.50]	2.6%	3.6%
Osrin 2005	34	12 (1.4)	30	11.8 (1.6)	0.20	[-0.50; 1.00]	0.6%	1.0%
Roberfroid 2008	72	11 (1.7)	76	10.7 (1.7)	0.30	[-0.20; 0.90]	1.1%	1.7%
Fixed Effect Model					-0.11	[-0.24; 0.02]	18.8%	--
Random Effects Model					-0.04	[-0.23; 0.15]	--	20.4%
Heterogeneity: $I^2 = 39\%$, $\tau^2 = 0.0211$, $p = 0.15$								
**Lower Dose Iron: 120-180 total pills**								
Adu-Afarwuah 2015	209	11.7 (1.2)	208	12 (1.1)	-0.40	[-0.60; -0.20]	8.0%	7.3%
Roberfroid 2008	138	10.9 (1.7)	143	11.1 (1.5)	-0.30	[-0.60; 0.10]	2.6%	3.6%
Sunawang 2009	376	11 (1.2)	376	11.2 (1.2)	-0.16	[-0.32; 0.00]	12.5%	8.9%
Osrin 2005	407	11.8 (1.2)	408	11.8 (1.4)	-0.10	[-0.20; 0.10]	14.2%	9.3%
Bliznashka 2022	317	11.4 (1.2)	327	11.3 (1.2)	0.03	[-0.19; 0.24]	6.9%	6.7%
Persson 2012	169	11.6 (1.4)	313	11.5 (1.2)	0.10	[-0.10; 0.40]	5.1%	5.7%
Fixed Effect Model					-0.14	[-0.22; -0.05]	49.4%	--
Random Effects Model					-0.13	[-0.27; 0.00]	--	41.4%
Heterogeneity: $I^2 = 63\%$, $\tau^2 = 0.0182$, $p = 0.02$								
**Lower Dose Iron: ≥180 total pills**								
Adu-Afarwuah 2015	14	11.5 (1.4)	17	12.1 (1.2)	-0.60	[-1.50; 0.40]	0.4%	0.6%
Persson 2012	147	11.5 (1.1)	315	11.6 (1.2)	-0.10	[-0.30; 0.10]	8.0%	7.3%
Roberfroid 2008	107	10.9 (1.7)	102	10.9 (1.5)	0.00	[-0.40; 0.40]	2.0%	2.9%
Sunawang 2009	73	10.9 (1.1)	73	10.9 (0.9)	0.05	[-0.29; 0.38]	2.8%	3.8%
Bliznashka 2022	214	11.6 (1.4)	261	11.5 (1.1)	0.12	[-0.16; 0.40]	4.1%	4.9%
Osrin 2005	90	11.8 (1.3)	72	11.6 (1.3)	0.20	[-0.20; 0.60]	2.0%	2.9%
Fixed Effect Model					0.00	[-0.13; 0.13]	19.3%	--
Random Effects Model					0.00	[-0.13; 0.13]	--	22.3%
Heterogeneity: $I^2 = 0\%$, $\tau^2 = 0$, $p = 0.51$								
**Fixed Effect Model**					**-0.09**	**[-0.15; -0.03]**	**100.0%**	**--**
**Random Effects Model**					**-0.07**	**[-0.14; 0.01]**	**--**	**100.0%**

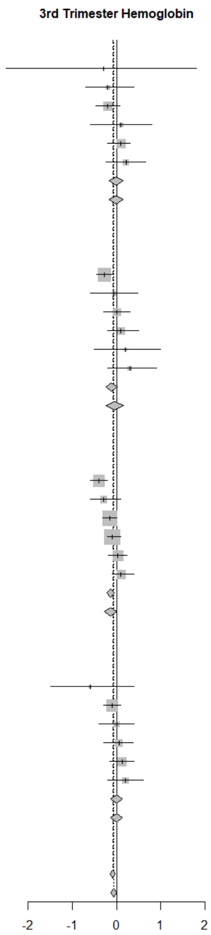

Heterogeneity: $I^2 = 33\%$, $\tau^2 = 0.0101$, $p = 0.06$

Test of subgroup differences (fixed effect): $\chi^2_3 = 4.23$, df = 3 ($p = 0.24$)

Test for subgroup differences (random effects): $\chi^2_3 = 2.25$, df = 3 ($p = 0.52$)

Supplemental Figure 3.57. The effect of MMS vs. IFA on hemoglobin, in trials with same iron dose in MMS than IFA, stratified by total pill count

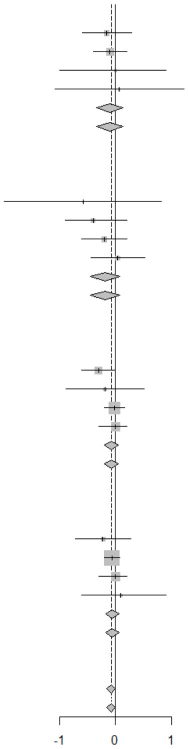

Study	Total MMS	MMS Mean (SD)	Total IFA	IFA Mean (SD)	MD	95%-CI	Weight (Fixed)	Weight (random)
**Same Dose Iron: <90 total pills**								
West 2014	30	11.29 (0.92)	24	11.44 (0.96)	-0.15	[-0.59; 0.29]	3.2%	3.2%
Persson 2012	122	11.1 (1.3)	219	11.2 (1.4)	-0.10	[-0.40; 0.20]	6.8%	6.8%
Ashorn 2015	24	11.2 (1.6)	19	11.2 (1.5)	0.00	[-1.00; 0.90]	0.7%	0.7%
Christian 2003	15	10.8 (1.5)	8	10.7 (1.4)	0.07	[-1.08; 1.23]	0.5%	0.5%
**Fixed Effect Model**					-0.10	[-0.34; 0.13]	11.1%	--
**Random Effects Model**					-0.10	[-0.34; 0.13]	--	11.1%
Heterogeneity: $I^2 = 0\%$, $\tau^2 = 0$, $p = 0.98$								
**Same Dose Iron: 90-120 total pills**								
Christian 2003	11	11.4 (1.8)	9	12 (1.7)	-0.58	[-1.98; 0.82]	0.3%	0.3%
Ashorn 2015	72	11 (1.5)	67	11.4 (1.6)	-0.40	[-0.90; 0.20]	2.0%	2.0%
Persson 2012	54	11.4 (1.3)	107	11.6 (1.2)	-0.20	[-0.60; 0.20]	3.8%	3.8%
West 2014	29	11.06 (1.31)	47	11.03 (1.05)	0.04	[-0.44; 0.52]	2.7%	2.7%
**Fixed Effect Model**					-0.19	[-0.45; 0.08]	8.8%	--
**Random Effects Model**					-0.19	[-0.45; 0.08]	--	8.8%
Heterogeneity: $I^2 = 0\%$, $\tau^2 = 0$, $p = 0.63$								
**Same Dose Iron: 120-180 pills**								
Ashorn 2015	228	10.9 (1.4)	237	11.2 (1.5)	-0.30	[-0.60; 0.00]	6.8%	6.8%
Christian 2003	57	11.4 (1.9)	44	11.6 (1.5)	-0.18	[-0.88; 0.51]	1.3%	1.3%
West 2014	194	11.20 (1.00)	230	11.22 (1.10)	-0.02	[-0.20; 0.17]	18.1%	18.1%
Persson 2012	163	11.5 (1.3)	259	11.5 (1.3)	0.00	[-0.30; 0.20]	9.8%	9.8%
**Fixed Effect Model**					-0.07	[-0.20; 0.06]	36.0%	--
**Random Effects Model**					-0.07	[-0.20; 0.06]	--	36.0%
Heterogeneity: $I^2 = 0\%$, $\tau^2 = 0$, $p = 0.40$								
**Same Dose Iron: ≥180 total pills**								
Christian 2003	84	11.6 (1.5)	69	11.8 (1.4)	-0.22	[-0.72; 0.28]	2.4%	2.4%
West 2014	521	11.00 (1.05)	515	11.06 (0.99)	-0.06	[-0.20; 0.08]	30.8%	30.8%
Persson 2012	151	11.6 (1.1)	327	11.6 (1.2)	0.00	[-0.30; 0.20]	9.8%	9.8%
Ashorn 2015	27	11.3 (1.3)	20	11.2 (1.2)	0.10	[-0.60; 0.90]	1.1%	1.1%
**Fixed Effect Model**					-0.05	[-0.17; 0.07]	44.1%	--
**Random Effects Model**					-0.05	[-0.17; 0.07]	--	44.1%
Heterogeneity: $I^2 = 0\%$, $\tau^2 = 0$, $p = 0.86$								
**Fixed Effect Model**					**-0.08**	**[-0.16; 0.00]**	**100.0%**	**--**
**Random Effects Model**					**-0.08**	**[-0.16; 0.00]**	**--**	**100.0%**

Heterogeneity: $I^2 = 0\%$, $\tau^2 = 0$, $p = 0.97$
Test of subgroup differences (fixed effect): $\chi^2_3 = 0.88$, df = 3 ($p = 0.83$)
Test for subgroup differences (random effects): $\chi^2_3 = 0.88$, df = 3 ($p = 0.83$)

Supplemental Figure 3.58. The effect of MMS vs. IFA on anemia, in trials with lower iron dose in MMS than IFA, stratified by total pill count

Study	Events/Total (MMS)	Events/Total (IFA)	RR	95%-CI	Weight (Fixed)	Weight (random)
**Lower Dose Iron: <90 total pills**						
Bliznashka 2022	8/34	13/37	0.67	[0.35; 1.28]	0.8%	1.0%
Roberfroid 2008	28/54	28/52	0.96	[0.67; 1.38]	2.4%	3.0%
Persson 2012	45/115	79/203	1.01	[0.76; 1.34]	3.9%	4.6%
Sunawang 2009	101/179	81/162	1.13	[0.95; 1.35]	10.3%	10.0%
Adu-Afarwuah 2015	13/42	6/36	1.86	[0.79; 4.38]	0.4%	0.6%
Osrin 2005	1/2	0/4	4.50	[0.67; 30.23]	0.1%	0.1%
Fixed Effect Model			1.07	[0.94; 1.23]	17.9%	--
Random Effects Model			1.06	[0.88; 1.27]	--	19.3%
Heterogeneity: I^2 = 25%, τ^2 = 0.0127, p = 0.25						
**Lower Dose Iron: 90-120 total pills**						
Osrin 2005	6/34	10/30	0.53	[0.22; 1.28]	0.4%	0.5%
Roberfroid 2008	35/72	45/76	0.82	[0.61; 1.11]	3.5%	4.2%
Adu-Afarwuah 2015	21/96	17/87	1.12	[0.63; 1.98]	1.0%	1.3%
Persson 2012	16/44	37/116	1.14	[0.71; 1.83]	1.4%	1.8%
Bliznashka 2022	24/84	21/87	1.18	[0.61; 2.31]	0.7%	0.9%
Sunawang 2009	81/153	63/146	1.23	[0.99; 1.52]	6.9%	7.4%
Fixed Effect Model			1.07	[0.92; 1.24]	14.0%	--
Random Effects Model			1.03	[0.84; 1.27]	--	16.2%
Heterogeneity: I^2 = 31%, τ^2 = 0.0201, p = 0.20						
**Lower Dose Iron: 120-180 pills**						
Osrin 2005	95/407	100/408	0.95	[0.75; 1.22]	5.4%	6.0%
Persson 2012	56/169	102/313	1.02	[0.78; 1.33]	4.5%	5.1%
Sunawang 2009	172/376	159/376	1.08	[0.97; 1.20]	28.0%	18.5%
Roberfroid 2008	67/138	64/143	1.08	[0.84; 1.39]	5.0%	5.7%
Bliznashka 2022	97/317	79/327	1.27	[1.00; 1.61]	5.6%	6.2%
Adu-Afarwuah 2015	52/209	32/208	1.62	[1.09; 2.40]	2.0%	2.5%
Fixed Effect Model			1.10	[1.01; 1.19]	50.5%	--
Random Effects Model			1.11	[1.00; 1.23]	--	44.1%
Heterogeneity: I^2 = 28%, τ^2 = 0.0049, p = 0.23						
**Lower Dose Iron: ≥180 total pills**						
Osrin 2005	18/90	18/72	0.80	[0.45; 1.42]	1.0%	1.2%
Roberfroid 2008	53/107	56/102	0.90	[0.70; 1.17]	4.8%	5.5%
Sunawang 2009	35/73	36/73	0.97	[0.78; 1.22]	6.3%	6.9%
Persson 2012	42/147	91/315	0.99	[0.73; 1.35]	3.4%	4.0%
Bliznashka 2022	59/214	55/261	1.31	[0.87; 1.97]	1.9%	2.4%
Adu-Afarwuah 2015	5/14	4/17	1.52	[0.50; 4.60]	0.3%	0.3%
Fixed Effect Model			0.98	[0.86; 1.12]	17.6%	--
Random Effects Model			0.98	[0.86; 1.12]	--	20.4%
Heterogeneity: I^2 = 0%, τ^2 = 0, p = 0.63						
**Fixed Effect Model**			**1.07**	**[1.01; 1.13]**	**100.0%**	**--**
**Random Effects Model**			**1.06**	**[1.00; 1.14]**	**--**	**100.0%**

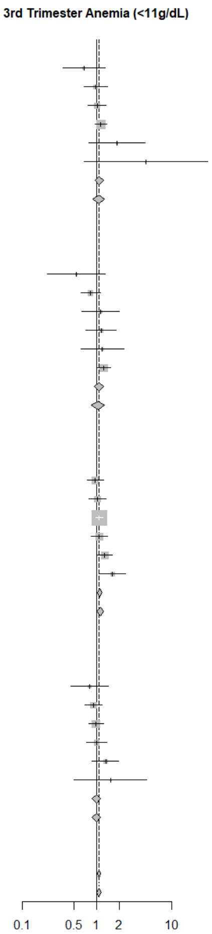

Heterogeneity: I^2 = 13%, τ^2 = 0.0031, p = 0.29
Test of subgroup differences (fixed effect): χ^2_3 = 1.97, df = 3 (p = 0.58)
Test for subgroup differences (random effects): χ^2_3 = 1.94, df = 3 (p = 0.59)

Supplemental Figure 3.59. The effect of MMS vs. IFA on anemia, in trials with same iron dose in MMS than IFA, stratified by total pill count

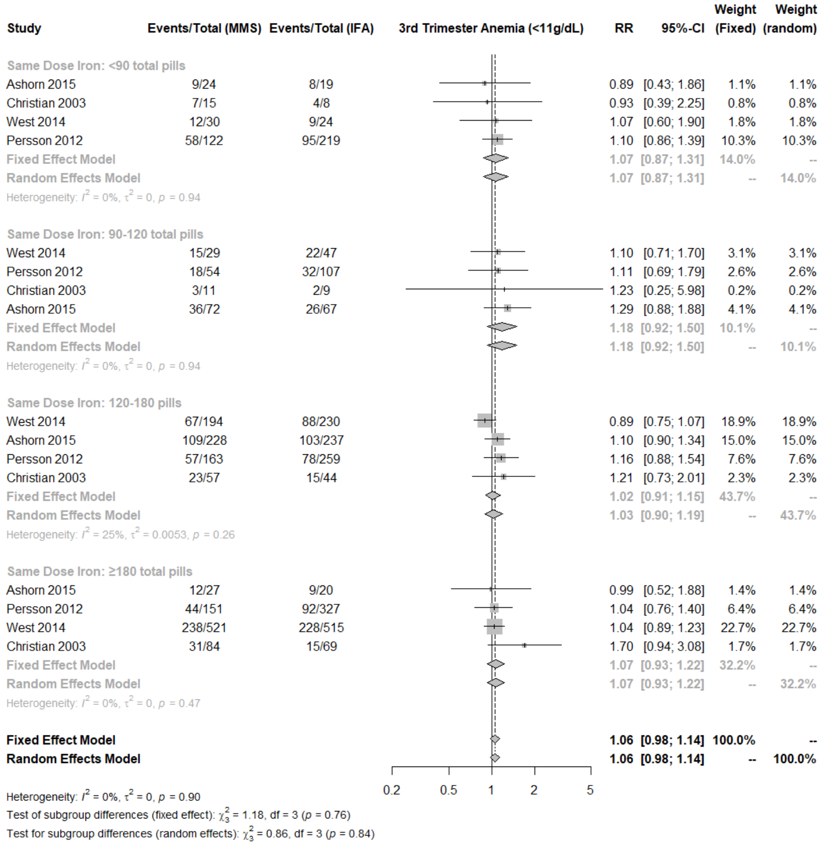

Supplemental Figure 3.60. The effect of MMS vs. IFA on IDA, in trials with lower iron dose in MMS than IFA, stratified by total pill count

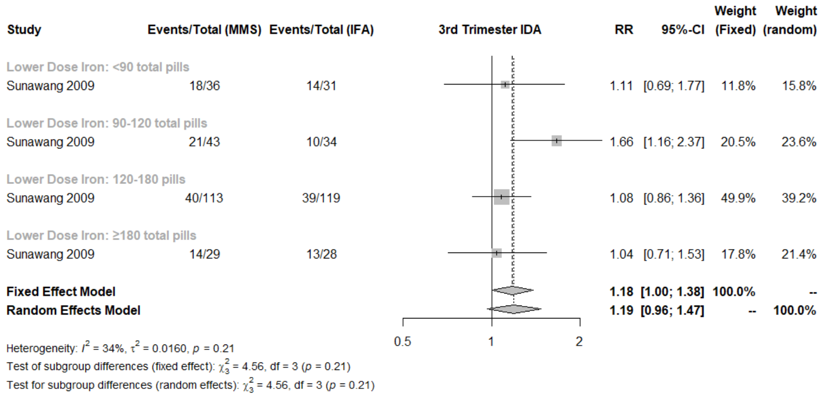

Supplemental Figure 3.61. The effect of MMS vs. IFA on IDA, in trials with same iron dose in MMS than IFA, stratified by total pill count

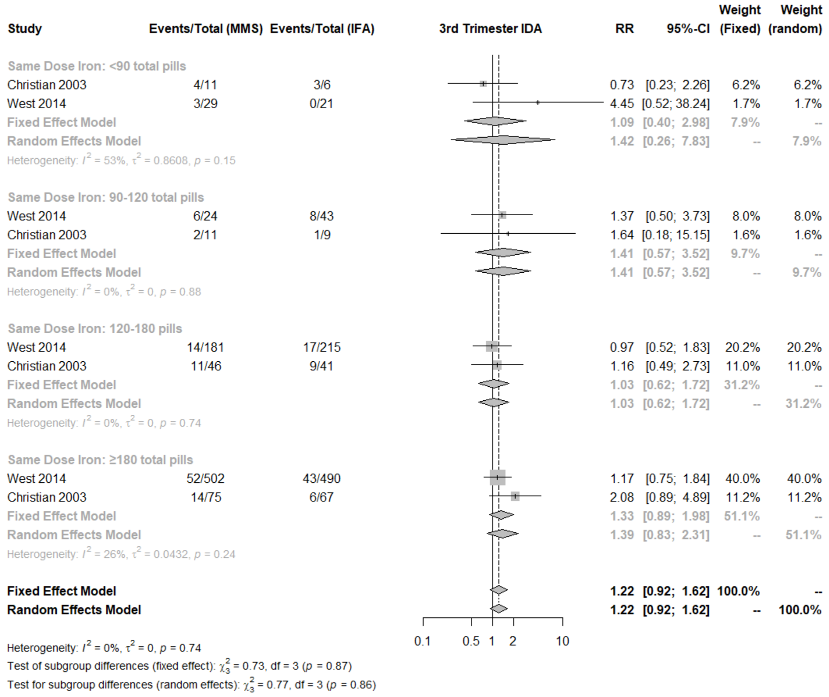

Supplemental Figure 3.62. The effect of MMS vs. IFA on birthweight, in trials with lower iron dose in MMS than IFA, stratified by total pill count

Study	Total MMS	MMS Mean (SD)	Total IFA	IFA Mean (SD)	MD	95%-CI	Weight (Fixed)	Weight (random)
**Lower Dose Iron: <90 total pills**								
Osrin 2005	6	2430 (816)	5	2830 (645)	-400.00	[-1420.00; 620.00]	0.0%	0.0%
Zagre 2007	29	3141 (759)	28	3164 (487)	-22.91	[-398.14; 352.33]	0.2%	0.2%
Sunawang 2009	179	3037 (477)	169	3054 (427)	-16.97	[-85.99; 52.05]	6.0%	6.0%
Persson 2012	116	2649 (402)	211	2628 (429)	21.00	[-74.00; 117.00]	3.1%	3.1%
Kaestel 2005	403	3043 (493)	184	3015 (468)	28.00	[-57.00; 113.00]	4.0%	4.0%
Adu-Afarwuah 2015	59	2805 (557)	48	2761 (511)	44.00	[-163.00; 250.00]	0.7%	0.7%
Roberfroid 2008	125	2854 (512)	126	2787 (489)	67.00	[-58.00; 191.00]	1.8%	1.8%
Bliznashka 2022	77	2999 (525)	71	2894 (551)	104.21	[-139.66; 348.07]	0.5%	0.5%
Bhutta 2009	66	3083 (661)	37	2919 (605)	163.99	[4.45; 323.53]	1.1%	1.1%
Fixed Effect Model					25.60	[-14.89; 66.08]	17.5%	--
Random Effects Model					25.60	[-14.89; 66.08]	--	17.5%
Heterogeneity: $I^2 = 0\%$, $\tau^2 = 0$, $p = 0.65$								
**Lower Dose Iron: 90-120 total pills**								
Persson 2012	45	2623 (433)	123	2715 (433)	-92.00	[-241.00; 57.00]	1.3%	1.3%
Osrin 2005	35	2499 (538)	32	2546 (567)	-48.00	[-317.00; 222.00]	0.4%	0.4%
Sunawang 2009	157	3061 (486)	149	3074 (484)	-13.35	[-72.23; 45.53]	8.3%	8.3%
Roberfroid 2008	101	2910 (436)	104	2874 (433)	37.00	[-83.00; 156.00]	2.0%	2.0%
Zagre 2007	81	2953 (685)	69	2901 (751)	51.64	[-263.71; 366.99]	0.3%	0.3%
Bliznashka 2022	134	3077 (453)	117	3020 (444)	57.64	[-100.50; 215.79]	1.1%	1.1%
Kaestel 2005	178	3093 (506)	95	2971 (441)	122.00	[1.00; 243.00]	2.0%	2.0%
Bhutta 2009	80	3030 (631)	81	2907 (598)	123.46	[-72.30; 319.21]	0.7%	0.7%
Adu-Afarwuah 2015	101	3009 (390)	107	2883 (477)	126.00	[6.00; 246.00]	2.0%	2.0%
Fixed Effect Model					27.04	[-12.77; 66.84]	18.1%	--
Random Effects Model					37.46	[-14.38; 89.31]	--	18.1%
Heterogeneity: $I^2 = 25\%$, $\tau^2 = 1476.5589$, $p = 0.22$								
**Lower Dose Iron: 120-180 pills**								
Persson 2012	175	2654 (419)	323	2650 (404)	4.00	[-71.00; 80.00]	5.0%	5.0%
Sunawang 2009	372	3102 (416)	367	3094 (408)	8.01	[-62.16; 78.18]	5.8%	5.8%
Adu-Afarwuah 2015	209	3053 (366)	207	3013 (408)	39.00	[-35.00; 114.00]	5.2%	5.2%
Bliznashka 2022	380	3111 (481)	424	3056 (390)	55.09	[-88.63; 198.82]	1.4%	1.4%
Zagre 2007	686	3056 (554)	564	3000 (565)	55.89	[-50.90; 162.69]	2.5%	2.5%
Roberfroid 2008	163	2927 (429)	167	2871 (411)	56.00	[-35.00; 147.00]	3.5%	3.5%
Osrin 2005	414	2818 (440)	436	2728 (412)	89.00	[32.00; 147.00]	8.7%	8.7%
Bhutta 2009	366	2958 (636)	480	2851 (530)	107.02	[-9.17; 223.22]	2.1%	2.1%
Kaestel 2005	120	3172 (457)	70	3055 (472)	118.00	[-19.00; 255.00]	1.5%	1.5%
Fixed Effect Model					52.05	[23.71; 80.38]	35.6%	--
Random Effects Model					52.05	[23.71; 80.38]	--	35.6%
Heterogeneity: $I^2 = 0\%$, $\tau^2 = 0$, $p = 0.59$								
**Lower Dose Iron: ≥180 total pills**								
Kaestel 2005	14	3277 (511)	5	3394 (328)	-117.00	[-638.00; 405.00]	0.1%	0.1%
Roberfroid 2008	125	2959 (441)	119	2995 (344)	-37.00	[-137.00; 63.00]	2.9%	2.9%
Adu-Afarwuah 2015	14	2913 (362)	18	2941 (361)	-28.00	[-291.00; 235.00]	0.4%	0.4%
Sunawang 2009	71	3155 (381)	72	3146 (351)	9.10	[-39.79; 57.99]	12.0%	12.0%
Bhutta 2009	177	2993 (510)	193	2978 (574)	14.76	[-99.32; 128.83]	2.2%	2.2%
Persson 2012	149	2754 (415)	323	2736 (375)	18.00	[-57.00; 94.00]	5.0%	5.0%
Zagre 2007	661	3111 (519)	666	3049 (433)	62.55	[-30.89; 155.98]	3.3%	3.3%
Osrin 2005	94	2938 (381)	71	2842 (362)	96.00	[-19.00; 212.00]	2.1%	2.1%
Bliznashka 2022	205	3184 (483)	262	3049 (454)	134.67	[-50.58; 319.93]	0.8%	0.8%
Fixed Effect Model					21.69	[-9.82; 53.19]	28.8%	--
Random Effects Model					21.69	[-9.82; 53.19]	--	28.8%
Heterogeneity: $I^2 = 0\%$, $\tau^2 = 0$, $p = 0.67$								
**Fixed Effect Model**					**34.16**	**[17.24; 51.08]**	**100.0%**	**--**
**Random Effects Model**					**34.16**	**[17.24; 51.08]**	**--**	**100.0%**

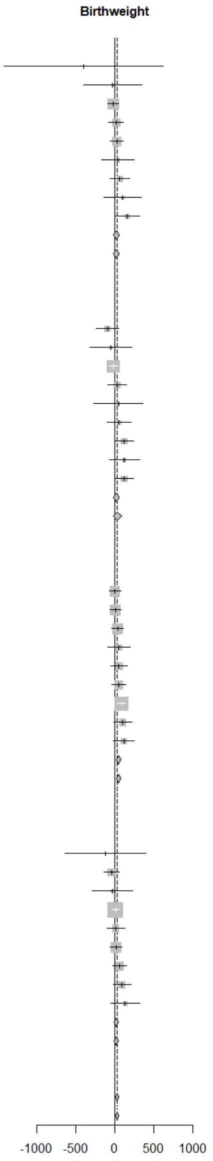

Heterogeneity: $I^2 = 0\%$, $\tau^2 = 0$, $p = 0.65$
Test of subgroup differences (fixed effect): $\chi^2_3 = 2.43$, df = 3 ($p = 0.49$)
Test for subgroup differences (random effects): $\chi^2_3 = 2.28$, df = 3 ($p = 0.52$)

Supplemental Figure 3.63. The effect of MMS vs. IFA on birthweight, in trials with same iron dose in MMS than IFA, stratified by total pill count

Study	Total MMS	MMS Mean (SD)	Total IFA	IFA Mean (SD)	MD	95%-CI	Weight (Fixed)	Weight (random)
**Same Dose Iron: <90 total pills**								
Ashorn 2015	34	2713 (624)	33	2772 (484)	-59.00	[-333.00; 214.00]	0.1%	0.1%
Persson 2012	125	2611 (394)	225	2634 (459)	-23.00	[-119.00; 73.00]	1.1%	1.1%
Christian 2003	143	2537 (544)	125	2535 (480)	1.81	[-136.53; 140.15]	0.5%	0.5%
West 2014	631	2549 (430)	599	2508 (432)	40.95	[-8.45; 90.34]	4.0%	4.0%
Fawzi 2007	893	3036 (549)	897	2977 (586)	59.00	[6.00; 111.00]	3.5%	3.5%
Fixed Effect Model					36.97	[4.47; 69.47]	9.2%	--
Random Effects Model					36.97	[4.47; 69.47]	--	9.2%
Heterogeneity: $I^2 = 0\%$, $\tau^2 = 0$, $p = 0.57$								
**Same Dose Iron: 90-120 total pills**								
Ashorn 2015	77	2872 (483)	80	2866 (518)	7.00	[-151.00; 165.00]	0.4%	0.4%
Persson 2012	55	2650 (382)	107	2627 (448)	23.00	[-117.00; 163.00]	0.5%	0.5%
West 2014	412	2500 (455)	423	2435 (472)	65.85	[4.95; 126.74]	2.6%	2.6%
Fawzi 2007	1106	3167 (471)	1166	3089 (483)	78.00	[39.00; 117.00]	6.4%	6.4%
Christian 2003	79	2605 (453)	77	2522 (461)	82.27	[-70.44; 234.98]	0.4%	0.4%
Fixed Effect Model					69.76	[39.06; 100.46]	10.3%	--
Random Effects Model					69.76	[39.06; 100.46]	--	10.3%
Heterogeneity: $I^2 = 0\%$, $\tau^2 = 0$, $p = 0.87$								
**Same Dose Iron: 120-180 pills**								
Persson 2012	167	2644 (437)	272	2640 (430)	4.00	[-79.00; 88.00]	1.4%	1.4%
Ashorn 2015	240	3033 (416)	252	3004 (385)	29.00	[-42.00; 100.00]	1.9%	1.9%
Christian 2003	267	2662 (428)	250	2616 (460)	46.06	[-38.42; 130.54]	1.4%	1.4%
West 2014	2666	2486 (427)	2784	2433 (437)	52.74	[28.67; 76.81]	16.7%	16.7%
Fawzi 2007	1611	3239 (474)	1517	3172 (465)	67.00	[34.00; 100.00]	8.9%	8.9%
Fixed Effect Model					52.89	[35.00; 70.77]	30.3%	--
Random Effects Model					52.89	[35.00; 70.77]	--	30.3%
Heterogeneity: $I^2 = 0\%$, $\tau^2 = 0$, $p = 0.65$								
**Same Dose Iron: ≥180 total pills**								
Persson 2012	154	2739 (420)	338	2755 (421)	-16.00	[-97.00; 64.00]	1.5%	1.5%
Christian 2003	365	2728 (446)	299	2732 (399)	-3.61	[-66.28; 59.07]	2.5%	2.5%
West 2014	6930	2631 (384)	6720	2580 (391)	50.95	[36.42; 65.49]	45.8%	45.8%
Ashorn 2015	26	3012 (463)	17	2951 (461)	61.00	[-230.00; 352.00]	0.1%	0.1%
Fawzi 2007	77	3337 (493)	73	3190 (452)	147.00	[-6.00; 300.00]	0.4%	0.4%
Fixed Effect Model					47.10	[33.23; 60.98]	50.3%	--
Random Effects Model					32.42	[-8.97; 73.80]	--	50.3%
Heterogeneity: $I^2 = 41\%$, $\tau^2 = 837.1065$, $p = 0.15$								
**Fixed Effect Model**					**50.25**	**[40.41; 60.09]**	**100.0%**	**--**
**Random Effects Model**					**50.25**	**[40.41; 60.09]**	**--**	**100.0%**

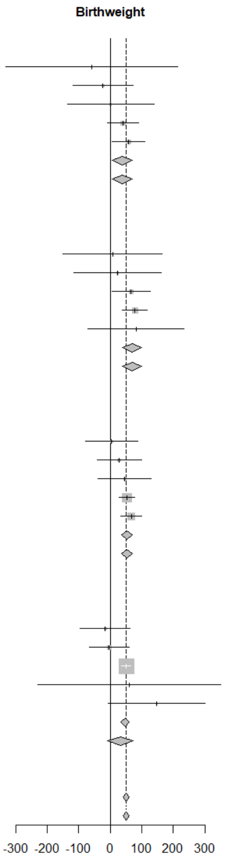

Heterogeneity: $I^2 = 0\%$, $\tau^2 = 0$, $p = 0.66$
Test of subgroup differences (fixed effect): $\chi^2_3 = 2.47$, df = 3 ($p = 0.48$)
Test for subgroup differences (random effects): $\chi^2_3 = 2.97$, df = 3 ($p = 0.40$)

Objective 2 Outcomes stratified by Adherence

Supplemental Figure 4.1. The effect of MMS on Anemia, stratified by Adherence

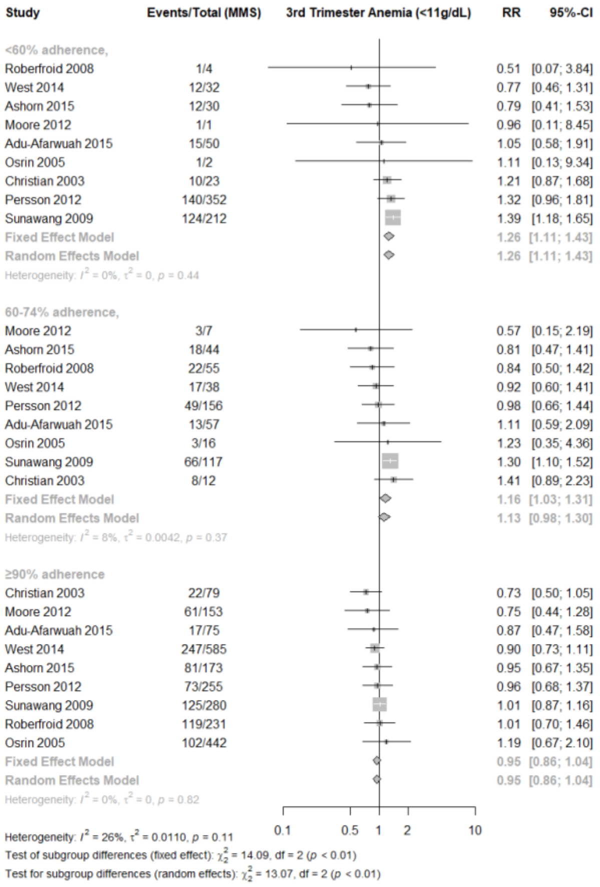

Supplemental Figure 4.2. The effect of MMS on Birthweight Percentile, stratified by Adherence

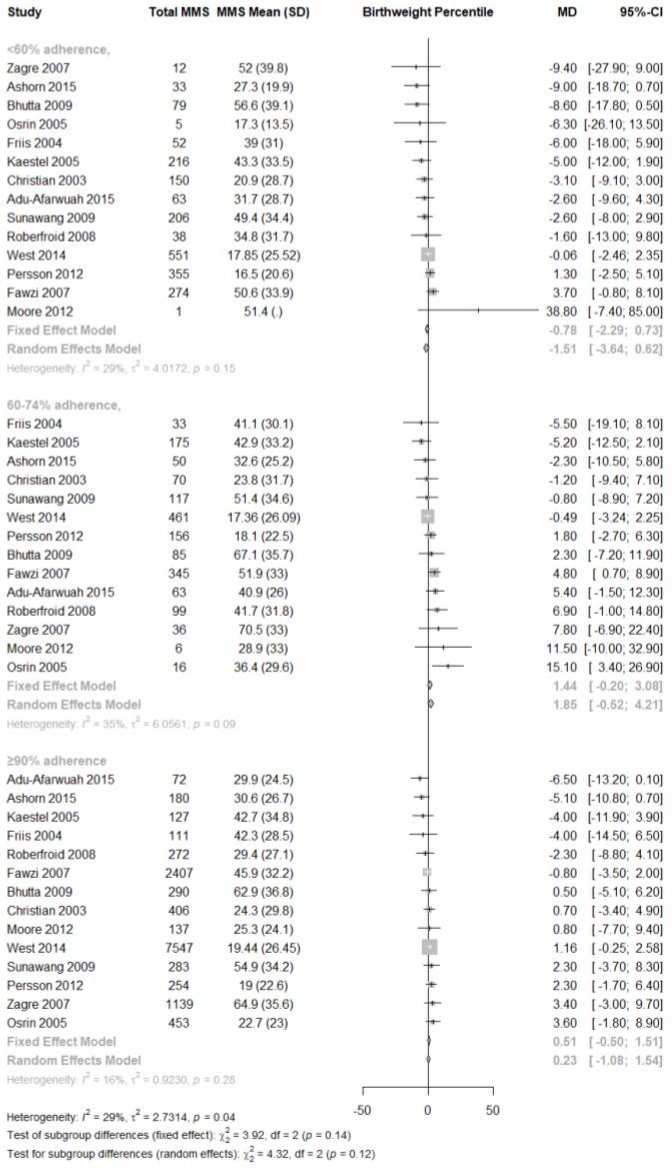

Supplemental Figure 4.3. The effect of MMS on Birthweight, stratified by Adherence

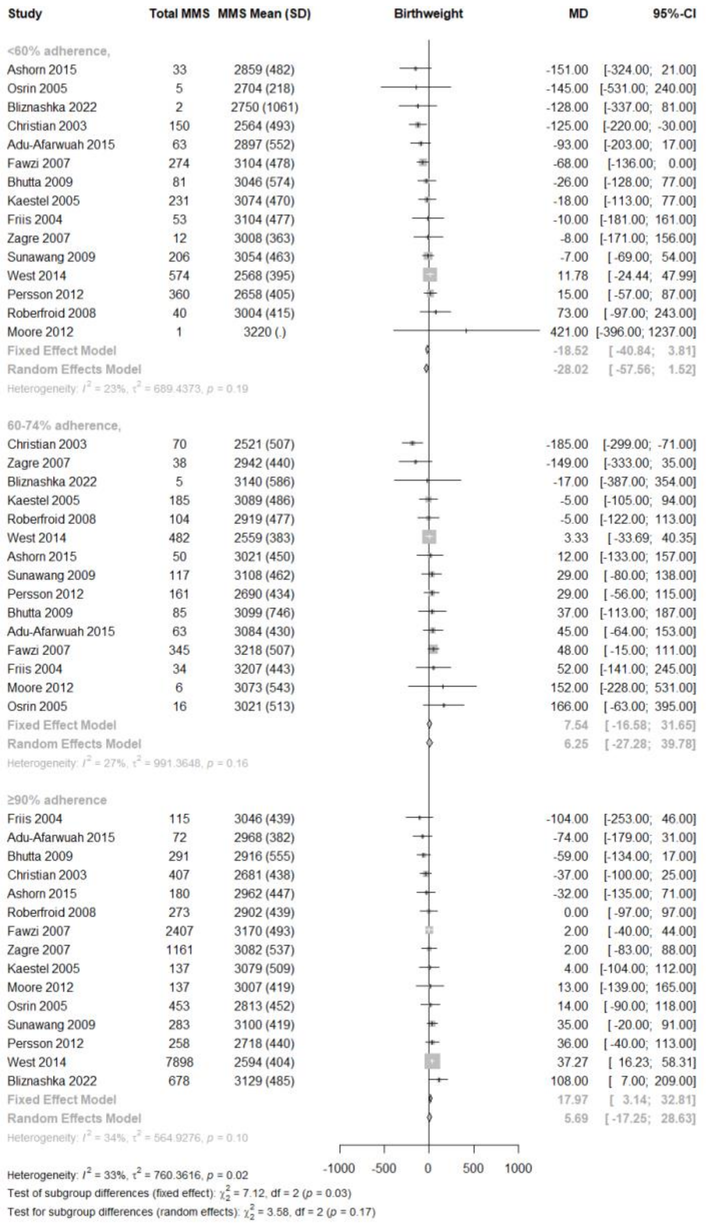

Supplemental Figure 4.4. The effect of MMS on Continuous Gestation, stratified by Adherence

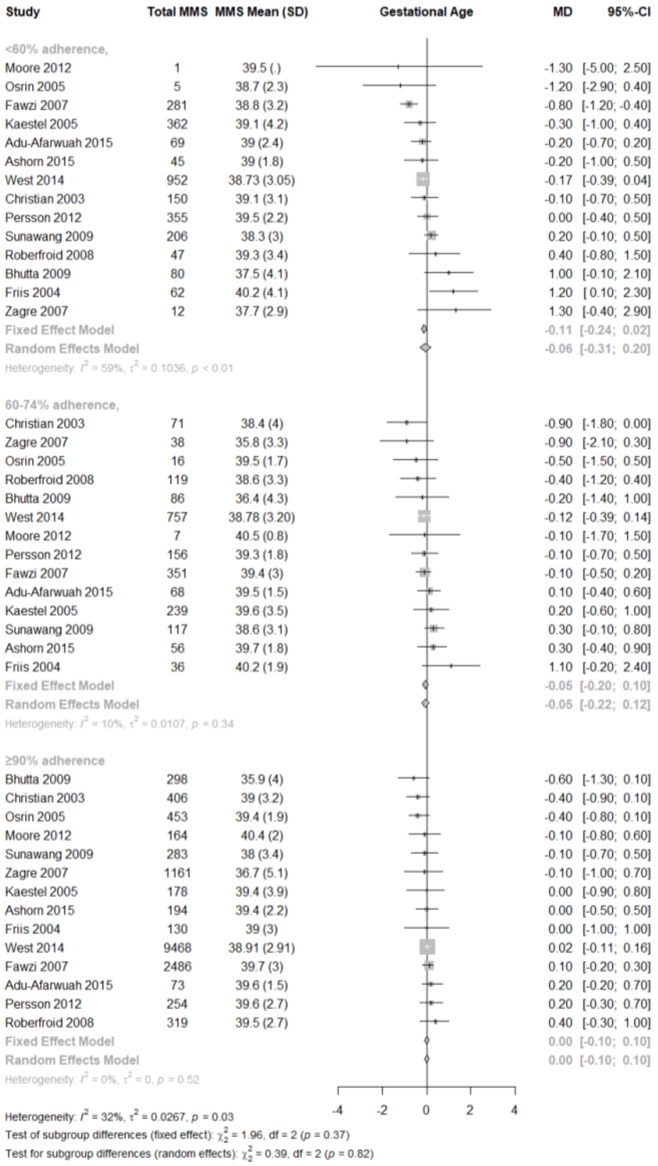

Supplemental Figure 4.5. The effect of MMS on Fetal Death, stratified by Adherence

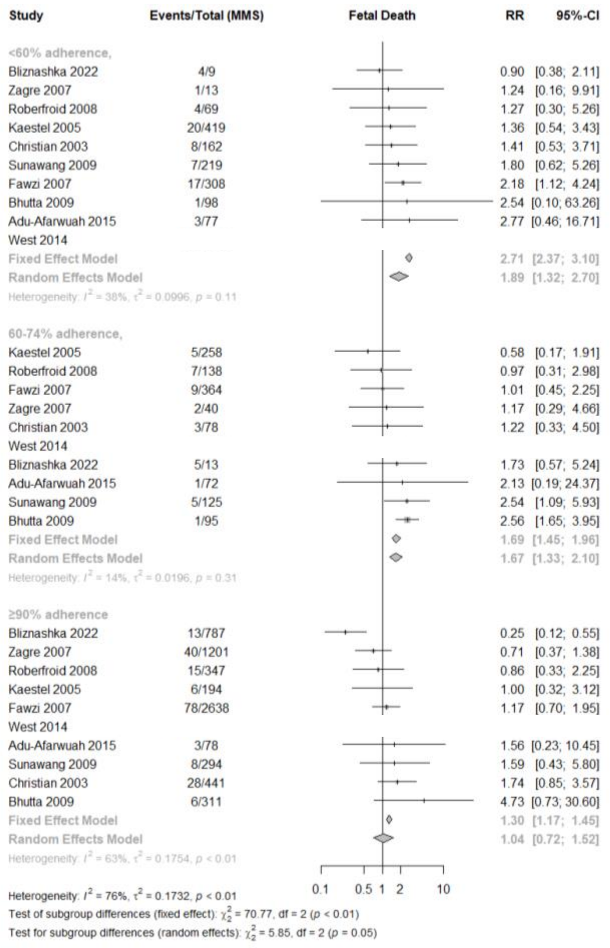

Supplemental Figure 4.6. The effect of MMS on Hemoglobin Continuous, stratified by Adherence

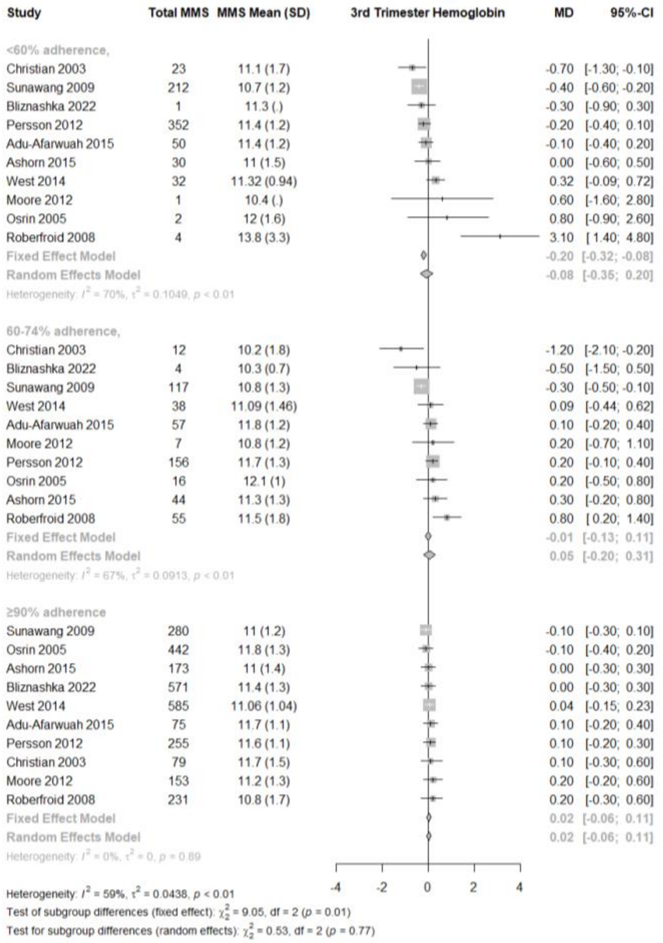

Supplemental Figure 4.7. The effect of MMS on IDA, stratified by Adherence

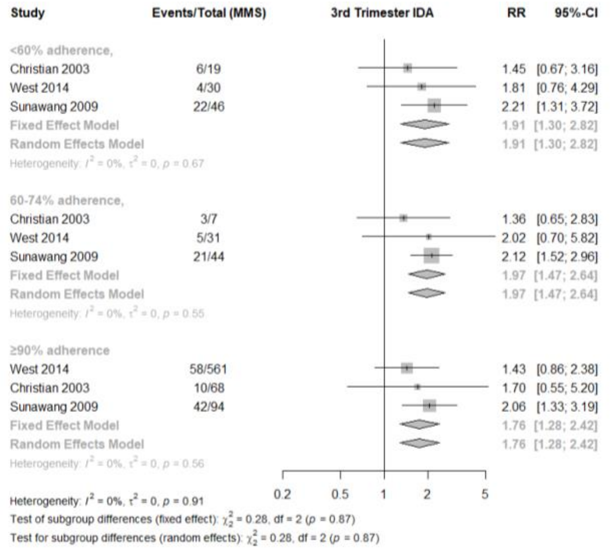

Supplemental Figure 4.8. The effect of MMS on Infant Mortality, stratified by Adherence

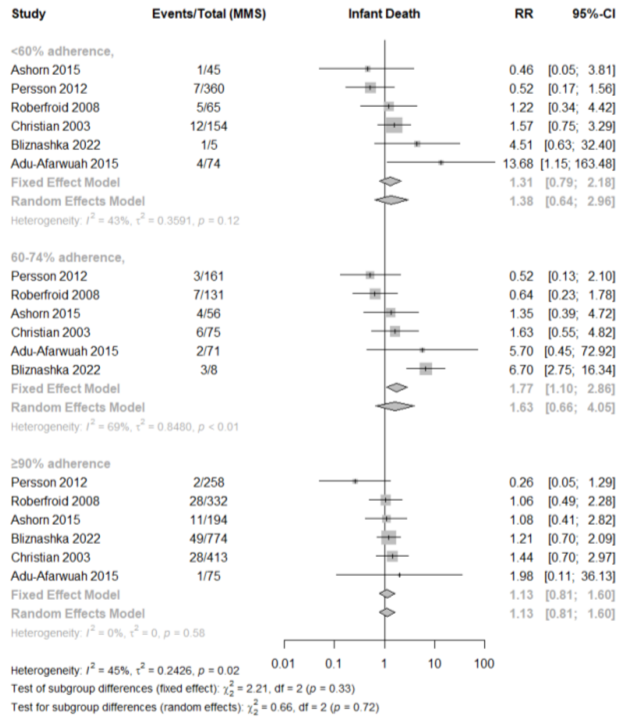

Supplemental Figure 4.9. The effect of MMS on LGA90, stratified by Adherence

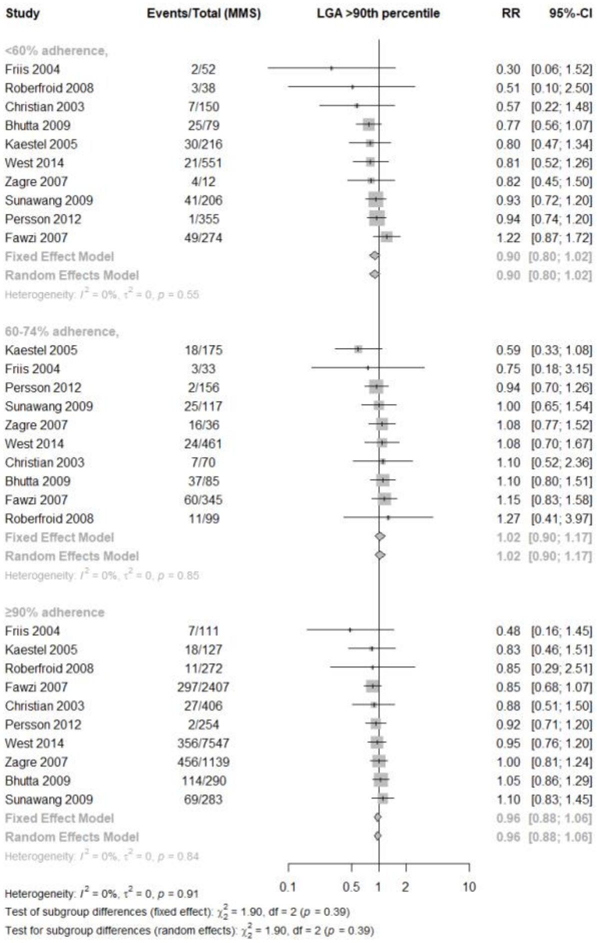

Supplemental Figure 4.10. The effect of MMS on Low Birthweight, stratified by Adherence

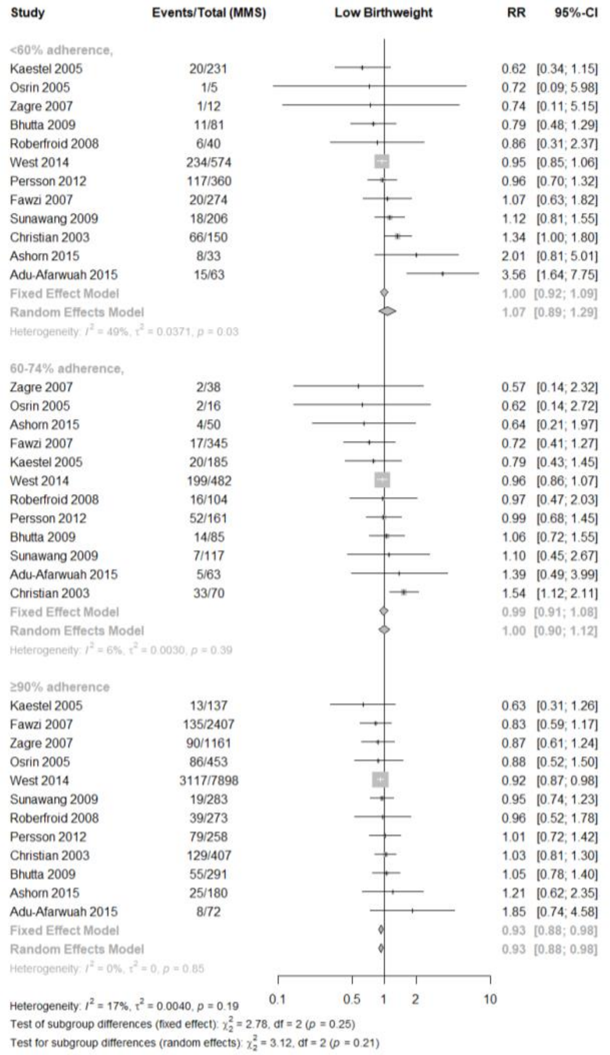

Supplemental Figure 4.11. The effect of MMS on Neonatal Mortality, stratified by Adherence

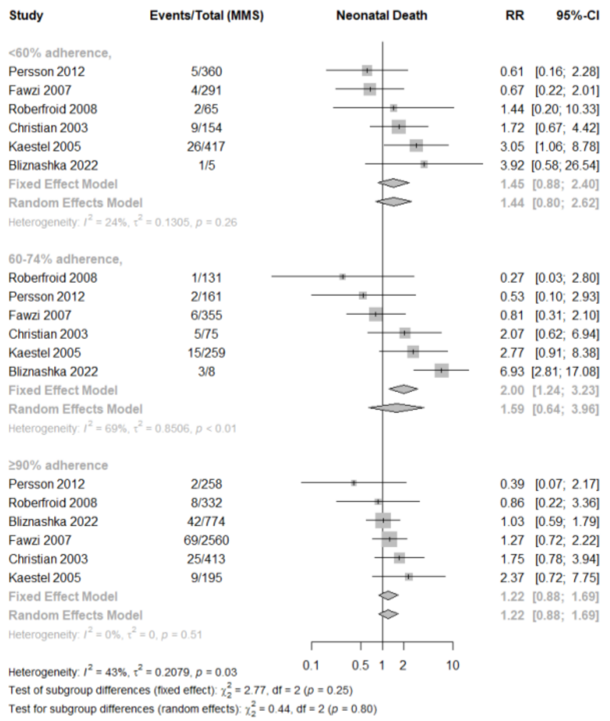

Supplemental Figure 4.12. The effect of MMS on Preterm, stratified by Adherence

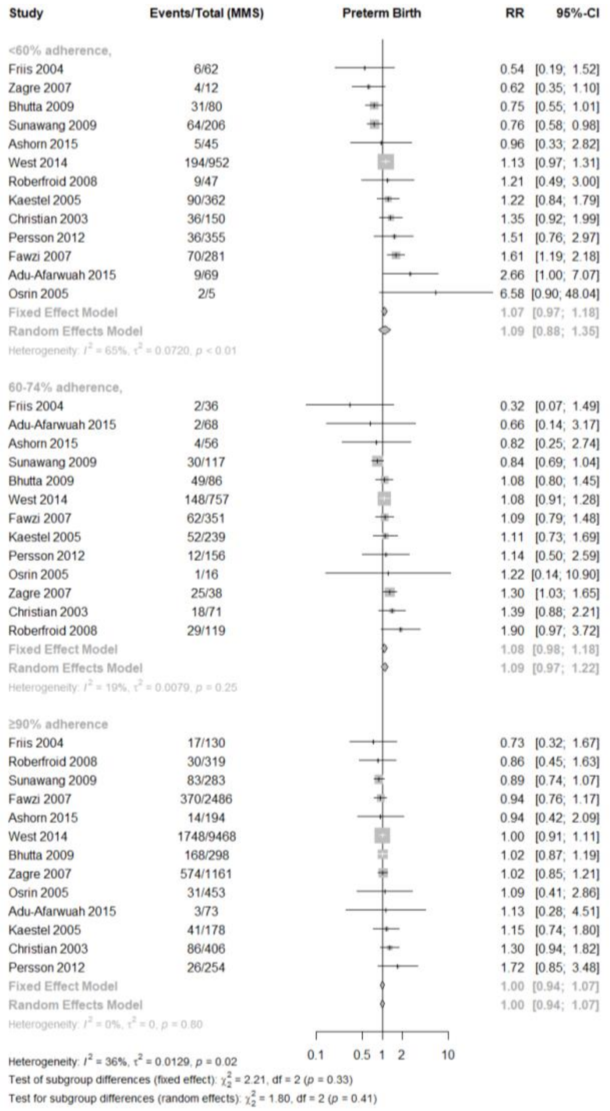

Supplemental Figure 4.13. The effect of MMS on SGA10, stratified by Adherence

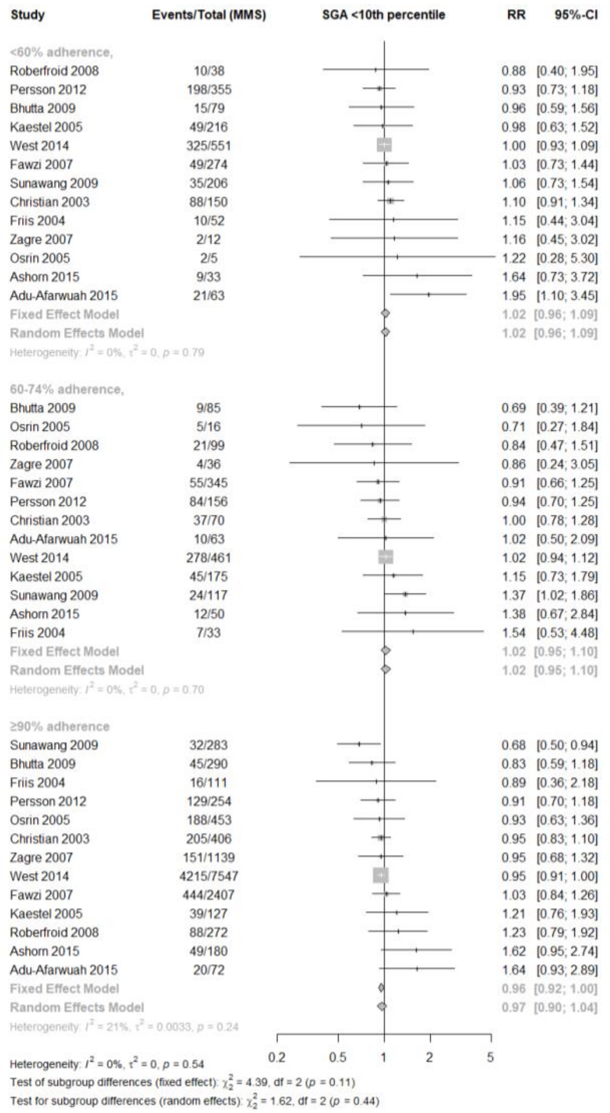

Supplemental Figure 4.14. The effect of MMS on Stillbirth, stratified by Adherence

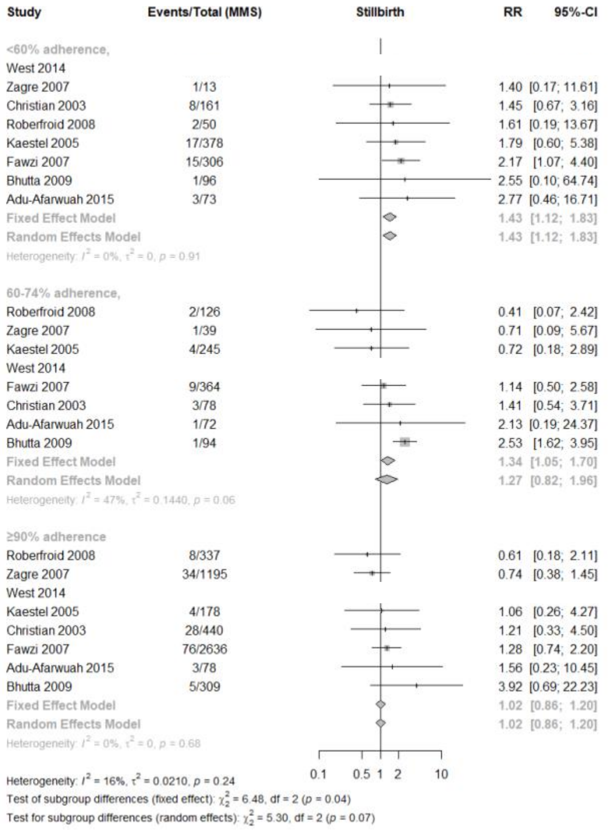

Objective 2 Outcomes stratified by Adherence and Gestational Age

Supplemental Figure 4.15. The effect of MMS on Anemia, stratified by Adherence and Gestational Age

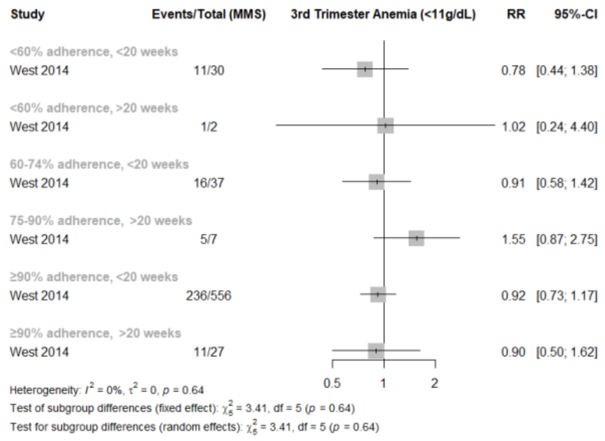

Supplemental Figure 4.16. The effect of MMS on Birthweight Percentile, stratified by Adherence and Gestational Age

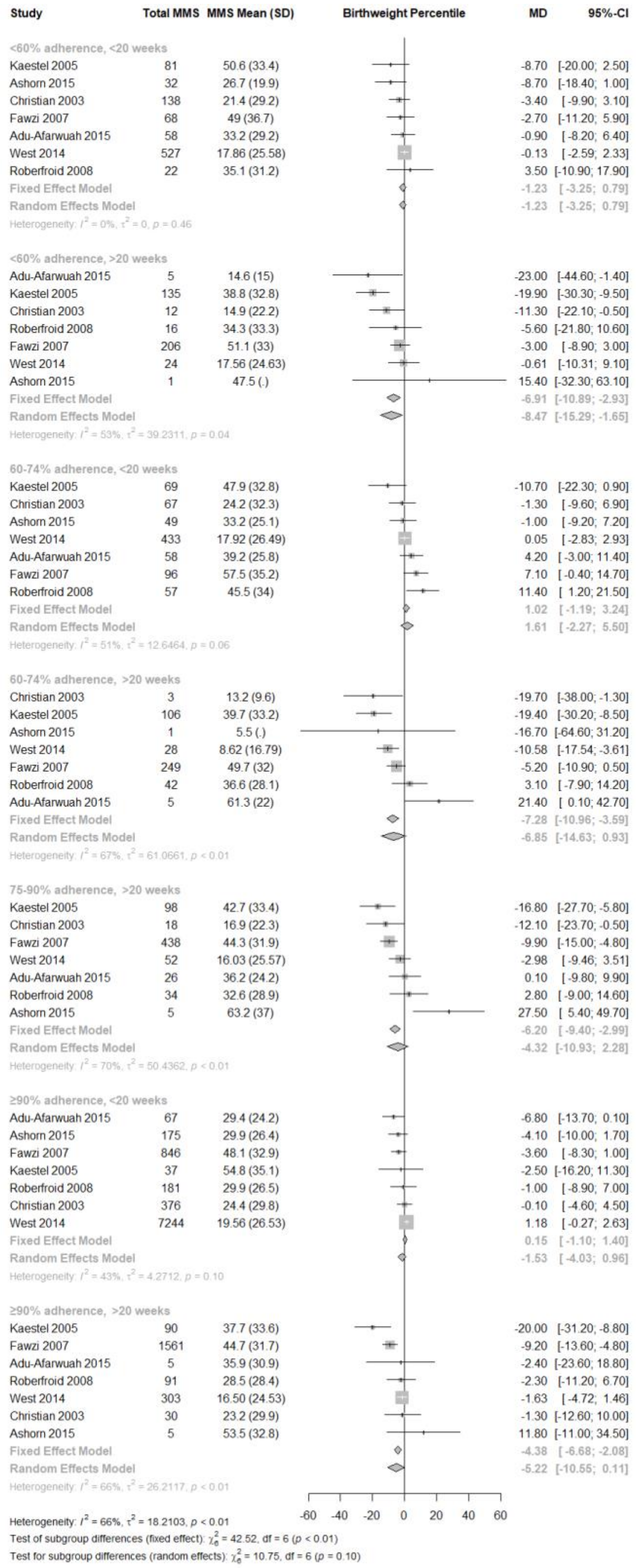

Supplemental Figure 4.17. The effect of MMS on Birthweight, stratified by Adherence and Gestational Age

Study	Total MMS	MMS Mean (SD)	MD	95%-CI
**<60% adherence, <20 weeks**				
Ashorn 2015	32	2849 (486)	-149.00	[-322.00; 24.00]
Fawzi 2007	68	3057 (480)	-127.00	[-259.00; 4.00]
Christian 2003	138	2579 (491)	-124.00	[-227.00; -22.00]
Adu-Afarwuah 2015	58	2909 (555)	-81.00	[-195.00; 34.00]
Kaestel 2005	81	3124 (505)	-48.00	[-210.00; 113.00]
West 2014	543	2567 (390)	14.63	[-22.58; 51.84]
Roberfroid 2008	22	2988 (468)	164.00	[-53.00; 382.00]
Fixed Effect Model			-17.34	[-48.29; 13.60]
Random Effects Model			-55.88	[-126.53; 14.77]
Heterogeneity: I^2 = 62%, τ^2 = 4906.3452, p = 0.01				
**<60% adherence, >20 weeks**				
Christian 2003	12	2396 (503)	-285.00	[-588.00; 18.00]
Adu-Afarwuah 2015	5	2758 (555)	-235.00	[-575.00; 105.00]
Fawzi 2007	206	3120 (478)	-105.00	[-197.00; -13.00]
Kaestel 2005	135	3045 (446)	-73.00	[-221.00; 76.00]
Roberfroid 2008	16	3036 (371)	29.00	[-215.00; 273.00]
West 2014	28	2590 (495)	45.42	[-133.46; 224.30]
Ashorn 2015	1	3169 (.)	237.00	[-612.00; 1086.00]
Fixed Effect Model			-80.08	[-145.67; -14.50]
Random Effects Model			-79.86	[-146.06; -13.66]
Heterogeneity: I^2 = 1%, τ^2 = 65.5625, p = 0.42				
**60-74% adherence, <20 weeks**				
Christian 2003	67	2510 (511)	-204.00	[-330.00; -77.00]
Kaestel 2005	70	3036 (478)	-110.00	[-277.00; 56.00]
Roberfroid 2008	58	2901 (480)	-23.00	[-175.00; 129.00]
Fawzi 2007	96	3164 (492)	-1.00	[-117.00; 115.00]
West 2014	444	2556 (376)	4.78	[-33.70; 43.27]
Adu-Afarwuah 2015	58	3044 (415)	12.00	[-100.00; 125.00]
Ashorn 2015	49	3023 (455)	26.00	[-119.00; 171.00]
Fixed Effect Model			-12.15	[-43.49; 19.19]
Random Effects Model			-32.14	[-89.51; 25.23]
Heterogeneity: I^2 = 47%, τ^2 = 2560.9116, p = 0.08				
**60-74% adherence, >20 weeks**				
Christian 2003	3	2761 (416)	-66.00	[-541.00; 408.00]
Kaestel 2005	106	3109 (496)	-28.00	[-182.00; 127.00]
Fawzi 2007	249	3239 (512)	9.00	[-79.00; 96.00]
Roberfroid 2008	42	2934 (443)	18.00	[-149.00; 185.00]
West 2014	34	2599 (472)	46.53	[-111.58; 204.65]
Ashorn 2015	1	2900 (.)	133.00	[-720.00; 986.00]
Adu-Afarwuah 2015	5	3546 (351)	442.00	[107.00; 777.00]
Fixed Effect Model			24.09	[-37.56; 85.74]
Random Effects Model			28.33	[-43.01; 99.67]
Heterogeneity: I^2 = 12%, τ^2 = 1173.9278, p = 0.34				
**75-90% adherence, >20 weeks**				
Christian 2003	18	2650 (590)	-142.00	[-387.00; 103.00]
Kaestel 2005	99	3055 (469)	-80.00	[-236.00; 76.00]
Fawzi 2007	438	3156 (535)	-64.00	[-142.00; 14.00]
Adu-Afarwuah 2015	26	3027 (401)	1.00	[-154.00; 156.00]
Roberfroid 2008	34	2882 (541)	38.00	[-140.00; 216.00]
West 2014	64	2630 (469)	76.69	[-38.56; 191.95]
Ashorn 2015	5	3361 (612)	347.00	[-47.00; 742.00]
Fixed Effect Model			-18.38	[-69.88; 33.13]
Random Effects Model			-8.99	[-80.59; 62.61]
Heterogeneity: I^2 = 35%, τ^2 = 3042.3601, p = 0.16				
**≥90% adherence, <20 weeks**				
Adu-Afarwuah 2015	67	2959 (374)	-80.00	[-189.00; 29.00]
Christian 2003	376	2684 (431)	-43.00	[-108.00; 22.00]
Fawzi 2007	846	3154 (486)	-39.00	[-110.00; 33.00]
Ashorn 2015	175	2969 (431)	-8.00	[-112.00; 96.00]
Kaestel 2005	38	3079 (550)	-7.00	[-203.00; 188.00]
Roberfroid 2008	181	2899 (459)	10.00	[-109.00; 130.00]
West 2014	7471	2594 (404)	41.66	[20.25; 63.07]
Fixed Effect Model			22.91	[4.30; 41.52]
Random Effects Model			-11.52	[-55.80; 32.76]
Heterogeneity: I^2 = 56%, τ^2 = 1667.7556, p = 0.03				
**≥90% adherence, >20 weeks**				
Ashorn 2015	5	2724 (892)	-334.00	[-739.00; 70.00]
Christian 2003	30	2646 (527)	-103.00	[-267.00; 61.00]
Fawzi 2007	1561	3179 (497)	-41.00	[-109.00; 27.00]
Kaestel 2005	91	3086 (477)	-39.00	[-198.00; 121.00]
Adu-Afarwuah 2015	5	3084 (511)	15.00	[-319.00; 349.00]
Roberfroid 2008	92	2908 (399)	17.00	[-118.00; 152.00]
West 2014	366	2578 (420)	25.74	[-23.98; 75.46]
Fixed Effect Model			-6.24	[-42.35; 29.86]
Random Effects Model			-11.51	[-54.16; 31.14]
Heterogeneity: I^2 = 11%, τ^2 = 399.3716, p = 0.35				

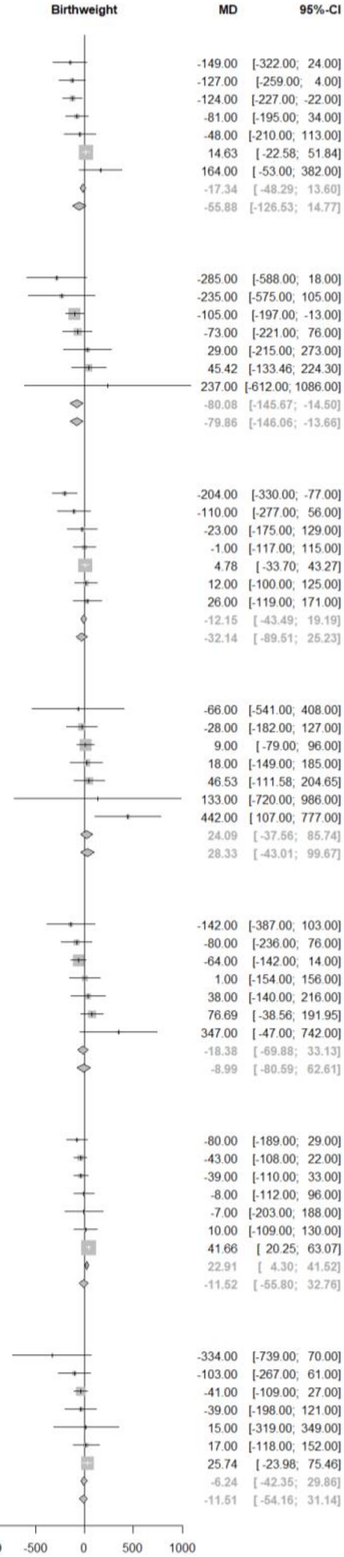

Heterogeneity: I^2 = 43%, τ^2 = 1670.3570, p < 0.01
Test of subgroup differences (fixed effect): χ^2_6 = 14.44, df = 6 (p = 0.03)
Test for subgroup differences (random effects): χ^2_6 = 6.42, df = 6 (p = 0.38)

Supplemental Figure 4.18. The effect of MMS on Continuous Gestation, stratified by Adherence and Gestational Age

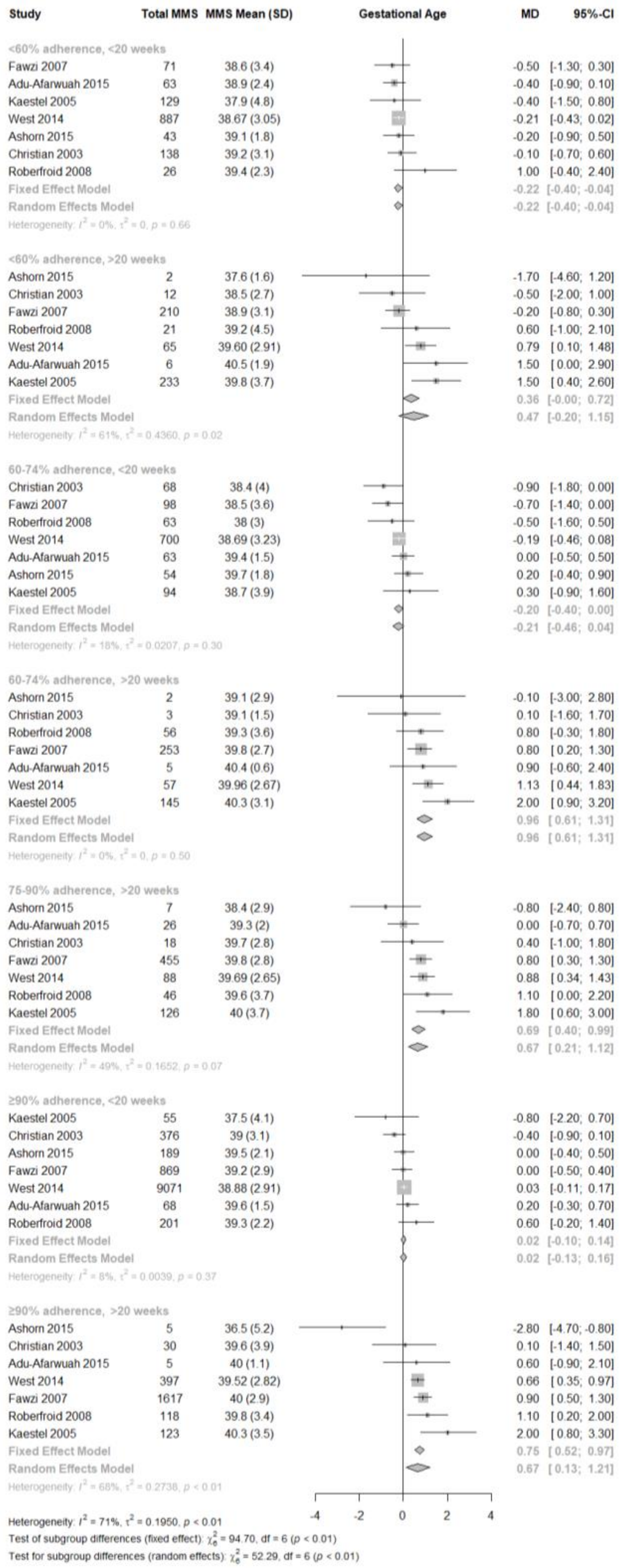

Supplemental Figure 4.19. The effect of MMS on Fetal Death, stratified by Adherence and Gestational Age

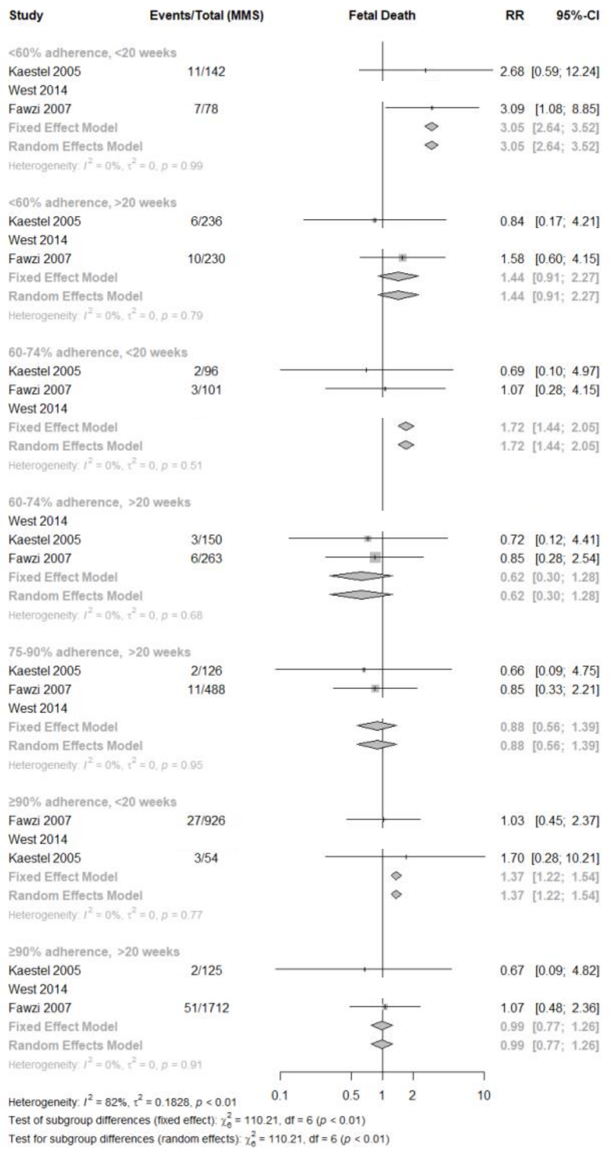

Supplemental Figure 4.20. The effect of MMS on Hemoglobin Continuous, stratified by Adherence and Gestational Age

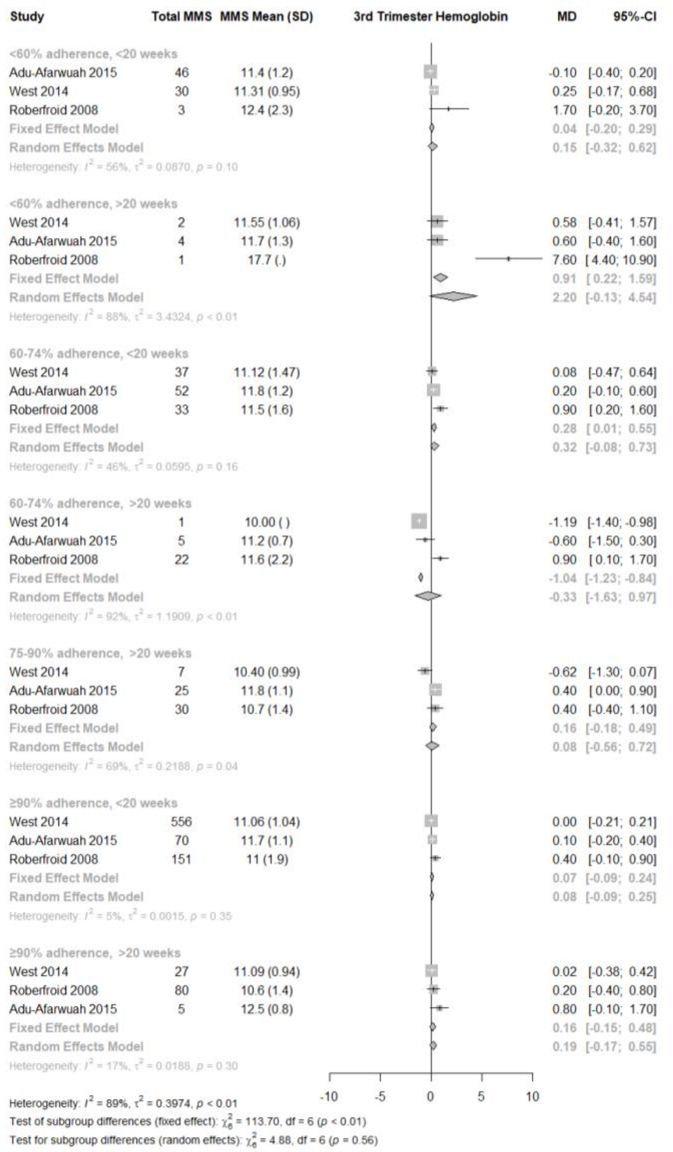

Supplemental Figure 4.21. The effect of MMS on IDA stratified by Adherence and Gestational Age

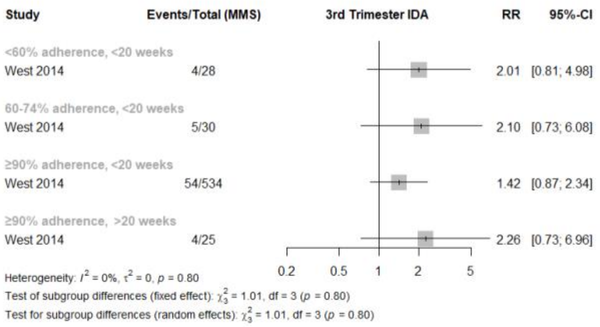

Supplemental Figure 4.22. The effect of MMS on Infant Mortality, stratified by Adherence and Gestational Age

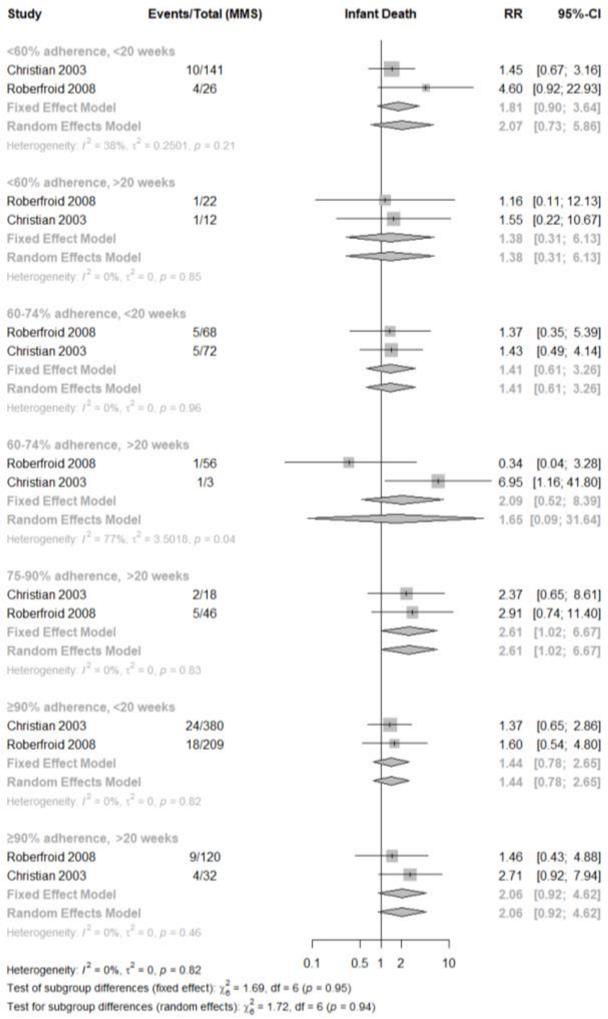

Supplemental Figure 4.23. The effect of MMS on LGA90, stratified by Adherence and Gestational Age

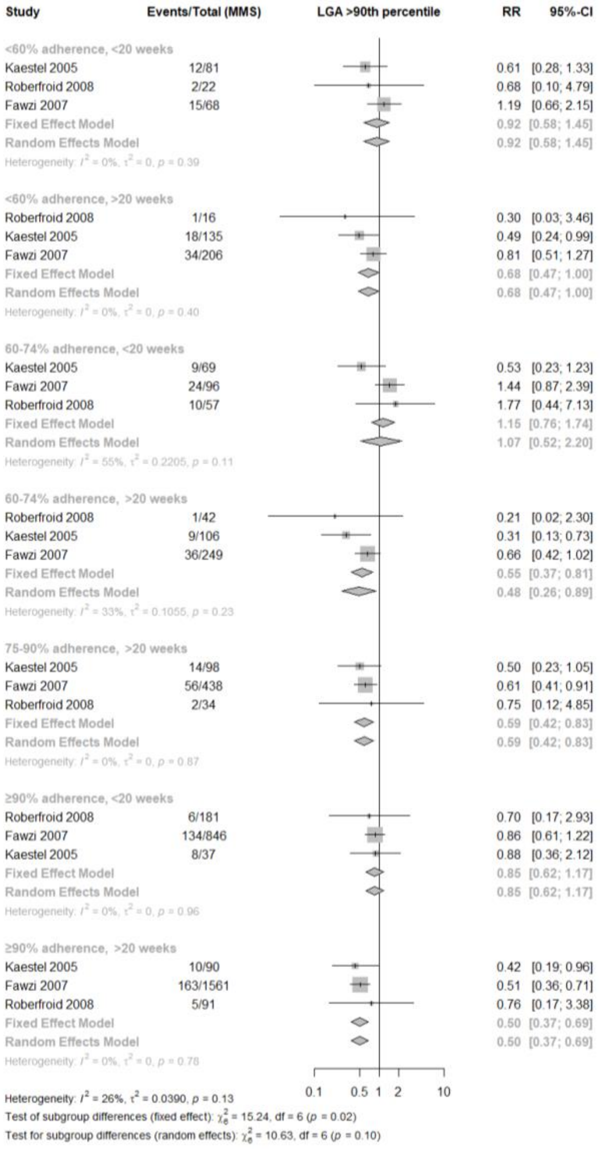

Supplemental Figure 4.24. The effect of MMS on Low Birthweight, stratified by Adherence and Gestational Age

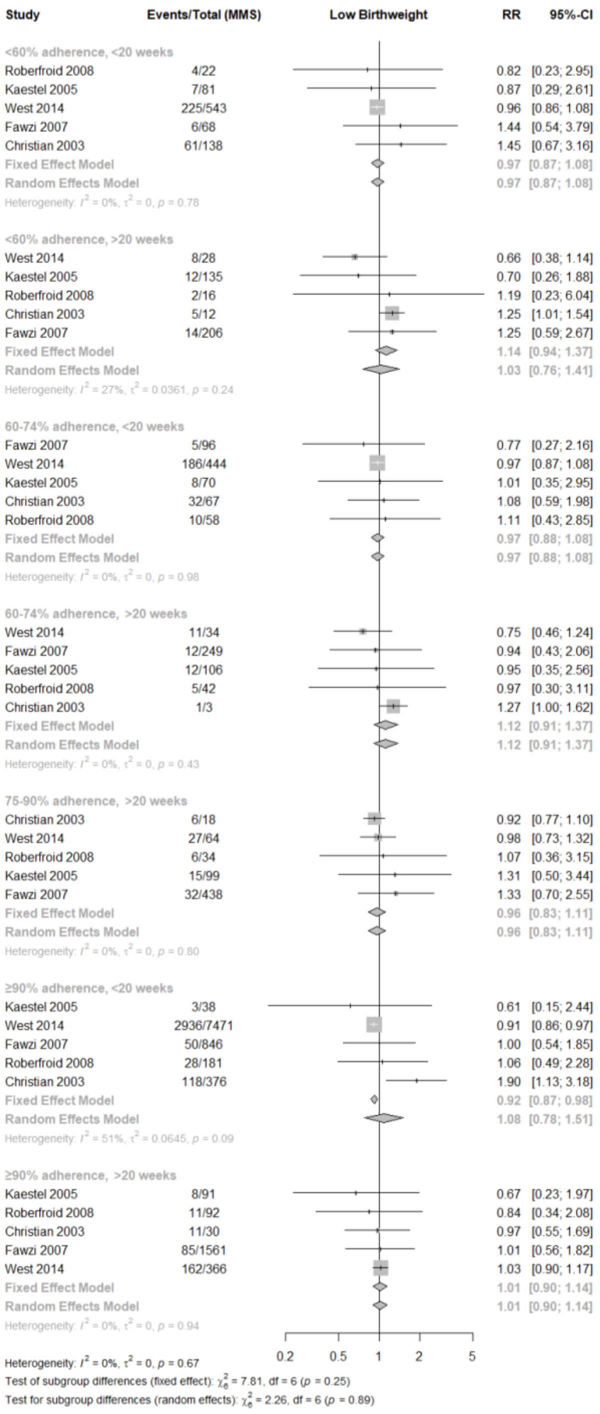

Supplemental Figure 4.25. The effect of MMS on Neonatal Mortality, stratified by Adherence and Gestational Age

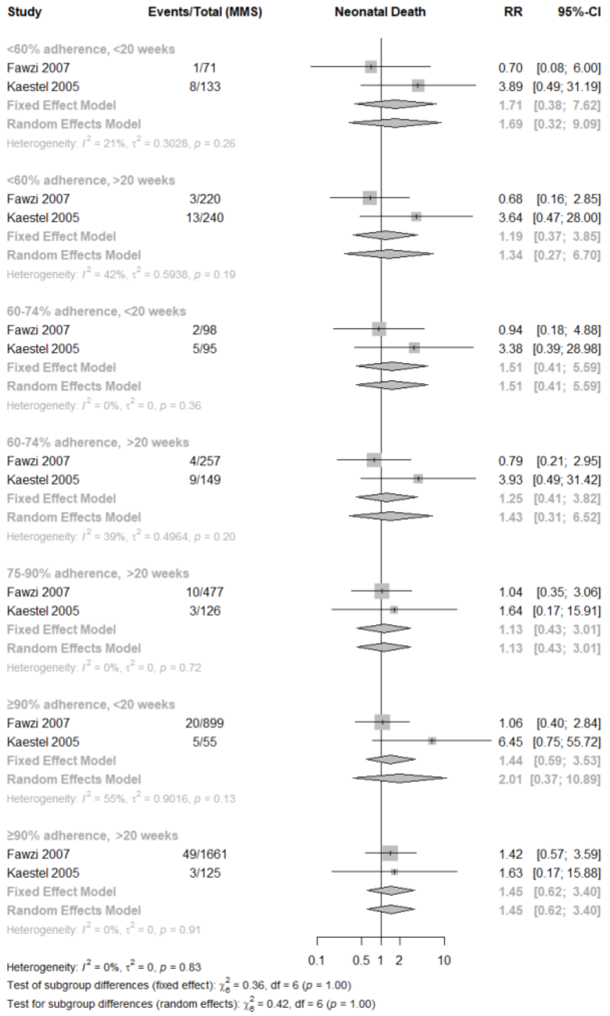

Supplemental Figure 4.26. The effect of MMS on Preterm, stratified by Adherence and Gestational Age

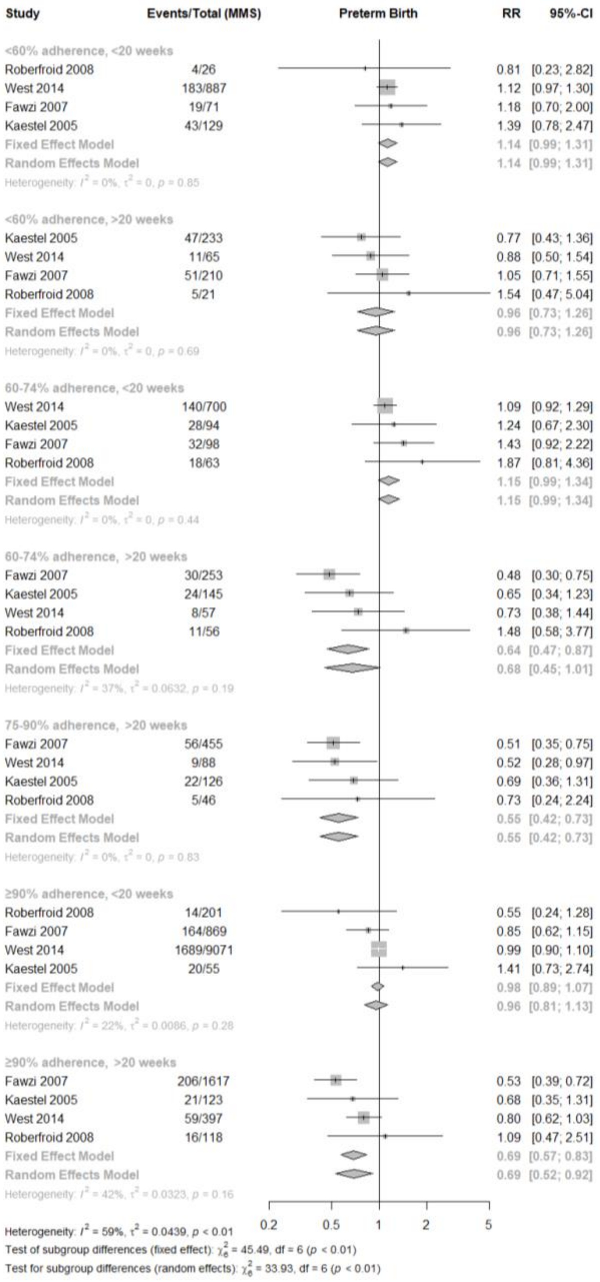

Supplemental Figure 4.27. The effect of MMS on SGA10, stratified by Adherence and Gestational Age

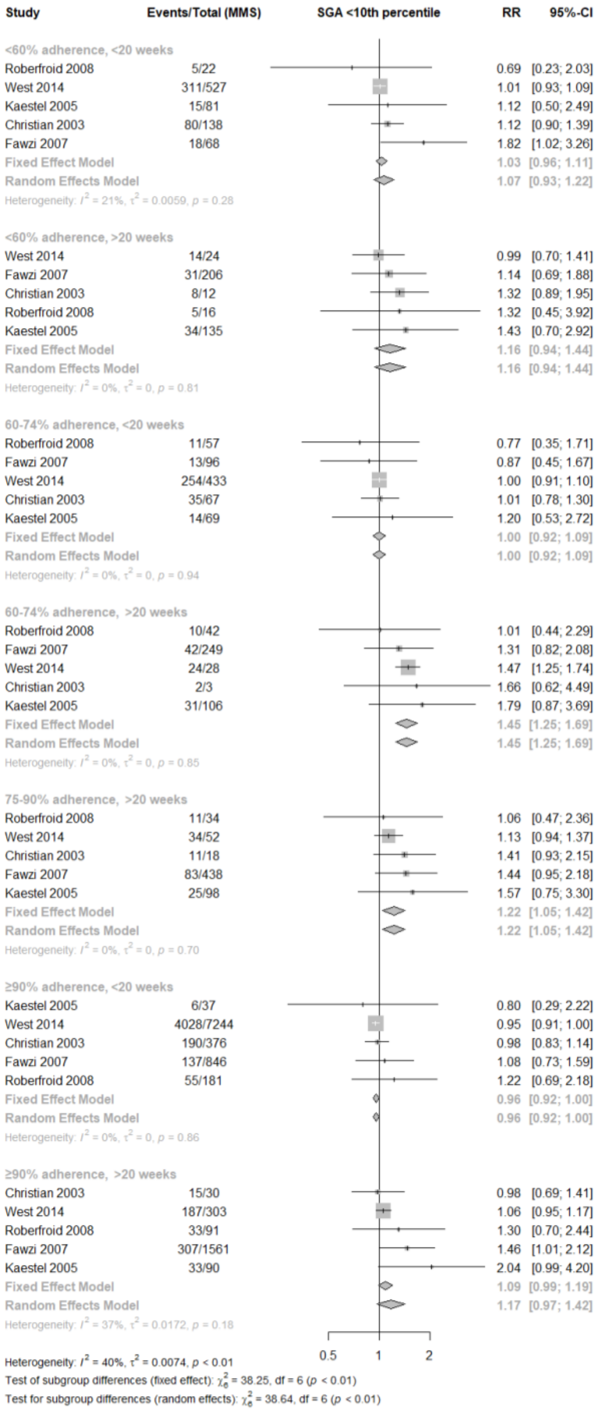

Supplemental Figure 4.28. The effect of MMS on Stillbirth, stratified by Adherence and Gestational Age

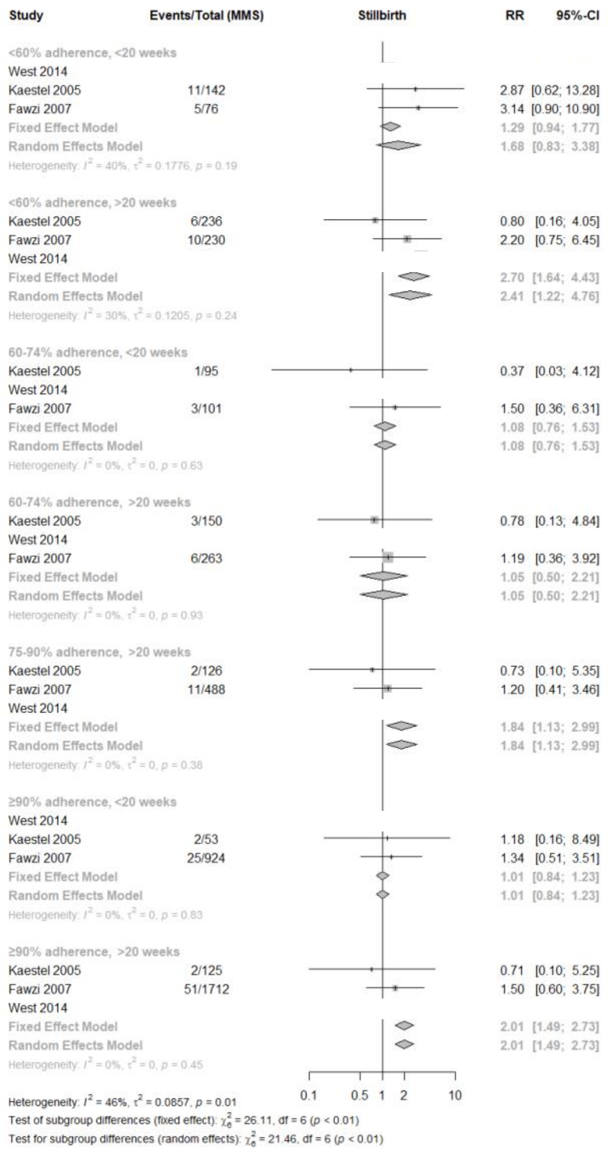

Objective 2 Outcomes stratified by Total Pill Count

Supplemental Figure 4.29. The effect of MMS on Anemia, stratified by Total Pill Count

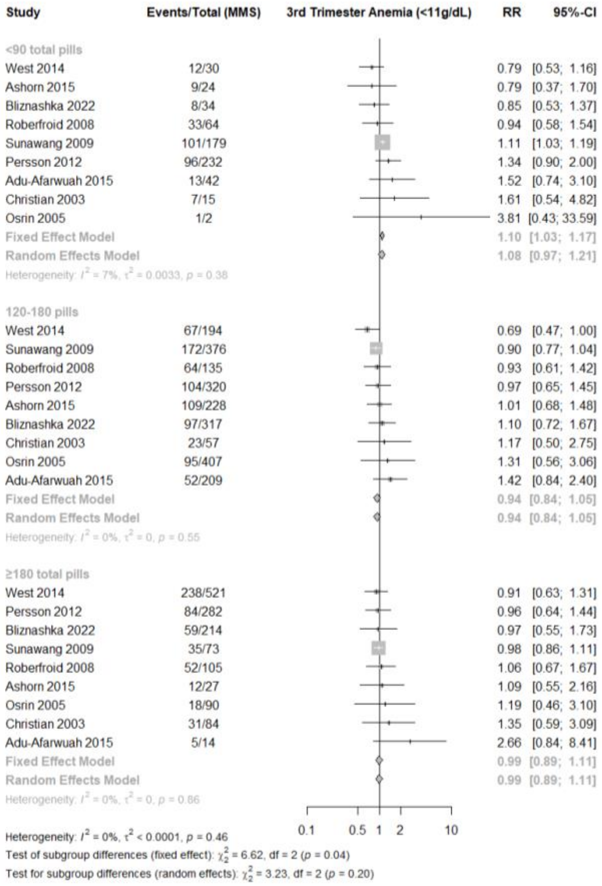

Supplemental Figure 4.30. The effect of MMS on Birthweight Percentile, stratified by Total Pill Count

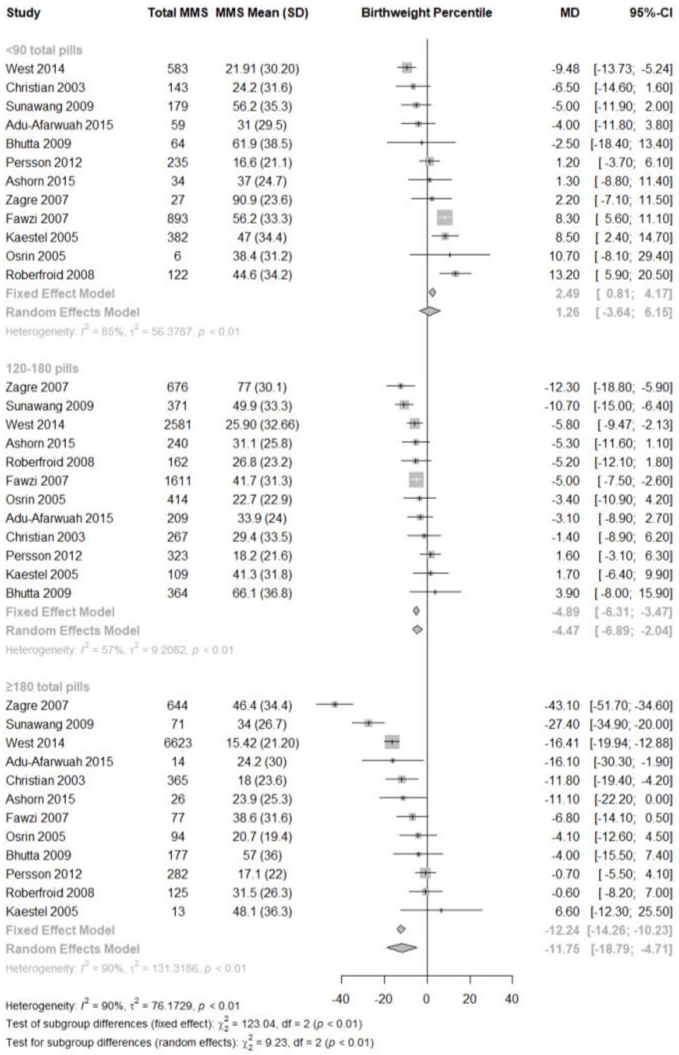

Supplemental Figure 4.31. The effect of MMS on Birthweight, stratified by Total Pill Count

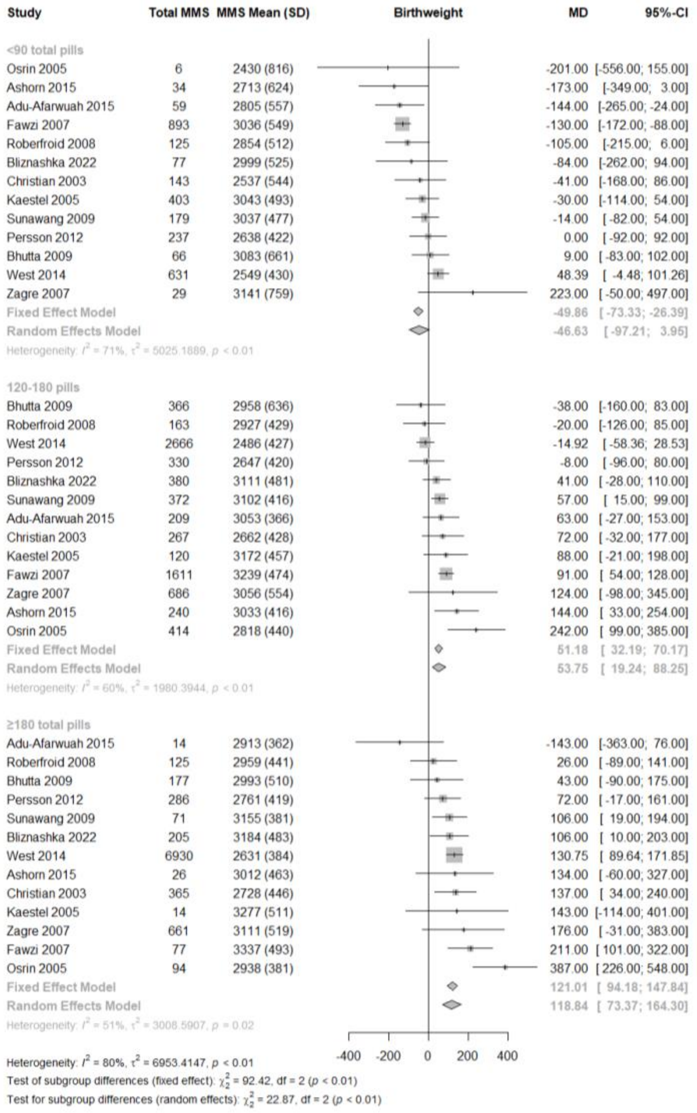

Supplemental Figure 4.32. The effect of MMS on Continuous Gestation, stratified by Total Pill Count

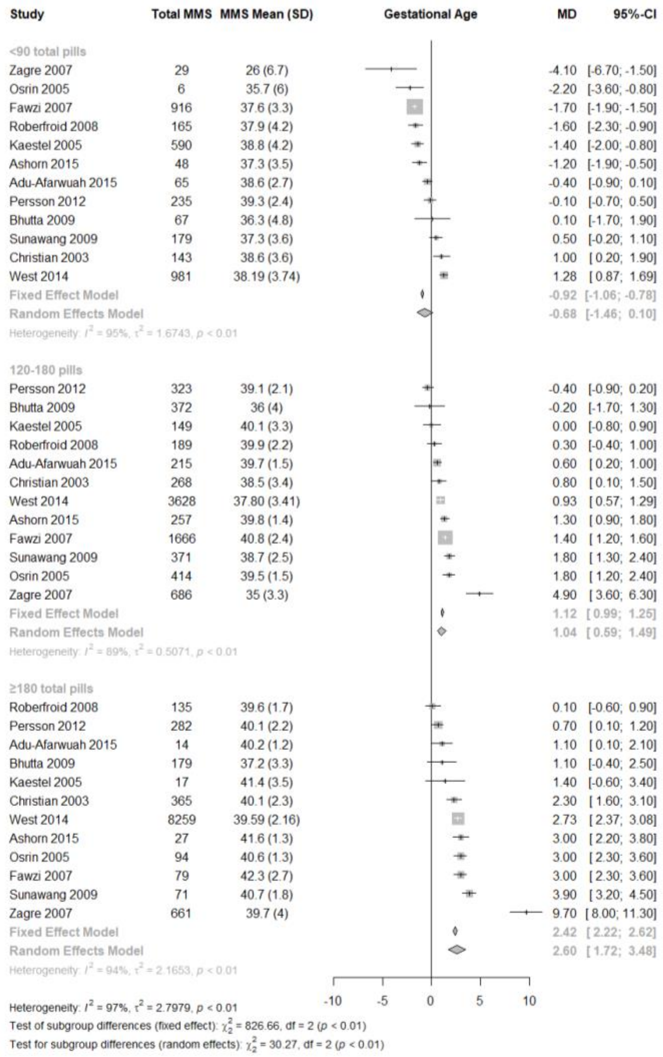

Supplemental Figure 4.33. The effect of MMS on Fetal Death, stratified by Total Pill Count

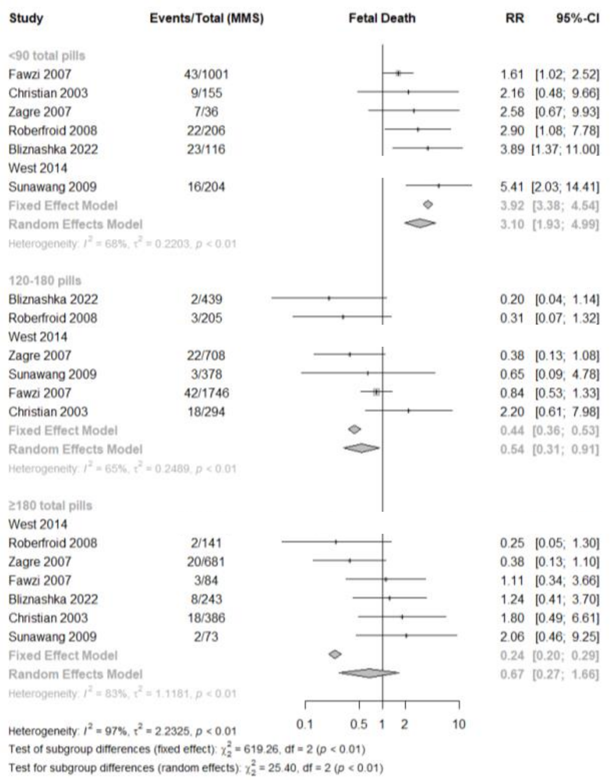

Supplemental Figure 4.34. The effect of MMS on Hemoglobin Continuous, stratified by Total Pill Count

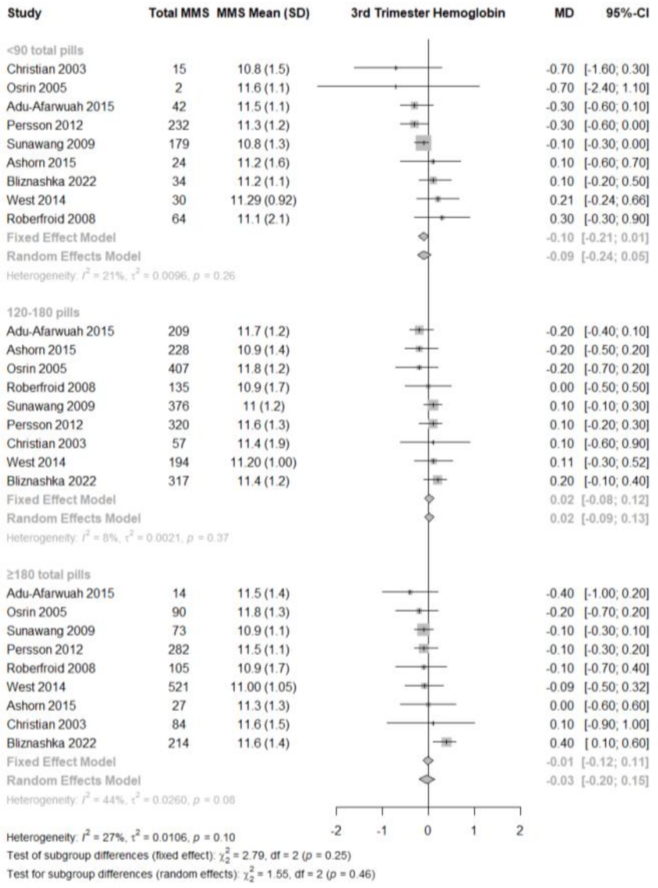

Supplemental Figure 4.35. The effect of MMS on IDA, stratified by Total Pill Count

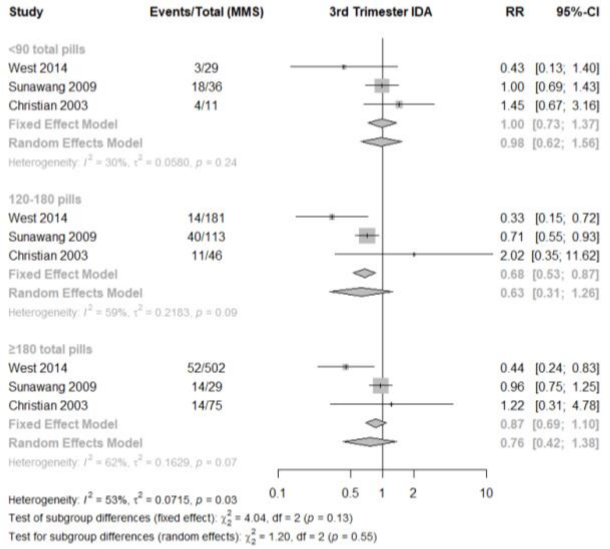

Supplemental Figure 4.36. The effect of MMS on Infant Mortality, stratified by Total Pill Count

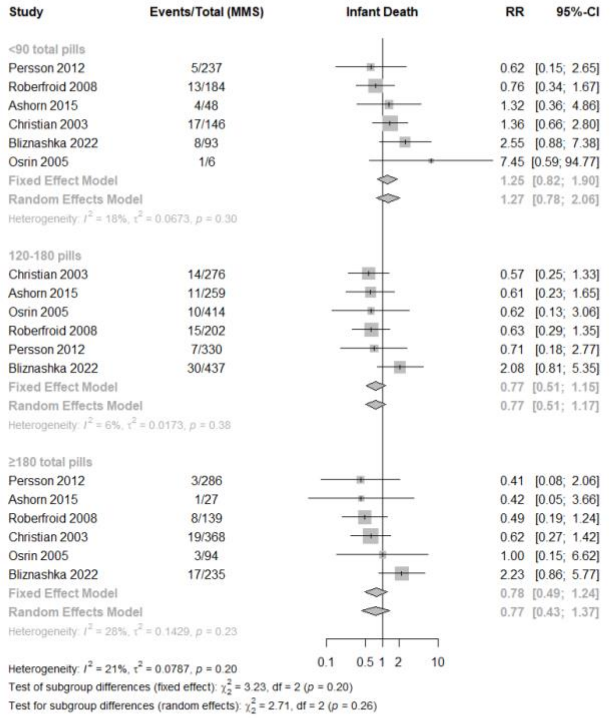

Supplemental Figure 4.37. The effect of MMS on LGA90, stratified by Total Pill Count

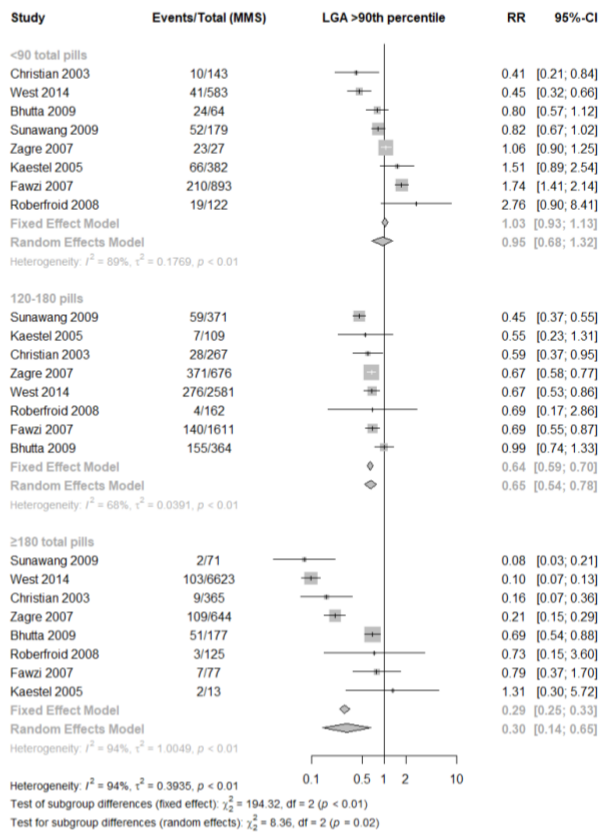

Supplemental Figure 4.38. The effect of MMS on Low Birthweight, stratified by Total Pill Count

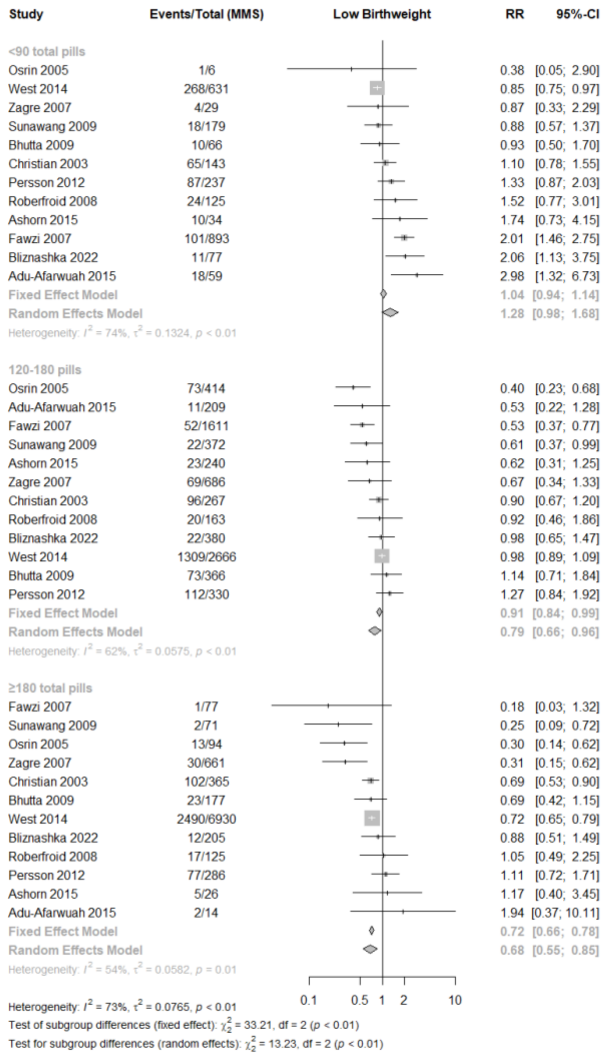

Supplemental Figure 4.39. The effect of MMS on Neonatal Mortality, stratified by Total Pill Count

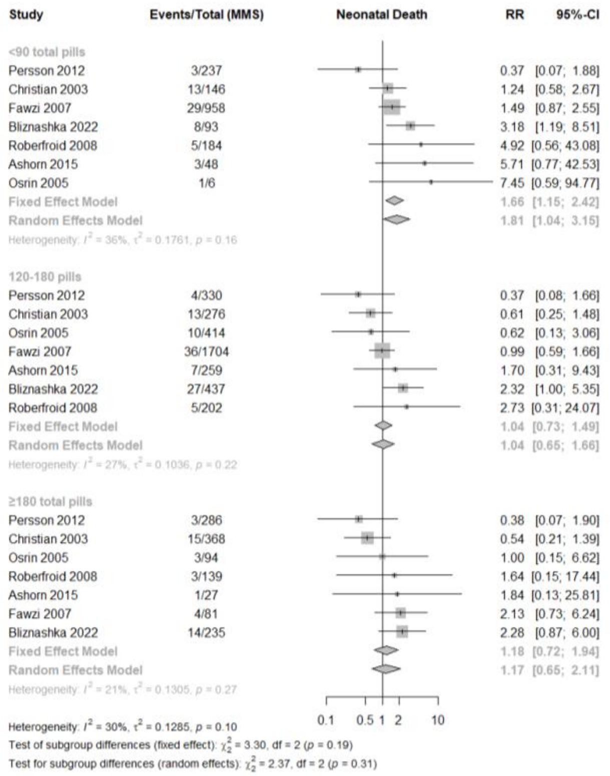

Supplemental Figure 4.40. The effect of MMS on Preterm, stratified by Total Pill Count

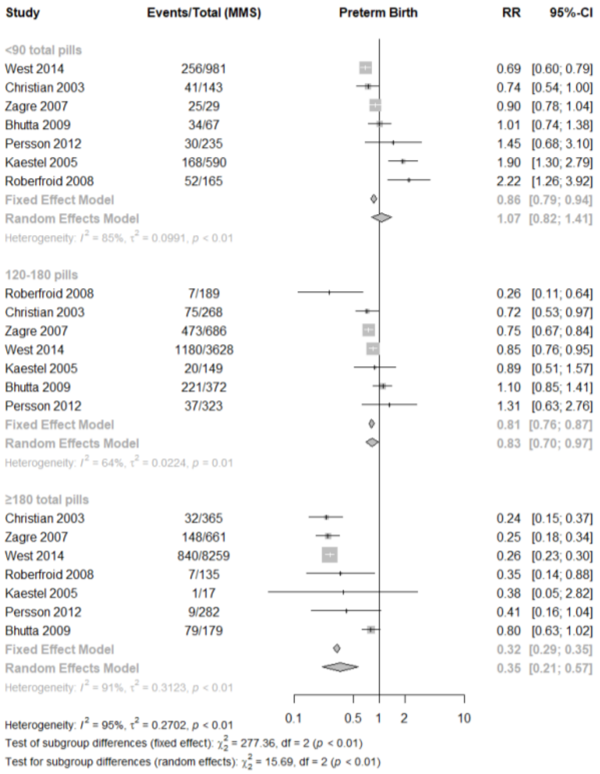

Supplemental Figure 4.41. The effect of MMS on SGA10, stratified by Total Pill Count

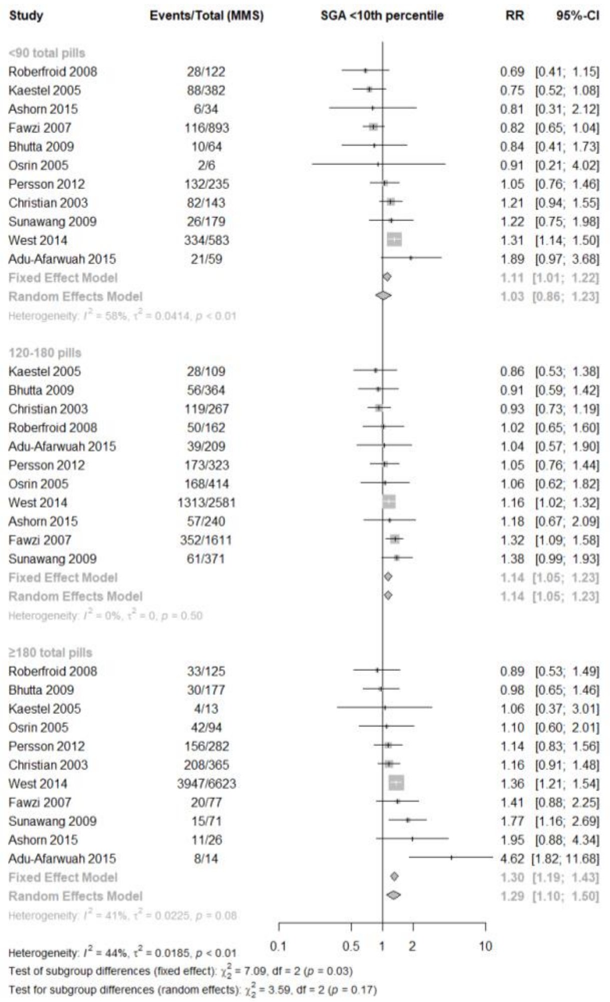

Supplemental Figure 4.42. The effect of MMS on Stillbirth, stratified by Total Pill Count

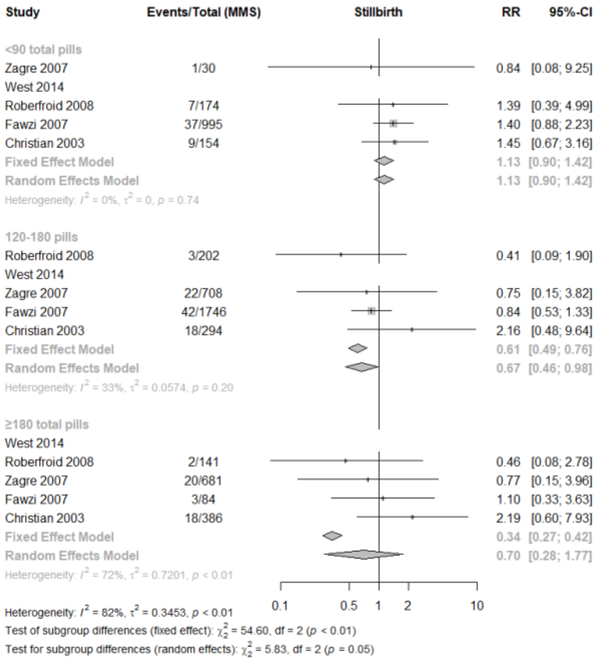

Supplement: Multimedia component 1 [file mmc1.pdf]
